# Potent Neuronal Nicotinamide Adenine Dinucleotide-Boosting Tetrahydroquinoxalines: Structure–Activity Relationships and Early Drug Metabolism and Pharmacokinetics Evaluation

**DOI:** 10.1021/acsmedchemlett.6c00058

**Published:** 2026-03-04

**Authors:** Petra Cuřínová, Melissa Jöe, Filip Cesar, Alan Nicol, Kristián Schwan, Michal Kohout, Carmine Varrichio, Aljona Saleh, Craig E. Wheelock, Gauti Jóhannesson, Václav Eigner, James R. Tribble, Andrea Brancale, Pete A. Williams

**Affiliations:** † Department of Organic Chemistry, 430769 University of Chemistry and Technology in Prague, 166 28 Prague, Czech Republic; ‡ Department of Clinical Neuroscience, Division of Eye and Vision, 27106 St. Erik Eye Hospital, Karolinska Institutet, 171 64 Stockholm, Sweden; § School of Pharmacy and Pharmaceutical Sciences, Cardiff University, CF10 3NB Cardiff, U.K.; ∥ Drug Discovery and Development Platform, Science for Life Laboratory, 751 24 Uppsala, Sweden; ⊥ Department of Clinical Sciences, Ophthalmology, Umeå University, 901 85 Umeå, Sweden; 10 Department of Ophthalmology, University of Iceland, 600169-2039 Reykjavik, Iceland; 7 Unit of Integrative Metabolomics, Institute of Environmental Medicine, Karolinska Institute, 171 77 Stockholm, Sweden; 8 Institute of Physics AS CR v.v.i., 182 00 Prague, Czech Republic; 9 Centre for Eye Research Australia, Royal Victorian Eye and Ear Hospital, 3002 Melbourne, Australia; a Department of Respiratory Medicine and Allergy, Karolinska University Hospital, SE 171 76 Solna, Sweden

**Keywords:** Neurodegeneration; NAD metabolism; NMNAT2; tetrahydroquinoxaline derivatives

## Abstract

We designed and synthesized a series of novel 1,2,3,4-tetrahydroquinoxaline derivatives and evaluated their ability to increase nicotinamide adenine dinucleotide (NAD) levels in primary cortical neurons. Several compounds demonstrated nanomolar potency and enabled the establishment of clear structure–activity relationships (SAR), highlighting key substituents required for activity. Qualitative 3DSAR analysis further identified favorable steric, electrostatic, and hydrophobic features associated with NAD enhancement. Selected lead compounds were assessed for *in vitro* drug metabolism and pharmacokinetics (DMPK) properties, showing good cell permeability and species-dependent metabolic stability in liver microsomes, with improved stability in human systems compared with rodent systems. These findings identify tetrahydroquinoxalines as a promising class of neuronal NAD-boosting agents and provide a strong foundation for further optimization toward neuroprotective drug candidates.

## Graphical abstract

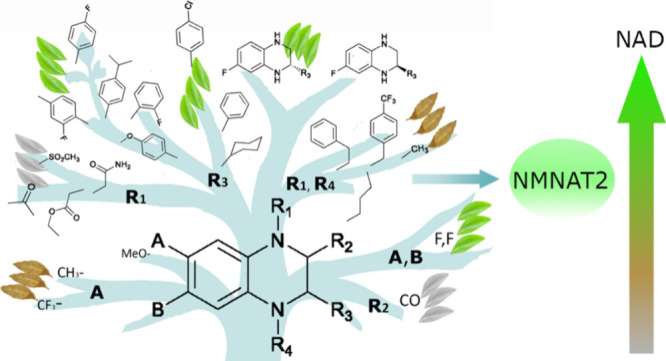

Neurodegenerative disease represents a substantial and growing health and economic burden worldwide, one that is expected to rise further with an increasingly aged population. Current therapies merely alleviate symptoms, providing no means to halt the progression of neurodegeneration or prevent further deterioration.^1^

A promising approach to targeting central mechanisms of neurodegenerative disease is to enhance the production of nicotinamide adenine dinucleotide (NAD).^2,3^ NAD is an essential metabolite that functions as a coenzyme in cellular redox reactions and as a substrate for NAD-consuming enzymes involved in DNA repair, programmed cell death, metabolic regulation, transcription, and inflammation. In neurons, NAD is generated primarily through the NAD salvage pathway.^4,5^ Within this pathway, NAD is first degraded to nicotinamide (NAM) by NADases and then converted to nicotinamide mononucleotide (NMN) by nicotinamide phosphoribosyl transferase (NAMPT). NMN is subsequently transformed to NAD by nicotinamide mononucleotide adenylyl transferases (NMNATs). An elevated NMN:NAD ratio triggers Wallerian degeneration, a form of axon self-destruction occurring distal to injury.^6^ This process begins when NMNAT2 levels fall or its catalytic capacity is reduced, increasing the NMN:NAD ratio and activating SARM1, which initiates the degenerative cascade.^7^

NMNAT2 is a neuron-specific NMNAT isozyme that is essential for neuronal development and survival. Acute depletion of NMNAT2 induces neurite degeneration *in vitro*, and deletion of NMNAT2 in mice results in impaired axon outgrowth and underdeveloped skeletal muscle due to failed innervation.^8–10^ NMNAT2 dysfunction has also been implicated in human disease. Reduced NMNAT2 expression has been reported in the brains of patients with Alzheimer’s disease (AD) and Parkinson’s disease (PD)^11,12^ and in the neuronal layers of the retina in glaucoma patients.^13^ Overexpression of NMNATs provides neuroprotection in multiple *in vitro* and *in vivo* models of neurodegeneration.^14,15^

Previous studies suggest that epigallocatechin gallate (EGCG), a polyphenol found in green tea, can positively modulate NMNAT2.^16,17^ Work from our group has demonstrated that EGCG increases NAD *in vitro* and provides neuroprotection, *in vitro*, *ex vivo*, and *in vivo* models.^18,19^ However, despite its potency, EGCG has poor bioavailability and numerous off-target activities, making it an unsuitable drug candidate. Building on this, we previously transitioned from the EGCG scaffold to simpler, more drug-like chemical frameworks. 1,2,3,4-Tetrahydroquinoxaline-based compounds exhibited strong neuroprotective activity in an *ex vivo* retinal axotomy model. In the present study, we designed a series of novel derivatives of the 1,2,3,4-tetrahydroquinoxaline core to further explore their structure–activity relationship (SAR) and to evaluate their drug metabolism and pharmacokinetics (DMPK) properties *in vitro*. Although NMNAT1, NMNAT3, and several bacterial NMNATs have been structurally characterized, NMNAT2 has so far resisted three-dimensional structural analysis owing to its poor solubility and pronounced tendency to aggregate.^20^ This limitation also represents a substantial obstacle to the development of reliable structure-based biophysical assays. Despite these technical challenges, we adopted a more classical, ligand-based medicinal chemistry approach to establish initial structure–activity relationships. This strategy was further justified by the limited utility of the previously developed homology model at this early stage of design optimization. Using this chemistry-driven approach, we nevertheless identified several compounds with nanomolar activity that represent a promising starting point for the development of a novel class of neuroprotective agents.

The original hit molecule, EGCG, is a complex, labile polyphenol with poor drug properties. Structural modifications of EGCG leading to a 1,2,3,4-tetrahydroquinoxaline compound family provided improved drug-like properties and retained substantial neuroprotective activity in an *ex vivo* retinal axotomy model.^17^ We are reporting here the expansion of this work through the development and assessment of 1,2,3,4-tetrahydroquinoxaline derivatives, examining their structure–activity relationships (SARs) and oxidation stability. Full spectrographic characterization of the compounds reported is included in the Supporting Information file (Figures S1–S153).

The previously reported one-pot synthesis required relatively expensive reductants and produced mixtures of compounds at different reduction levels.^21^ Here, we report a newly developed synthetic protocol that overcomes these limitations (Scheme 1). The target 1,2,3,4-tetrahydroquinoxaline derivatives are obtained through a two-step procedure, separating the cyclization and reduction steps. In the cyclization step, 1,2-phenylenediamine derivatives are condensed with 2-phenyl-2-oxoacetate derivatives in the presence of acetic acid. Overnight stirring in toluene at 100 °C yields the quinoxalinone intermediate (IM,Scheme 1). To avoid material loss during workup due to the low solubility of quinoxalinones, the reaction mixture is simply evaporated to dryness, and the resulting solid is used directly in the reduction step, assuming full conversion and quantitative yield. The reduction is carried out in anhydrous THF using 5 equiv of lithium aluminum hydride at 60 °C overnight. The resulting 1,2,3,4-tetrahydroquinoxalines are readily soluble in the solvents required for workup and isomer separation (Figures S155–S157), providing the target compounds in high yields while avoiding labor-intensive purification. Compounds **3** and **4** form as isomers in an approximately 1:1 ratio because the electronic effect of fluorine in the starting 4-fluoro-1,2-phenylenediamine does not influence the nucleophilicity of the 2-amino group, and the initial Schiff base formation is not regioselective. After the two-step synthesis, these isomers are separated via flash chromatography using an ethyl acetate/cyclohexane gradient, with the **3**-series eluting first and the **4**-series eluting later (Scheme 2). Small amounts of fully aromatic side products (quinoxalines) also appear as earlier-eluting compounds. During the synthesis of the **5**-series, the **5a** intermediate exhibited unexpected behavior: the fluorine atoms of the CF_3_ group were gradually replaced by nucleophilic hydride from LiAlH_4_, yielding **5b** instead of **5a**. Thus, a non-nucleophilic borohydride was used for this step. For **5c**, the methoxy group on 4-methoxy-1,2-phenylenediamine favors Schiff base formation at the 2-amino group, giving isomers in a 4:1 ratio; therefore, after reduction, mainly **5c** is isolated, with only trace amounts of the alternative isomer.

**Scheme 1:**
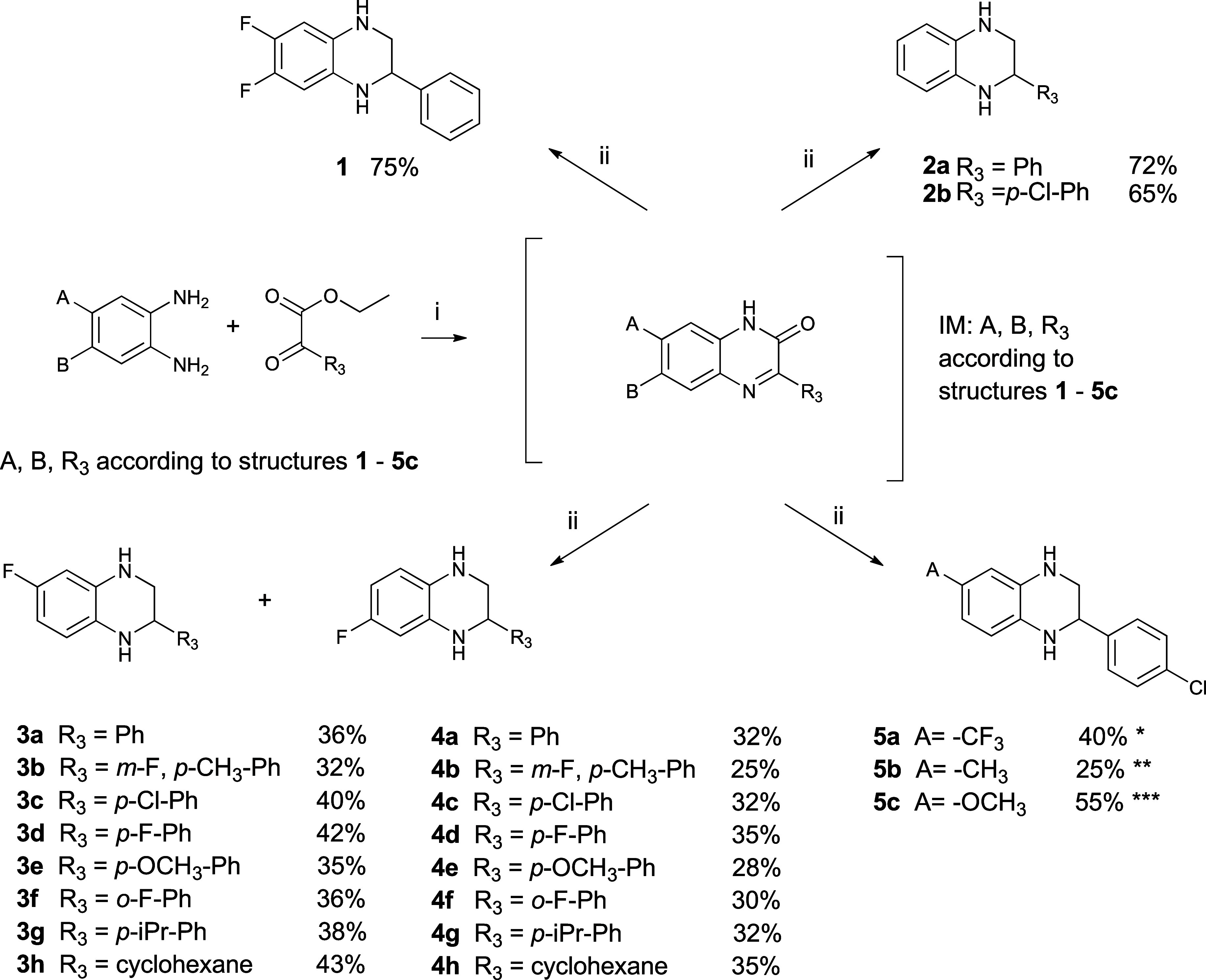
Synthesis of 1,2,3,4-Tetrahydroquinoxalines^a^

a (i) 1 equiv of acetic acid in toluene, 100 °C overnight and (ii) 5 equiv of LiAlH_4_ in THF overnight. The yields are given for a two-step procedure. Compounds **3** and **4** are formed in one reaction as isomers; therefore the % of **3** + % of **4** is at maximum 100%. *For the compound **5a**, BH_3_ solution in THF was used as a reductant, as in the presence of LiAlH_4_ only the formation of **5b** was observed after nucleophilic attack of hydride to the CF_3_ group. **Obtained as a side product from LiAlH_4_ reduction of **5a-IM**. *Due to the electron effects of the −OCH_3_ group, the formation of **5c** was favored, and only traces of an isomer were observed.

**Scheme 2:**
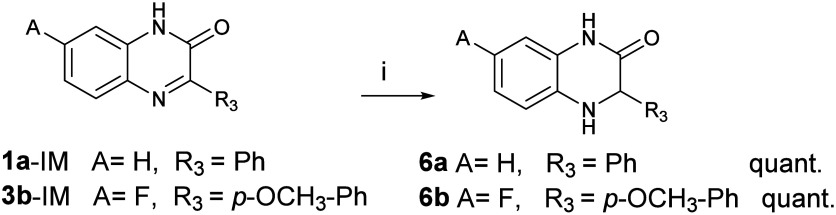
Synthesis of 1,3,4-Trihydroquinoxalin-2-ones^a^

a (i) 8 bar H_2_, 0.1 mass equiv of 10% Pd/C, MeOH, rt, overnight.

To obtain partially reduced compounds, palladium-catalyzed hydrogenation at 8 bar of hydrogen was employed. Under these conditions, the Schiff base was readily reduced, while the amide group remained intact. The products were obtained in quantitative yields.

Selected compounds were *N*-alkylated to 1) investigate the impact of *N*-alkylation on biological activity and 2) to stabilize the molecules against aromatization, which is a major decomposition pathway for tetrahydroquinoxalines (Scheme 3).

**Scheme 3:**
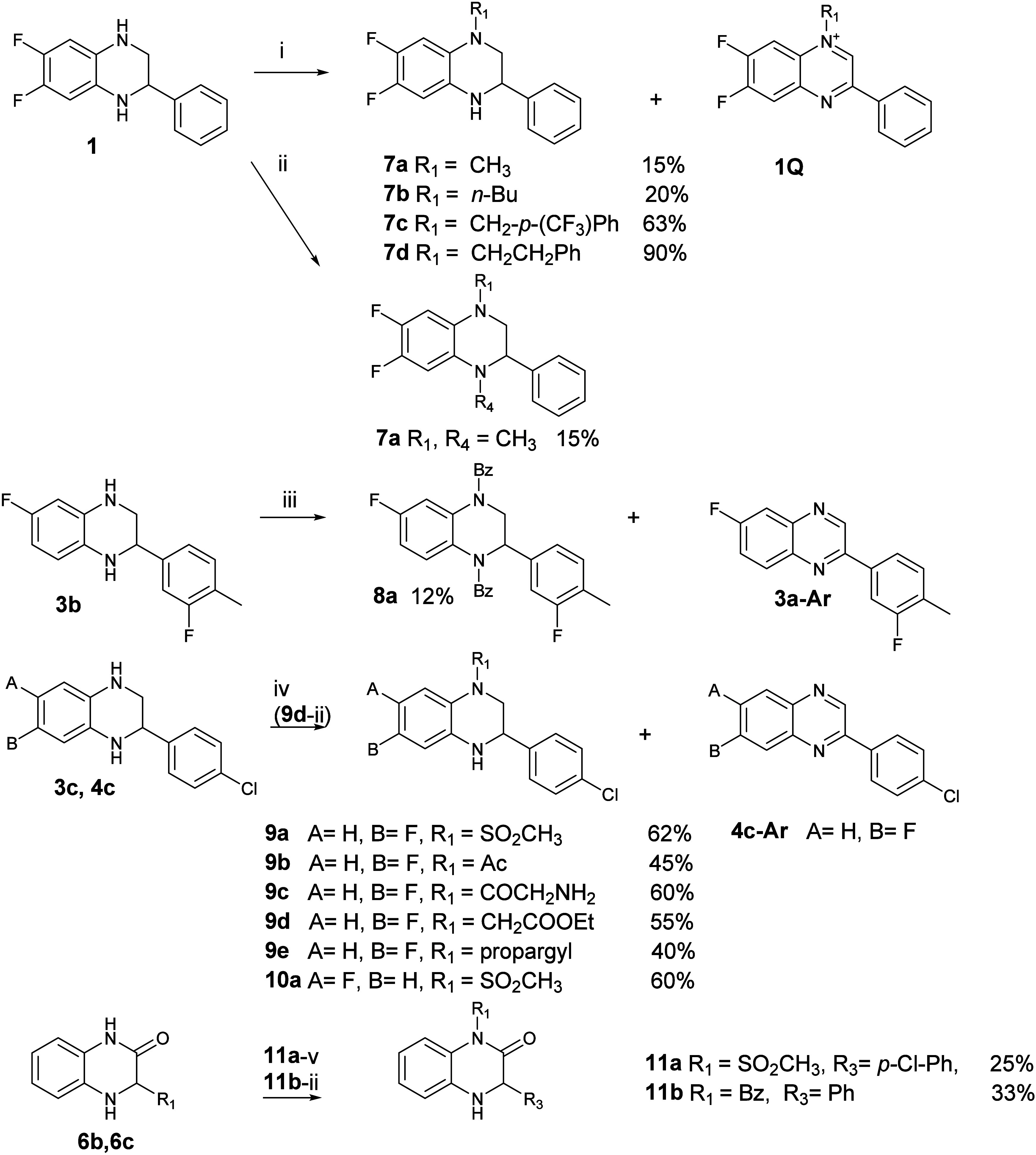
*N-*Alkylation of 1,2,3,4-Tetrahydroquinoxalines and 1,3,4-Trihydroquinoxalin-2-ones^a^

a (i) R-X 1 equiv, NaH 1 equiv, DMF, 60 °C overnight, (ii) R-X excess, NaH 5 equiv, rt overnight, (iii) BzBr, 5 equiv, NaH 5 equiv, DMF, 60° C overnight, (iv) R-Cl 1 equiv, DIPEA 1.2 equiv, DCM, rt, overnight, (v) R-CHO 1 equiv, ZnCl_2_ 1.5 equiv, NaCNBH_4_ 1.5 equiv, MeOH.

Alkylation reactions were carried out in DMF, where the nucleophilicity of the starting 1,2,3,4-tetrahydroquinoxalines was increased by deprotonation with NaH. Once gas evolution ceased (after approximately 5 min), the relevant alkyl halides were added, and the mixture was heated overnight, giving products in variable yields. Product yield depended strongly on the nature of the R group, with susceptibility to aromatization and formation of quinoxalinium salts (**1Q**) increasing in the order: propargyl < CH_2_–*p*-(CF_3_)­Ph < benzyl < methyl < *n*-butyl. Pure alkylated products were obtained by chromatographic removal of the salts (S158–S160).

Derivative **7b** was isolated as a 1:1 mixture of the free amine and its quinoxalinium salt, and it was reduced back to **7b** using NaBH_4_ in methanol. For **7d**, aromatization was followed by styrene elimination and dealkylation; this issue was resolved by introducing the substituent *via* reductive amination using 2-phenylacetaldehyde. Double alkylation, due to high base loading, consistently led to aromatization (**3b-Ar**, **4c-Ar**).

In contrast, acylation and sulfonylation proceeded smoothly and substantially improved the oxidative stability (S161). However, aromatic side product **4c-Ar** always formed upon base addition. Regioselectivity in both alkylation and acylation/sulfonylation was governed by steric effects, making double substitution challenging. To alkylate or sulfonylate the more hindered nitrogen atom, derivatization was performed on trihydroquinoxalinone **6a**, in which the less hindered nitrogen is amidic and non-nucleophilic.

The products were readily obtained and easily crystallized, and the structure of **11b** was confirmed by X-ray diffraction (Figure S154). Selected derivatives, **4a** and **4b**, were separated into individual enantiomers using chiral HPLC on an amylose column (Figures S162–S164). Furthermore, crystals suitable for X-ray analysis were obtained from ethyl acetate, and the absolute configuration of the fast-eluting **4a** enantiomer was confirmed as *R*. The slow-eluting enantiomer was crystallized and assigned the *S* configuration (Figure 1). The configurations of the **4b** enantiomers were assumed based on structural similarity and the same elution order (fast-eluting *R*, slow-eluting *S*).

**Figure 1:**
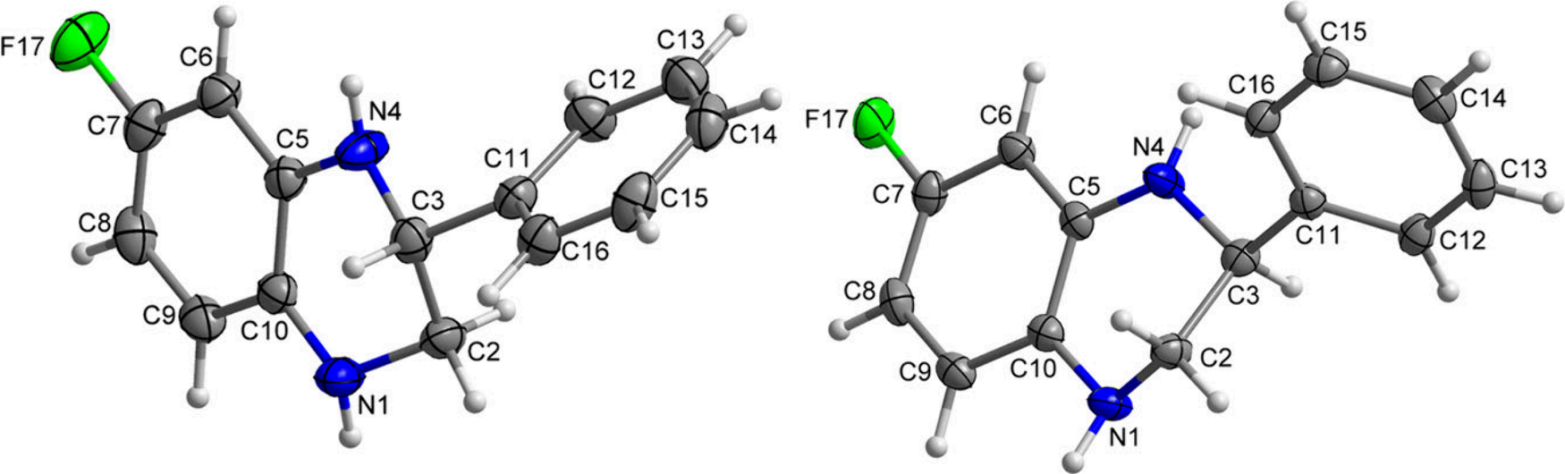
Crystal structures of *R*-**4a** (left) and *S*-**4a** (right).

During synthetic procedures involving base, substantial aromatization was observed as a side reaction. Aromatization was also detected when final compounds were left in DMSO solution for extended periods without freezing. Therefore, we examined the stability of three representative compounds: **3c** (NH nonderivatized), **7d** (*N*-alkylated), and **9a** (*N*-sulfonylated). Each sample was dissolved in acetonitrile and treated with either 1 M HCl or NaH (60% in paraffin). UPLC measurements were taken before treatment, immediately after addition, and after 24 h. All samples exposed to NaH after 24 h showed complete decomposition of the starting material (Table 1, Figure S165).

**Table 1:** Susceptibility of **3c**, **7d**, and **9a** to Aromatization^a^

Cpd	**3c**			**7d**			**9a**		
[%]	**3c**	3c-Ar	other	**7d**	**3c-Ar**	**7d-Ar**	**9a**	3c-Ar	other
No treatment	97	3	0	87	7	6	97	3	0
HCl, immediate	86	14	0	87	7	6	97	3	0
HCl, 24 h	77	23	0	87	7	6	97	3	0
NaH, immediate	0	100	0	40	7	35*	33	43	18**
NaH, 24 h	0	55	45**	0	0	100**	0	63	37**

a *+9% of **7d** with one double bond present. **undefined products.

Sample **3c**, which contains nonderivatized NH groups, was relatively stable under acidic conditions and after 24 h contained 22% of aromatic derivative. In contrast, the addition of NaH caused immediate full conversion to the aromatic analogue. For **7d**, partial stabilization under acidic conditions was observed, but exposure to NaH unexpectedly resulted in the formation of aromatic ammonium ions; nevertheless, 40% of **7d** remained nonaromatic. Conversion of the amine into a sulfonamide in **9a** was expected to prevent aromatization completely. Indeed, **9a** remained stable under acidic conditions for 24 h. However, NaH caused sulfonamide hydrolysis followed by aromatization of the resulting NH-containing product. Immediately after NaH addition, the sample contained 43% of aromatic product, 33% of unreacted **9a**, and various undefined species.

To assess the NAD boosting potential of the newly generated compounds, cortical neurons were incubated for 2 h through a concentration series (50, 500, and 5 μM) (Table 2; Figure S158). To test the specificity of the compounds to the NAD salvage pathway, the upstream enzyme NAMPT was inhibited by coincubation with FK866 (a potent NAMPT inhibitor). In these assays, an ∼2-fold increase is likely to be the physiological maximum for these cells. Based on current research, there appears to be a physiological NAD ceiling in neurons at approximately 200% of baseline levels, even when supplemented with NAD precursors like nicotinamide or nicotinamide riboside. This ceiling likely reflects the kinetic limitations of NAD biosynthetic enzymes, particularly NMNAT2, which follows standard/Michaelis–Menten saturation kinetics with defined maximum velocities (*K*_m_ of 82 μM and *V*_max_ of 4.20 nmol/min [*N.B.* in mitochondrial preps], indicating clear saturation limits). Clinical studies consistently show that NAD increases the plateau at 1.2- to 1.6-fold above baseline with various precursor supplementations, and importantly, this 200% threshold appears sufficient to provide complete neuroprotection in retinal and optic nerve models. The ceiling suggests there is an optimal therapeutic window for NAD enhancement, with further increases beyond this level offering diminishing returns and potentially limiting the effectiveness of higher-dose NAD precursor strategies.

**Table 2:**
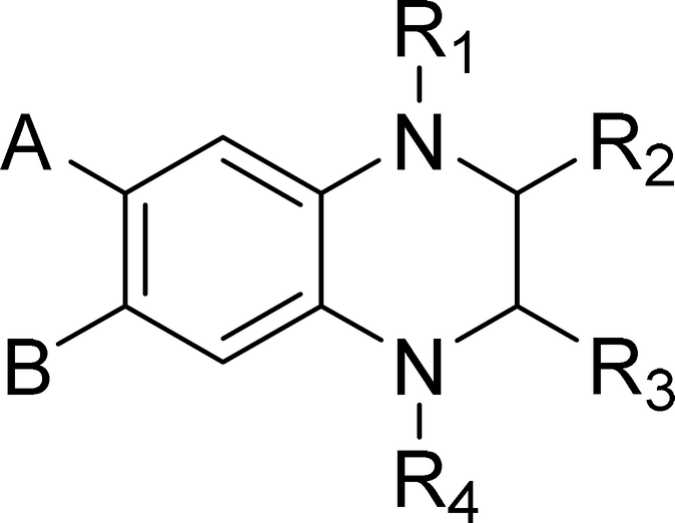
Biological Results^a^

Cpd	A; B	R_1_; R_4_	R_2_	R_3_	50 nM	500 nM	5 μM
**1**	F; F	H; H	H	Ph	0.94	2.09	1.95
**3a**	F; H	H; H	H	Ph	1.25	1.97	2.00
**3b**	F; H	H; H	H	*m*-F, *p*-CH_3_-Ph	1.05	1.82	1.82
**3c**	F; H	H; H	H	*p*-Cl-Ph	1.38	2.01	2.00
**3d**	F; H	H; H	H	*p*-F-Ph	1.17	2.05	2.00
**3e**	F; H	H; H	H	*p*-OMe-Ph	1.29	1.16	2.02
**3f**	F; H	H; H	H	*o*-F-Ph	1.14	1.85	1.89
**3g**	F; H	H; H	H	*p*-iPr-Ph	1.39	1.71	1.88
**3h**	F; H	H; H	H	Cyclohex.	1.03	1.59	1.96
*R* **-4a**	H; F	H; H	H	Ph	1.12	1.93	1.91
*S* **-4a**	H; F	H; H	H	Ph	1.08	1.76	1.85
*R* **-4b**	H; F	H; H	H	*m*-F, *p*-CH_3_-Ph	1.43	1.95	2.02
*S* **-4b**	H; F	H; H	H	*m*-F, *p*-CH_3_-Ph	1.33	1.92	1.91
**4c**	H; F	H; H	H	*p*-Cl-Ph	1.32	1.88	1.81
**4d**	H; F	H; H	H	*p*-F-Ph	1.08	1.07	1.98
**4e**	H; F	H; H	H	*p*-OMe-Ph	1.09	0.95	1.95
**4f**	H; F	H; H	H	*o*-F-Ph	1.26	1.95	2.04
**4g**	H; F	H; H	H	*p*-iPr-Ph	1.31	2	2.01
**4h**	H; F	H; H	H	Cyclohex.	0.77	1.59	1.96
**5a**	CF_3_; H	H; H	H	*p*-Cl-Ph	-	1.2	-
**5b**	CH_3_; H	H; H	H	*p*-Cl-Ph	-	1.3	-
**5c**	OMe; H	H; H	H	*p-*Cl-Ph	-	1.3	-
**6a**	H; H	H; H	=O	Ph	-	-	-
**6c**	H; F and F; H	H; H	=O	*p*-OMe-Ph	-	1.5	-
**7a**	F; F	CH_3_; H	H	H	-	1.2	-
**7b**	F; F	*n*-Bu; H	H	H	0.82	1.5	-
**7c**	F; F	CH_2_Ph*-p*-CF_3_; H	H	H	0.86	1.5	-
**7d**	F; F	Et-Ph; H	H	H	0.76	1.5	-
**7e**	F; F	Me; Me	H	H	-	-	1.6
**8a**	F; H	Bz; Bz	H	*m*-F, *p*-CH_3_-Ph	-	-	1.4
**9a**	H; F	SO_2_CH_3_; H	H	*p*-Cl-Ph	-	-	1.4
**9b**	H; F	Ac; H	H	*p*-Cl-Ph	-	-	not active
**9c**	H; F	COCH_2_NH_2_; H	H	*p*-Cl-Ph	-	-	not active
**9d**	H; F	CH_2_COOEt; H	H	*p*-Cl-Ph	-	-	1.3
**9e**	H; F	propargyl; H	H	*p*-Cl-Ph	-	1.5	-
**10a**	F; H	SO_2_CH_3_; H	H	*p*-Cl-Ph	-	1.25	-
**11a**	H; H	H; SO_2_CH_3_	=O	Ph	-	-	not active
**11b**	H; H	H; Bz	=O	Ph	-	-	not active
**3b-Ar**	F; H	Aromatic	Aromatic	*m*-F, *p*-CH_3_-Ph	-	-	1.7
**4c-Ar**	H; F	Aromatic	Aromatic	*p*-Cl-Ph	-	1.2	-

a Activity is measured as fold increase in NAD concentration versus the DMSO control; “-” not determined.

Some clear SAR elements emerge from the biological results. Fluorine atoms are the preferred substituents on the aromatic ring of the 1,2,3,4-tetrahydroquinazoline core, regardless of whether they are located at position 6 or 7 (for example, **3c** and **4c**) or at both positions (**1**). Other substitutions lead to reduced activity (**5a–c**), with only marginal activity remaining at 500 nM. These effects mainly reflect steric influences since the same trend occurs whether the substituent is electron donating (**5b**, **5c**) or electron withdrawing (**5a**). Substitutions on the phenyl group (R_3_) are generally well tolerated, with the derivatives remaining consistently very active at 500 nM, regardless of whether the substituent occupies the *ortho*, *meta*, or *para* position and whether it is a small halide, methyl, or methoxy group. Remarkably, activity remains very high even when the substituent is as large as an isopropyl group (**3g**, **4g**). Replacement of the phenyl R_3_ with a cyclohexyl analogue (**3h**) results in a slight decrease in activity, although the compound remains active at 500 nM. Alkylation of the nitrogen atoms was introduced not only for SAR exploration but also to chemically stabilize the compounds against aromatization. Alkylation on the less sterically hindered nitrogen by R_1_ results in reduced activity for all tested compounds (**7a–e**) when compared to the unsubstituted analogues. Nevertheless, the compounds retain fair activity at 500 nM. Double alkylation (R_1_ and R_4_), as well as acetylation or sulfonylation, renders the compounds virtually inactive, even at the highest concentration tested. Compounds **6a–c**, which contain a 3,4-dihydroquinoxalin-2­(1*H*)-one core rather than the 1,2,3,4-tetrahydroquinoxaline core, and the fully aromatized quinoxalines (**3b-Ar**, **4c-Ar**) show no significant activity, even at 5 μM. Finally, no significant difference in potency was observed between the separate enantiomers (*R* **-4a** and *S* **-4a**; *R* **-4b** and *S* **-4b**).

To further elucidate the structure–activity relationship (SAR), a qualitative 3D-QSAR model was generated from molecular field points describing the electrostatic, steric, and hydrophobic potentials of the compounds and their spatial distribution (Figure 2). Differences in these field points across the molecules were analyzed to identify the functional groups and moieties responsible for NAD boosting activity. To enable comparison of the different molecular field points, it was necessary to generate a chemically robust 3D alignment for all molecules in the data set. For this reason, compounds **4g** and *R* **-4b** were selected as reference potent molecules across all tested concentrations, and **7c** was included to broaden the chemical diversity. These three reference structures were used to construct a common 3D ligand pharmacophore template, reconstructing a plausible bioactive conformation and shared pharmacophore by aligning field points (extrema of electrostatic, steric, and hydrophobic fields) together with molecular shape. All active and inactive compounds underwent conformational sampling and were aligned to the selected pharmacophore, with alignments optimized to maximize combined molecular field and shape similarity. From the aligned set, qualitative 3D-SAR models were generated for the three potency readouts (50 nM, 500 nM, and 5 μM), corresponding to Figure 2A, 2B, and 2C, respectively. The resulting maps revealed activity cliff regions, areas where small differences in field or shape correspond to large shifts in potency, highlighting the contributions most critical for biological activity. In detail, a recurrent favorable hydrophobic region was identified adjacent to the *para* substituent of the R_3_ group, together with small unfavorable hydrophobic regions near N1. Electrostatic contributions varied with concentration: at 50 nM (Figure 2A), a weakly negative favored region was observed above N4, together with a faint positive favored region beneath the core (in contact with R_3_); at 500 nM (Figure 2B), a pronounced positive favored region appeared within the phenyl side (R_3_), indicating potency sensitive zones where a combination of lipophilic volume and local positive potential is beneficial; at 5 μM (Figure 2C), the favorable hydrophobic region persisted but was flatter, and electrostatic features were more diffuse, consistent with lower model resolution at higher concentration. Overall, the maps indicate good tolerance for phenyl and cyclohexyl as R_3_ groups, a penalty for additional bulk or polarity around the endocyclic nitrogen atoms and a benefit from electron-withdrawing substitution on the core (consistent with fluorine at either the A or B position). A hydrophobic activity cliff region was also identified at position 4 of the R_3_ substituent, matching the observation that *para* methyl and isopropyl groups enhance potency in this series. The qualitative 3D model further rationalizes the inactivity of carbonyl containing and fully aromatized analogues and the minimal enantio-dependence observed experimentally.

**Figure 2:**
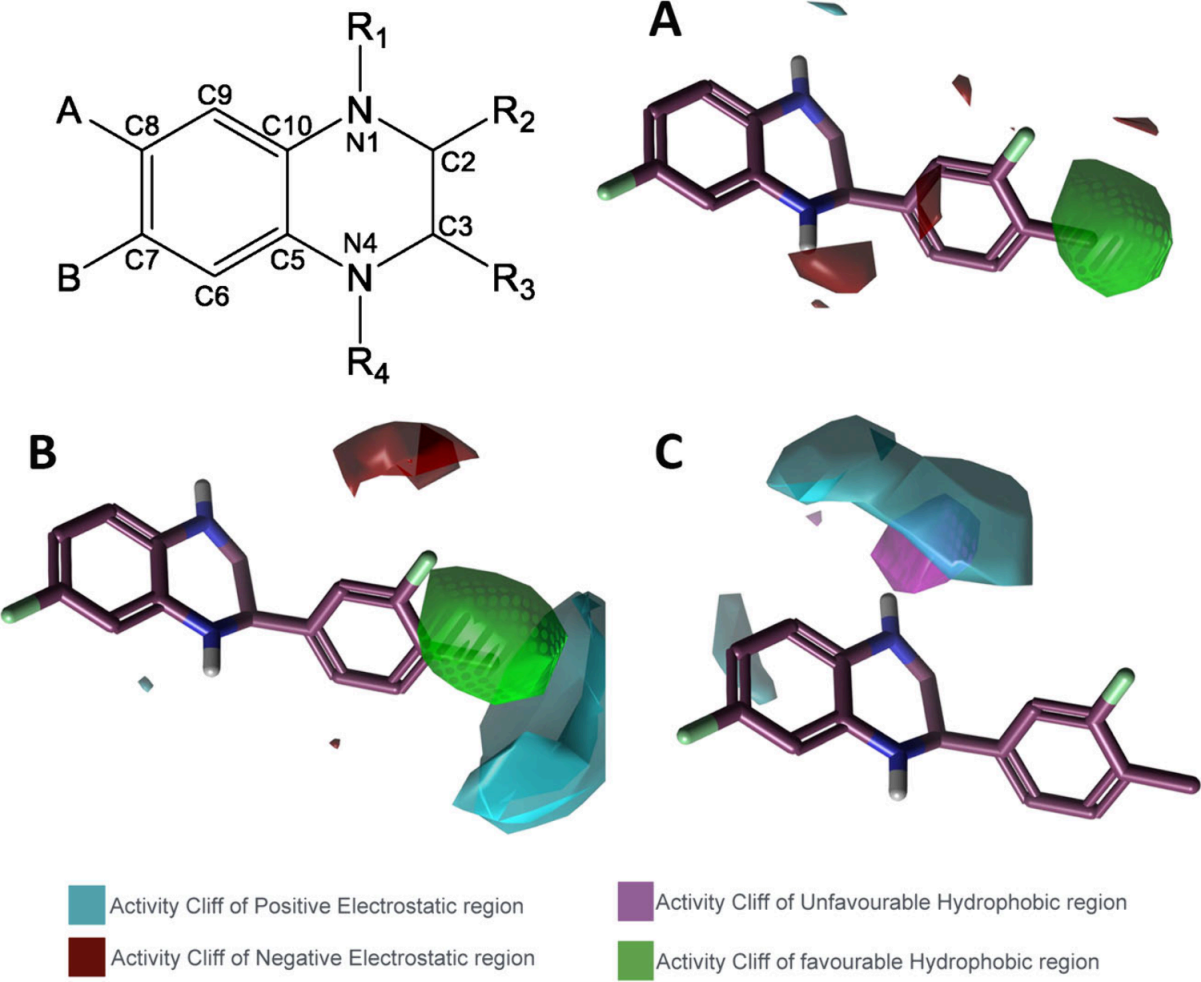
Qualitative 3D-SAR models derived from molecular field points. All models were built on a common pharmacophore template generated from **4g**, *R* **-4b**, and **7c** and depict qualitative hydrophobic and electrostatic activity-cliff regions that highlight where small changes in field or shape correspond to large shifts in potency. (A) 3D-SAR model generated from the NAD-boosting data at 50 nM. (B) 3D-SAR model generated from the NAD-boosting data at 500 nM. (C) 3D-SAR model generated from the NAD-boosting data at 5 μM.

A selection of potent and structurally diverse compounds (**1**, **3e**, **3d**, **3f**, **4c**, *R-* **4b**, *S-* **4b**, and **4f**) was chosen for further *in vitro* DMPK evaluation, including assessments of cell permeability and metabolic stability. All compounds showed a good lipophilic profile (1.9 < log *P* < 2.9) and demonstrated good cell permeability with low evidence of reflux (Figure S159). Liver microsomal stability was evaluated by using mouse liver microsomes (MLMs) and human liver microsomes (HLMs). All compounds showed poor metabolic stability in MLMs, with extraction ratios above 0.8. However, in HLMs, two compounds (**3f** and **4d**) exhibited good stability. In summary, the DMPK results demonstrate that the compounds tested possess favorable cell permeability, poor metabolic stability in MLMs, and improved, although still variable, stability in HLMs. Furthermore, none of the compounds showed satisfactory aqueous solubility.

It is interesting to note that the separated enantiomers *R-* **4b** and *S-* **4b** displayed comparable NAD-boosting activity and similar DMPK profiles, indicating that stereochemistry at this position does not substantially influence biological activity or pharmacokinetic behavior. This suggests that the racemic mixture may represent a viable option for further development, which could simplify the synthesis and formulation. However, additional *in vivo* studies will be required to determine whether stereochemistry affects pharmacological performance under physiological conditions. The observed differences in dissolution kinetics between the enantiomers were unexpected and are likely due to the low aqueous solubility of this compound class and possible variations in amorphous character of the isolated samples (Figure S167).

In summary, we report the design and synthesis of a novel series of 1,2,3,4-tetrahydroquinoxaline derivatives that potently increase NAD levels in cortical neurons at nanomolar concentrations. Structure–activity relationship and qualitative 3D-SAR analyses identified key steric, electrostatic, and hydrophobic features governing activity, while early DMPK studies demonstrated favorable cell permeability but highlighted limitations in aqueous solubility and metabolic stability. These results establish tetrahydroquinoxalines as a promising scaffold for neuronal NAD enhancement. Ongoing efforts focus on improving drug-like properties, particularly solubility and metabolic stability, to enable *in vivo* evaluation. In parallel, we are developing biochemical and biophysical assays to demonstrate direct interaction with NMNAT2, which remains challenging due to the intrinsic instability of the protein outside the cellular environment. We are also continuously refining our computational model of NMNAT2 to improve its predictive accuracy and enable its use in the rational design of next-generation analogues. Together, these approaches will support further optimization and validation of this compound class as potential neuroprotective agents.

## Supplementary Material



## Notes

Experimental section (chemistry; molecular modeling; biological assays), NMR spectra, UPLC traces, and supplemental figures (chrystallography; chiral HPLC separation; additional biological data; *in vitro* DMPK results) (PDF)

## Abbreviation List

AD: Alzheimer’s disease
DCM: Dichloromethane
DMF: Dimethylformamide
DMPK: Drug metabolism and pharmacokinetics
DMSO: Dimethyl sulfoxide
DIPEA: *N*,*N*-Diisopropylethylamine
EGCG: Epigallocatechin gallate
GSH: Glutathione
HLMs: Human liver microsomes
MLMs: Mouse liver microsomes
NAD: Nicotinamide adenine dinucleotide
NAM: Nicotinamide
NAMPT: Nicotinamide phosphoribosyl transferase
NMN: Nicotinamide mononucleotide
NMNAT: Nicotinamide mononucleotide adenylyl transferase
NR: Nicotinamide riboside
PD: Parkinson disease
RLMs: Rat liver microsomes
SAR: Structure activity relationship
SARM1: Sterile alpha and TIR motif containing protein
THF: Tetrahydrofuran
UPLC: Ultra-performance liquid chromatography
