# Supplementary material for: Potent Neuronal Nicotinamide Adenine Dinucleotide-Boosting Tetrahydroquinoxalines: Structure–Activity Relationships and Early Drug Metabolism and Pharmacokinetics Evaluation
Source: ACS Med Chem Lett. 2026 Mar 4;17(4):916–24. doi: 10.1021/acsmedchemlett.6c00058 (PMC13071616; doi:10.1021/acsmedchemlett.6c00058)
Supplement: Supplementary file 1 [file ml6c00058_si_001.pdf]

Potent Neuronal Nicotinamide Adenine Dinucleotide-Boosting Tetrahydroquinoxalines: Structure–Activity Relationships and Early Drug Metabolism and Pharmacokinetics Evaluation

Authors: Petra Cuřínová[1†,] Melissa Jöe[2†], Filip Cesar[1], Alan Nicol[2], Kristián Schwan[1], Michal Kohout[1], Carmine Varrichio[3], Aljona Saleh[4], Craig E Wheelock[7], Gauti Jóhannesson,[2,5,6], Václav Eigner[8], James R Tribble[2], Andrea Brancale[1*], Pete A Williams[2,9*]

Affiliations:
1. Department of Organic Chemistry, University of Chemistry and Technology in Prague, Prague, Czech Republic
2. Department of Clinical Neuroscience, Division of Eye and Vision, St. Erik Eye Hospital, Karolinska Institutet, Stockholm, Sweden.
3. School of Pharmacy and Pharmaceutical Sciences, Cardiff University, Cardiff, UK.
4. Drug discovery and development platform, Science for Life Laboratory, Uppsala, Sweden
5. Department of Clinical Sciences, Ophthalmology, Umeå University, Umeå, Sweden
6. Department of Ophthalmology, University of Iceland, Iceland
7. Unit of Integrative Metabolomics, Institute of Environmental Medicine, Karolinska Institute, Stockholm, Sweden
8. Institute of Physics AS CR, v.v.i., Prague, Czech Republic
9. Centre for Eye Research Australia, Royal Victorian Eye and Ear Hospital, Melbourne, Australia

† Authors contributed equally
* Authors contributed equally
* To whom correspondence should be addressed:
Pete A Williams; Department of Clinical Neuroscience, Division of Eye and Vision, St. Erik Eye Hospital, Karolinska Institutet, Stockholm, Sweden; pete.williams@ki.se.
Andrea Brancale; Department of Organic Chemistry, University of Chemistry and Technology in Prague, Prague, Czech Republic; andrea.brancale@vscht.cz.

Table of Contents

1. Experimental Part ... 3
1.1 Chemistry – General ... 3
1.2 Chemistry – Synthesis ... 3
1.3 Chemical stability ... 19
1.4 Animal strain and husbandry ... 19
1.5 Luminescence-based NAD quantification ... 19
1.6 3D-Structure activity analysis (3D-SAR) ... 19
1.7 Caco-2 permeability ... 20
1.8 Metabolic stability in human, mouse and rat microsomes ... 20
1.9 LC-MS/MS ... 20
1.10 Analysis and statistics ... 21
2. Spectral characterization ... 22
3. Crystalographic data ... 98
4. HPLC/UPLC data ... 100
5. Cell assays ... 108
6. DMPK results ... 109
7. References ... 110

1. Experimental Part

1.1 Chemistry – General

Reagents were purchased from commercial sources and used without further purification. Anhydrous solvents were dried by standard procedures. Flash chromatography was performed on Pure chromatography C-805 instrument, (Buchi, Switzerland), with Pure-flash silica columns using gradient of ethyl acetate in cyclohexane. UPLC/MS was performed on Acquity UPLC H-Class Plus system (Waters) equipped with XBridge Premier BEH C18, 3.5 µm, 4.6 × 100 mm column in the gradient of 10-90 % of ACN in water (1% HCOOH) in 7 min runs at 1 mL/min flow rate. The products were detected by UV (205 and 254 nm) and MS (single quad, ESI pos or neg in 50-1200 m/z mass range). The ^{1}H (400 MHz), ^{13}C (101 MHz), ^{19}F NMR (376 MHz) spectra were recorded using a Jeol 400 spectrometer at 25 °C. Used solvents (DMSO-*d*$_6$, chloroform-*d*) were stored over molecular sieves. The ^{1}H and ^{13}C NMR spectra were referenced to the line of the solvent (δ/ppm; δH/δC: DMSO-*d*$_6$ 2.50/39.52, chloroform-*d*, 7.26/77.16). To assign all proton and carbon signals, a combination of 1D and 2D experiments (H,C-HSQC and H,C-HMBC) was used. The high-resolution mass spectra (HRMS) were measured on a MicrOtof III spectrometer (Bruker) with electrospray or atmospheric pressure chemical ionisation (ESI or APCI) source in positive mode. For calibration of accurate masses, ESI-APCI Low Concentration Tuning Mix (Agilent) was used. The isotope profiles were calculated using freely available software EnviPat Web2.4 [1]. Enantioseparation was performed on a Büchi Pure C-815 with an interface for flash chromatography and preparative high-performance liquid chromatography. The system was further equipped with a PDA detector and an evaporative light scattering detector. The chiral separation was performed in HPLC mode using the full PDA signal (210-400 nm) and specific 254 nm wavelength to collect the separated enantiomers**.** X-ray diffraction data were collected at 180 (2) K on a Bruker D8 Venture Photon CMOS diffractometer with Incoatec microfocus sealed X-ray tube (Cu-*Kα* radiation). The structure was solved by charge flipping methods [2] and anisotropically refined by full matrix least squares on F squared using the CRYSTALS [3]. The hydrogen atoms bonded to carbon atoms were placed in calculated positions and refined with a riding constrains, while hydrogen atoms bonded to nitrogen we refined with restrained geometry. MCE [4] was used for visualization of electron density maps**.** Optical rotation was measured using Jasco P-2000 polarimeter (Jasco, Japan) at 589 nm in a 10 cm cell, concentration is given in g/100 mL.

1.2 Chemistry – Synthesis

1,2,3,4-Tetrahydroquinoxalines- general procedure I

Corresponding 1,2-phenylene diamine (4 mmol) was dissolved in toluene (100 mL), acetic acid (0.5 mL) and ethyl ester of phenyl-2-oxoacetate derivative (4 mmol) were added. The reaction mixture was stirred at 100°C overnight. After cooling, the reaction mixture was evaporated at low pressure to dry and the residue was dried at 2 Torr for 3 h. The residue was then dissolved in anhydrous THF (100 mL) under argon atmosphere. The solution was cooled on an ice bath and lithium aluminium hydride (20 mmol) was added in portions. The cooling bath was removed and the reaction mixture was stirred at 60°C overnight. After cooling, water (100 mL) was carefully added and the product was extracted into ethyl acetate. The organic layers were dried by magnesium sulfate and after filtration evaporated to dryness under reduced pressure. The residue was chromatographed using flash chromatography in the gradient of ethyl acetate in cyclohexane.

6,7-Difluoro-2-phenyl-1,2,3,4-tetrahydroquinoxaline 1

The title compound was obtained from 4,5-difluoro-1,2-phenylene diamine and ethyl phenyl-2-oxoacetate according to the general procedure I as yellow low-melting solid in 75% yield.

^{1}H NMR (400 MHz, Chloroform-*d*) δ 7.44 - 7.30 (m, 5H), 6.37 (dd, *J* = 6.4, 1.1 Hz, 1H), 6.34 (dd, *J* = 6.4, 1.1 Hz, 1H), 4.41 (dd, *J* = 8.3, 3.3 Hz, 1H), 3.02 (bs, 2H), 3.43 (dd, *J* = 11.4, 3.4 Hz, 1H), 3.27 (dd, *J* = 11.4, 8.1 Hz, 1H).

^{13}C NMR (101 MHz, Chloroform-*d*) δ 143.3 (dd, *J* = 237.7, 9.0 Hz), 143.2 (dd, *J* = 237.7, 9.0 Hz), 141.3, 129.9 (dd, *J* = 7.6, 2.9 Hz), 128.9, 128.5 (dd, *J* = 7.6, 2.8 Hz), 128.1, 127.9, 103.2 (dd, *J* = 20.5, 1.4 Hz), 102.8 (dd, *J* = 20.5, 1.4 Hz), 54.5, 48.8.

^{19}F NMR (376 MHz, Chloroform-*d*) δ -150.9, -151.0

HRMS (ESI+) calc for $[C_{14}H_{12}F_2N_2+H]^+$: 247.1041 found 247.1045 $[M+H]^+$.

2-Phenyl-1,2,3,4-tetrahydroquinoxaline 2a

The title compound was obtained from 1,2-phenylene diamine and ethyl phenyl-2-oxoacetate according to the general procedure I as yellow low-melting solid in 72% yield.

^{1}H NMR (400 MHz, Chloroform-*d*) δ 7.42 - 7.33 (m, 5H), 6.68 - 6.66 (m, 2H), 6.60 - 6.57 (m, 2H), 4.48 (dd, *J* = 8.2, 2.9 Hz, 1H), 3.43 (dd, *J* = 11.4, 3.0 Hz, 1H), 3.33 (dd, *J* = 11.4, 8.2 Hz, 1H).

^{13}C NMR (101 MHz, Chloroform-*d*) δ 142.0, 134.3, 133.0, 128.8, 128.0, 127.2, 119.0, 118.9, 114.8, 114.6, 54.8, 49.3.

HRMS (ESI+) calc for $[C_{14}H_{14}N_2+H]^+$: 211.1229 found 211.1224 $[M+H]^+$.

2-(4-Chlorophenyl)-1,2,3,4-tetrahydroquinoxaline 2b

The title compound was obtained from 1,2-phenylene diamine and ethyl (4-chlorophenyl)-2-oxoacetate according to the general procedure I as yellow solid in 65% yield.

^{1}H NMR (400 MHz, Chloroform-d) *δ* 7.34 - 7.29 (m, 4H), 6.65 - 6.62 (m, 2H), 6.58 - 6.55 (m, 2H), 4.45 (dd, *J* = 7.8, 2.7 Hz, 1H), 3.86 (bs, 1H), 3.79 (bs, 1H), 3.41 (dd, *J* = 10.2, 3.1 Hz, 1H), 3.24 (dd, *J* = 10.2, 6.9 Hz, 1H).

^{13}C NMR (101 MHz, Chloroform-*d*) δ 140.0, 133.8, 133.6, 132.8, 128.9, 128.4, 119.1, 119.0, 114.8, 114.6, 54.2, 49.1.

HRMS (ESI+) calc for $[C_{14}H_{13}ClN_2+H]^+$: 245.0840 found 245.0843 $[M+H]^+$.

6-Fluoro-2-phenyl-1,2,3,4-tetrahydroquinoxaline 3a, 7-fluoro-2-phenyl-1,2,3,4-tetrahydroquinoxaline 4a

The title compounds were obtained from 4-fluoro-1,2-phenylene diamine and ethyl 2-phenyl-2-oxoacetate according to the general procedure I. The isomers were separated by flash chromatography at isocratic flow of 20% ethyl acetate in cyclohexane. Fast eluting 3a was obtained as white crystalline solid in 36% yield. Slow eluting 4a was obtained as yellow crystals

in 32% yield. The enantiomers of 4a were separated on chiral HPLC Lux-amylose 3 in an isocratic flow of 35/65 *iso*-propyl alcohol in hexane with addition of 0.05% of diethyl amine. The enantiomers were crystalized from ethyl acetate solutions and characterised by X-ray diffraction as *R*-fast eluting enantiomer, *S*-slow eluting enantiomer.

3a

^{1}H NMR (400 MHz, Chloroform-*d*) δ 7.38 - 7.34 (m, 4H), 7.34 - 7.29 (m, 1H), 6.48 – 6.44 (m, 1H), 6.32 – 6.27 (m, 2H), 4.38 (dd, *J* = 8.1, 3.1 Hz, 1H), 3.91 (bs, 1H), 3.78 (bs, 1H), 3.43 (d, *J* = 11.5 Hz, 1H), 3.31 (dd, *J* = 11.1, 8.5 Hz, 1H).

^{13}C NMR (101 MHz, Chloroform-*d*) δ 157.0 (d, *J* = 234.5 Hz), 141.6, 134.0 (d, *J* = 8.3 Hz), 129.9, 128.8, 128.1, 127.1, 114.7 (d, *J* = 9.1 Hz), 104.2 (d, *J* = 22.3 Hz), 101.3 (d, *J* = 26.0 Hz), 54.6, 49.1.

^{19}F (376 MHz, Chloroform-*d*) δ -125.3

HRMS (ESI+) calc for $[C_{14}H_9FN_2+H]^+$: 229.1135 found 229.1130 $[M+H]^+$.

4a

^{1}H NMR (400 MHz, Chloroform-*d*) δ 7.41-7.29 (m, 5H), 6.55 – 6.46 (m, 1H), 6.35 – 6.27 (m, 2H), 4.48 (dd, *J* = 8.0, 3.1 Hz, 1H), 3.43 (dd, *J* = 11.2, 3.1 Hz, 1H), 3.25 (dd, *J* = 11.2, 8.0 Hz, 1H).

^{13}C NMR (101 MHz, Chloroform-*d*) δ 157.2 (d, *J* = 234.9 Hz), 141.5, 135.4 (d, *J* = 10.3 Hz), 128.8, 128.3 (d, *J* = 2.2 Hz), 128.2, 127.0, 115.6 (d, *J* = 9.3 Hz), 104.3 (d, *J* = 22.3 Hz), 101.3 (d, *J* = 26.0 Hz), 54.8, 49.1.

^{19}F (376 MHz, Chloroform-*d*) δ -124.7

HRMS (APCI+) calc for $[C_{14}H_9FN_2+H]^+$: 229.1135 found 229.1144 $[M+H]^+$.

R-4a $[\alpha]_D^{20}$ +4.1 +- 1.1 (*c* 0.15, $CHCl_3$).

X-Ray: *M* = 228.27 g.mol^{-1}, orthorhombic system, space group $P2_12_12_1$, *a* = 6.00666 (5) Å, *b* = 12.59608 (11) Å, *c* = 14.83212 (12) Å, *Z* = 4, *V* = 1122.20 (2) Å^3, D_c = 1.351 g.cm^{-3}, *μ*(Cu-Kα) = 0.75 mm^{-1}, crystal dimensions of 0.41 × 0.27 × 0.18 mm. The structure was deposited into Cambridge Structural Database under number CCDC 2505539.

S-4b $[\alpha]_D^{20}$ -4.3 +- 1.3 (*c* 0.15, $CHCl_3$).

X-Ray: *M* = 228.27 g.mol^{-1}, orthorhombic system, space group $P2_12_12_1$, *a* = 6.0144 (2) Å, *b* = 12.6163 (3) Å, *c* = 14.9289 (4) Å, *Z* = 4, *V* = 1132.80 (6) Å^3, D_c = 1.338 g.cm^{-3}, *μ*(Cu-Kα) = 0.75 mm^{-1}, crystal dimensions of 0.46 × 0.34 × 0.28 mm. The structure was deposited into Cambridge Structural Database under number CCDC 2505538.

6-Fluoro-2-(3-fluoro-4-methylphenyl)-1,2,3,4-tetrahydroquinoxaline 3b, 7-fluoro-2-(3-fluoro-4-methylphenyl)-1,2,3,4-tetrahydroquinoxaline 4b

The title compounds were obtained from 4-fluoro-1,2-phenylene diamine and ethyl 2-(3-fluoro-4-methylphenyl)-2-oxoacetate according to the general procedure I. The isomers were separated by flash chromatography at isocratic flow of 10% ethyl acetate in cyclohexane. Fast eluting **3b** was obtained as off-white solid in 32% yield. Slow eluting **4b** was obtained as yellow crystals in 25% yield. The enantiomers of **4b** were separated on chiral HPLC Lux-amylose 3

in an isocratic flow of 35/65 *iso*-propyl alcohol in hexane with addition of 0.05% of diethyl amine.

3b

^{1}H NMR (400 MHz, Chloroform-*d*) δ 7.17 (t, *J* = 7.7, 1H), 7.07-7.04 (m, 2H), 6.54- 6.50 (m, 1H), 6.36 – 6.30 (m, 2H), 4.38 (dd, *J* = 8.3, 3.0 Hz, 1H), 3.44 (dd, *J* = 11.2, 3.1 Hz, 1H), 3.31 (dd, *J* = 11.2, 8.3 Hz, 1H), 2.26 (d, *J* = 1.2 Hz, 3H).

^{13}C NMR (101 MHz, Chloroform-*d*) δ 161.5 (d, *J* = 245.1 Hz), 157.1 (d, *J* = 240.0 Hz), 141.4 (d, *J* = 6.5 Hz), 133.8 (d, *J* = 10.8 Hz), 131.7 (d, *J* = 6.5 Hz), 129.7, 124.5 (d, *J* = 17.0 Hz), 122.3 (d, *J* = 3.4 Hz), 114.9 (d, *J* = 9.3 Hz), 113.5 (d, *J* = 23.1 Hz), 104.3 (*J* = 23.1 Hz), 101.3 (d, *J* = 27.4 Hz), 53.9, 48.9, 13.5 (d, *J* = 3.6 Hz).

^{19}F (376 MHz, Chloroform-*d*) δ -116.8, -125.2

HRMS (APCI+) calc for $[C_{15}H_{14}N_2F_2+H]^+$: 261.1198 found 261.1203 $[M+H]^+$

4b

^{1}H NMR (400 MHz, Chloroform-*d*) δ 7.17 (t, *J* = 7.9, 1H), 7.05-7.97 (m, 2H), 6.58- 6.45 (m, 1H), 6.37 – 6.27 (m, 2H), 4.44 (dd, *J* = 7.8, 3.0 Hz, 1H), 3.97 (bs, 2H), 3.41 (dd, *J* = 11.2, 3.1 Hz, 1H), 3.22 (dd, *J* = 11.2, 7.7 Hz, 1H), 2.27 (d, *J* = 1.2 Hz, 3H).

^{13}C NMR (101 MHz, Chloroform-*d*) δ 161.6 (d, *J* = 245.2 Hz), 157.2 (d, *J* = 235.0 Hz), 141.5 (*J* = 7.0 Hz), 135.1 (*J* = 10.2 Hz), 131.8 (d, *J* = 5.3 Hz), 128.5 (d, *J* = 1.9 Hz), 124.5 (d, *J* = 17.3 Hz), 122.2 (d, *J* = 3.1 Hz), 115.5 (d, *J* = 9.1 Hz), 113.4 (d, *J* = 22.7 Hz), 104.4 (*J* = 22.6 Hz), 101.2 (d, *J* = 26.1 Hz), 54.2, 49.0, 14.4 (d, *J* = 3.6 Hz).

^{19}F (376 MHz, Chloroform-*d*) δ -116.5, -124.7.

HRMS (APCI+) calc for $[C_{15}H_{14}N_2F_2+H]^+$: 261.1198 found 261.1198 $[M+H]^+$

R-4b $[\alpha]_D^{20}$ +5.0+-1.2 (*c* 0.15, $CHCl_3$).

S-4b $[\alpha]_D^{20}$ -4.9+-1.4 (*c* 0.15, $CHCl_3$).

2-(4-Chlorophenyl)-6-fluoro-1,2,3,4-tetrahydroquinoxaline 3c, 2-(4-chlorophenyl)-7-fluoro-1,2,3,4-tetrahydroquinoxaline 4c

The title compounds were obtained from 4-fluoro-1,2-phenylene diamine and ethyl 2-(4-chlorophenyl)-2-oxoacetate according to the general procedure I. The isomers were separated by flash chromatography at isocratic flow of 10% ethyl acetate in cyclohexane. Fast eluting **3c** was obtained as off-white low-melting solid in 40% yield. Slow eluting **4c** was obtained as yellow low melting solid in 32% yield.

3c

^{1}H NMR (400 MHz, Chloroform-*d*) δ 7.36 - 7.22 (m, 4H), 6.49 – 6.45 (m, 1H), 6.33 – 6.28 (m, 2H), 4.45 (dd, *J* = 7.7, 3.1 Hz, 1H), 3.97 (bs, 1H), 3.66 (bs, 1H), 3.38 (dd, *J* = 11.2, 3.2 Hz, 1H), 3.18 (dd, *J* = 11.2, 7.7 Hz, 1H).

^{13}C NMR (101 MHz, Chloroform-*d*) δ 156.9 (d, *J* = 234.6 Hz), 140.1, 133.9 (d, *J* = 10.1 Hz), 133.8, 129.6 (d, *J* = 1.8 Hz), 128.9, 128.4, 114.9 (d, *J* = 9.2 Hz), 104.3 (d, *J* = 22.6 Hz), 101.2 (d, *J* = 26.0 Hz), 53.9, 48.9.

^{19}F NMR (376 MHz, Chloroform-*d*) δ -125.0

HRMS (ESI+) calc for $[C_{14}H_{12}FClN_2+H]^+$: calc 263.0746 found 263.0749 $[M+H]^+$.

4c

^{1}H NMR (400 MHz, Chloroform-*d*) δ 7.36 - 7.22 (m, 4H), 6.49 – 6.45 (m, 1H), 6.33 – 6.28 (m, 2H), 4.45 (dd, *J* = 7.7, 3.1 Hz, 1H), 3.97 (bs, 1H), 3.66 (bs, 1H), 3.38 (dd, *J* = 11.2, 3.2 Hz, 1H), 3.18 (dd, *J* = 11.2, 7.7 Hz, 1H).

^{13}C NMR (101 MHz, Chloroform-*d*) δ 157.0 (d, *J* = 235.0 Hz), 140.2, 135.0 (d, *J* = 11.5 Hz), 133.8, 128.9, 128.5, 128.3, 115.4 (d, *J* = 9.2 Hz), 104.4 (d, *J* = 22.6 Hz), 101.2 (d, *J* = 26.0 Hz), 54.2, 48.9.

^{19}F NMR (376 MHz, Chloroform-*d*) δ -124.8.

HRMS (APCI+) calc for $[C_{14}H_{12}FClN_2+H]^+$: calc 263.0746 found 263.0746 $[M+H]^+$.

2-(4-Fluorophenyl)-6-fluoro-1,2,3,4-tetrahydroquinoxaline 3d, 2-(4-fluorophenyl)-7-fluoro-1,2,3,4-tetrahydroquinoxaline 4d

The title compounds were obtained from 4-fluoro-1,2-phenylene diamine and ethyl 2-(4-fluorophenyl)-2-oxoacetate according to the general procedure I. The isomers were separated by flash chromatography at isocratic flow of 15% ethyl acetate in cyclohexane. Fast eluting **3d** was obtained as yellow low-melting solid in 42% yield. Slow eluting **4d** was obtained as yellow low-melting solid in 35% yield.

3d

^{1}H NMR (400 MHz, Chloroform-*d*) δ 7.33 (dd, *J* = 8.7, 2.3 Hz, 2H), 7.04 (t, *J* = 8.1 Hz, 2H), 6.45 (dd, *J* = 8.1, 3.5 Hz, 1H), 6.32 - 6.26 (m, 2H), 4.37 (dd, *J* = 8.2, 3.4 Hz, 1H), 3.82 (bs, 1H), 3.40 (dd, *J* = 11.3, 2.7 Hz, 1H), 3.26 (dd, *J* = 11.6, 8.0 Hz, 1H).

^{13}C NMR (101 MHz, Chloroform-*d*) δ 162.6 (d, *J* = 246.3 Hz), 157.0 (d, *J* = 234.2 Hz), 137.3 (d, *J* = 4.6 Hz), 133.9 (d, *J* = 11.2 Hz), 128.7 (d, *J* = 8.2 Hz), 128.6 (d, *J* = 8.0 Hz), 115.7 (d, *J* = 21.4 Hz), 114.8 (d, *J* = 9.3 Hz), 104.3 (d, *J* = 23.6 Hz), 101.4 (d, *J* = 26.6 Hz), 53.9, 49.1.

^{19}F NMR (376 MHz, Chloroform-*d*) δ -114.3, -125.1

HRMS (ESI+) calc for $[C_{14}H_{12}N_2F_2+H]^+$: 247.1041 found 247.1044 $[M+H]^+$.

4d

^{1}H NMR (400 MHz, Chloroform-*d*) δ 7.32 (dd, *J* = 8.6, 5.4 Hz, 2H), 7.04 (t, *J* = 8.6 Hz, 2H), 6.47 (dd, *J* = 7.6, 2.9 Hz, 1H), 6.34 - 6.28 (m, 2H), 4.45 (dd, *J* = 8.2, 3.4 Hz, 1H), 3.96 (bs, 1H), 3.39 (dd, *J* = 10.9, 2.7 Hz, 1H), 3.22 (dd, *J* = 11.4, 8.0 Hz, 1H).

^{13}C NMR (101 MHz, Chloroform-*d*) δ 162.5 (d, *J* = 246.3 Hz), 157.0 (d, *J* = 234.2 Hz), 137.4 (d, *J* = 4.6 Hz), 135.0 (d, *J* = 11.2 Hz), 128.6, 128.5 (d, *J* = 8.2 Hz), 115.6 (d, *J* = 21.4 Hz), 115.2 (d, *J* = 9.3 Hz), 104.3 (d, *J* = 23.6 Hz), 101.2 (d, *J* = 26.6 Hz), 54.2, 49.1.

^{19}F NMR (376 MHz, Chloroform-*d*) δ -114.3, -124.9

HRMS (ESI+) calc for $[C_{14}H_{12}N_2F_2+H]^+$: 247.1041 found 247.1036 $[M+H]^+$.

6-Fluoro-2-(4-methoxyphenyl)-1,2,3,4-tetrahydroquinoxaline 3e, 7-fluoro-2-(4-methoxyphenyl)- 1,2,3,4-tetrahydroquinoxaline 4e

The title compounds were obtained from 4-fluoro-1,2-phenylene diamine and ethyl 2-(4-methoxyphenyl)-2-oxoacetate according to the general procedure I. The isomers were separated by flash chromatography at isocratic flow of 10% ethyl acetate in cyclohexane. Fast eluting **3e** was obtained as yellow solid in 35% yield. Slow eluting **4e** was obtained as yellow low-melting solid in 28% yield.

3e

^{1}H NMR (400 MHz, Chloroform-*d*) δ 7.27 (d, *J* = 8.7 Hz, 2H), 6.89 (d, *J* = 8.7 Hz, 2H), 6.53 – 6.46 (m, 1H), 6.34 – 6.26 (m, 2H), 4.44 (dd, *J* = 8.2, 3.1 Hz, 1H), 3.81 (s, 3H), 3.39 (dd, *J* = 11.2, 3.1 Hz, 1H), 3.24 (dd, *J* = 11.2, 8.1 Hz, 1H).

^{13}C NMR (101 MHz, Chloroform-*d*) δ 159.5, 157.2 (d, *J* = 234.7 Hz), 135.4 (d, *J* = 10.2 Hz), 133.6, 128.5, 128.1, 115.5 (d, *J* = 9.3 Hz), 114.2, 104.1 (d, *J* = 22.6 Hz), 101.1 (d, *J* = 26.0 Hz), 55.5, 54.3, 49.3.

^{19}F NMR (376 MHz, Chloroform-*d*) δ -125.0

HRMS (ESI+) calc for $[C_{15}H_{15}N_2OF+H]^+$: 259.1241 found 259.1238 $[M+H]^+$.

4e

H NMR (400 MHz, Chloroform-*d*) δ 7.29 (d, *J* = 8.8 Hz, 2H), 6.90 (d, *J* = 8.7 Hz, 2H), 6.46 – 6.42 (m, 1H), 6.31 – 6.26 (m, 2H), 4.33 (dd, *J* = 8.2, 3.1 Hz, 1H), 3.80 (s, 3H), 3.39 (dd, *J* = 10.2, 3.1 Hz, 1H), 3.28 (dd, *J* = 11.2, 8.2 Hz, 1H).

^{13}C NMR (101 MHz, Chloroform-*d*) δ 159.4, 157.3 (d, *J* = 234.5 Hz), 133.9 (d, *J* = 11.2 Hz), 133.6, 128.2, 127.5, 114.6 (d, *J* = 9.3 Hz), 114.1, 104.0 (d, *J* = 22.6 Hz), 101.1 (d, *J* = 26.0 Hz), 55.4, 53.9, 49.1.

^{19}F NMR (376 MHz, Chloroform-*d*) δ -125.0

HRMS (ESI+) calc for $[C_{15}H_{15}N_2OF+H]^+$: 259.1241 found 259.1235 $[M+H]^+$.

6-Fluoro-2-(2-fluorophenyl)-1,2,3,4-tetrahydroquinoxaline 3f, 7-fluoro-2-(2-fluorophenyl)- 1,2,3,4-tetrahydroquinoxaline 4f

The title compounds were obtained from 4-fluoro-1,2-phenylene diamine and ethyl 2-(2-fluorophenyl)-2-oxoacetate according to the general procedure I. The isomers were separated by flash chromatography at isocratic flow of 20% ethyl acetate in cyclohexane. Fast eluting **3f** was obtained as white semi-solid in 36% yield. Slow eluting **4f** was obtained as yellow low-melting solid in 30% yield.

3f

^{1}H NMR (400 MHz, Chloroform-*d*) δ 7.43 - 7.37 (m, 1H), 7.31 - 7.25 (m, 1H), 7.15 (td, *J* = 7.5, 1.3 Hz, 1H), 7.07 (ddd, *J* = 10.5, 8.2, 1.2 Hz, 1H), 6.50 (ddd, *J* = 7.5, 5.4, 1.0 Hz, 1H), 6.39 - 6.27 (m, 2H), 4.79 (dd, *J* = 6.6, 3.2 Hz, 1H), 3.81 (bs, 2H), 3.52 (dd, *J* = 11.2, 3.2 Hz, 1H), 3.30 (dd, *J* = 11.2, 6.6 Hz, 1H).

^{13}C NMR (101 MHz, Chloroform-*d*) δ 160.2 (d, *J* = 249.8 Hz), 157.0 (d, *J* = 235.1 Hz), 133.9 (d, *J* = 8.6 Hz), 129.6, 129.2 (d, *J* = 8.6 Hz), 128.8 (d, *J* = 10.7 Hz), 128.1 (d, *J* = 5.0 Hz), 124.6 (d, *J* = 5.0 Hz), 115.5 (d, *J* = 21.6 Hz), 114.9 (d, *J* = 11.8 Hz), 104.5 (d, *J* = 20.8 Hz), 101.5 (d, *J* = 26.6 Hz), 47.3 (d, *J* = 3.7 Hz), 47.1.

^{19}F NMR (376 MHz, Chloroform-*d*) δ-119.7, -125.2.

HRMS (APCI+) calc for $[C_{14}H_{12}F_2N_2+H]^+$: 247.1041 found 247.1051 $[M+H]^+$.

4f

^{1}H NMR (400 MHz, Chloroform-*d*) δ 7.41 (td, *J* = 7.5, 1.5 Hz, 1H), 7.31 - 7.23 (m, 1H), 7.13 (td, *J* = 7.5, 1.2 Hz, 1H), 7.05 (ddd, *J* = 10.5, 8.2, 1.2 Hz, 1H), 6.48 (dd, *J* = 8.5, 5.4, Hz, 1H), 6.39 - 6.28 (m, 2H), 4.87 (dd, *J* = 7.4, 3.0 Hz, 1H), 3.97 (bs, 1H), 3.62 (bs, 1H), 3.51 (dd, *J* = 11.2, 3.1 Hz, 1H), 3.25 (dd, *J* = 11.2, 7.3 Hz, 1H).

^{13}C NMR (101 MHz, Chloroform-*d*) δ 160.1 (d, *J* = 245.3 Hz), 157.3 (d, *J* = 235.8 Hz), 135.0 (d, *J* = 9.2 Hz), 129.2 (d, *J* = 8.3 Hz), 129.0 (d, *J* = 12.6 Hz), 128.5 (d, *J* = 1.7 Hz), 128.1 (d, *J* = 4.1 Hz), 124.5 (*d*, *J* = 3.4 Hz), 115.7 (d, *J* = 9.2 Hz), 115.4 (d, *J* = 21.7 Hz), 104.4 (d, *J* = 21.4 Hz), 101.3 (d, *J* = 26.0 Hz), 47.6 (d, *J* = 3.3 Hz), 47.0.

^{19}F NMR (376 MHz, Chloroform-*d*) δ -119.9, 124.7.

HRMS (APCI+) calc for $[C_{14}H_{12}F_2N_2+H]^+$: 247.1041 found 247.1047 $[M+H]^+$.

6-Fluoro-2-(4-*iso*-propylphenyl)-1,2,3,4-tetrahydroquinoxaline 3g, 7-fluoro-2-(4-*iso*-propylphenyl)- 1,2,3,4-tetrahydroquinoxaline 4g

The title compounds were obtained from 4-fluoro-1,2-phenylene diamine and ethyl 2-(4-*iso*-propylphenyl)-2-oxoacetate according to the general procedure I. The isomers were separated by flash chromatography at isocratic flow of 20% ethyl acetate in cyclohexane. Fast eluting **3g** was obtained as off-white solid in 38% yield. Slow eluting **4g** was obtained as yellow solid in 32% yield.

3g

^{1}H NMR (400 MHz, Chloroform-*d*) δ 7.30 (d, *J* = 8.2 Hz, 2H), 7.23 (d, *J* = 8.2 Hz, 2H), 6.51 – 6.42 (m, 1H), 6.32 – 6.27 (m, 2H), 4.36 (dd, *J* = 8.4, 3.1 Hz, 1H), 3.43 (dd, *J* = 11.1, 3.1 Hz, 1H), 3.34 (dd, *J* = 11.1, 8.4 Hz, 1H), 2.92 (hept, *J* = 6.9 Hz, 1H), 1.26 (d, *J* = 7.0 Hz, 6H).

^{13}C NMR (101 MHz, Chloroform-*d*) δ 157.0 (d, *J* = 234.5 Hz), 148.9, 138.8, 134.0 (d, *J* = 10.3 Hz), 130.0 (d, *J* = 2.3 Hz), 127.1, 126.9, 114.8 (d, *J* = 9.2 Hz), 104.4 (d, *J* = 22.6 Hz), 101.5 (d, *J* = 26.0 Hz), 54.4, 49.0, 34.0, 24.1.

^{19}F NMR (376 MHz, Chloroform-*d*) δ -125.2

HRMS (APCI+) calcd for $[C_{17}H_{20}N_2F+H]^+$: 271.1605; found 271.1608 $[M+H]^+$.

4g

^{1}H NMR (400 MHz, Chloroform-*d*) δ 7.27 (d, *J* = 8.5 Hz, 2H), 7.21 (d, *J* = 8.5 Hz, 2H), 6.54 – 6.48 (m, 1H), 6.35 – 6.25 (m, 2H), 4.43 (dd, *J* = 8.1, 3.1 Hz, 1H), 3.97 (bs, 1H), 3.39 (dd, *J* = 11.2, 3.2 Hz, 1H), 3.22 (dd, *J* = 11.2, 8.1 Hz, 1H), 2.88 (hept, *J* = 6.9 Hz, 1H), 1.23 (d, *J* = 7.0 Hz, 6H).

^{13}C NMR (101 MHz, Chloroform-*d*) δ 157.1 (d, *J* = 234.5 Hz), 148.8, 138.9, 135.3 (d, *J* = 11.5 Hz), 128.6 (d, *J* = 2.0 Hz), 126.9, 126.8, 115.4 (d, *J* = 9.2 Hz), 104.0 (d, *J* = 22.6 Hz), 101.3 (d, *J* = 26.0 Hz), 54.6, 49.1, 33.9, 24.1.

^{19}F NMR (376 MHz, Chloroform-*d*) δ -125.0.

HRMS (APCI+) calc for $[C_{17}H_{20}N_2F+H]^+$: 271.1605 found 271.1608 $[M+H]^+$.

2-Cyclohexyl-6-fluoro-1,2,3,4-tetrahydroquinoxaline 3h, 2-cyclohexyl-7-fluoro-1,2,3,4-tetrahydroquinoxaline 4h

The title compounds were obtained from 4-fluoro-1,2-phenylene diamine and ethyl 2-cyclohexyl-2-oxoacetate according to the general procedure I. The isomers were separated by flash chromatography at isocratic flow of 10% ethyl acetate in cyclohexane. Fast eluting **3h** was obtained as off-white solid in 43% yield. Slow eluting **4h** was obtained as yellow solid in 35% yield.

3h

^{1}H NMR (400 MHz, Chloroform-*d*) δ 6.37 (dd, *J* = 8.8, 6.3 Hz 1H), 6.26 - 6.18 (m, 2H), 3.64 (bs, 1H), 3.36 (dd, *J* = 10.8, 2.7 Hz 1H), 3.14 (dd, *J* = 10.4, 7.7 Hz 1H), 3.02 (m, 1H), 1.89 - 1.68 (m, 5H), 1.43 - 1.34 (m, 1H), 1.31 - 0.98 (m, 5H).

^{13}C NMR (101 MHz, Chloroform-*d*) δ 156.7 (d, *J* = 235.1 Hz), 134.6 (d, *J* = 9.1 Hz), 129.5 (d, *J* = 1.6 Hz), 114.7 (d, *J* = 10.4 Hz), 104.0 (d, *J* = 22.8 Hz), 100.8 (d, *J* = 26.8 Hz), 55.0, 44.0, 40.6, 29.2/29.0, 26.5/26.19, 26.23.

^{19}F NMR (376 MHz, Chloroform-*d*) δ -125.8

HRMS (ESI+) calc for $[C_{14}H_{19}FN_2+H]^+$ 235.1605, found 235.1602 $[M+H]^+$.

4h

^{1}H NMR (400 MHz, Chloroform-*d*) δ 6.41 - 6.38 (m, 1H), 6.26 - 6.20 (m, 2H), 3.78 (bs, 1H), 3.34 - 3.29 (m, 1H), 3.14 - 3.08 (m, 2H), 1.87 - 1.68 (m, 5H), 1.45 -1.37 (m, 1H), 1.31 - 0.98 (m, 5H).

^{13}C NMR (101 MHz, Chloroform-*d*) δ 156.9 (d, *J* = 235.1 Hz), 135.1 (d, *J* = 9.1 Hz), 129.2 (d, *J* = 1.6 Hz), 114.9 (d, *J* = 10.4 Hz), 103.6 (d, *J* = 22.8 Hz), 101.0 (d, *J* = 26.8 Hz), 55.4, 43.9, 40.8, 29.3/29.0, 26.5/26.2, 26.3.

^{19}F NMR (376 MHz, Chloroform-*d*) δ -125.4

HRMS (ESI+) calc for $[C_{14}H_{19}FN_2+H]^+$ 235.1605, found 235.1612 $[M+H]^+$.

2-(4-Chlorophenyl)-6-trifluoromethyl-1,2,3,4-tetrahydroquinoxaline 5a

The title compound was obtained cyclizing 4-trifluoromethyl-1,2-phenylene diamine with ethyl 2-(4-chlorophenyl)-2-oxoacetate according to the general procedure I. The reduction step was performed dissolving IM-5a dry solid in THF under argon atmosphere and adding BH_3*THF solution (1M, 5 equiv.) at cooling. After the addition, the reaction mixture was heated to 60°C overnight. After cooling, the solvents were evaporated, the residue was suspended in DCM and washed with water. The organic layer was dried by magnesium sulfate and after filtration evaporated to dryness. The isomer **5a** was isolated after repetitive evolution of the preparative TLC plate in ethyl acetate-cyclohexane 1/6 ratio eluent as a yellow solid in 40% yield.

^{1}H NMR (400 MHz, Chloroform-*d*) δ 7.33 (d, *J* = 8.5 Hz, 2H), 7.27 (d, *J* = 8.5 Hz, 2H), 6.87 (dd, *J* = 8.2, 2.0 Hz, 1H), 6.77 (d, *J* = 2 Hz, 1H), 6.57 (d, *J* = 8.2 Hz, 1H), 4.49 (dd, *J* = 7.8, 3.2 Hz, 1H), 4.16 (bs, 1H), 3.94 (bs, 1H), 3.47 (dd, *J* = 11.2, 3.3 Hz, 1H), 3.25 (dd, *J* = 11.2, 7.7 Hz, 1H).

^{13}C NMR (101 MHz, Chloroform-*d*) δ 139.8, 136.5, 133.9, 132.4, 129.0, 128.3, 124.9 (q, *J* = 272.2 Hz), 120.5 (q, *J* = 32.0 Hz), 116.2 (q, *J* = 4.2 Hz), 113.4, 111.0 (q, *J* = 4.2 Hz), 53.9, 48.4.

^{19}F (376 MHz, Chloroform-*d*) δ -61.1

HRMS (APCI+) calc for $[C_{15}H_{12}N_2ClF_3+H]^+$: 312.0635 found 312.0633 $[M+H]^+$.

2-(4-Chlorophenyl)-6-methyl-1,2,3,4-tetrahydroquinoxaline 5b

The title compound was obtained from 4-trifluoromethyl-1,2-phenylene diamine and ethyl 2-(4-chlorophenyl)-2-oxoacetate according to the general procedure I. The lithium aluminium hydride reduction caused replacement of the fluorine atoms in the trifluoromethyl group by hydrides. The product was isolated by the flash chromatography at isocratic flow of 10% ethyl acetate in cyclohexane. The compound **5b** was obtained as off-white solid in 25% yield.

^{1}H NMR (400 MHz, Chloroform-*d*) δ 7.32 - 7.29 (m, 4H), 6.49 - 6.39 (m, 3H), 4.44 (dd, *J* = 8.1, 3.1 Hz, 1H), 3.39 (dd, *J* = 11.0, 3.1 Hz, 1H), 3.23 (dd, *J* = 11.0, 8.0 Hz, 1H), 2.20 (s, 3H).

^{13}C NMR (101 MHz, Chloroform-*d*) δ 140.7, 134.0, 133.6, 130.3, 128.9, 128.4, 119.5, 115.3, 115.2, 54.4, 49.3, 20.8.

HRMS (APCI+) calc for $[C_{15}H_{15}ClN_2+H]^+$: 259.0996 found 259.0993 $[M+H]^+$.

2-(4-Chlorophenyl)-6-methoxy-1,2,3,4-tetrahydroquinoxaline 5c

The title compound was obtained from 4-methoxy-1,2-phenylene diamine and ethyl 2-(4-chlorophenyl)-2-oxoacetate according to the general procedure I. The isomers were separated by flash chromatography at isocratic flow of 20% ethyl acetate in cyclohexane. The compound **5c** was obtained as off-white solid in 55% yield.

^{1}H NMR (400 MHz, Chloroform-*d*) δ 7.31-7.30 (m, 4H), 6.49 (d, *J* = 8.4 Hz, 1H), 6.25-6.14 (m, 2H), 4.36 (dd, *J* = 8.2, 3.0 Hz, 1H), 3.72 (s, 3H), 3.41 (dd, *J* = 11.1, 3.0 Hz, 1H), 3.27 (dd, *J* = 11.1, 8.3 Hz, 1H).

^{13}C NMR (101 MHz, Chloroform-*d*) δ 153.5, 140.5, 133.7,133.6, 128.8, 128.5, 127.6, 115.3, 103.7, 101.2, 55.7, 54.3, 49.2.

HRMS (ESI+) calc for $[C_{15}H_{15}OClN_2+H]^+$: 275.0945, found 275.0946 $[M+H]^+$.

Dihydroquinoxalin-2(1H)-ones- general procedure II

Respective 1,2-phenylene diamine (4 mmol) was dissolved in toluene (100 mL), acetic acid (0.5 mL) and ethyl ester of phenyl-2-oxoacetate derivative (4 mmol) were added. The reaction mixture was stirred at 100°C overnight. After cooling, the reaction mixture was evaporated at low pressure to dry and the residue was dried at 2 Torr for 3 h. The residue was put into autoclave, dissolved in methanol (50 mL) and palladium on carbon (10%, 0.1 mass equivalents) was added. The autoclave was closed and filled with hydrogen gas to 8 Bar. The reaction mixture was stirred at rt overnight. The methanol solution was filterer and evaporated to dry giving pure products.

3-Phenyl-3,4-dihydroquinoxalin-2(1H)-one 6a

The title compound was obtained from 1,2-phenylene diamine and ethyl 2-phenyl-2-oxoacetate according to the general procedure II in 70% yield over 2 steps.

^{1}H NMR (400 MHz, DMSO-*d*$_6$) δ 10.38 (s, 1H), 7.29 - 7.19 (m, 3H), 6.76 - 6.58 (m, 3H), 6.62 (s, 1H), 6.57 - 6.53 (m, 1H), 4.88 (s, 1H).

^{13}C NMR (101 MHz, DMSO-*d*$_6$) δ 166.4, 140.8, 134.3, 128.8, 128.1, 127.4, 125.8, 123.5, 118.1, 115.3, 113.8, 59.8.

HRMS (ESI+) calc for [$C_{14}H_{12}N_2O$+Na]$^+$: 247.0841 found 247.0840 [M+Na]$^+$.

7-Fluoro-3-(4-methoxyphenyl)-3,4-dihydroquinoxalin-2(1H)-one 6b

The title compound was obtained from 4-fluoro-1,2-phenylene diamine and ethyl 2-(4-methoxyphenyl)-2-oxoacetate according to the general procedure II in 73% yield over 2 steps.

^{1}H NMR (400 MHz, DMSO- *d*$_6$) δ 8.42 (bs, 1H, B), 8.38 (bs, 1H, A), 7.33-7.28 (m, 4H, A+B) 6.89 - 6.84 (m, 4H, A+B), 6.64 - 6.61 (m, 1H, A, +2H, B), 6.49 (d, *J* = 8.9, 1H, B), 6.46 - 6.40 (m, 2H, A), 5.00 - 4.99 (m, 1H, A), 4.95 - 4.93 (m, 1H, B), 4.32 (bs, 1H, A), 4.14 (bs, 1H, B), 3.77 (s, 6H, A+B).

^{13}C NMR (101 MHz, DMSO- *d*$_6$) δ 167.6 (CO, A), 166.7 (CO, B), 160.9 (*C*OCH$_3$, B), 159.9 (*C*OCH$_3$, A), 159.7 (d, *J* = 239.8 Hz, CF, A), 156.7 (d, *J* = 234.6 Hz, CF, B), 134.5 (Cq-NH, A), 130.8 (Cq-CH, A), 130.5 (Cq-CH, B), 129.3 (Cq-NH, B), 128.5 (ArCH, B), 128.4 (Cq-NH, A), 127.9 (Cq-NH, B), 120.9 (Cq-NH, A), 116.0 (d, *J* = 9.2 Hz, *C*H-CH-CF, A), 114.5 (d, *J* = 9.2 Hz, *C*H-CH-CF, B), 114.4 (ArCH, A), 114.3 (ArCH, B), 109.9 (d, *J* = 20.8 Hz, CH-CF, B), 105.6 (d, *J* = 20.8 Hz, CH-CF, A), 102.9 (d, *J* = 26.9 Hz, CH-CF, B), 101.0 (d, *J* = 26.9 Hz, CH-CF, A), 60.3 (CH, B), 59.9 (CH, A), 55.4 (CH3, A+B).

^{19}F -118.5 (A), -124.0 (B).

HRMS (ESI+) calc for [$C_{15}H_{13}FN_2O_2$+Na]$^+$: 295.0853 found 295.0858 [M+Na]$^+$.

***N*-Alkylation of tetrahydroquinoxalines III**

Respective tetrahydroquinoxaline derivative (1 mmol) was dissolved in anhydrous DMF 25 mL. Sodium hydride (60% in paraffin, 1 equiv.) was added and the solution was stirred 5 min at room temperature. Alkylating agent was added and the mixture was heated to 60°C for specified time, observing the reaction progress by TLC. After completion, the reaction was cooled, diluted by ethyl acetate and washed with brine. The organic layer was dried by magnesium sulfate and evaporated to dryness under reduced pressure.

6,7-Difluoro-1-methyl-3-phenyl-2,3,4-trihydroquinoxaline 7a

Starting from compound **1** and methyl iodide, the title compound was prepared according to the general procedure III. The reaction was finished after 5 h and the product was obtained as brownish solid after purification by preparative TLC using ethyl acetate-cyclohexane 1/4 as eluent. The compound was isolated in 15% yield.

^{1}H NMR (400 MHz, Chloroform-*d*) δ 7.36 - 7.30 (m, 5H), 6.39 - 6.31 (m, 2H), 4.54 (dd, *J* = 8.2, 3.4 Hz, 1H), 3.24 (dd, *J* = 11.2, 3.1 Hz, 1H), 3.15 (dd, *J* = 11.2, 8.1 Hz, 1H), 2.81 (s, 3H).

^{13}C NMR (101 MHz, Chloroform-*d*) δ 143.4 (dd, *J* = 233.1, 10.1 Hz), 142.7 (dd, *J* = 234.0, 12.0 Hz), 141.2, 131.8 (d, *J* = 10.7 Hz), 130.5 (d, *J* = 10.7 Hz), 128.8, 128.2, 127.0, 101.9 (d, *J* = 22.2 Hz), 100.6 (d, *J* = 22.6 Hz), 57.2, 54.8, 39.4.

^{19}F NMR (376 MHz, Chloroform-*d*) δ -151.1, -152.4

HRMS (APCI+) calc for $[C_{15}H_{14}F_2N_2+H]^+$: 261.1198 found 261.1192 $[M+H]^+$.

6,7-Difluoro-1-butyl-3-phenyl-2,3,4-trihydroquinoxaline 7b

Starting from compound **1** and butyl bromide, the title compound was prepared according to the general procedure III. The reaction was finished after 3 h, after disappearance of the staring compound. The crude product contained inseparable 7a-Q salt. Therefore, it was dissolved in methanol and stirred with 2 equivalents of sodium borohydride for 1h at the rt. The reaction was quenched with water and the product was extracted to DCM. The organic layers were dried by magnesium sulfate and evaporated, giving **7b** as greenish solid in 20% yield.

^{1}H NMR (400 MHz, Chloroform-*d*) δ 7.37 - 7.30 (m, 5H), 6.38 - 6.32 (m, 2H), 4.38 (dd, *J* = 8.2, 3.4 Hz, 1H), 3.81 (bs, 1H), 3.28 - 3.19 (m, 3H), 3.07 (dd, *J* = 11.2, 8.1 Hz, 1H), 1.59 - 1.49 (m, 2H), 1.37 - 1.27 (m, 2H), 0.92 (t, *J* = 6.9 Hz, 3H).

^{13}C NMR (101 MHz, Chloroform-*d*) δ 143.6 (dd, *J* = 233.1, 13.1 Hz), 141.9 (dd, *J* = 234.0, 13.3 Hz), 141.2, 130.5 (dd, *J* = 8.7, 2.3 Hz), 130.2 (dd, *J* = 8.7, 2.4 Hz), 128.8, 128.2, 127.0, 102.2 (d, *J* = 22.4 Hz), 100.1 (d, *J* = 22.9 Hz), 55.3, 54.3, 51.6, 28.0, 20.5, 14.0.

^{19}F NMR (376 MHz, Chloroform-*d*) δ -150.7, -153.6

HRMS (ESI+) calc for $[C_{18}H_{20}F_2N_2+H]^+$: 303.1667 found 303.1671 $[M+H]^+$.

6,7-Difluoro-3-phenyl-1-(4-trifluoromethylbenzyl)-2,3,4-trihydroquinoxaline 7c

Starting from compound **1** and 4-trifluoromethylbenzyl bromide, the title compound was prepared according to the general procedure III. The reaction was finished after 3 h, and the product was obtained after purification on a preparative TLC plate using ethyl acetate-cyclohexane 1/4 mixture as an eluent. **7c** was isolated as white solid in 63% yield.

^{1}H NMR (400 MHz, Chloroform-*d*) δ 7.56 (d, *J* = 7.9 Hz, 2H), 7.38 - 7.30 (m, 7H), 6.40 (dd, *J* = 10.5, 5.6 Hz, 1H), 6.27 (dd, *J* = 12.5, 8.7 Hz, 1H), 4.50 (dd, *J* = 6.6, 1.7 Hz, 1H), 4.45 (d, *J* = 16.6 Hz, 1H), 4.35 (d, *J* = 16.6 Hz, 1H), 3.96 (bs, 1H), 3.34 (m, 2H).

^{13}C NMR (101 MHz, Chloroform-*d*) δ 143.4 (dd, *J* = 235.7, 14.8 Hz), 142.8 (dd, *J* = 235.7, 15.0 Hz), 142.1, 140.9, 130.3, 130.2, 129.6 (q, *J* = 33.1 Hz), 128.8, 128.3, 127.2, 127.0, 125.8 (q, *J* = 4.3 Hz), 124.4 (q, *J* = 272.9 Hz), 102.6 (d, *J* = 20.4 Hz), 101.1 (dd, *J* = 20.4 Hz), 55.8, 55.6, 54.4.

^{19}F NMR (376 MHz, Chloroform-*d*) δ -62.3, -150.4, -151.8

HRMS (ESI+) calc for $[C_{22}H_{17}F_5N_2+H]^+$: 405.1383.1241 found 405.1384 $[M+H]^+$.

6,7-Difluoro-1-phenethyl-3-phenyl-2,3,4-trihydroquinoxaline 7d

As the synthesis of the title compound **7d** starting from compound **1** and 2-(bromoethyl)benzene according to the general procedure III was unsuccessful, the procedure

was altered. The compound 1 was stirred in methanol with 1 equivalent of 2-phenylacetaldehyde at 0°C for 1 h. After that, zinc chloride, (1.5 equivalent) and sodium cyanoborohydride (1.5 equivalent) were added and the reaction was heated to 50°C overnight. The reaction was quenched by addition of water and the product was extracted into dichloromethane. After drying by magnesium sulfate, the filtrated solution was evaporated giving pure product **7d** as yellow gel in 90% yield.

^{1}H NMR (400 MHz, Chloroform-*d*) δ 7.38 - 7.26 (m, 8H), 7.21 - 7.19 (m, 2H), 6.44 – 6.34 (m, 2H), 4.28 (dd, *J* = 6.4, 2.4 Hz, 1H), 3.81 (bs, 1H), 3.50 - 3.36 (m, 2H), 3.27 – 3.16 (m, 2H), 2.90 - 2.75 (m, 2H).

^{13}C NMR (101 MHz, Chloroform-*d*) δ 144.0 (dd, *J* = 234.9, 14.3 Hz), 141.6 (dd, *J* = 235.0, 14.1 Hz), 140.9, 139.4, 130.2 (dd, *J* = 7.7, 2.4 Hz), 129.9 (dd, *J* = 7.5, 2.3 Hz), 128.9, 128.8, 128.7, 128.2, 127.0, 126.5,102.5 (d, *J* = 22.4 Hz), 99.9 (dd, *J* = 22.0 Hz), 55.7, 54.1, 53.7, 32.2.

^{19}F NMR (376 MHz, Chloroform-*d*) δ -150.3, -153.2

HRMS (ESI+) calc for $[C_{14}H_{13}ClN_2+H]^+$: 351.1668 found 351.1672 $[M+H]^+$.

Double *N*-Alkylation of tetrahydroquinoxalines IV

Respective tetrahydroquinoxaline derivative (1 mmol) was dissolved in anhydrous DMF 25 mL. Sodium hydride (60% in paraffine, 5 equiv.) was added and the solution was stirred 5 min at room temperature. Alkylating agent (5 equiv.) was added and the mixture was heated to 60°C for specified time, observing the reaction progress by TLC. After completion, the reaction was cooled, diluted by ethyl acetate and washed with brine. The organic layer was dried by magnesium sulfate and evaporated to dryness under reduced pressure.

6,7-Difluoro-1,4-dimethyl-2-phenyl-2,3-dihydroquinoxaline 7e

Starting from compound **1** and methyl iodide, the reaction according to general procedure IV gave very low yields (cca 3%) due to extensive aromatization of the starting compound. For the synthesis of 7e, DMF was omitted and the alkylating agent (methyl iodide, 5 mL) was used as solvent for the reaction. The reaction was carried out at the room temperature overnight. The product was isolated by preparative TLC using the mixture methanol/chloroform 1/100 as an eluent. The title compound was obtained as yellow oil in 15% yield.

^{1}H NMR (400 MHz, Chloroform-*d*) δ 7.35 - 7.26 (m, 3H), 7.19 (d, *J* = 8.3 Hz, 2H), 6.38 - 6.31 (m, 2H), 4.40 (dd, *J* = 4.7, 3.8 Hz, 1H), 3.33 (dd, *J* = 11.2, 3.3 Hz, 1H), 3.11 (dd, *J* = 11.2, 5.5 Hz, 1H), 2.73 (s, 3H), 2.70 (s, 3H).

^{13}C NMR (101 MHz, Chloroform-*d*) δ 142.9 (dd, *J* = 233.7, 12.3 Hz), 141.8 (dd, *J* = 233.8, 12.6 Hz), 141.7, 133.4 (d, *J* = 8.7 Hz), 132.8 (d, *J* = 9.2 Hz), 128.6, 127.7, 127.0, 100.0 (d, *J* = 22.7 Hz), 99.0 (d, *J* = 22.9 Hz), 52.7, 56.6, 39.6, 37.5.

^{19}F NMR (376 MHz, Chloroform-*d*) δ -151.4, -153.6

HRMS (ESI+) calc for $[C_{16}H_{16}F_2N_2+Na]^+$: 297.1173, found 297.1174 $[M+Na]^+$.

1,4-Dibenzyl-6-fluoro-2-(3-fluoro-4-methylphenyl)-2,3-dihydroquinoxaline 8a

Starting from compound **3b** and benzylbromide, the title compound was prepared according to general procedure IV. The title compound was purified by preparative TLC using the mixture of ethyl acetate/cyclohexane 1/4 as an eluent. Compound **8a** was obtained as greenish oil in 12% yield.

^{1}H NMR (400 MHz, Chloroform-*d*) δ 7.32 - 7.21 (m, 6H), 7.19 - 7.16 (m, 3H), 7.07 (t, *J* = 8.4 Hz, 1H), 7.01 - 6.98 (m, 2H), 6.85 - 6.82 (m, 2H), 6.42 (dd, *J* = 8.7, 5.5 Hz, 1H), 6.37-6.32 (m, 2H), 4.58 - 4.54 (m, 2H), 4.35 (d, *J* = 15.6 Hz, 1H), 4.21 (dd, *J* = 16.2, 6.0 Hz, 2H), 3.58 (dd, *J* = 11.4, 3.4 Hz, 1H), 3.24 (dd, *J* = 11.4, 3.4 Hz, 1H).

^{13}C NMR (101 MHz, Chloroform-*d*) δ 161.5 (d, *J* = 243.4 Hz), 156.3 (d, *J* = 234.3 Hz), 142.2, 138.3, 137.5, 136.6 (d, *J* = 10.7 Hz), 133.6, 131.3 (d, *J* = 20.2 Hz), 128.7, 128.5, 127.3, 127.1, 127.0, 126.7, 123.8, 122.5, 113.9 (d, *J* = 19.7 Hz), 111.1 (d, *J* = 20.2 Hz), 104.5 (d, *J* = 25.3 Hz), 101.1 (d, *J* = 26.8 Hz), 60.6, 55.4, 53.9, 53.5, 14.2.

^{19}F (376 MHz, Chloroform-*d*) δ -117.3, -126.6.

HRMS (ESI+) calc for $[C_{29}H_{26}F_2N_2+H]^+$: 441.2137 found 441.2131 $[M+H]^+$.

Acylation/sulfonation of tetrahydroquinoxalines V

Respective tetrahydroquinoxaline derivative (1 mmol) was dissolved in dichloromethane 25 mL. DIPEA (1.1 mmol) was added followed by the addition of acylating/sulfonating agent (1.2 mmol). The reaction mixture was stirred at rt overnight. The reaction was quenched by addition of water and the product was extracted to dichloromethane. The organic layers were dried by magnesium sulfate and evaporated to dryness under reduced pressure.

3-(4-Chlorophenyl)-6-fluoro-1-(methylsulfonyl)-2,3,4-trihydroquinoxaline 9a

Starting from compound **4c** and methansulfonyl chloride, the title compound was prepared according to the general procedure V. The product was obtained after purification on a preparative TLC plate using ethyl acetate-cyclohexane 1/2 mixture as an eluent. **9a** was isolated as yellow solid in 62% yield.

^{1}H NMR (400 MHz, Chloroform-*d*) δ 7.47 (dd, J = 8.9, 5.7 Hz, 1H) 7.35 (d, *J* = 8.3 Hz, 2H), 7.29 (d, *J* = 8.3 Hz, 2H), 6.48 - 6.37 (m, 2H), 4.51 (dd, *J* = 9.7, 3.8 Hz, 1H), 4.40 (bs, 1H), 4.22 (ddd, *J* = 14.0, 3.8, 1.5 Hz, 1H), 3.20 (dd, *J* = 14.0, 9.7 Hz, 1H), 2.84 (s, 3H).

^{13}C NMR (101 MHz, Chloroform-*d*) δ 161.4 (d, *J* = 235.8 Hz), 138.5 (d, *J* = 10.1 Hz), 137.6, 134.7, 129.3, 128.2, 126.0 (d, *J* = 9.7 Hz), 117.8, 105.0 (d, *J* = 23.1 Hz), 101.3 (d, *J* = 26.4 Hz), 53.6, 50.3, 39.4.

^{19}F NMR (376 MHz, Chloroform-*d*) δ -115.3

HRMS (ESI+) calc for $[C_{15}H_{14}ClFN_2O_2S+H]^+$: 341.521 found 341.518 $[M+H]^+$.

1-(3-(4-Chlorophenyl)-7-fluoro-3,4-dihydroquinoxalin-1(2H)-yl)ethan-1-one 9b

Starting from compound **4c** and acetyl chloride, the title compound was prepared according to the general procedure V. The product was obtained after purification on a preparative TLC plate using ethyl acetate-cyclohexane 1/4 mixture as an eluent. **9b** was isolated as white semi-solid in 45% yield.

^{1}H NMR (400 MHz, Chloroform-*d*) δ 7.34 – 7.19 (m, 2H), 7.27 - 7.18 (m, 2H), 6.98 -6.83 (m, 1H), 6.43 – 6.34 (m, 2H), 4.59 – 4.49 (m, 1H), 4.36 (d, *J* = 12.4 Hz, 1H), 3.31 (dd, *J* = 12.7, 7.9 Hz, 1H), 2.17 (s, 3H).

^{13}C NMR (101 MHz, Chloroform-*d*) δ 169.7, 161.4 (d, *J* = 243.3 Hz), 139.0, 138.5, 134.1, 129.0, 128.0, 125.6 (d, *J* = 10.4 Hz), 120.8, 103.2 (d, *J* = 23.1 Hz), 100.6 (d, *J* = 26.2 Hz), 55.6, 45.5, 22.3.

^{19}F NMR (376 MHz, Chloroform-*d*) δ -115.6.

HRMS (APCI+) calc for $[C_{16}H_{14}OClFN_2]^+$: 305.0851, found 305.0849 $[M+H]^+$.

2-Amino-1-(3-(4-chlorophenyl)-7-fluoro-3,4-dihydroquinoxalin-1(2H)-yl)ethan-1-one 9c

Starting from compound **4c** and 2-iodoacetamide, the title compound was prepared according to the general procedure V. The product was obtained after purification on a preparative TLC plate using ethyl acetate-cyclohexane 1/2 mixture as an eluent. **9c** was isolated as yellow solid in 60% yield.

^{1}H NMR (400 MHz, Chloroform-*d*) δ 7.33 (d, *J* = 8.7 Hz, 2H), 7.27 (d, *J* = 8.7 Hz, 2H), 6.36 (m, 3H), 6.08 (bs, 1H), 5.81 (bs, 1H), 4.58 (dd, *J* = 7.8, 3.0 Hz, 1H), 4.22 (bs, 1H), 3.72 (d, *J* = 18.7 Hz, 1H), 3.58 (d, *J* = 18.1 Hz, 1H), 3.34 (dd, *J* = 11.2, 3.0 Hz, 1H), 3.22 (dd, *J* = 11.2, 7.1 Hz, 1H).

^{13}C NMR (101 MHz, Chloroform-*d*) δ 173.1, 157.6 (d, *J* = 237.9 Hz), 139.8, 135.8 (d, *J* = 10.0 Hz), 134.0, 129.4 (d, *J* = 2.1 Hz), 128.2, 128.0, 113.9 (d, *J* = 8.3 Hz), 104.5 (d, *J* = 23.7 Hz), 101.1 (d, *J* = 27.8 Hz), 57.5, 56.8, 56.9.

^{19}F NMR (376 MHz, Chloroform-*d*) δ -123.4.

HRMS (APCI+) calc for $[C_{16}H_{15}ClFON_3+H]^+$: 320.0960, found 320.0957 $[M+H]^+$.

Ethyl 2-(3-(4-chlorophenyl)-6-fluoro-2,3,4-trihydroquinoxalin-1-yl)acetate 9d

Starting from compound **4c** and ethyl 2-bromoacetate, the title compound was prepared according to the general procedure V. The product was obtained after purification on a preparative TLC plate using ethyl acetate-cyclohexane 1/4 mixture as an eluent. **9d** was isolated as off-white solid in 55% yield.

^{1}H NMR (400 MHz, Chloroform-*d*) δ 7.37 - 7.28 (m, 4H), 6.39 - 6.26 (m, 3H), 4.55 (dd, *J* = 8.0, 3.3 Hz, 1H), 4.17 (q, *J* = 7.1 Hz, 2H), 4.02 (d, *J* = 17.8 Hz, 1H), 3.86 (d, *J* = 17.8 Hz, 1H), 3.47 - 3.39 (m, 1H), 3.33 (dd, *J* = 11.2, 3.4 Hz, 1H), 1.24 (t, *J* = 7.1 Hz, 3H).

^{13}C NMR (101 MHz, Chloroform-*d*) δ 170.7, 156.7 (d, *J* = 235.9 Hz), 139.7, 135.2 (d, *J* = 10.6 Hz), 133.8, 129.9 (d, *J* = 1.6 Hz), 128.9, 128.4, 111.4 (d, *J* = 11.2 Hz), 104.2 (d, *J* = 22.3 Hz), 101.0 (d, *J* = 26.4 Hz), 61.1, 56.1, 54.0, 53.4, 14.3

^{19}F NMR (376 MHz, Chloroform-*d*) δ -125.9.

HRMS (APCI+) calc for $[C_{18}H_{18}N_2O_2ClF+H]^+$ calc 349.1113, found 349.118 $[M+H]^+$.

3-(4-Chlorophenyl)-6-fluoro-1-(prop-2-yn-1-yl)-2,3,4-trihydroquinoxaline 9e

Starting from compound **4c** and propargyl bromide, the title compound was prepared according to the general procedure III. The reaction was finished after 5 h, and the product was obtained after purification on a preparative TLC plate using ethyl acetate-cyclohexane 1/6 mixture as an eluent. **9e** was isolated as white solid in 40% yield.

^{1}H NMR (400 MHz, DMSO-*d*$_6$) δ 7.36 - 7.29 (m, 4H) 6.63 (dd, *J* = 8.8, 5.3 Hz, 1H), 6.41 (td, *J* = 8.6, 2.8, Hz, 1H), 6.32 (dd, *J* = 9.9, 2.8 Hz, 1H), 4.64 (dd, *J* = 8.0, 3.4 Hz, 1H), 4.14 - 3.99 (m, 2H), 3.87 (dd, *J* = 17.9, 2.4 Hz, 1H), 3.28 (dd, *J* = 11.2, 3.3 Hz, 1H), 3.17 (dd, *J* = 11.2, 8.0 Hz, 1H), 2.18 (t, *J* = 2.3 Hz, 1H).

^{13}C NMR (101 MHz, DMSO-*d*$_6$) δ 157.4 (d, *J* = 236.1 Hz), 140.0, 136.4 (d, *J* = 10.2 Hz), 133.9, 129.0, 128.8 (d, *J* = 2.4 Hz), 128.4, 113.9 (d, *J* = 9.2 Hz), 104.0 (d, *J* = 22.2 Hz), 101.1 (d, *J* = 26.1 Hz), 78.7, 72.8, 54.8, 54.3, 41.2.

^{19}F NMR (376 MHz, DMSO-*d*$_6$) δ -124.42

HRMS (APCI+) calc for $[C_{17}H_{14}F_2ClN_2]^+$: 300.0824, found 300.0827 $[M]^+$.

3-(4-Chlorophenyl)-7-fluoro-1-(methylsulfonyl)-2,3,4-trihydroquinoxaline 10a

Starting from compound **3c** and methansulfonyl chloride, the title compound was prepared according to the general procedure V. The product was obtained after purification on a preparative TLC plate using ethyl acetate-cyclohexane 1/2 mixture as an eluent. **10a** was isolated as yellow solid in 60% yield.

^{1}H NMR (400 MHz, Chloroform-*d*) δ 7.40 - 7.30 (m, 5H), 6.76 (td, *J* = 8.6, 2.4, Hz, 1H), 6.64 (dd, *J* = 8.8, 5.3 Hz, 1H), 4.46 (dd, *J* = 9.3, 3.3 Hz, 1H), 4.19 (m, 2H), 3.31 (dd, *J* = 13.6, 9.2 Hz, 1H), 2.85 (s, 3H).

^{13}C NMR (101 MHz, Chloroform-*d*) 155.6 (d, *J* = 243.3 Hz), 137.9, 134.5, 133.1, 129.3, 128.3, 122.6 (d, *J* = 10.1 Hz), 115.6 (d, *J* = 8.4 Hz), 113.0 (d, *J* = 23.4 Hz), 110.3 (d, *J* = 26.7 Hz), 53.5, 50.5, 39.2.

^{19}F NMR (376 MHz, Chloroform-*d*) δ -124.4

HRMS (ESI+) calc for $[C_{15}H_{14}ClFN_2O_2S+H]^+$: 341.521 found 341.520 $[M+H]^+$.

Quinoxalines

Quinoxaline derivatives were obtained as side products of tetrahydroquinoxaline alkylation, and they were obtained as a first fraction from the chromatographic purification of respective derivatives.

6-Fluoro-2-(3-fluoro-4-methylphenyl)quinoxaline 3b-Ar

On the attempts to alkylate **3b** in the double alkylation procedure IV, the compound **3b-Ar** was isolated as a main product of the reaction in 50% yield as white crystals.

^{1}H NMR (400 MHz, Chloroform-*d)* δ 9.28 (s, 1H), 8.14 (dd, J = 9.2, 5.7 Hz, 1H) 7.91 -7.83 (m, 2H), 7.75 (dd, *J* = 9.0, 2.8 Hz, 1H), 7.59 – 7.54 (m, 1H), 7.37 (t, *J* = 7.73 Hz, 1H), 2.38 (d, *J* = 1.7 Hz, 3H).

^{13}C NMR (101 MHz, Chloroform-*d*) δ 162.4 (d, *J* = 253.3 Hz), 162.0 (d, *J* = 242.9 Hz), 150.1, 143.7, 142.4 (d, *J* = 13.0 Hz), 139.5, 136.1 (d, *J* = 7.4 Hz), 132.3 (d, J = 5.3 Hz), 131.8 (d, J = 17.3 Hz) 127.5 (d, J = 17.3 Hz), 122.7 (d, *J* = 3.3 Hz), 121.0 (d, *J* = 26.0 Hz), 113.9 (d, *J* = 24.0 Hz), 112.8 (d, J = 21.6 Hz), 14.8.

^{19}F NMR (376 MHz, Chloroform-*d*) δ -108.2, -115.9.

HRMS (APCI+) calc for $[C_{15}H_{11}N_2F_2+H]^+$: 257.0884 found 257.0879 $[M+H]^+$.

2-(4-Chlorophenyl)-7-fluoroquinoxaline 4c-Ar

On the attempts to alkylate **4c** in the N-alkylation procedure III, the compound **4c**-Ar was isolated as a side product of the reactions in 10-30% yield.

^{1}H NMR (400 MHz, DMSO-*d6*) δ 9.56 (s, 1H), 8.34 (d, *J* = 8.4 Hz, 2H), 8.17 (dd, *J* = 9.2, 5.9 Hz, 1H), 7.88 (dd, *J* = 9.6, 2.8 Hz, 1H), 7.76 (ddd, *J* = 9.3, 8.5, 2.9 Hz 1H), 7.64 (d, *J* = 8.4 Hz, 2H).

^{13}C NMR (101 MHz, DMSO *d6*) δ 162.4 (d, *J* = 249.5 Hz), 150.6, 143.1 (d, *J* = 3.0 Hz), 142.1 (d, *J* = 13.6 Hz), 138.6, 135.8, 134.6, 131.4 (d, *J* = 10Hz), 129.4, 129.3, 120.2 (d, *J* = 26.1 Hz), 112.6 (d, *J* = 21.4 Hz).

^{19}F NMR (376 MHz, DMSO-*d6*) δ-107.9.

HRMS (APCI+) calc for $[C_{14}H_8N_2FCl\ +H]^+$ calc 259.0432, found 259.0438 $[M+H]^+$.

4-(Methylsulfonyl)-3-phenyl-3,4-dihydroquinoxalin-2(1H)-one 11a

Starting from compound **6a** and methansulfonyl chloride, the title compound was prepared according to the general procedure V. The product was isolated by preparative TLC using ethyl acetate/cyclohexane 1/4 mixture as eluent. **11a** was isolated as white powder in 25% yield.

^{1}H NMR (400 MHz, Chloroform-*d*) δ 8.67 (bs, 1H), 7.67 (dd, *J* = 8.1, 1.4 Hz, 1H), 7.39 – 7.36 (m, 2H), 7.28 – 7.26 (m, 3H), 7.19 (dt, *J* = 7.8, 1.3 Hz 1H), 7.09 (dt, *J* = 8.0, 1.9 Hz, 1H), 6.86 (dd, *J* = 7.9, 1.4 Hz 1H), 6.00 (s, 1H), 2.09 (s, 3H).

^{13}C NMR (101 MHz, Chloroform-*d*) δ 167.1, 133.2, 130.6, 128.9, 128.7, 128.0, 127.3, 126.4, 124.7, 123.3, 116.2, 64.4, 38.83.

HRMS (ESI+) calc for $[C_{15}H_{14}N_2O_3S+H]^+$: 303.0798; found 303.0797 $[M+H]^+$.

4-Benzyl-3-phenyl-3,4-dihydroquinoxalin-2(1H)-one 11b

Starting from compound **6a** and benzyl bromide, the title compound was prepared according to the general procedure III. The product was obtained after purification by crystallization with ethyl acetate. **11b** was isolated as white crystals in 33% yield.

^{1}H NMR (400 MHz, Chloroform-*d*) δ 10.02 (bs, 1H), 7.46 – 7.38 (m, 1H), 7.36 – 7.27 (m, 7H), 7.24 – 7.20 (m, 2H), 6.98 – 6.94 (m, 1H), 6.85 (dd, *J* = 7.7, 1.6 Hz, 1H), 6.77 – 6.72 (m, 2H), 5.01 (s, 1H), 4.67 (d, *J* = 15.2 Hz, 1H), 4.13 (d, *J* = 15.2 Hz, 1H).

^{13}C NMR (101 MHz, Chloroform-*d*) δ 166.8, 137.1, 136.5, 134.4, 129.0, 128.9, 128.6, 127.8, 127.7, 127.3, 125.3, 124.6, 118.9, 115.6, 112.4, 65.3, 51.8.

HRMS (APCI+) calc for $[C_{21}H_{19}N_2O]^+$: 314.1414; found 314.1412 $[M]^+$.

X-Ray: M = 314.39 g.mol^{-1}, monoclinic system, space group $P2_1/c$, a = 15.3156 (3) Å, b = 15.0662 (3) Å, c = 7.05713 (14) Å, β = 91.6862 (18)°, Z = 4, V = 1627.72 (7) Å^3, D_c = 1.283 g.cm^{-3}, μ(Cu-Kα) = 0.63 mm^{-1}, crystal dimensions of 0.43 × 0.30 × 0.07 mm. The structure was deposited into Cambridge Structural Database under number CCDC 2505537.

1.3 Chemical stability

Samples of **3a**, **7d** and **9a** were prepared diluting 3 mg of each compound in 2 mL of acetonitrile. The respective solutions were separated into 2 vials, containing 1 mL each. After initial measurement by UPLC (see section Chemistry - General) one vial for each compound was treated with 10 µL of 1M HCl, while to the other one 1 mg of NaH (60% in paraffin) was added to the solution. Immediately after addition, each sample was measured by UPLC. The samples were left in the UPLC autosampler (25° C) for 24 h and measured again.

1.4 Animal strain and husbandry

All breeding and experimental procedures were undertaken in accordance with the Association for Research for Vision and Ophthalmology Statement for the Use of Animals in Ophthalmic and Research. All rodent experiments are fully covered under the ethics application 3909-2023, approved by Stockholm's Committee for Ethical Animal Research. Animals were housed and fed in a 12h light/12h dark cycle with food and water available *ad libitum*. Male C57BL/6J mice (SCANBUR) were purchased at 10-12 weeks old and housed for 1-4 week before beginning experiments or bred at the facility and used at 10-16 weeks old.

1.5 Luminescence-based NAD quantification

C57BL/6J mice were euthanized through cervical dislocation and the whole cortex was removed and separated by the hemisphere. Each hemisphere (n = 4) was added to 800 µL dispase (Corning), finely chopped up and incubated at 37°C 350 rpm (Thermomixer C, Eppendorf). The samples were triturated until a homogenous single cell suspension was achieved. The cell suspensions were diluted to 2 million cells/mL and incubated with the compounds at 5 nM, 50 nM, 500 nM and 5 µM for 2 hours. For FK866 experiments, the samples were incubated with 100 µM FK866 for 1 hour, and then together with the compounds at 5 µM for 2 hours. After the incubation the samples were spun down (5 min, 3000 g), the supernatant was exchanged with equal volume of HBSS. Each sample was homogenized with a handheld homogenizer for 15 seconds (VDI 12, VWR). NAD luminescence assay was performed using luminescence-based kit (NAD/NADH Glo-, Promega). The reagents of the kit were prepared as per manufacturer's instructions. 50 µL of the samples and 50 µL of the NAD glo kit reagent were combined in a 96 well plate and the luminescence was measured. The luminescence count data was measured 50 minutes after the start of incubation of the reagent.

1.6 3D-Structure activity analysis (3D-SAR)

The 3D SAR studies and pharmacophore modelling were performed using the Activity Atlas and FieldTemplater modules implemented in Flare v10 (Cresset Inc., Cambridgeshire, UK) [5]. FieldTemplater [6] was run with equal weighting for field point and shape similarity (50 to 50) and produced 22 pharmacophore hypotheses from the three reference molecules **4g**, **R-4b**, and **7c**. The best template, which showed the highest overall 3D similarity (template similarity 0.79; field similarity 0.77; shape similarity 0.82), was selected as the reference pharmacophore for aligning the full dataset. For each ligand, conformational ensembles were generated using

the standard conformation hunt protocol implemented in Flare, employing an RMSD filter of 0.50 Å for duplicate removal, a ring library, trans handling for secondary amides, enumeration of undefined stereocentres, and retention of up to 100 conformers per molecule. All molecules were aligned to the pharmacophore hypothesis using the Dice metric with equal weighting for shape and fields; the field term comprised equally weighted positive, negative, and hydrophobic components. The best scoring combined field and shape conformation per ligand was retained for qualitative 3D SAR with Activity Atlas. Activity Atlas models were built using the standard protocol, employing the contribution weighted sum algorithm on a 1.0 Å grid with a distance dependent dielectric to generate qualitative maps that highlighted activity cliff regions. The 3D activity cliff maps were rendered at Confidence Level 4.5 (isosurface) to emphasise high confidence features and reduce the influence of weak or uncertain contributions.

1.7 Caco-2 permeability

Caco-2 cell monolayers (passage 94-105) were grown on permeable filter support and used for transport study on day 21 after seeding. Prior to the experiment a drug solution of 10µM was prepared and warmed to 37 C. The Caco-2 filters were washed with pre-warmed HBSS prior to the experiment, and thereafter the experiment was started by applying the donor solution on the apical or basolateral side. The transport experiments were carried out at pH 7.4 in both the apical and basolateral chamber. The experiments were performed at 37 °C and with a stirring rate of 500 rpm. The receiver compartment was sampled at 30 minutes, also a final sample from the donor chamber was taken to calculate the mass balance of the compound. The samples (100 µL) were transferred to a 96-well plate containing 100 µL methanol and Warfarin as internal standard and was sealed until LC-MS/MS. The experiments were performed by SciLifeLab, Sweden (https://www.scilifelab.se).

1.8 Metabolic stability in human, mouse and rat microsomes

Metabolic stability was determined in 0.5 mg/ml human (pool of 50 donors, XenoTech LLC, KS, USA), mouse (CD-1 mouse male, pool, XenoTech LLC, KS, USA) and rat (SD rat pool, XenoTech LLC, KS, USA) liver microsomes at a compound concentration of 1 µM in 100 mM KPO4 buffer pH 7.4 in a total incubation volume of 500 µL. The reaction was initiated by the addition of 1 mM NADPH. At various incubation times, i.e. at 0, 5, 10, 20, 40 and 60 min, a sample was withdrawn from the incubation and the reaction was terminated by addition of cold acetonitrile with warfarin (A2250, Sigma Aldrich) as an internal standard. The amount of parent compound remaining was analyzed by LC-MS/MS. The experiments were performed by SciLifeLab, Sweden (https://www.scilifelab.se).

1.9 LC-MS/MS

The test compound was optimized on a Waters Acquity UPLC XEVO TQ-S micro system (Waters Corp.) operating in multiple reaction monitoring (MRM) mode with positive or negative electrospray ionization. Compounds were optimized by using the QuanOptimize software (Waters Corp.). For chromatographic separation, a C18 BEH 1.7 µm column was used, with a general gradient of 1% to 90% of mobile phase B over a total running time of 2 min. Mobile phase A consisted of 5% acetonitrile and 0.1% formic acid in purified water, and mobile phase B of 0.1% formic acid in 100% acetonitrile. The flow rate was set to 0.5 ml/min and 5 µL of the sample was injected.

1.10 Analysis and statistics

All statistical analysis was performed in R. *Student's t*-test (two-sided) was used for statistical testing, * < 0.05, ** < 0.01, *** < 0.001, NS = non-significant (P > 0.05).

2. Spectral characterization

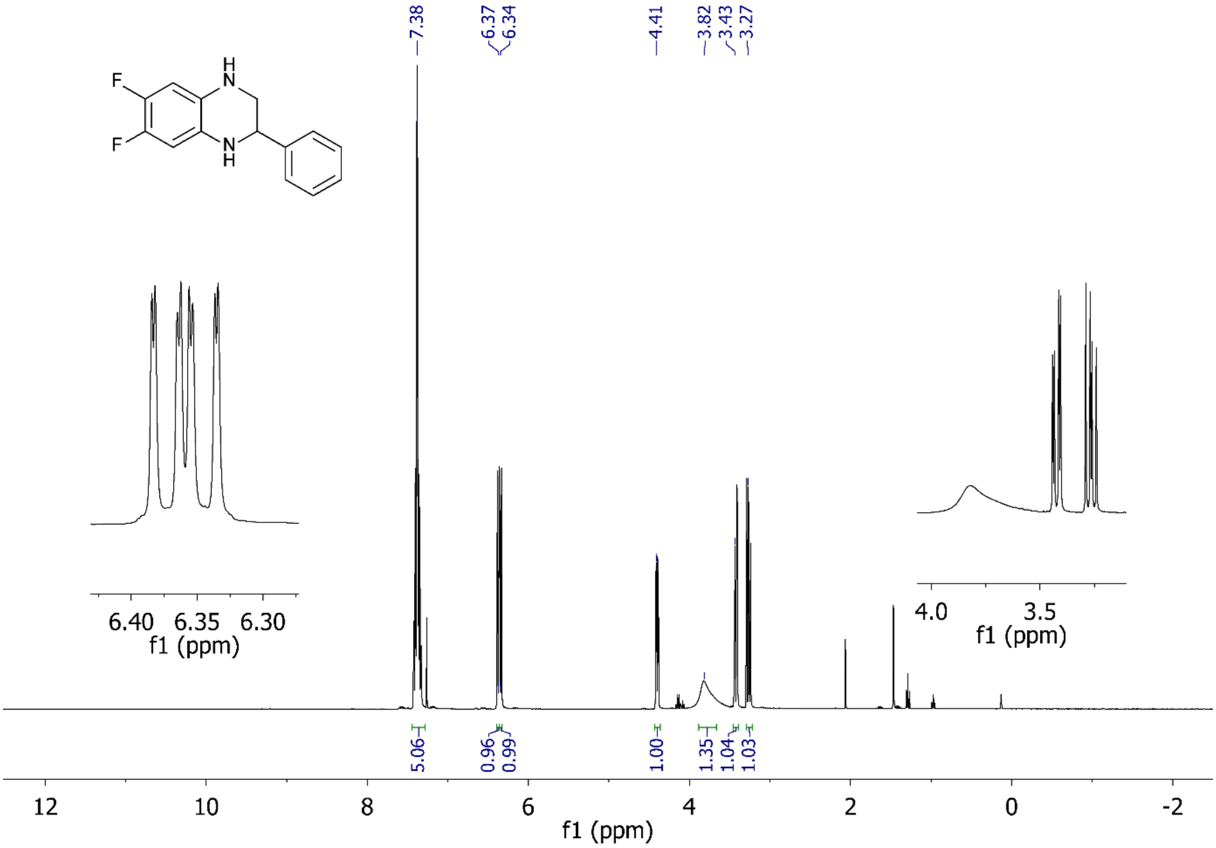

Figure S1. Compound **1**, ^{1}H NMR (400 MHz, $CDCl_3$).

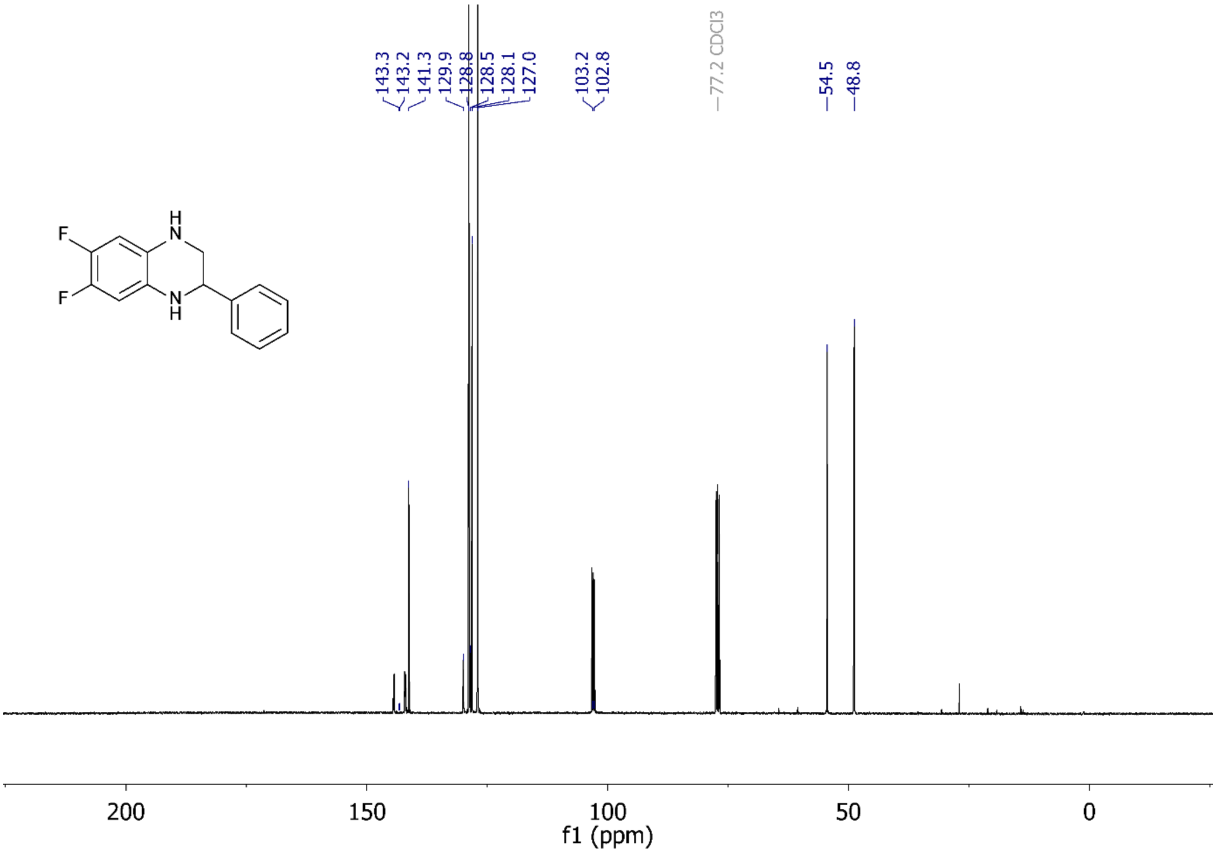

Figure S2. Compound **1**, ^{13}C NMR (101 MHz, $CDCl_3$).

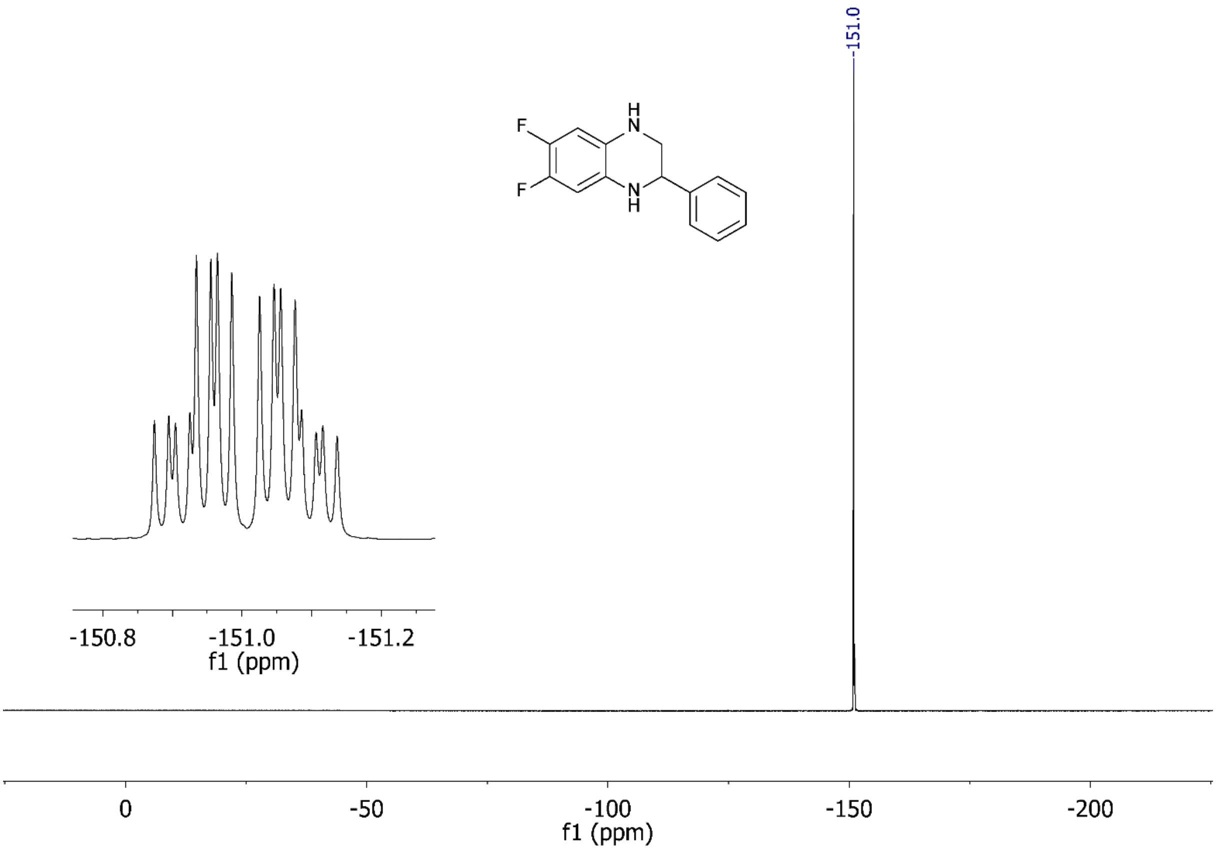

Figure S3. Compound **1**, ^{19}F NMR (376.5 MHz, $CDCl_3$).

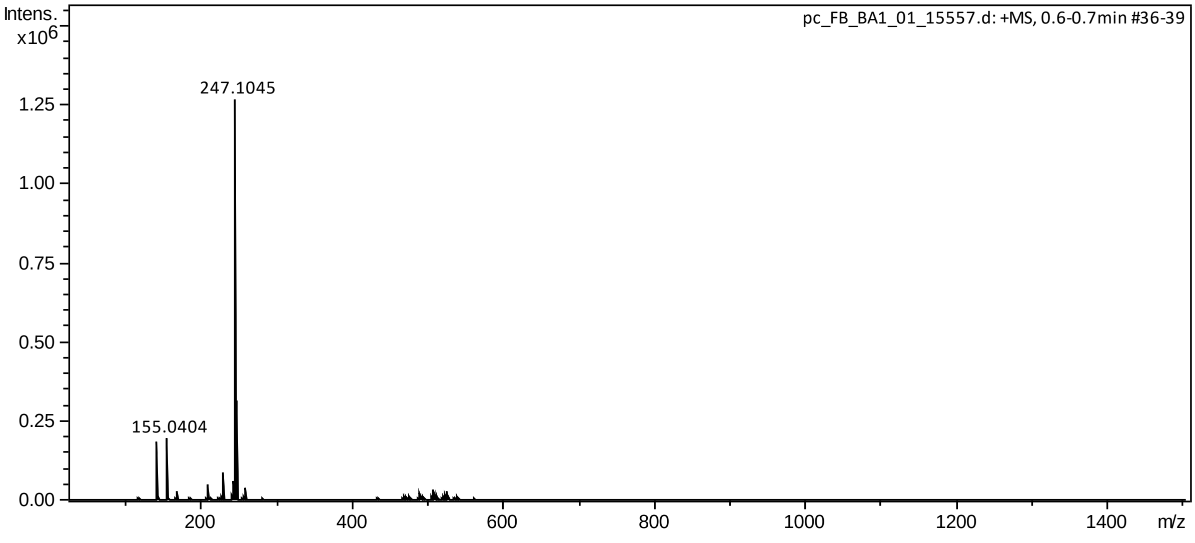

Figure S4. Compound **1**, HRMS (ESI+) calc for $[C_{14}H_{12}F_2N_2+H]^+$: 247.1041 found 247.1045 $[M+H]^+$.

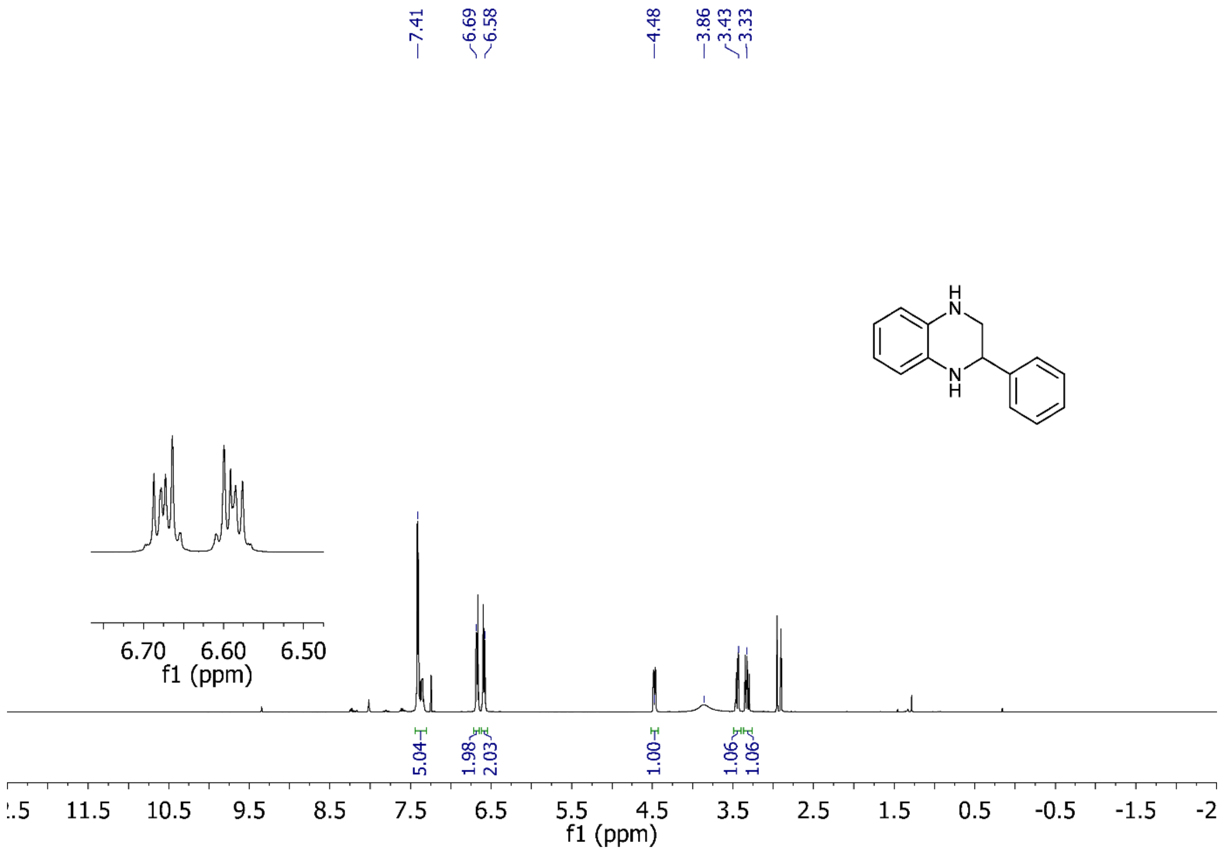

Figure S5. Compound **2a**, ^{1}H NMR (400 MHz, $CDCl_3$).

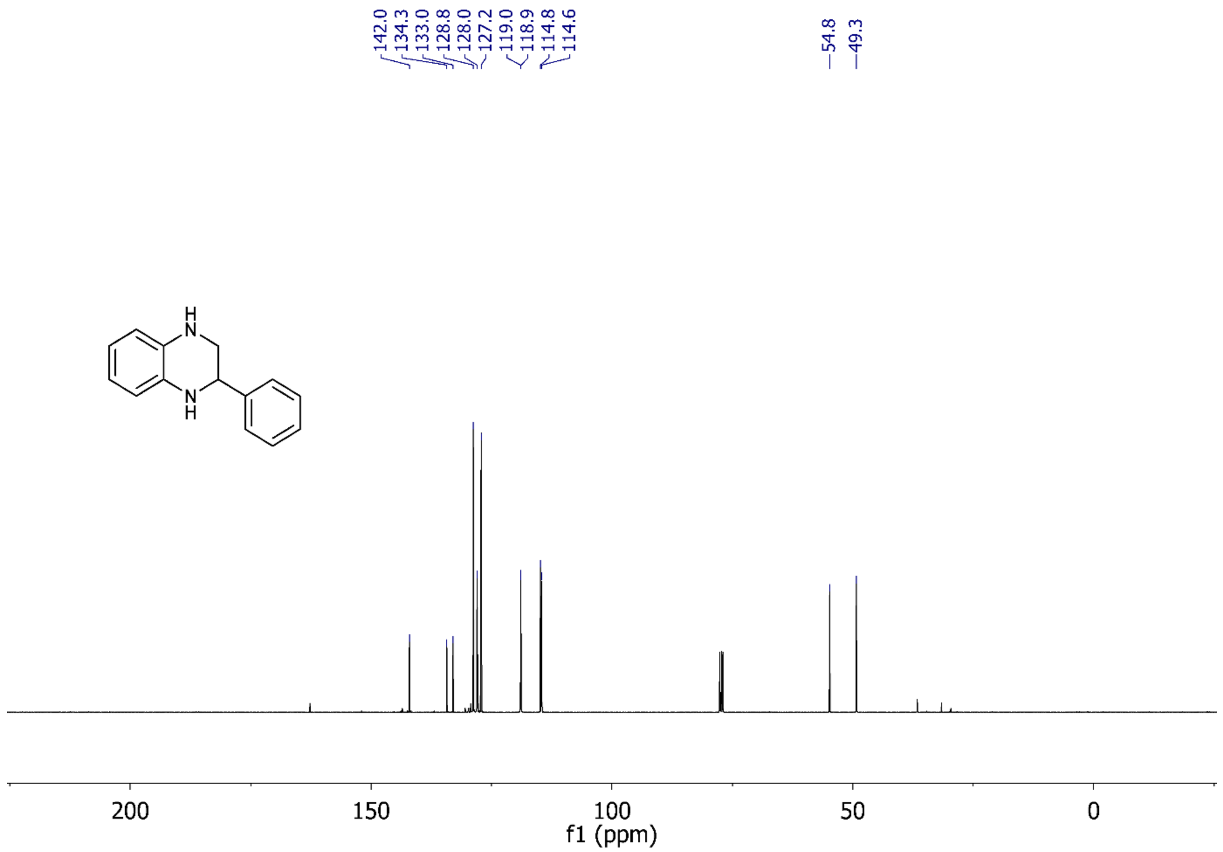

Figure S6. Compound **2a**, ^{13}C NMR (101 MHz, $CDCl_3$).

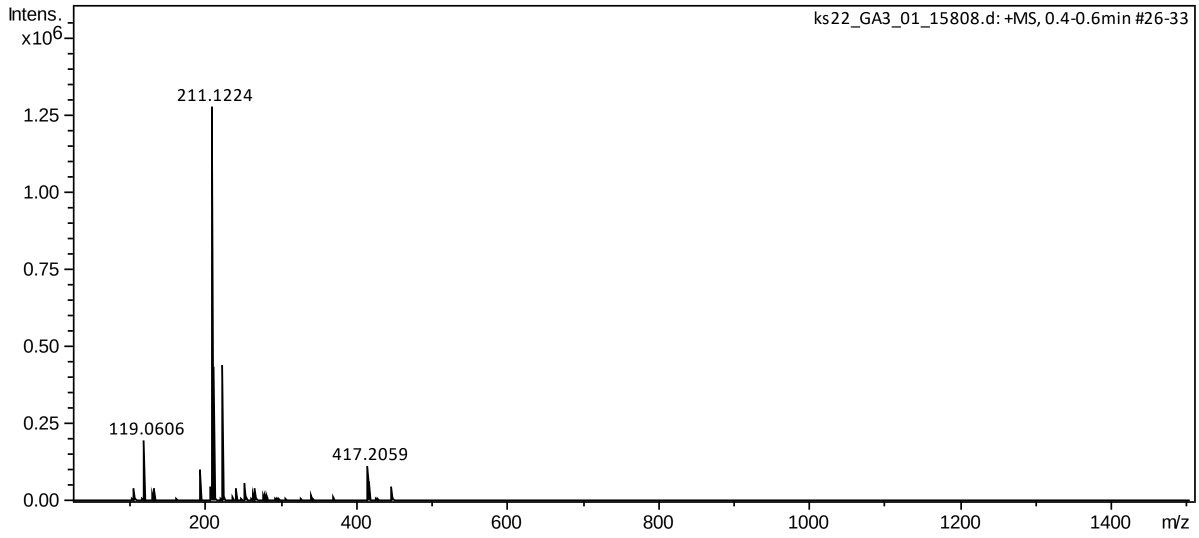

Figure S7. Compound **2a**, HRMS (ESI+) calc for $[C_{14}H_{14}N_2+H]^+$: 211.1229 found 211.1224 $[M+H]^+$.

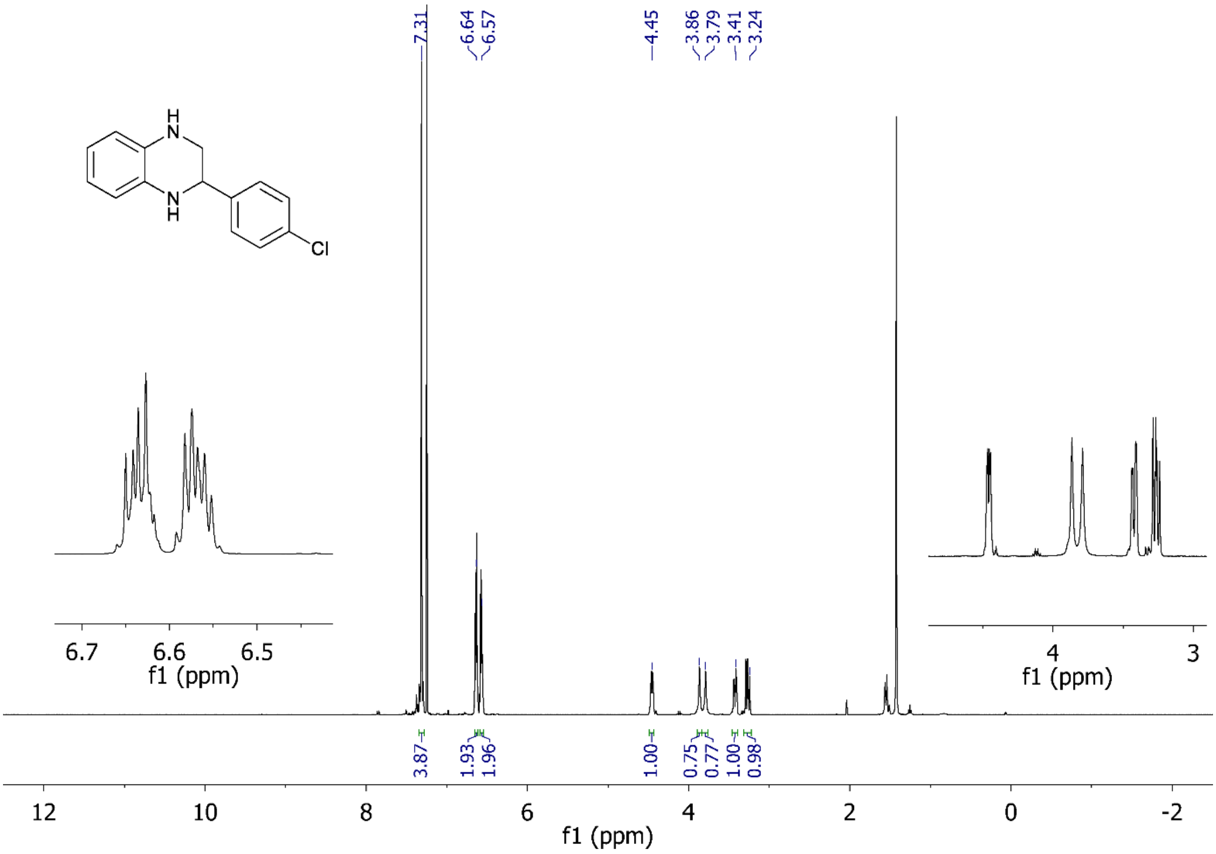

Figure S8. Compound **2b**, ^{1}H NMR (400 MHz, $CDCl_3$).

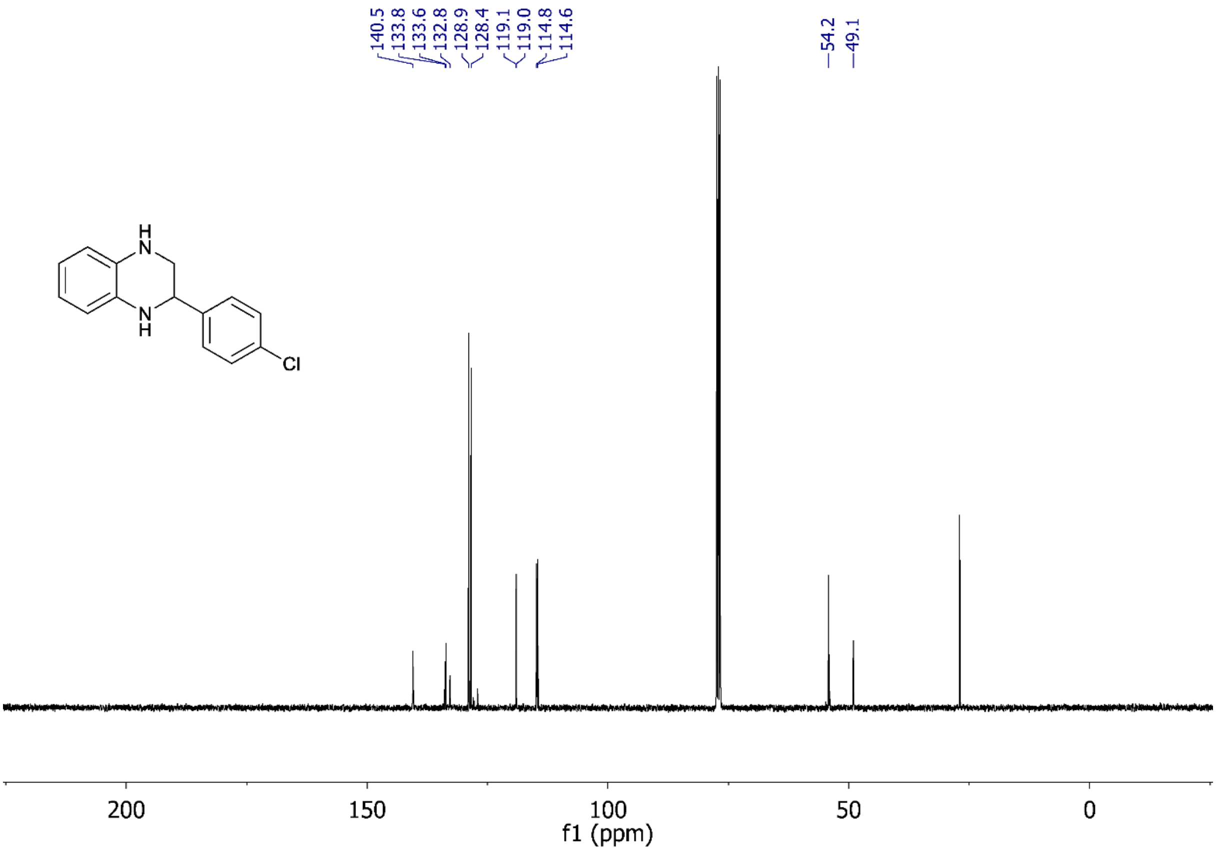

Figure S9. Compound **2b**, ^{13}C NMR (101 MHz, $CDCl_3$).

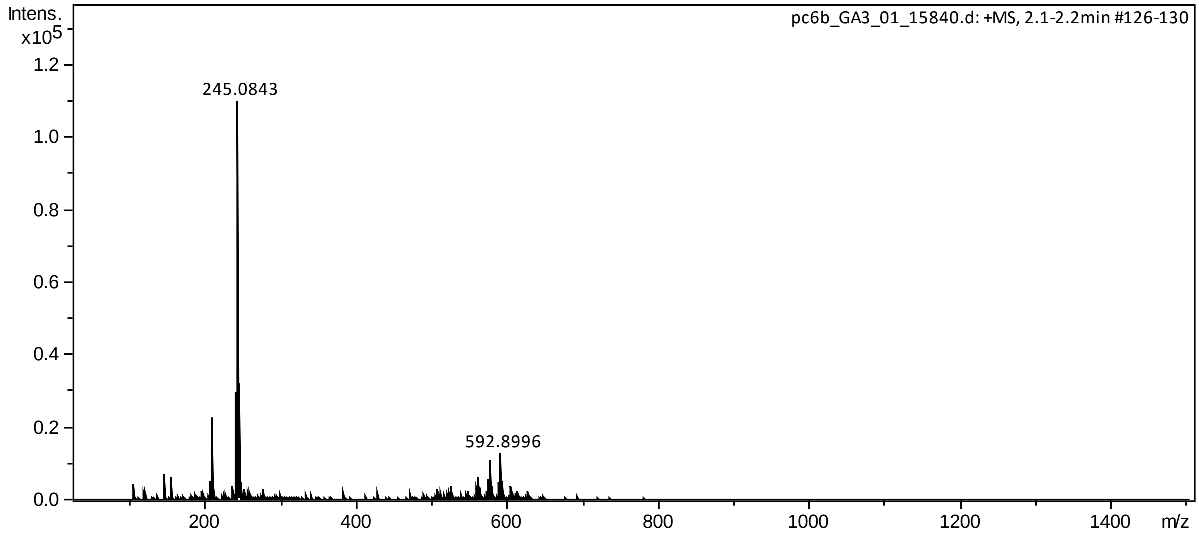

Figure S10. Compound **2b**, HRMS (ESI+) calc for $[C_{14}H_{13}ClN_2+H]^+$: 245.0840 found 245.0843 $[M+H]^+$.

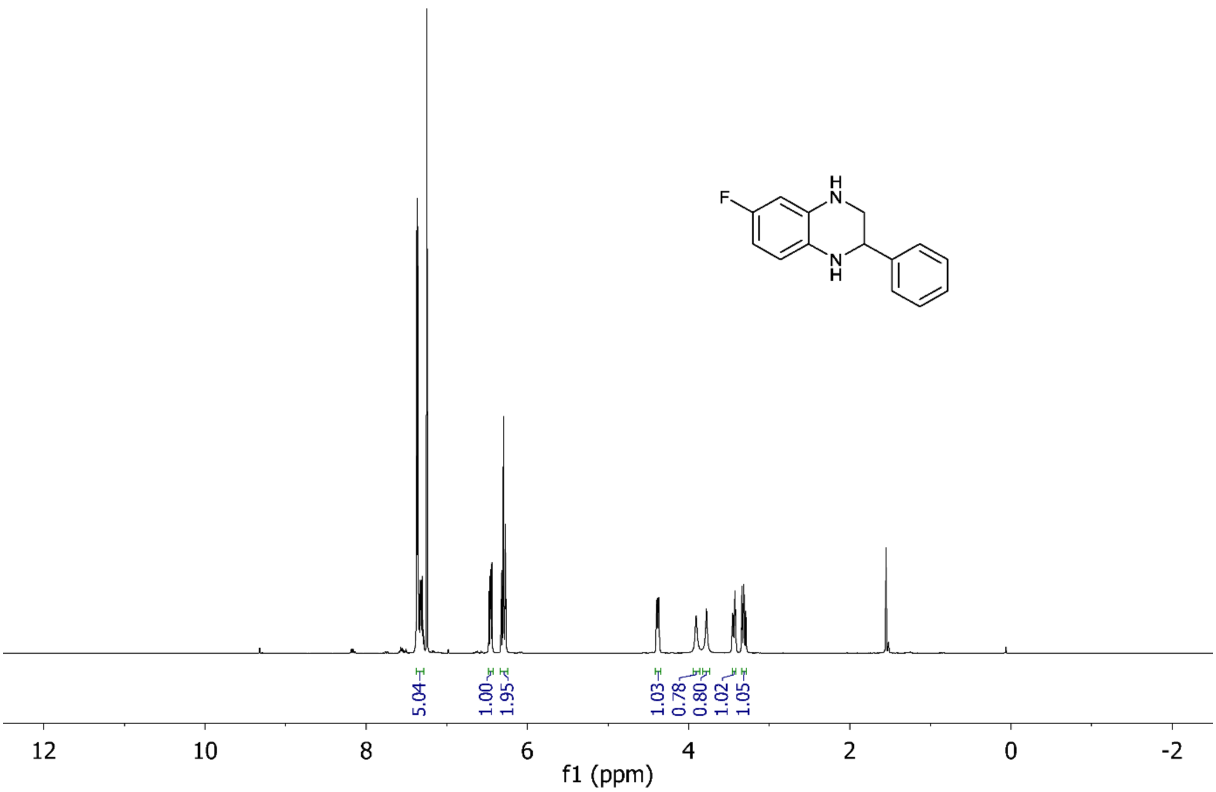

Figure S11. Compound **3a**, ^{1}H NMR (400 MHz, $CDCl_3$).

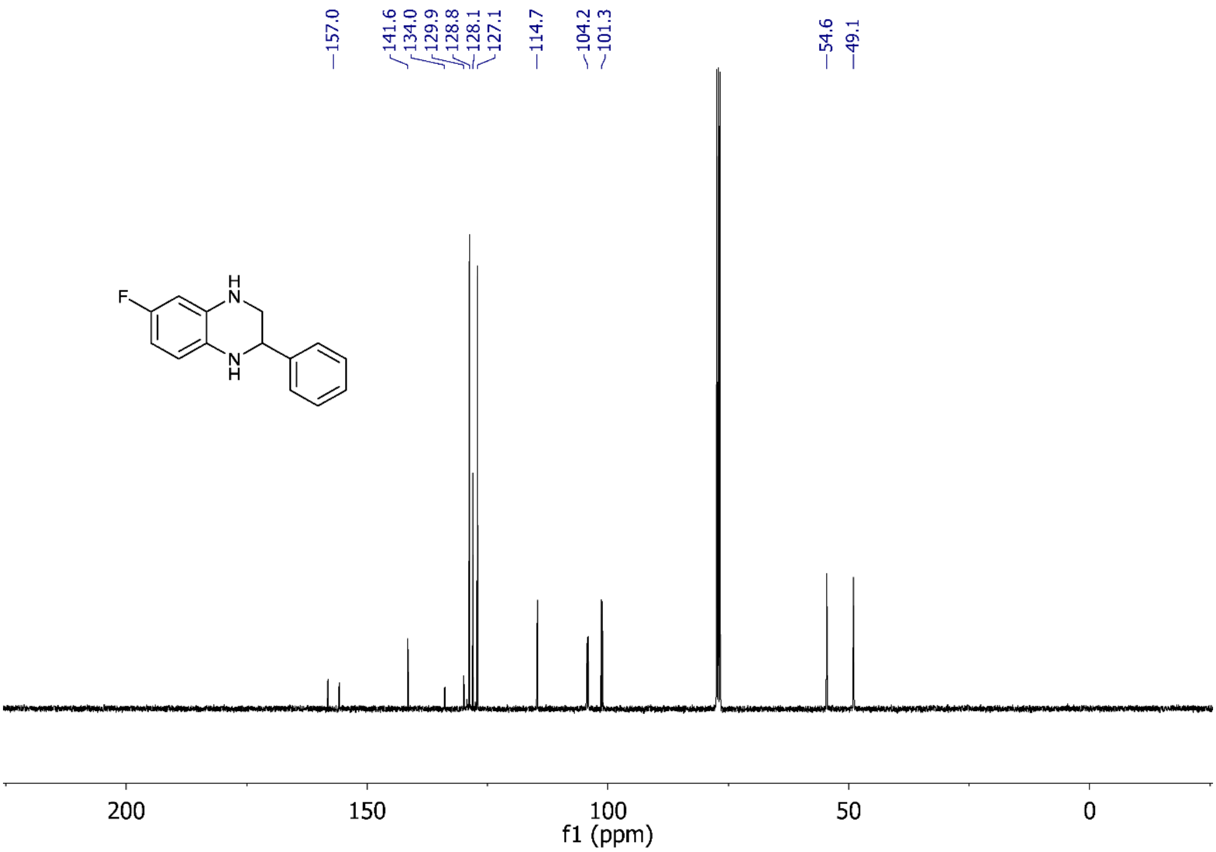

Figure S12. Compound **3a**, ^{13}C NMR (101 MHz, $CDCl_3$).

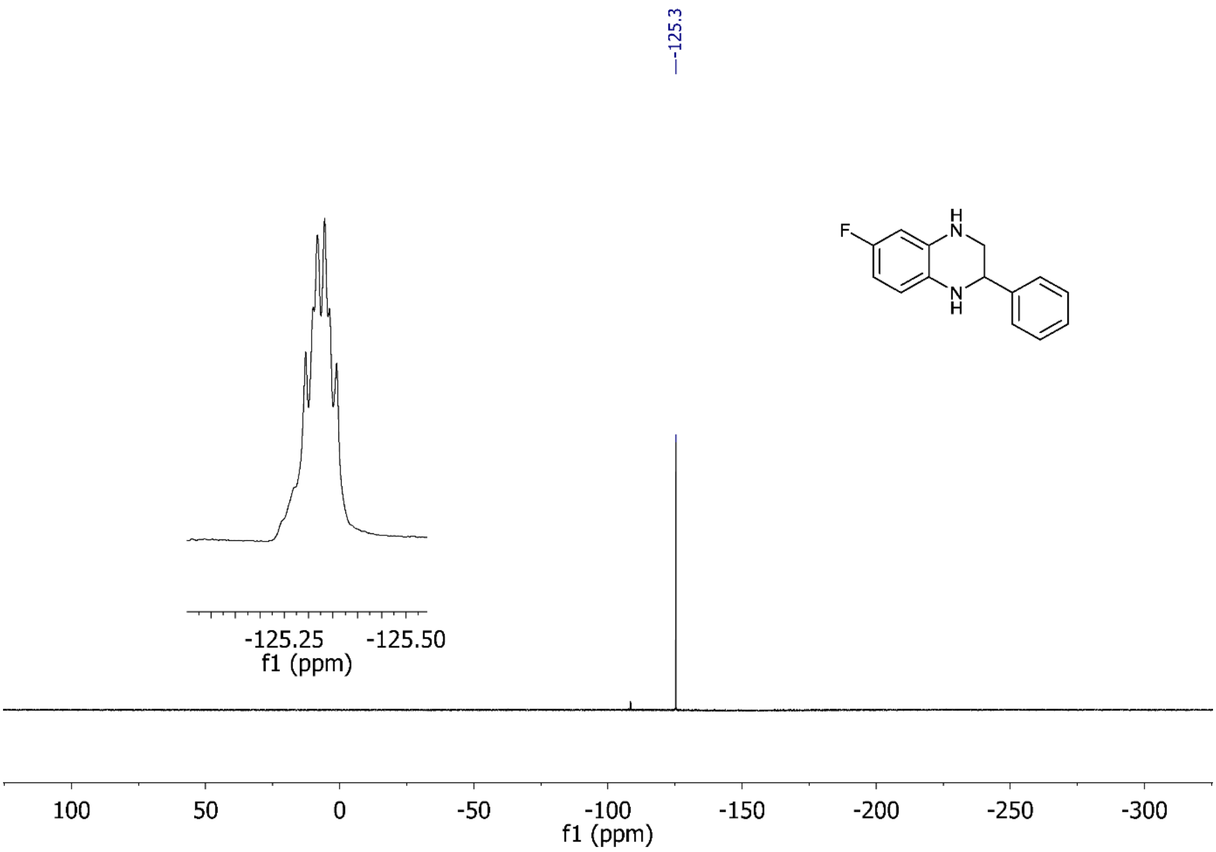

Figure S13. Compound **3a**, ^{19}F NMR (376.5 MHz, $CDCl_3$).

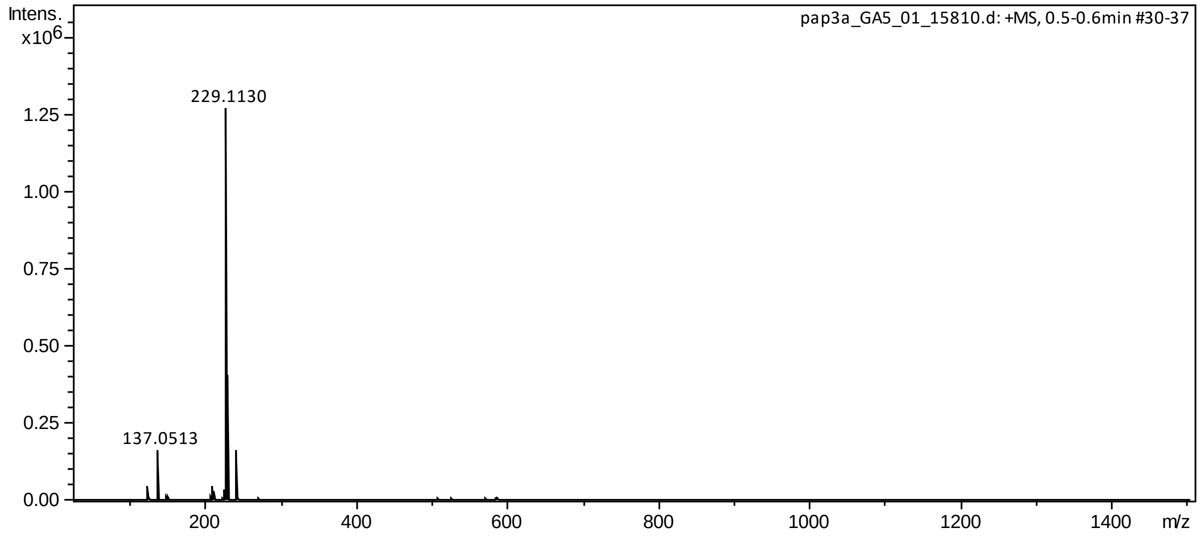

Figure S14. Compound **3a**, HRMS (ESI+) calc for $[C_{14}H_{13}FN_2+H]^+$: 229.1135 found 229.1130 $[M+H]^+$.

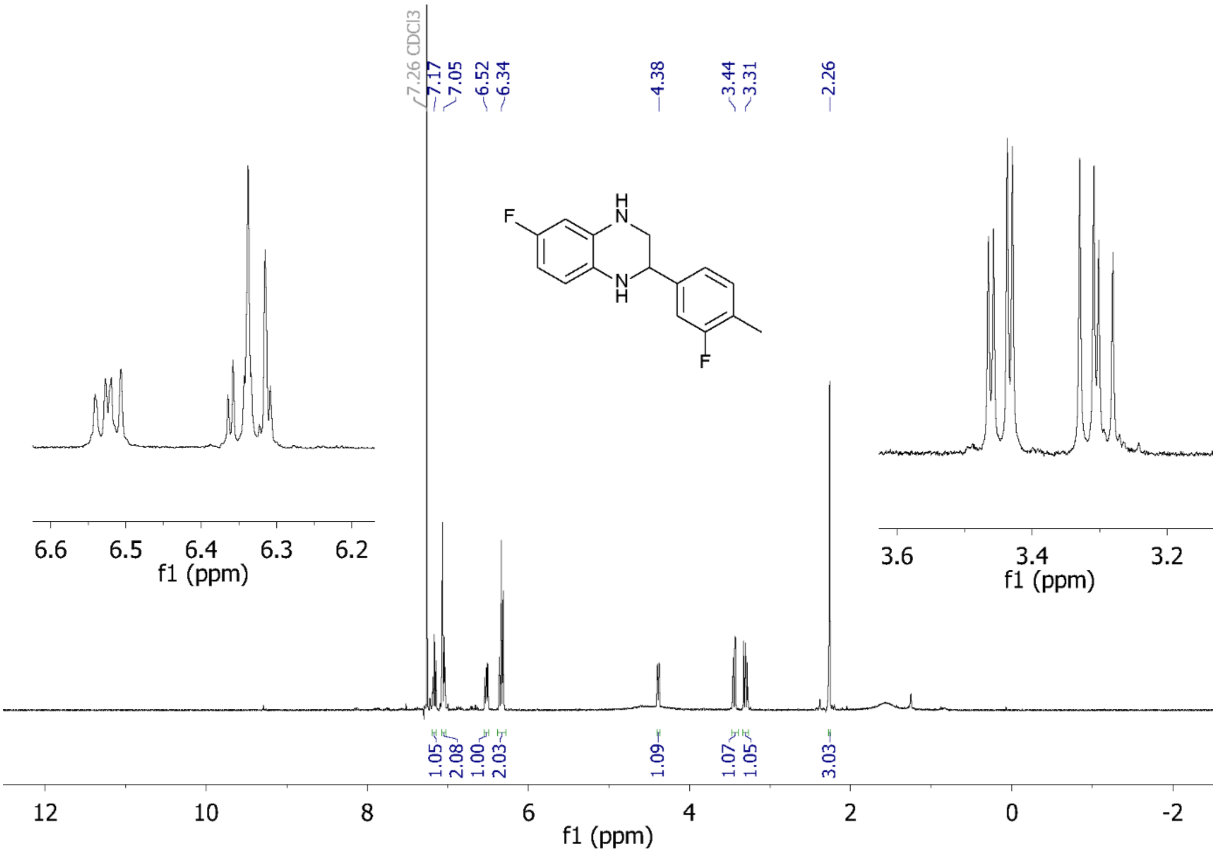

Figure S15. Compound **3b**, ^{1}H NMR (400 MHz, $CDCl_3$).

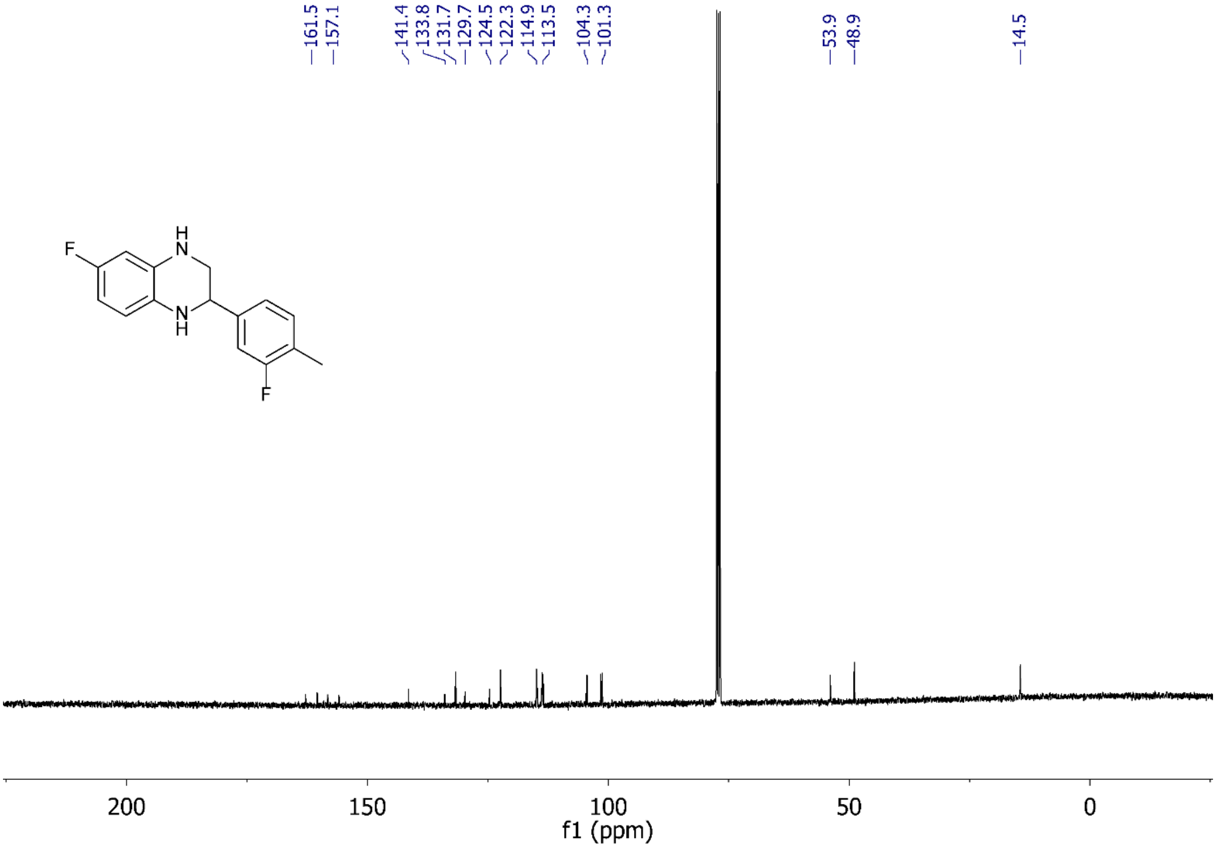

Figure S16. Compound **3b**, ^{13}C NMR (101 MHz, $CDCl_3$).

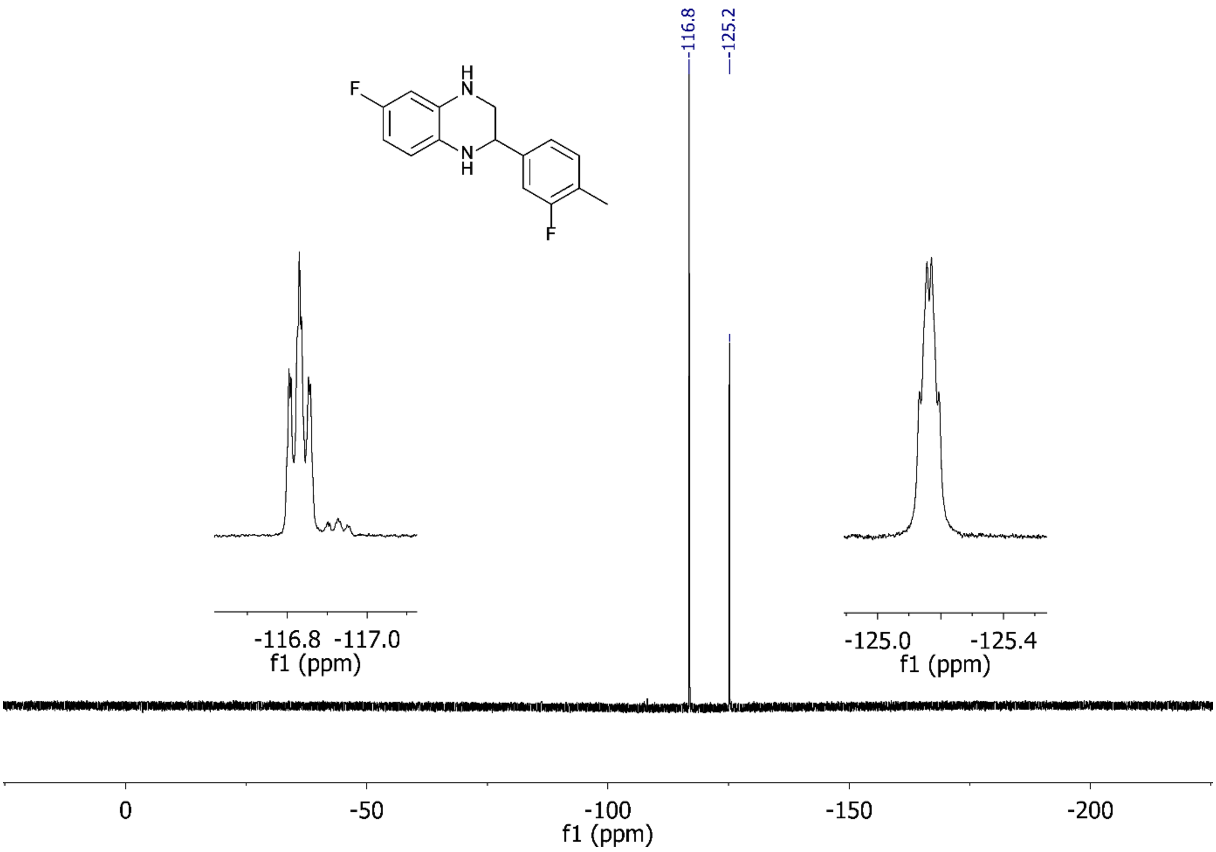

Figure S17. Compound **3b**, ^{19}F NMR (376.5 MHz, $CDCl_3$).

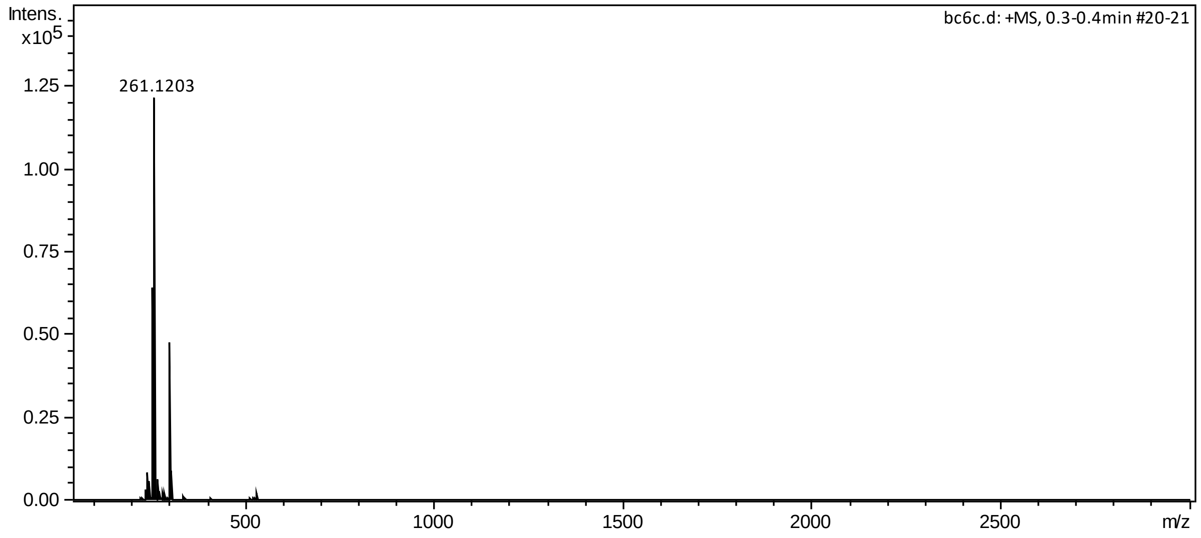

Figure S18. Compound **3b**, HRMS (APCI+) calc for $[C_{15}H_{14}N_2F_2+H]^+$: 261.1198 found 261.1203 $[M+H]^+$.

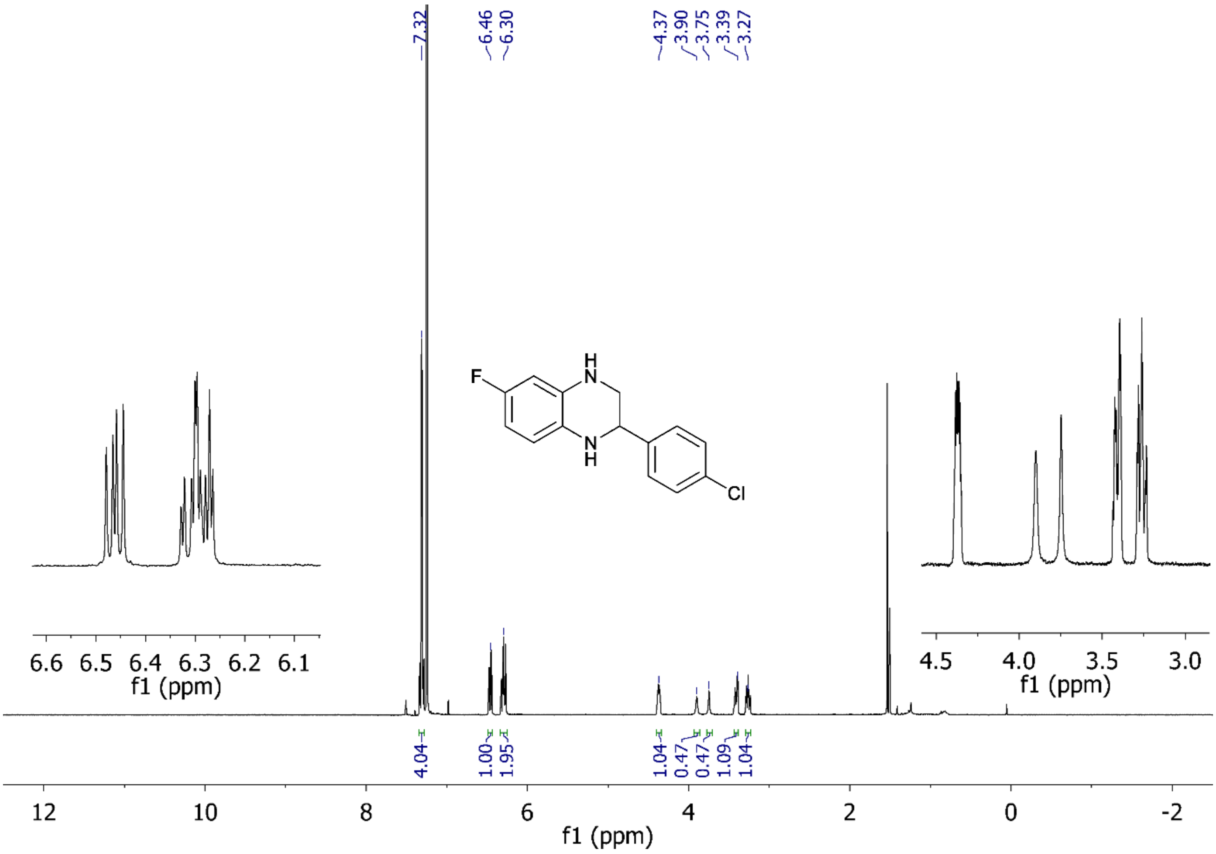

Figure S19. Compound **3c**, ^{1}H NMR (400 MHz, $CDCl_3$).

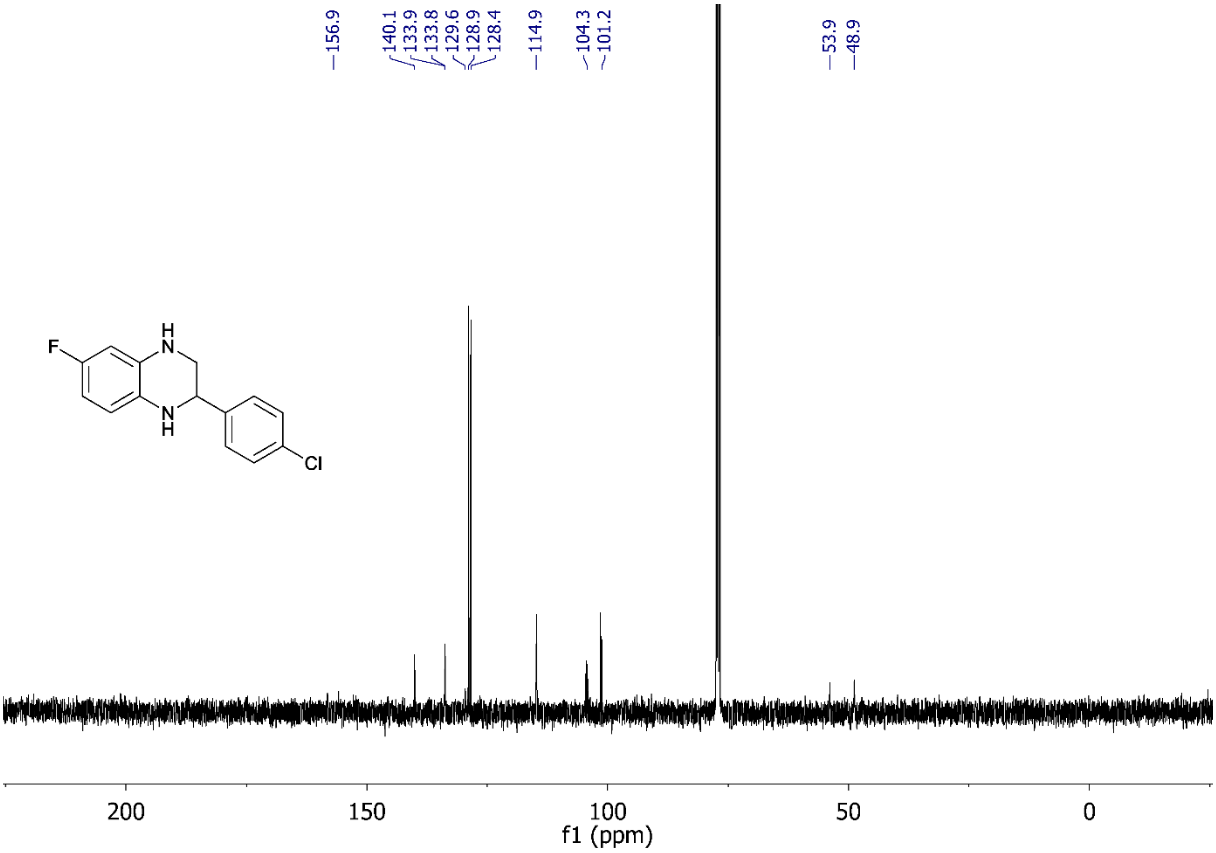

Figure S20. Compound **3c**, ^{13}C NMR (101 MHz, $CDCl_3$).

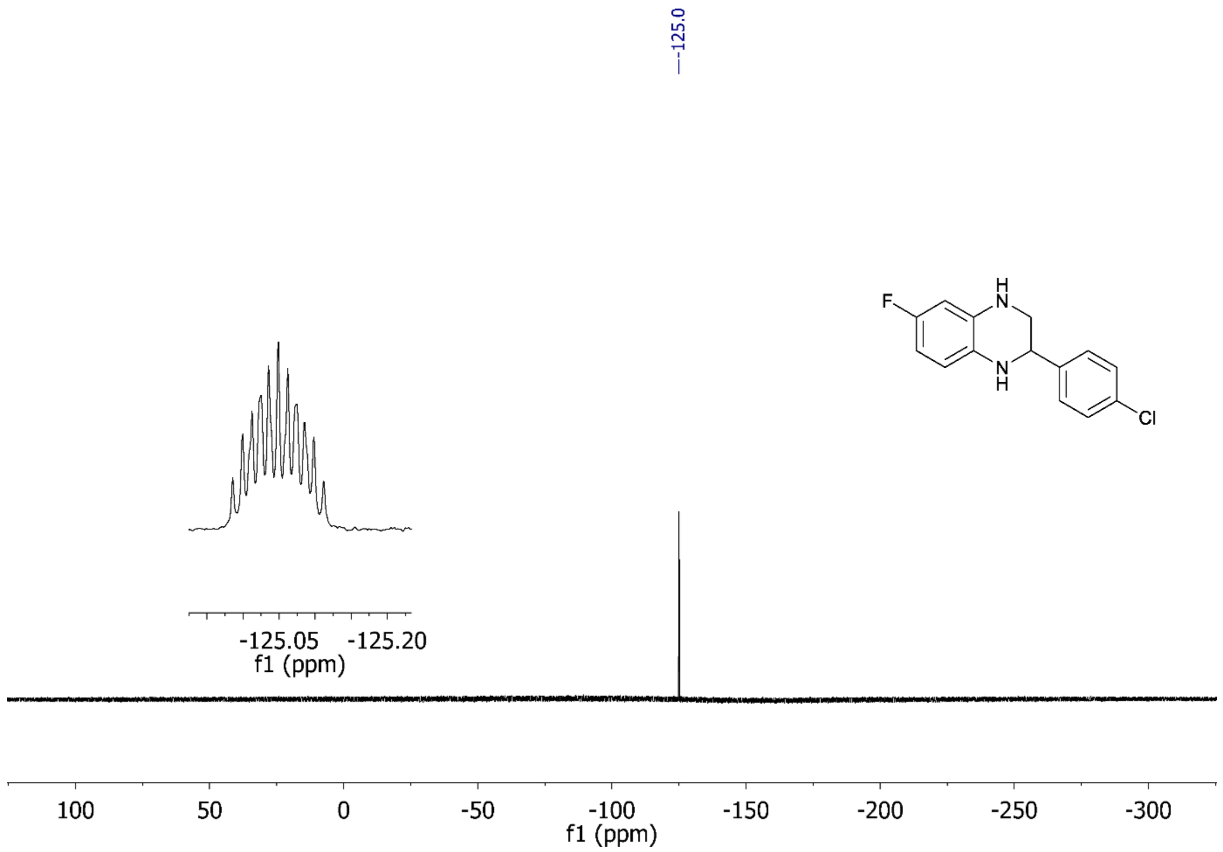

Figure S21. Compound **3c**, ^{19}F NMR (376.5 MHz, $CDCl_3$).

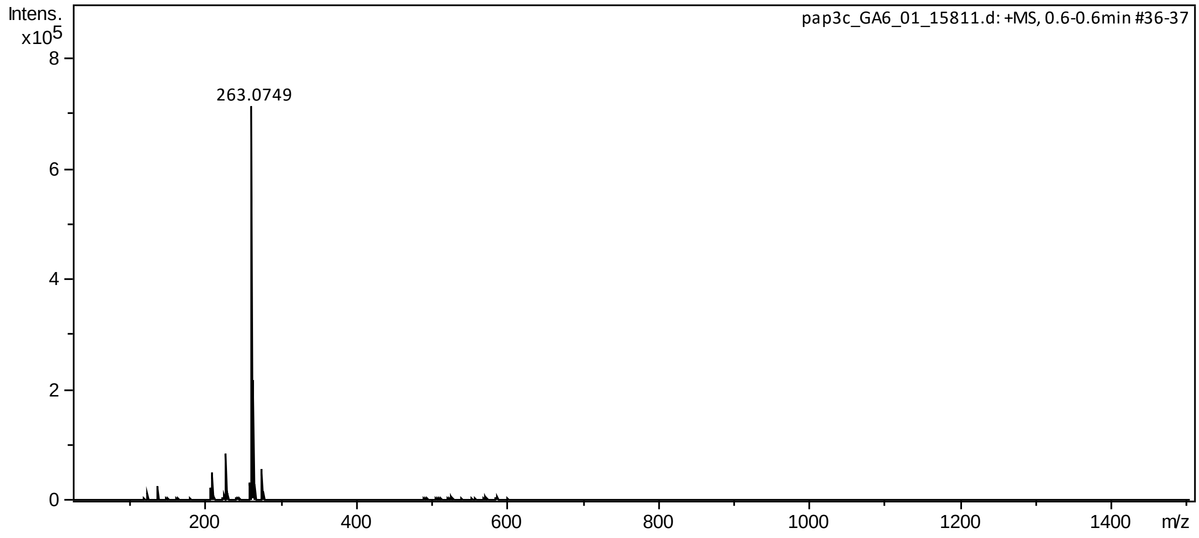

Figure S22. Compound **3c**, HRMS (ESI+) calc for $[C_{14}H_{12}ClFN_2+H]^+$:263.0746 found 263.0749 $[M+H]^+$.

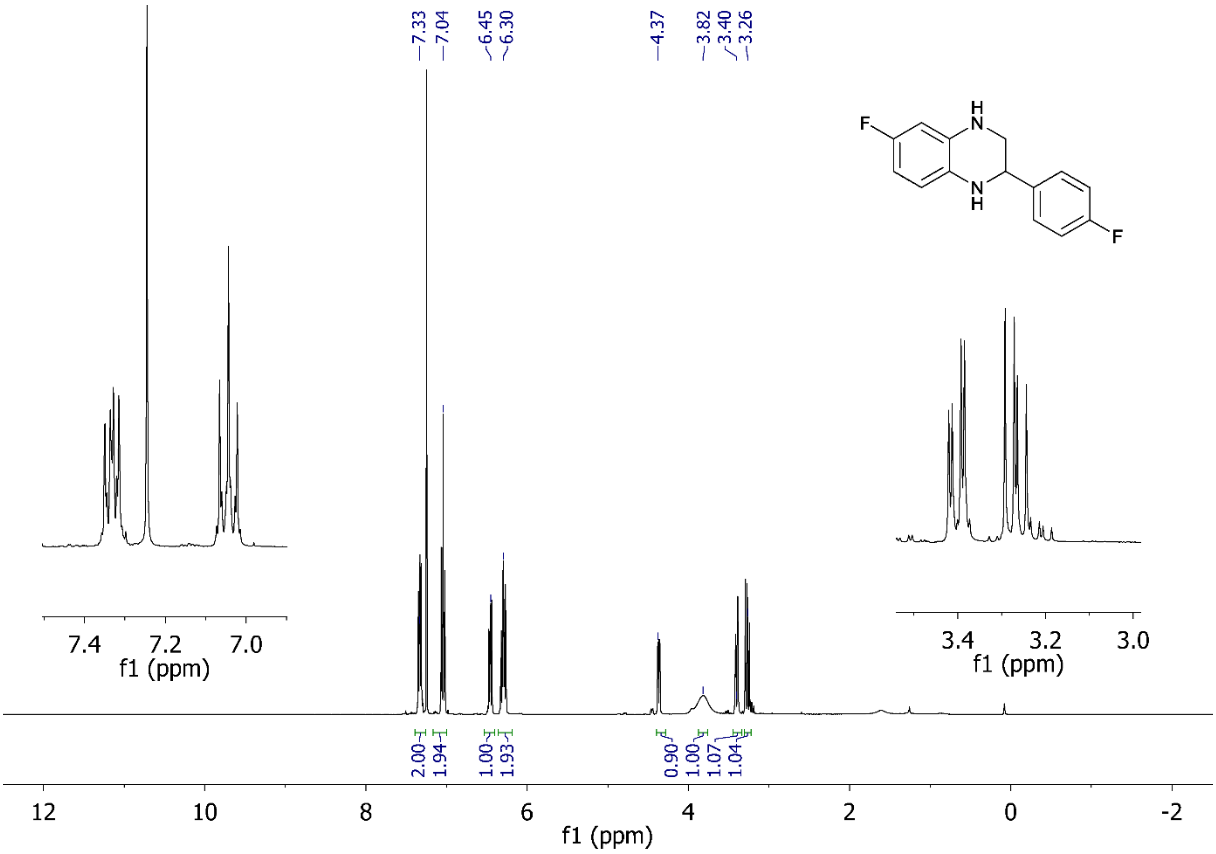

Figure S23. Compound **3d**, ^{1}H NMR (400 MHz, $CDCl_3$).

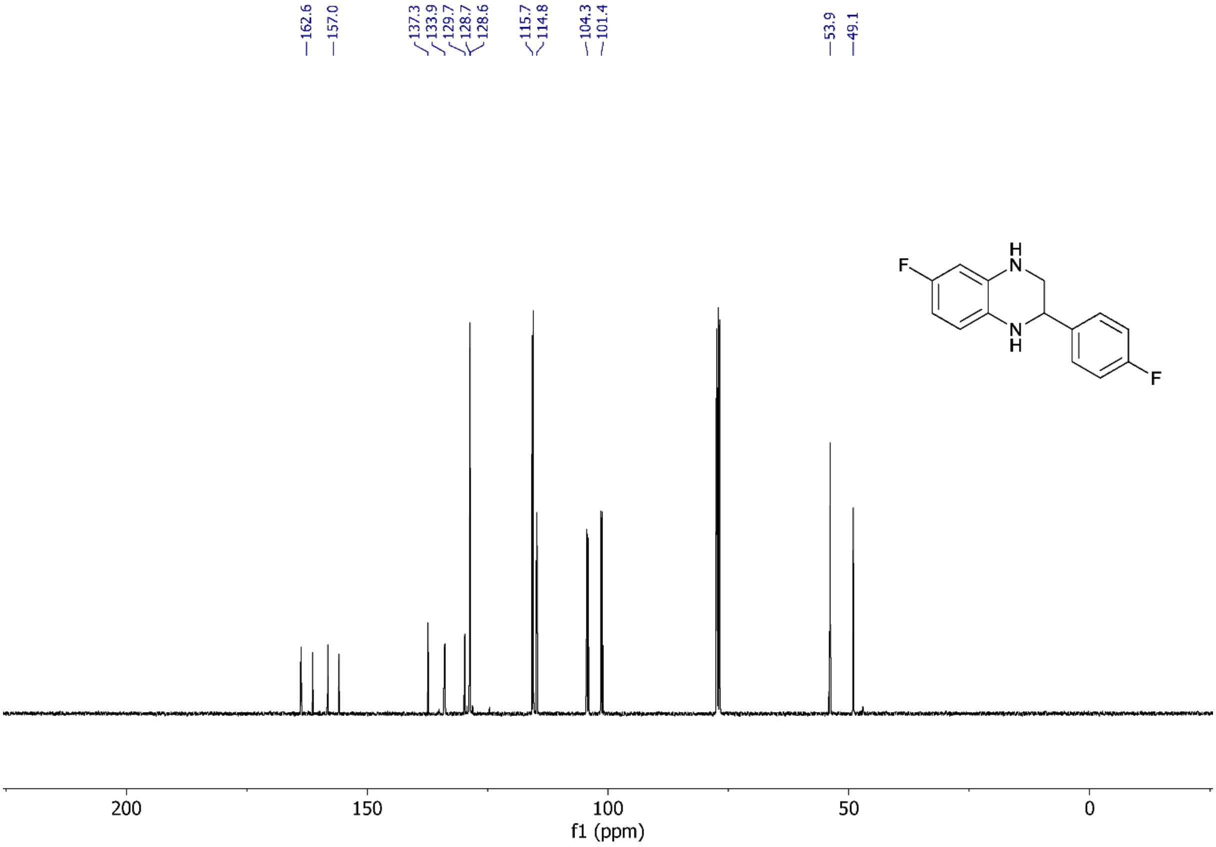

Figure S24. Compound **3d**, ^{13}C NMR (101 MHz, $CDCl_3$).

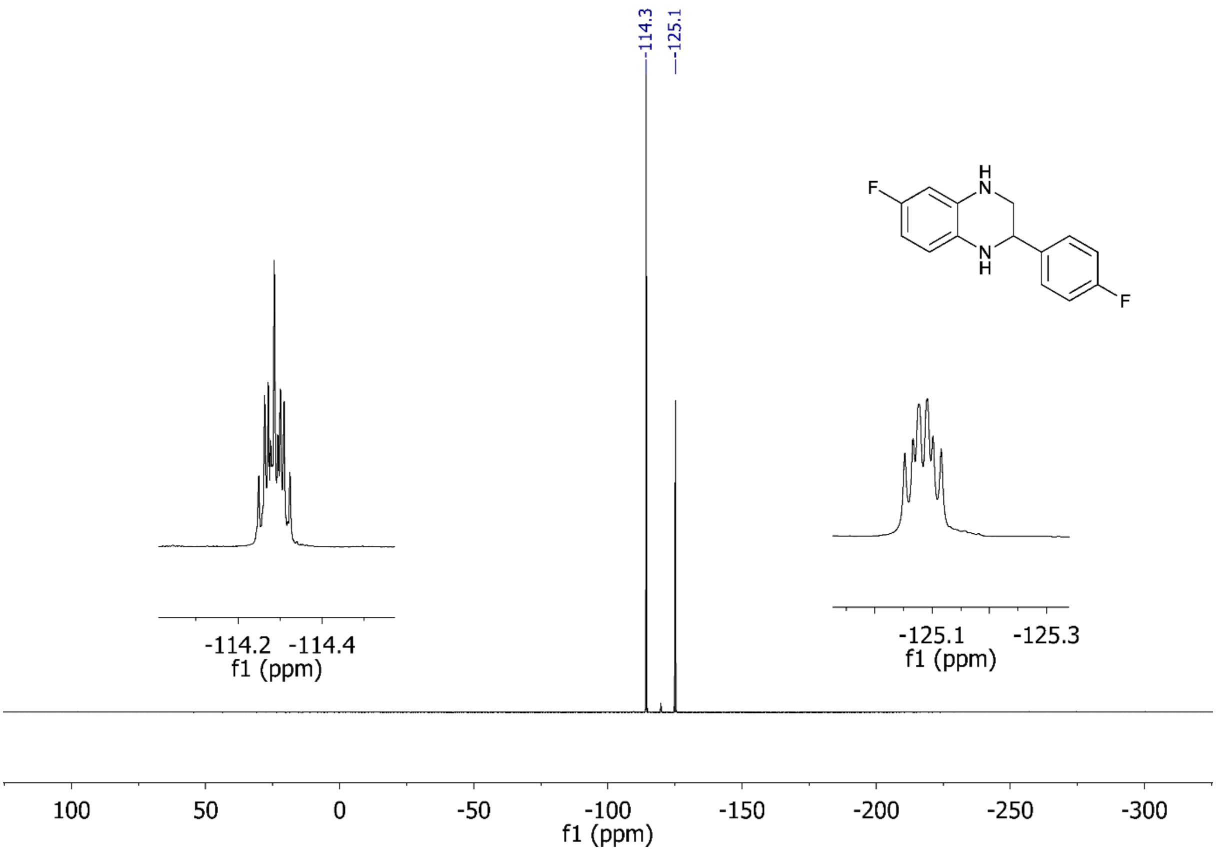

Figure S25. Compound **3d**, ^{19}F NMR (376.5 MHz, $CDCl_3$).

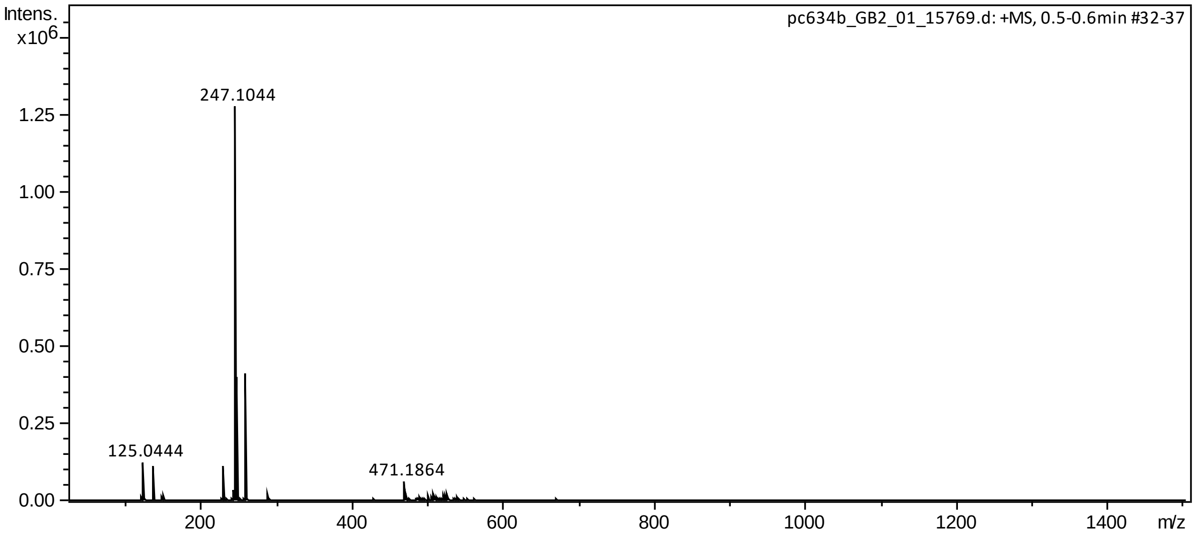

Figure S26. Compound **3d**, HRMS (ESI+) calc for $[C_{14}H_{12}N_2F_2+H]^+$: 247.1041 found 247.1044 $[M+H]^+$.

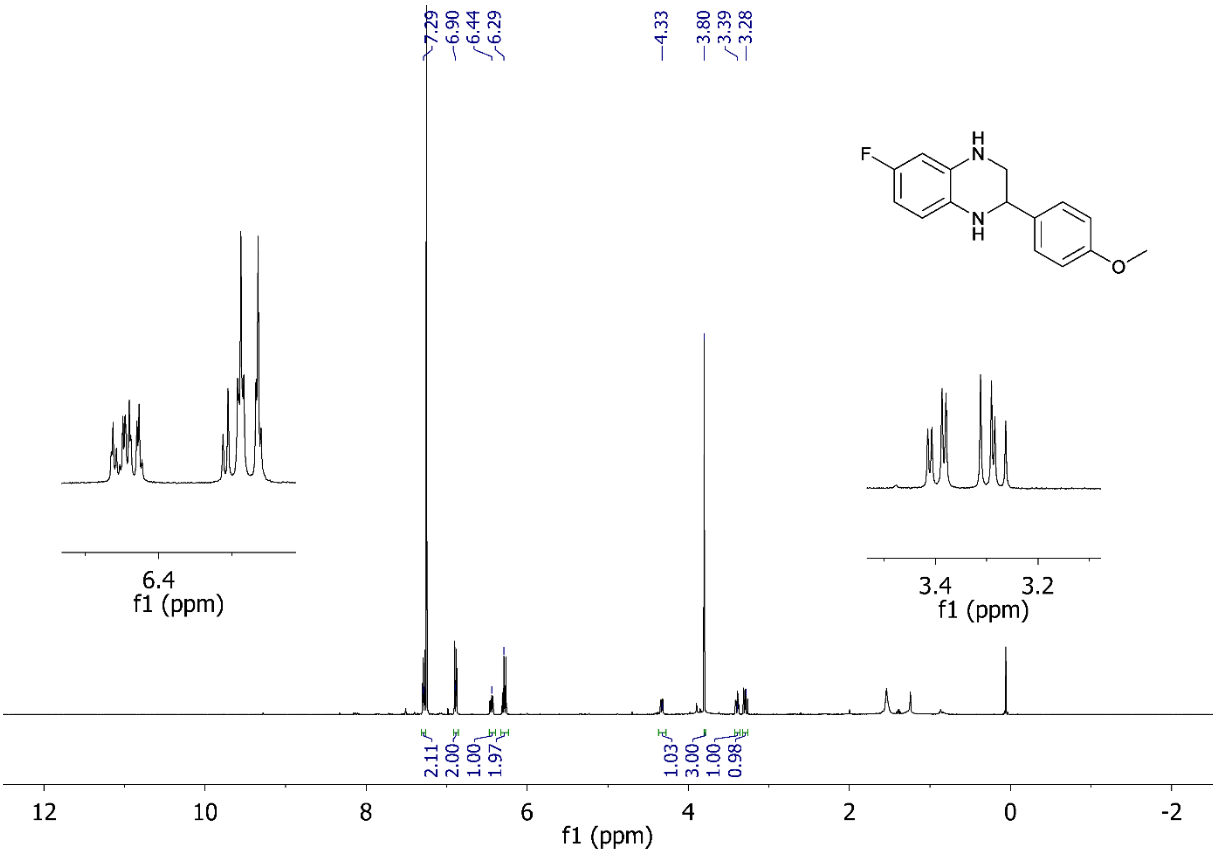

Figure S27. Compound **3e**, ^{1}H NMR (400 MHz, $CDCl_3$).

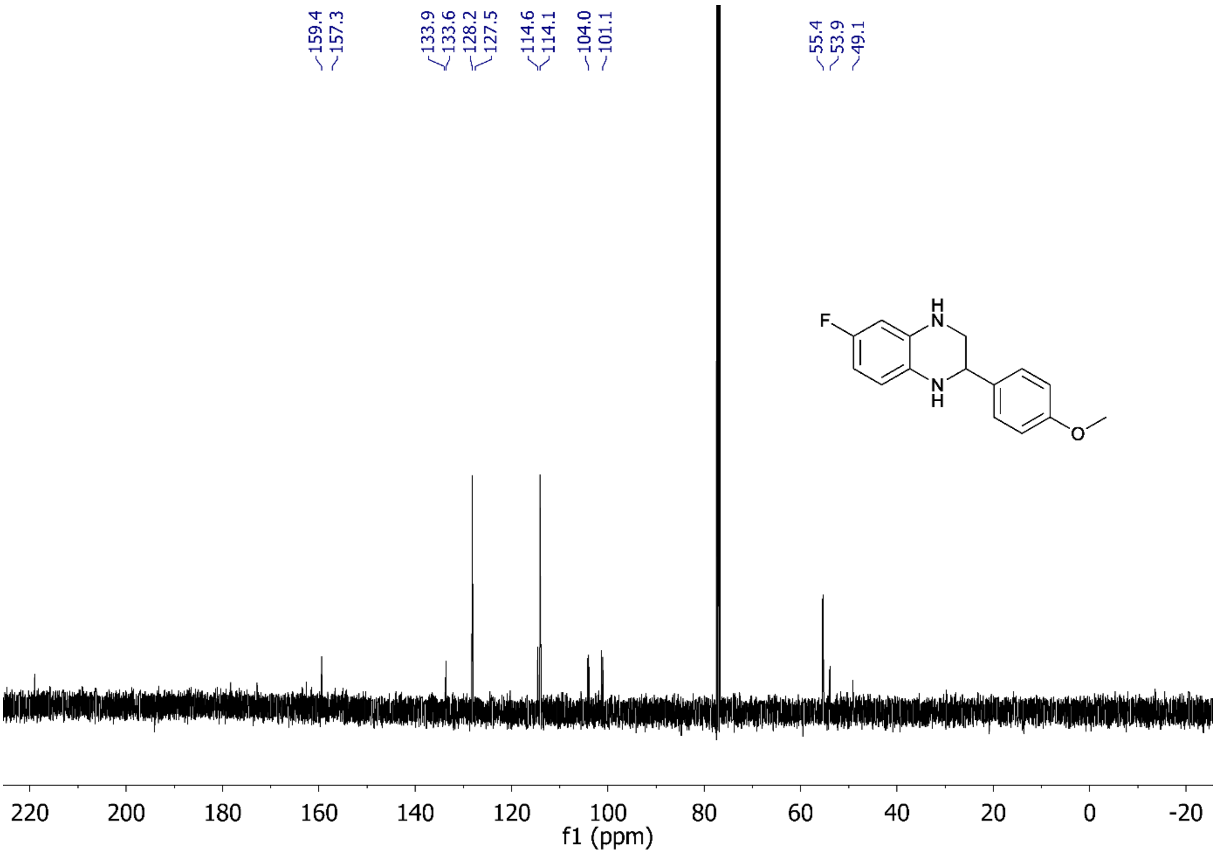

Figure S28. Compound **3e**, ^{13}C NMR (101 MHz, $CDCl_3$).

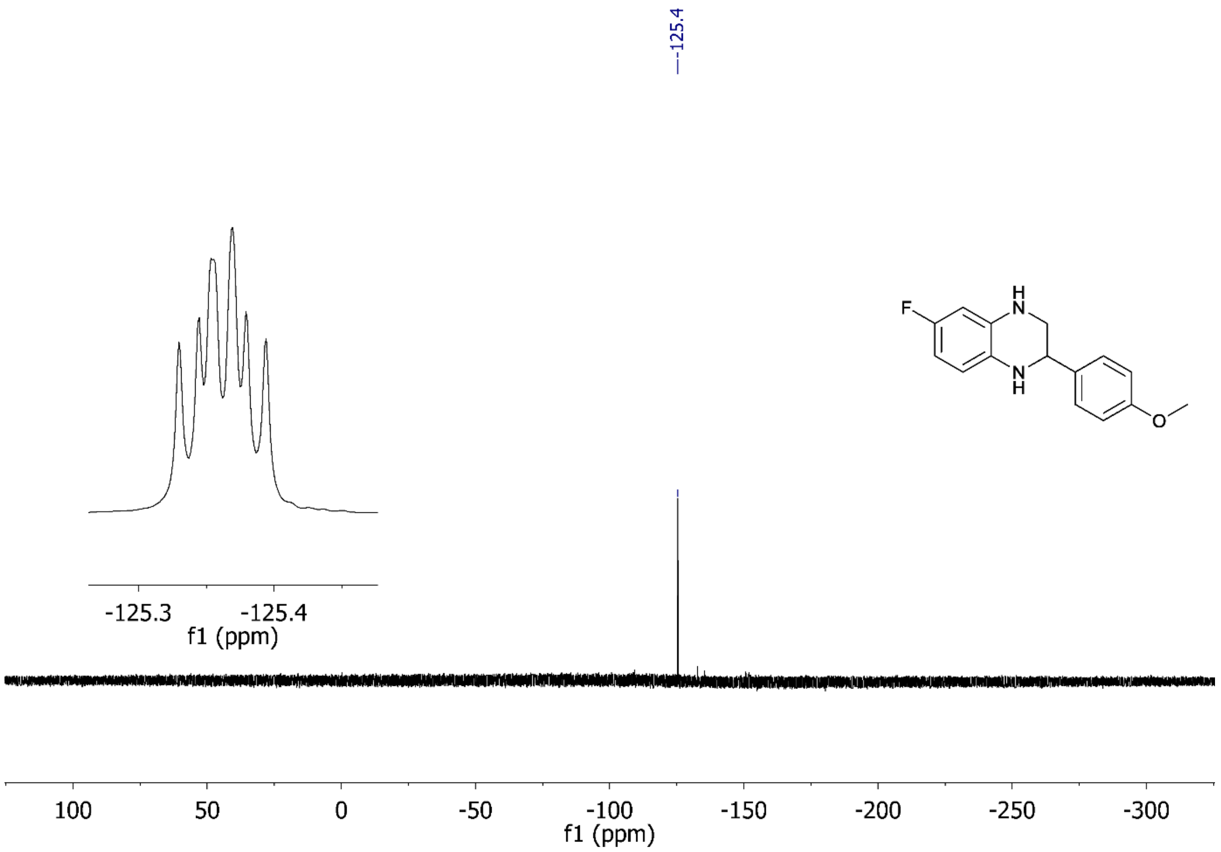

Figure S29. Compound **3e**, ^{19}F NMR (376.5 MHz, $CDCl_3$).

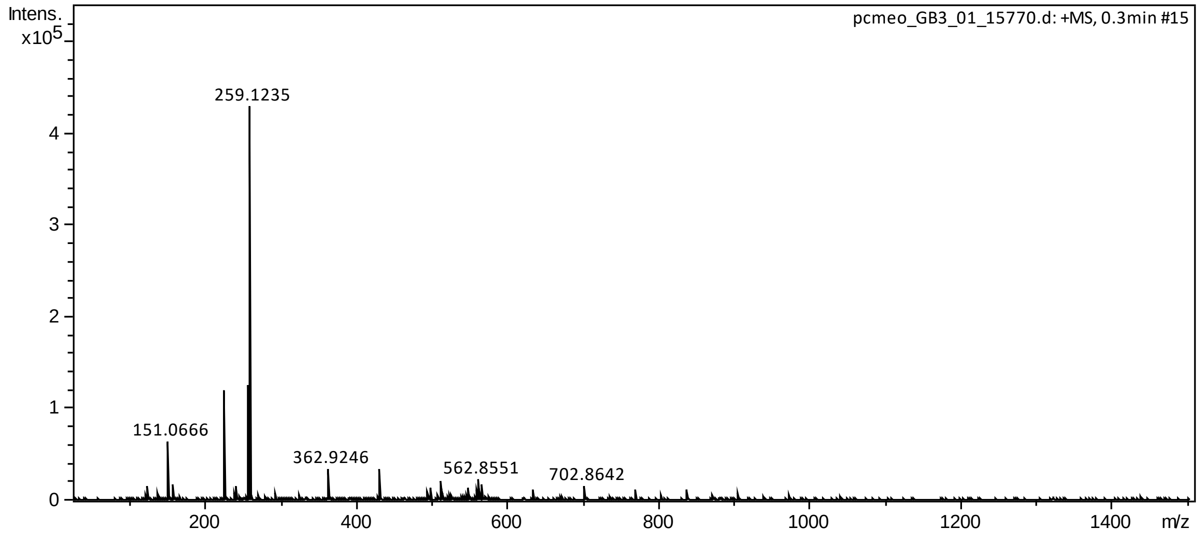

Figure S30. Compound **3e**, HRMS (ESI+) calc for $[C_{15}H_{15}N_2OF+H]^+$: 259.1241 found 259.1235 $[M+H]^+$.

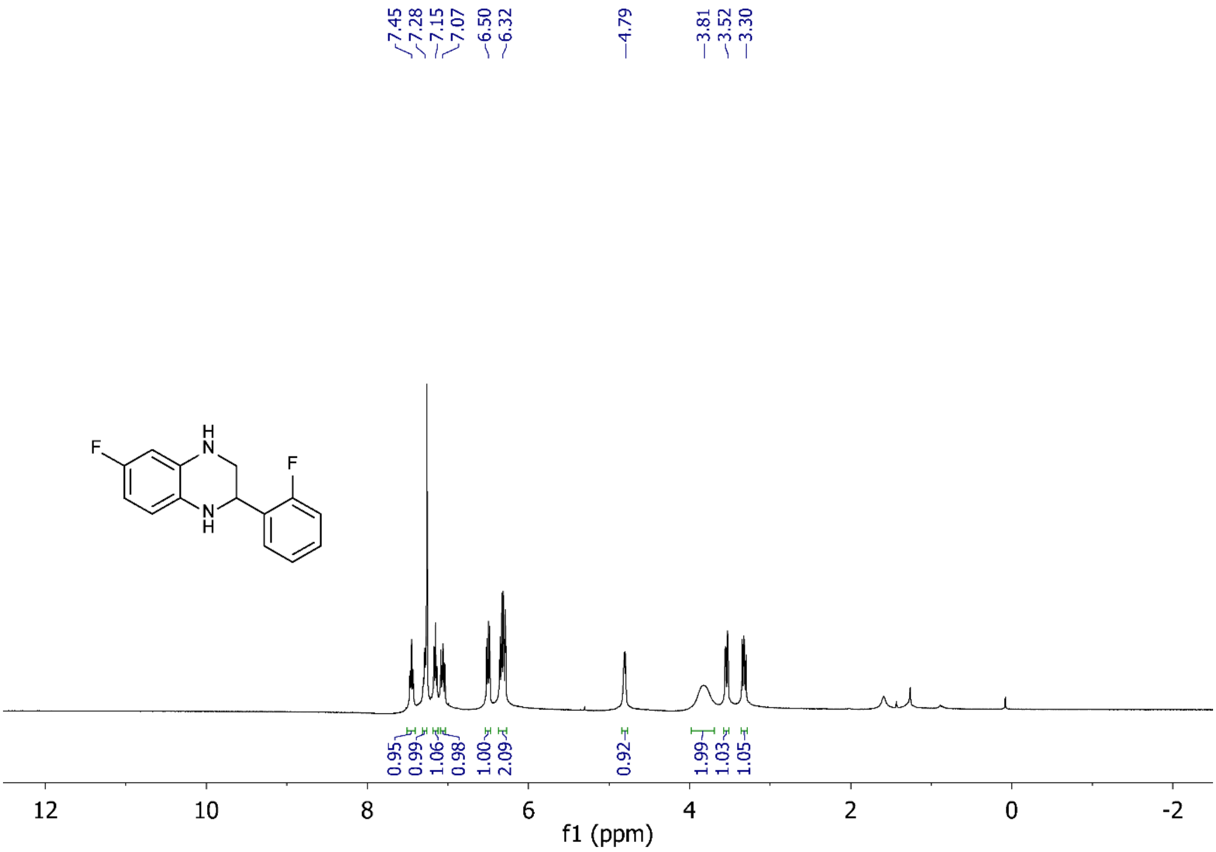

Figure S31. Compound **3f**, ^{1}H NMR (400 MHz, $CDCl_3$).

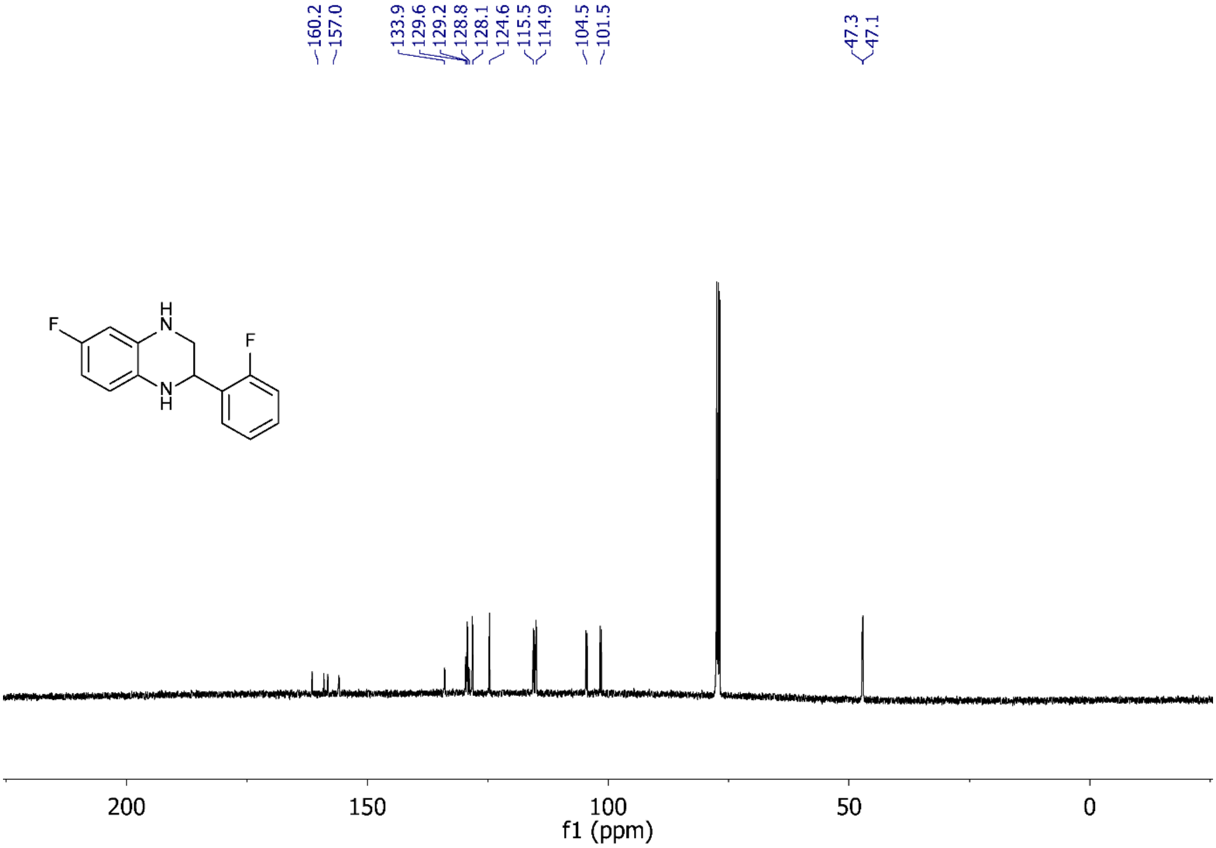

Figure S32. Compound **3f**, ^{13}C NMR (101 MHz, $CDCl_3$).

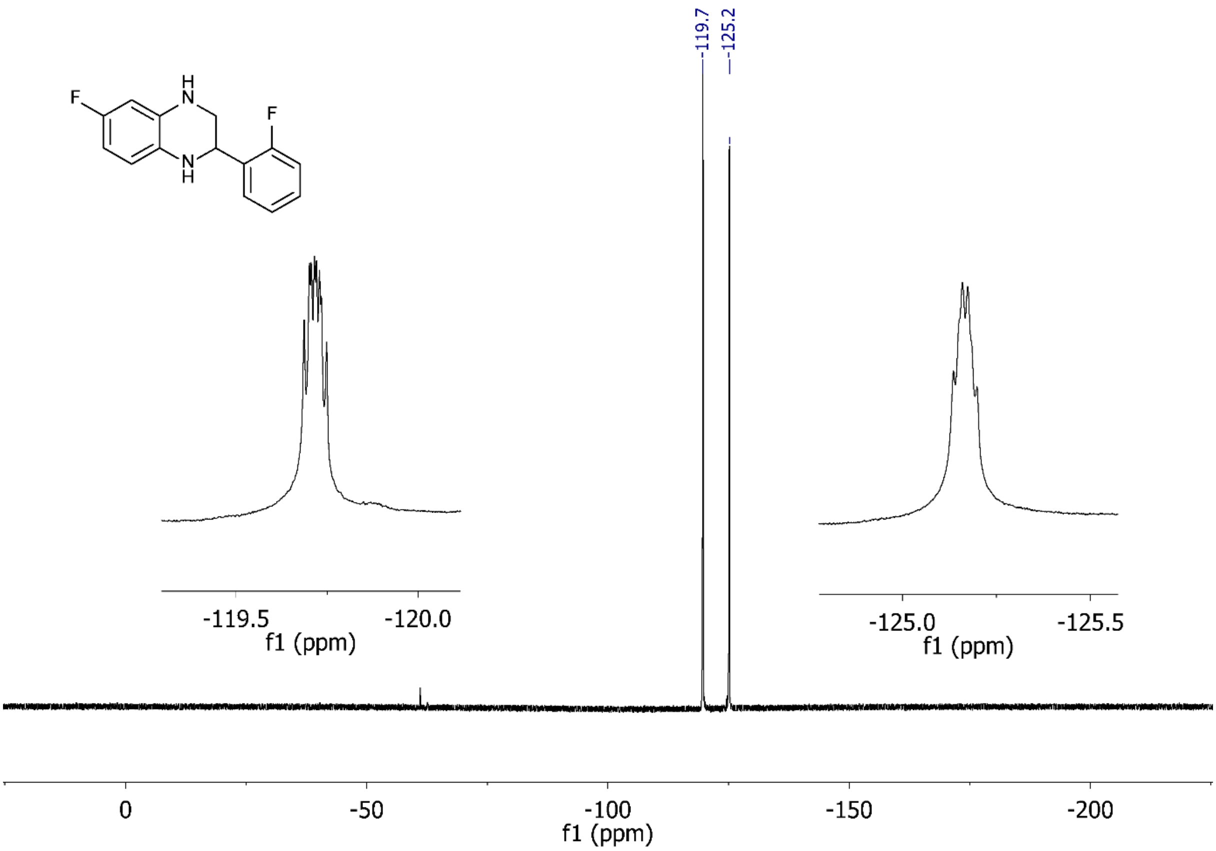

Figure S33. Compound **3f**, ^{19}F NMR (376.5 MHz, $CDCl_3$).

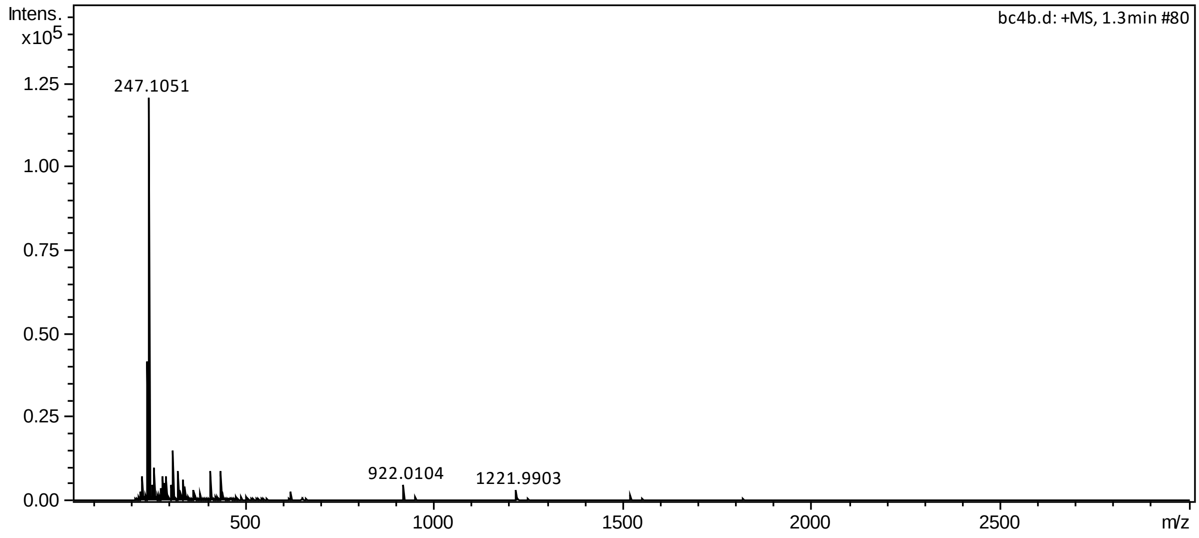

Figure S34. Compound **3f**, HRMS (APCI+) calc for $[C_{14}H_{12}F_2N_2+H]^+$: 247.1041 found 247.1051 $[M+H]^+$.

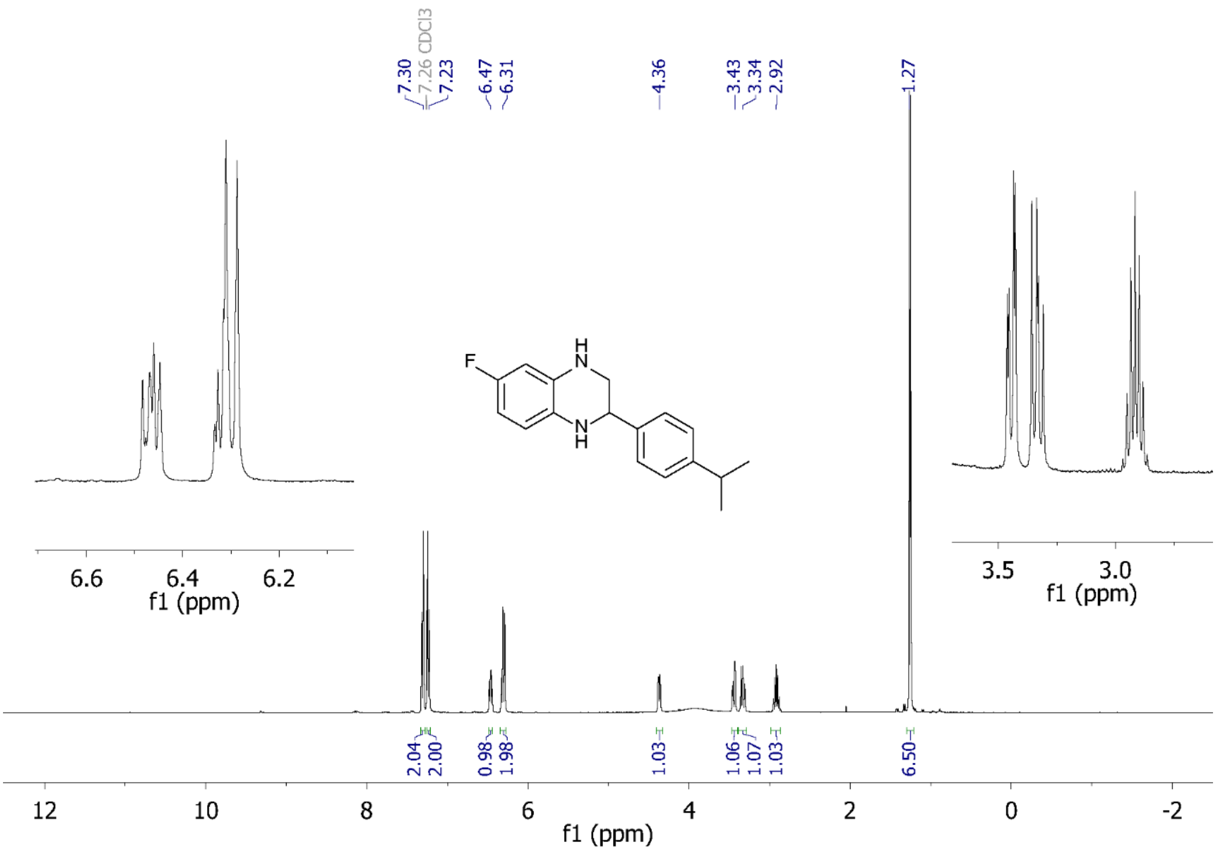

Figure S35. Compound **3g**, ^{1}H NMR (400 MHz, $CDCl_3$).

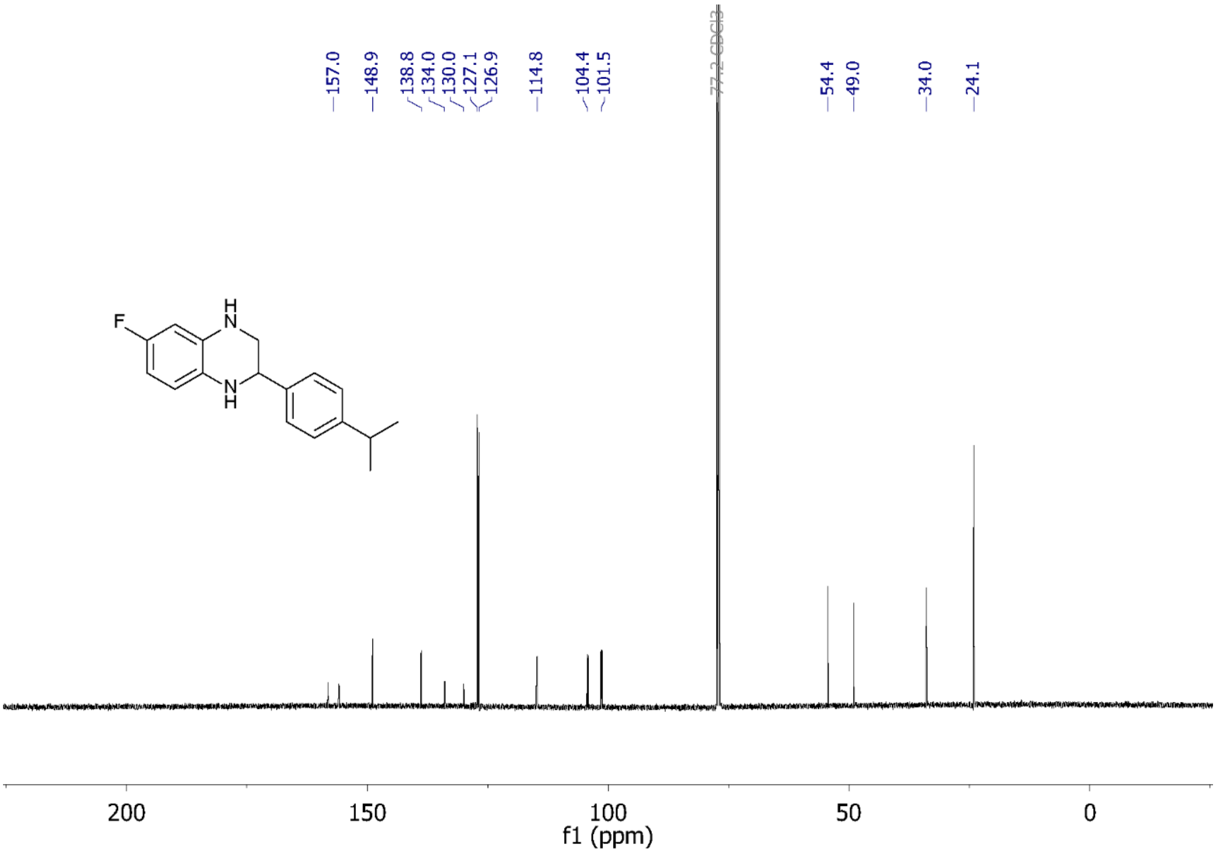

Figure S36. Compound **3g,** ^{13}C NMR (101 MHz, $CDCl_3$).

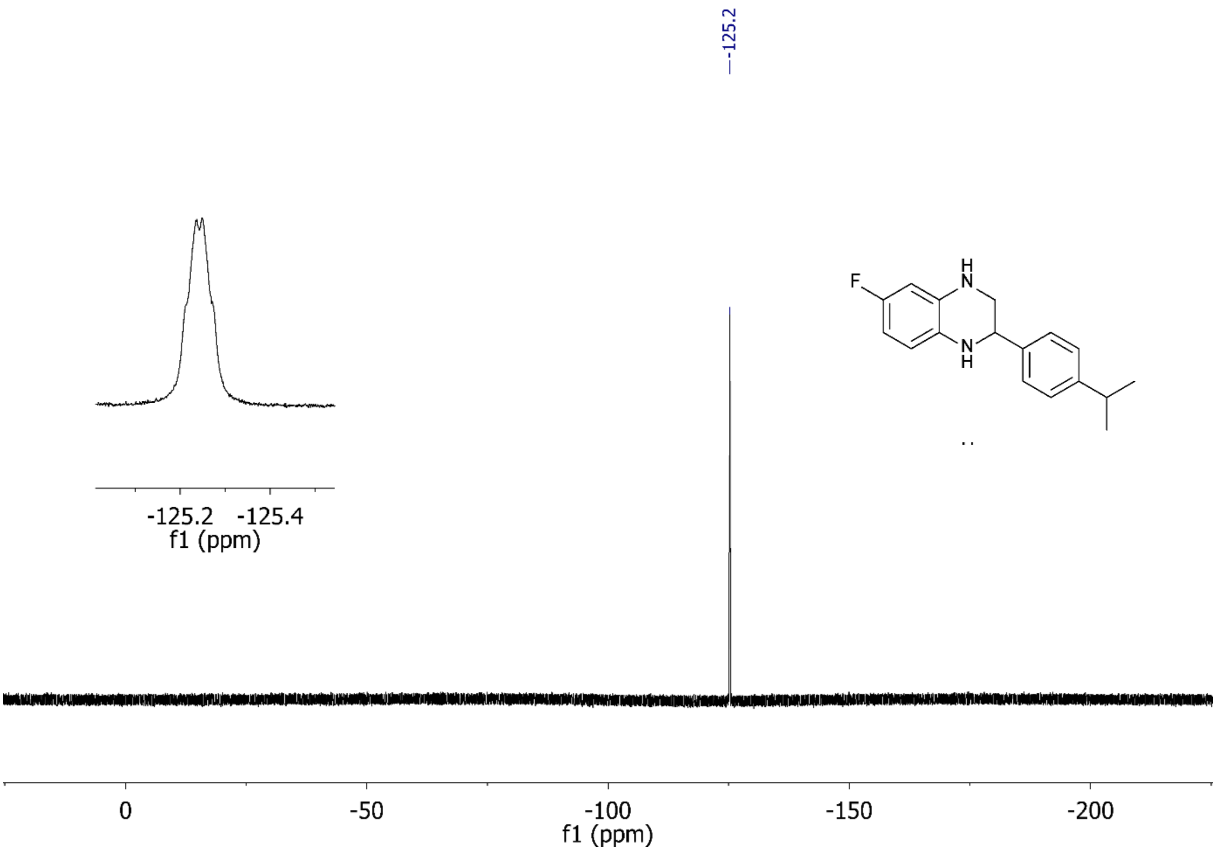

Figure S37. Compound **3g**, ^{19}F NMR (376.5 MHz, $CDCl_3$).

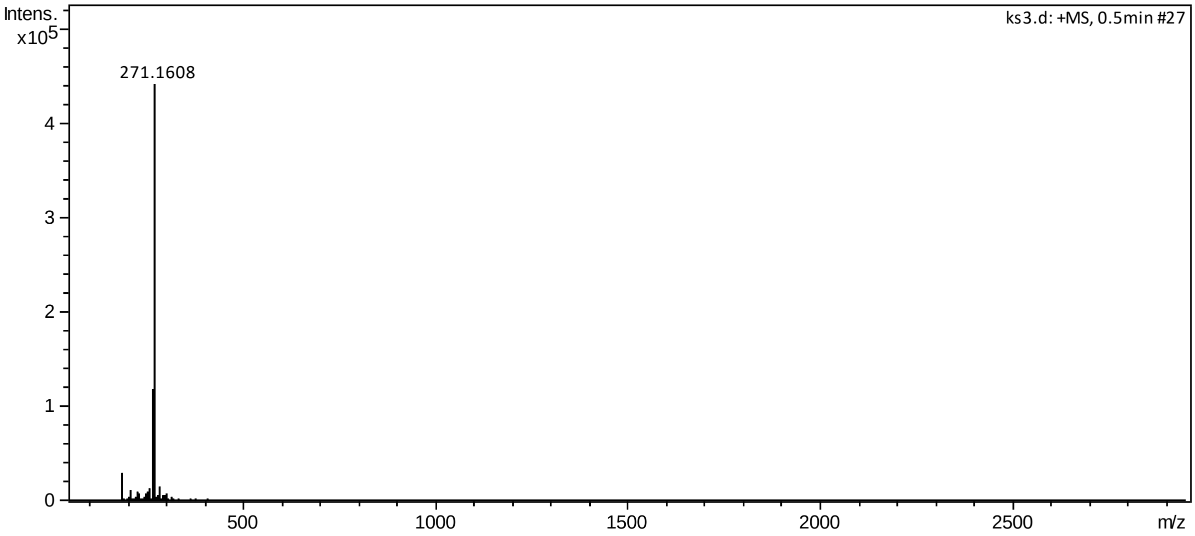

Figure S38. Compound **3g**, HRMS (APCI+) calc for $[C_{17}H_{20}N_2F+H]^+$: 271.1605 found 271.1608 $[M+H]^+$.

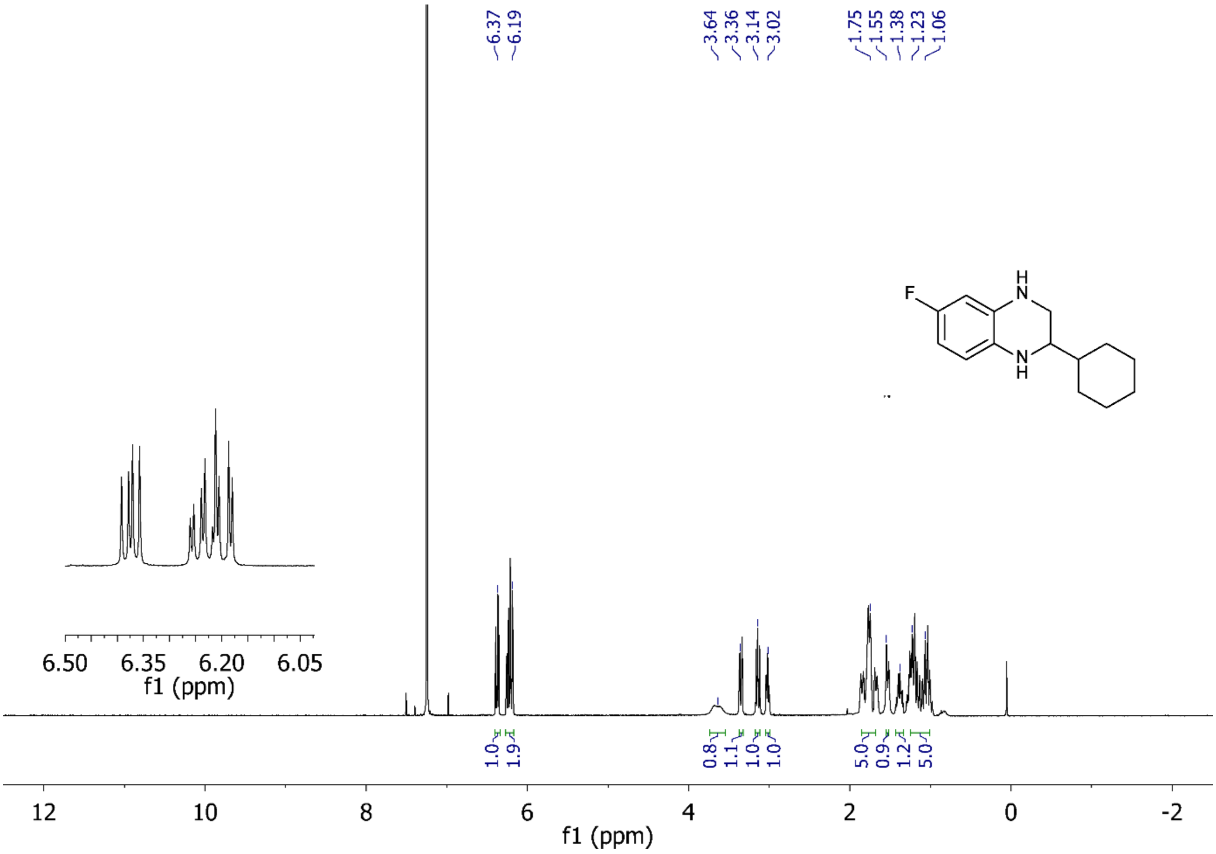

Figure S39. Compound **3h**, ^{1}H NMR (400 MHz, $CDCl_3$).

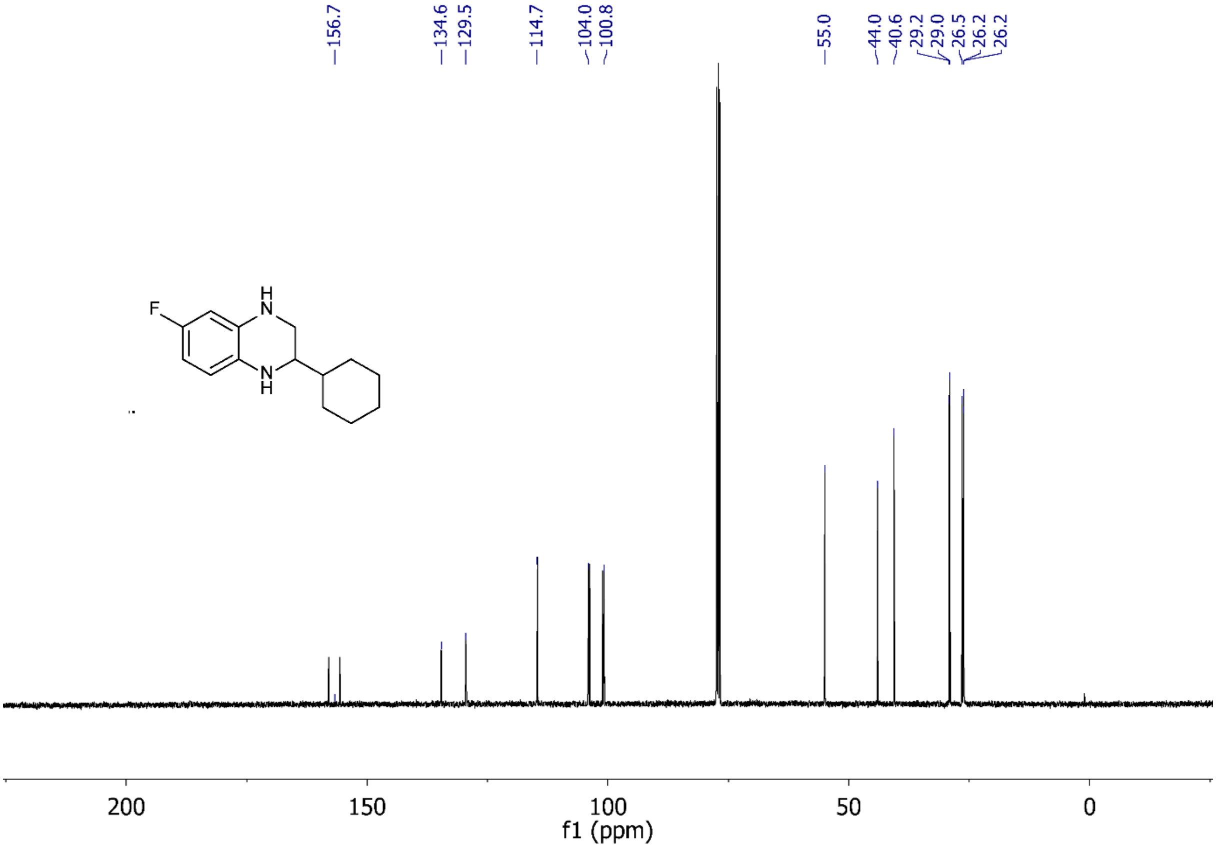

Figure S40. Compound **3h**, ^{13}C NMR (101 MHz, $CDCl_3$).

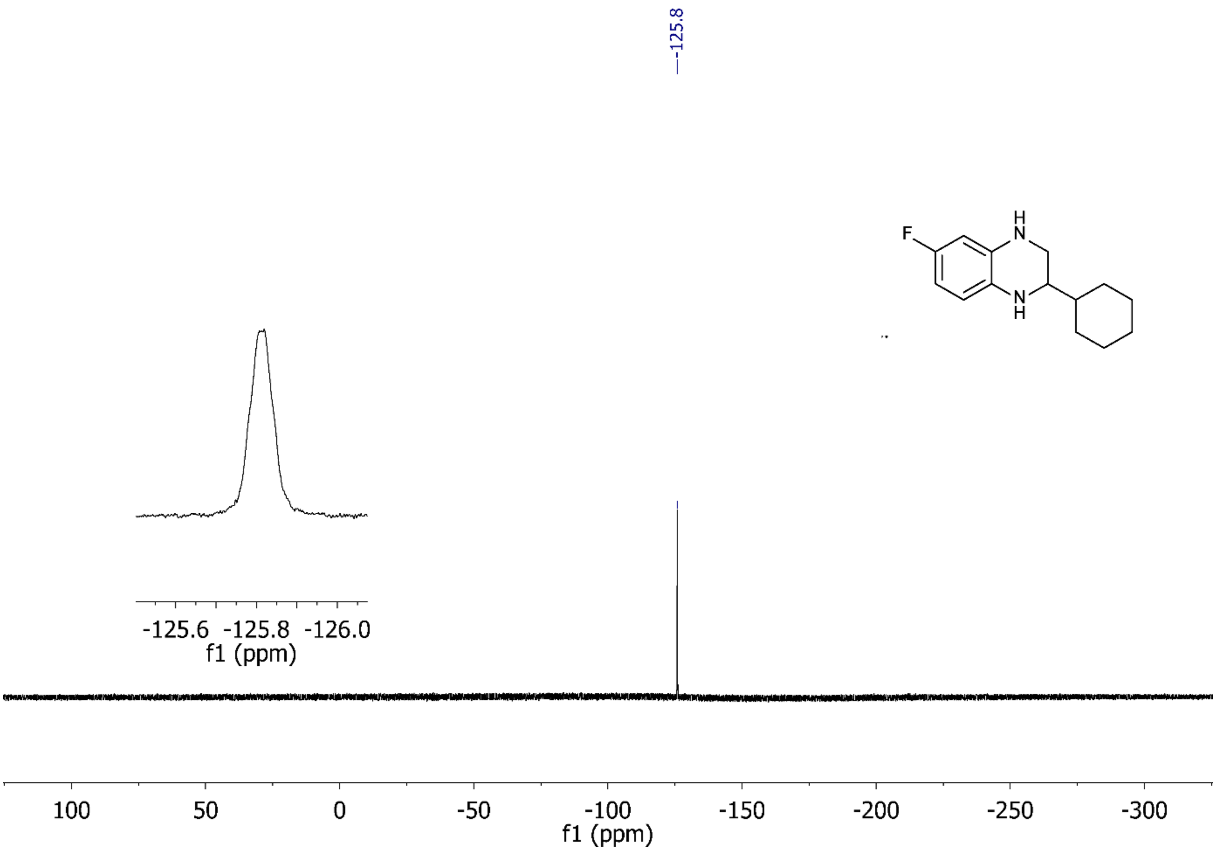

Figure S41. Compound **3h**, ^{19}F NMR (376.5 MHz, $CDCl_3$).

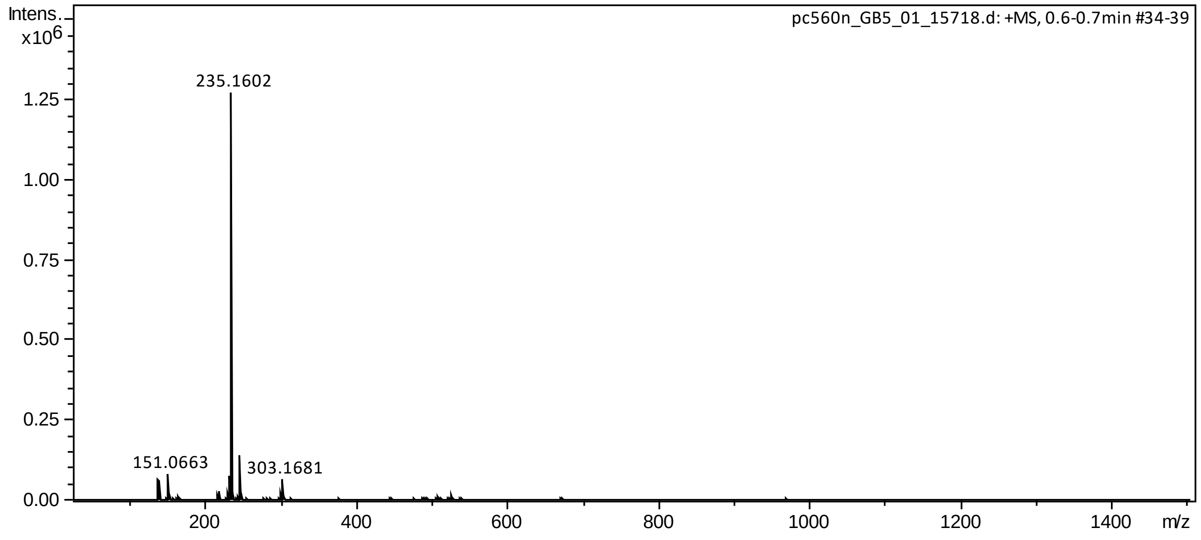

Figure S42. Compound **3h**, HRMS (ESI+) calc for $[C_{14}H_{19}FN_2+H]^+$: 235.1605 found 235.1602 $[M+H]^+$.

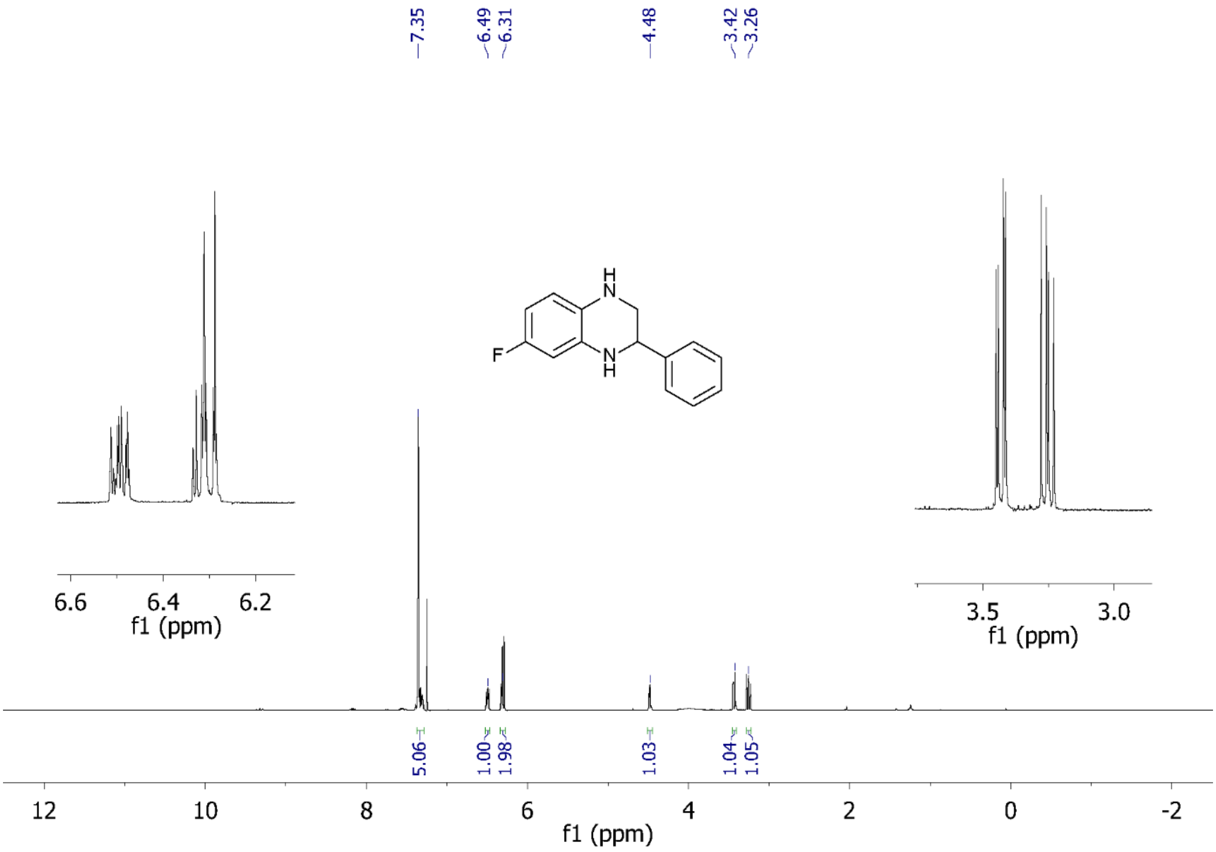

Figure S43. Compound **4a**, ^{1}H NMR (400 MHz, $CDCl_3$).

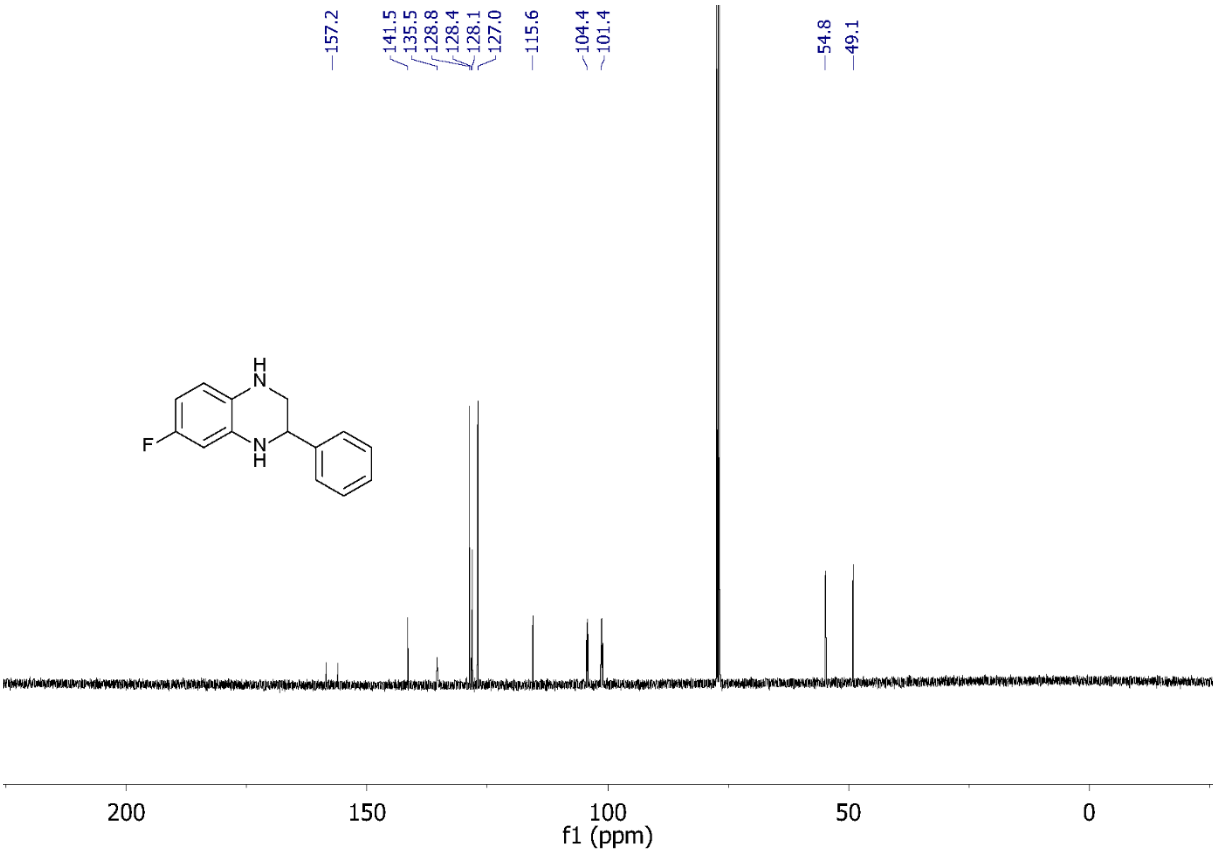

Figure S44. Compound **4a**, ^{13}C NMR (101 MHz, $CDCl_3$).

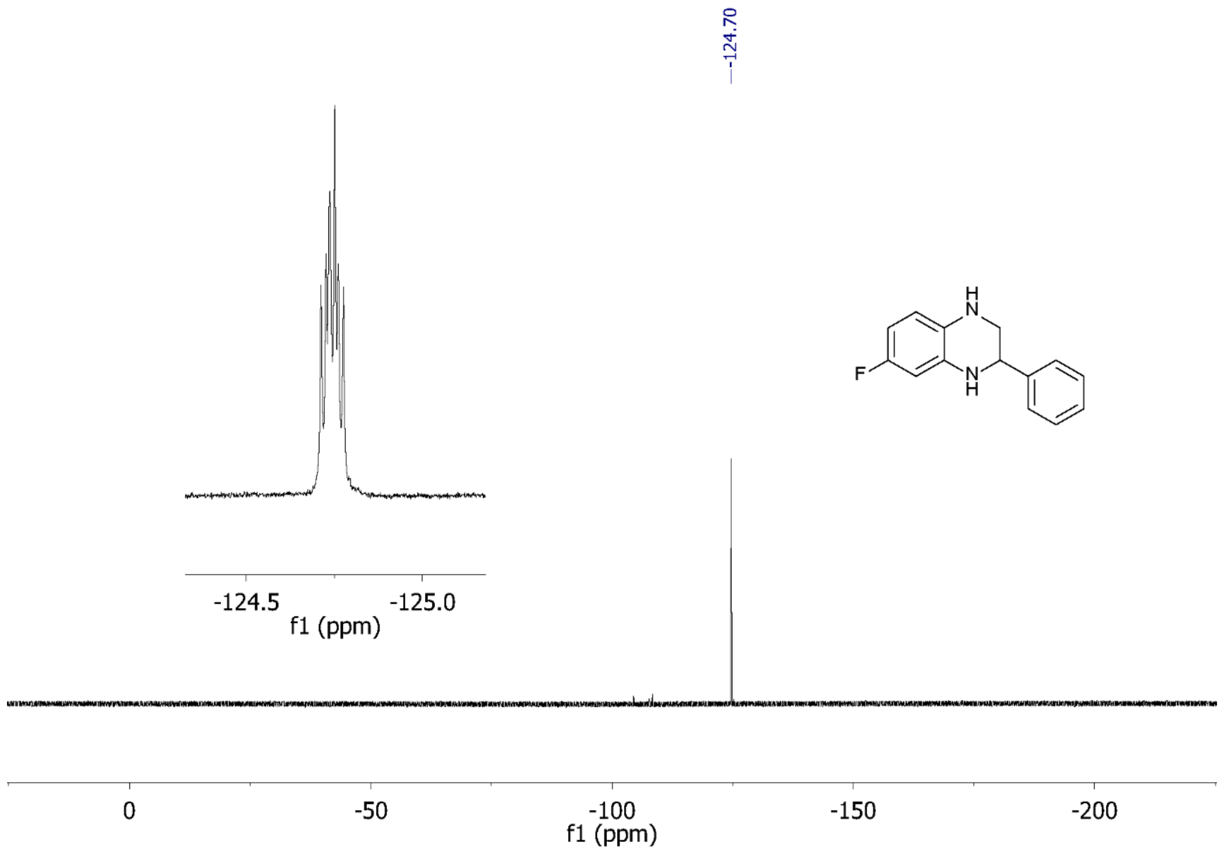

Figure S45. Compound **4a**, ^{19}F NMR (376.5 MHz, $CDCl_3$).

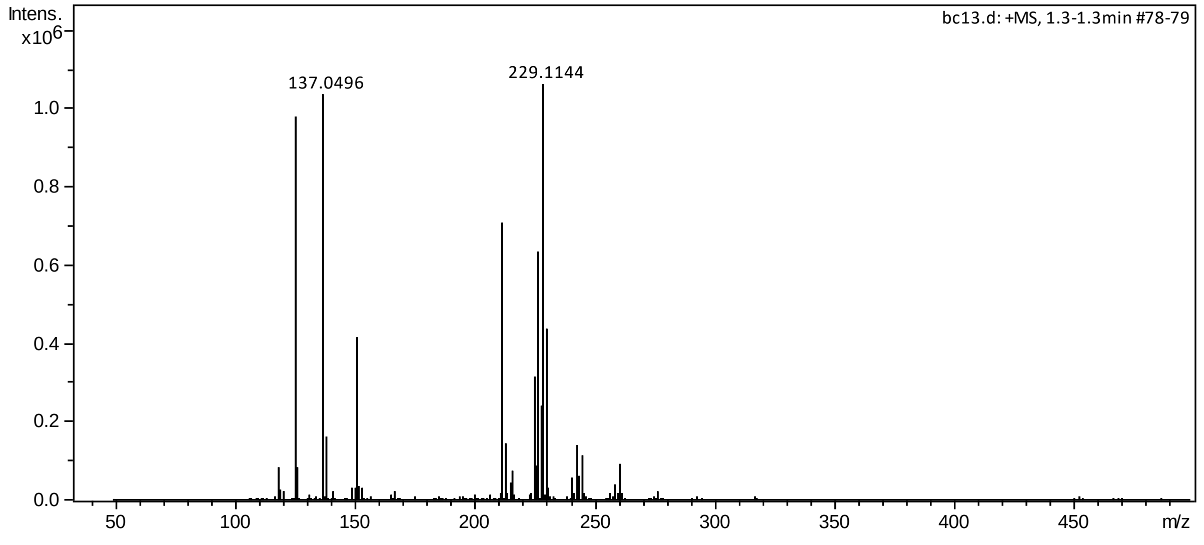

Figure S46. Compound **4a**, HRMS (APCI+) calc for $[C_{14}H_{13}FN_2+H]^+$: 229.1136 found 229.1144 $[M+H]^+$.

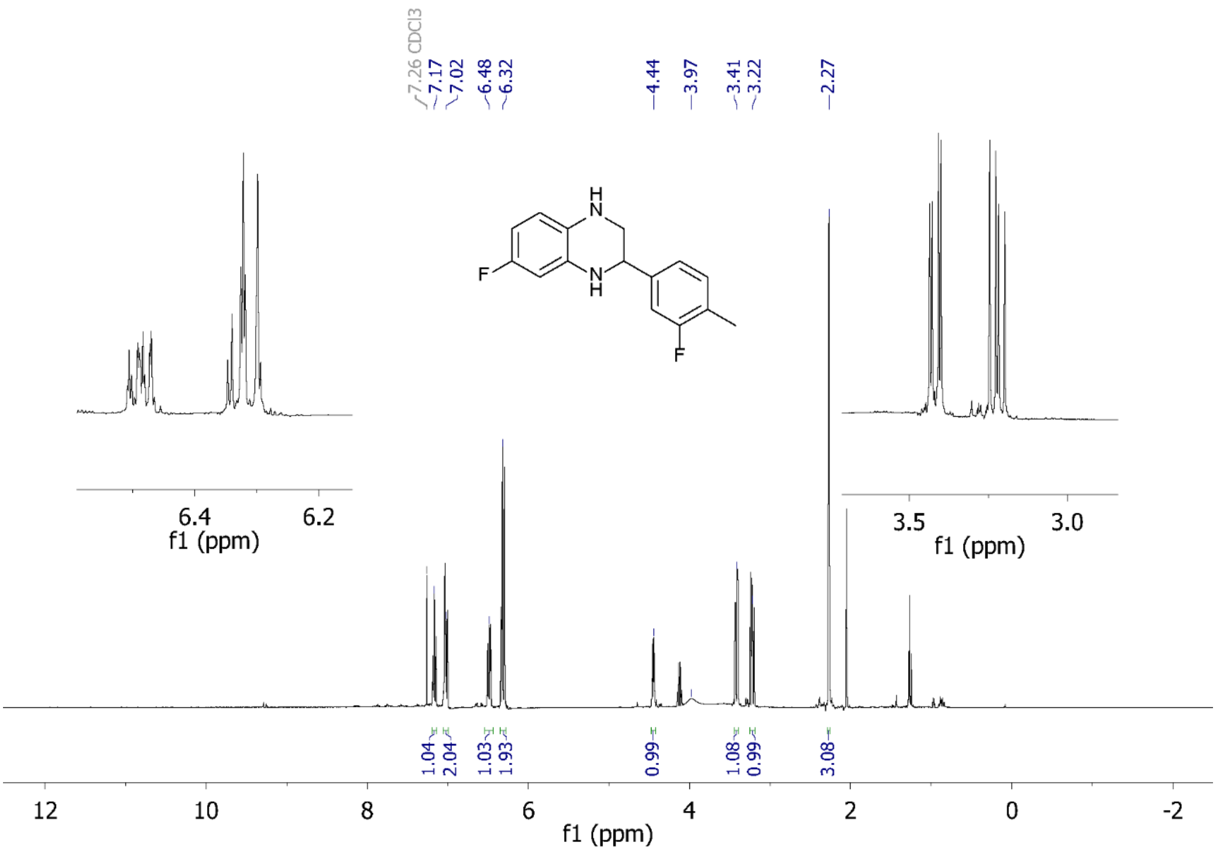

Figure S47. Compound **4b**, ^{1}H NMR (400 MHz, $CDCl_3$).

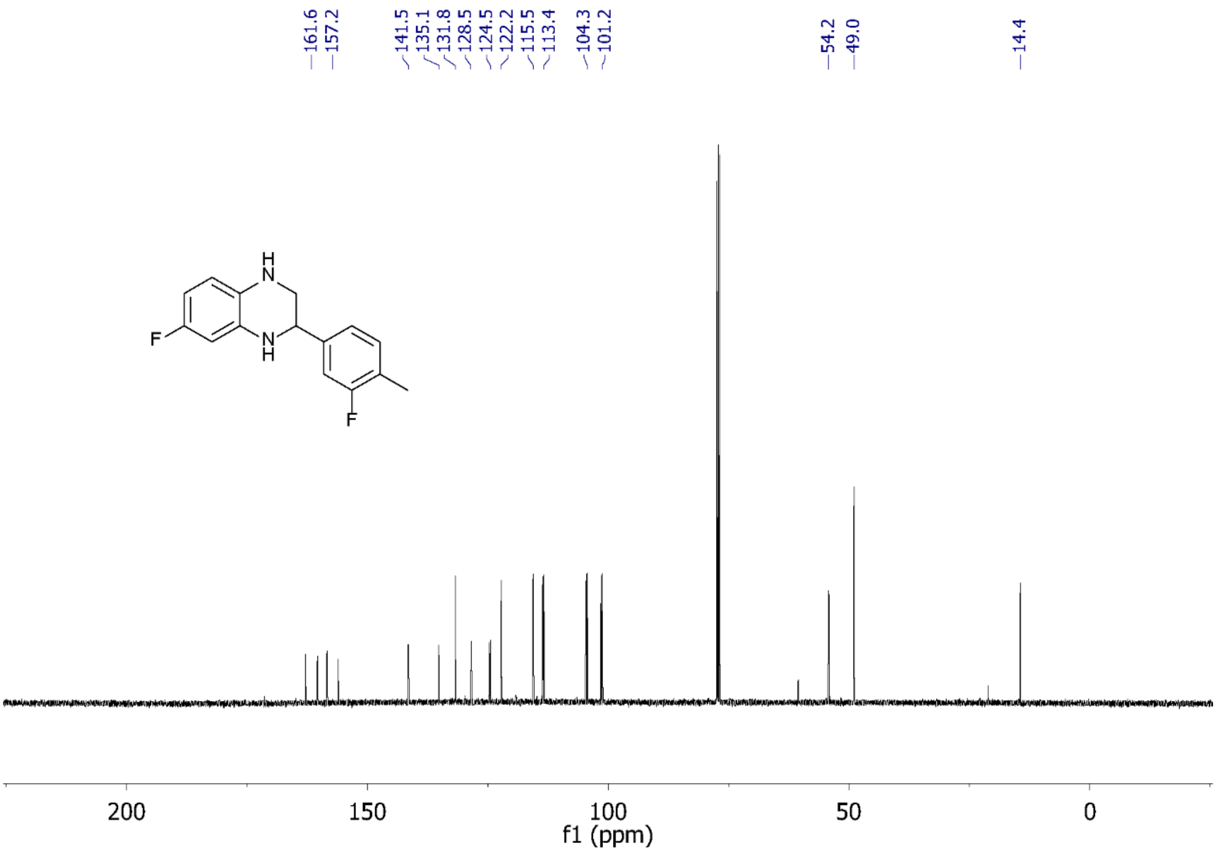

Figure S48. Compound **4b**, ^{13}C NMR (101 MHz, $CDCl_3$).

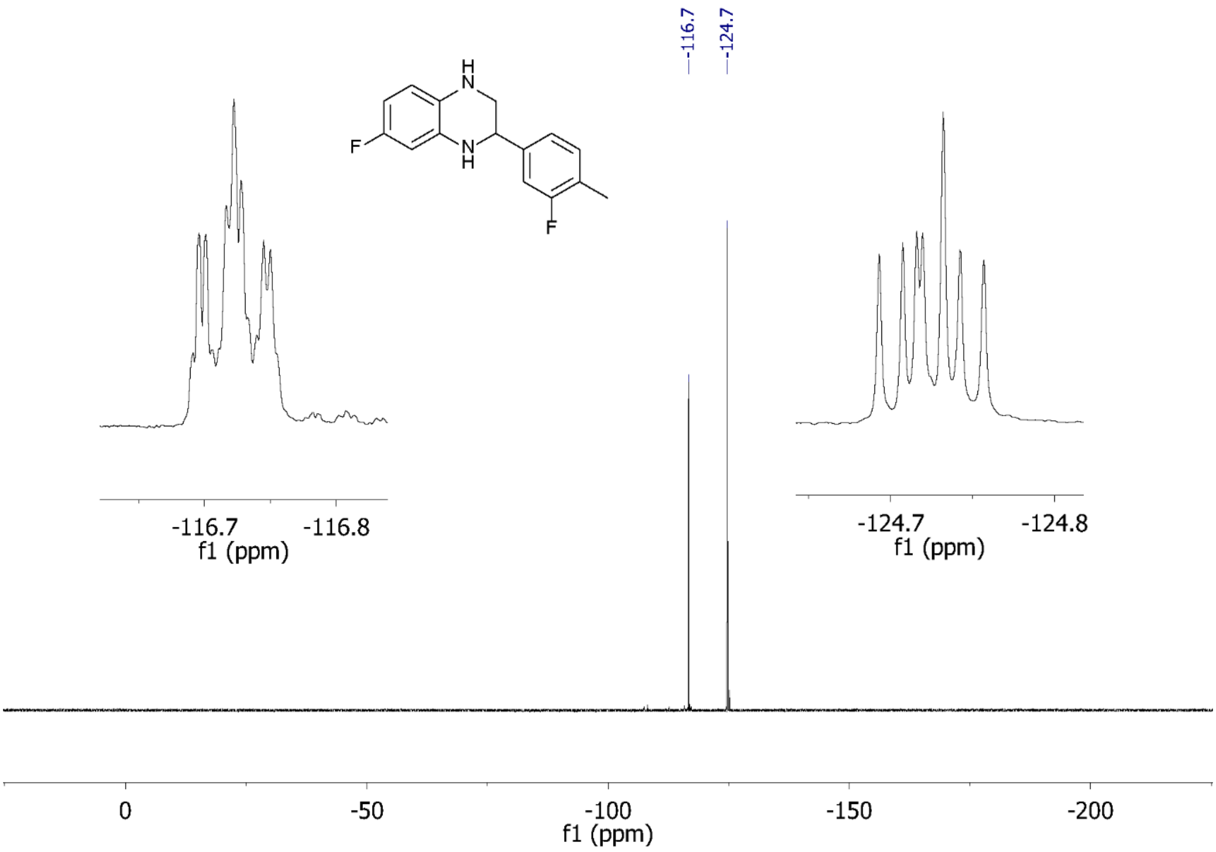

Figure S49. Compound **4b**, ^{19}F NMR (376.5 MHz, $CDCl_3$).

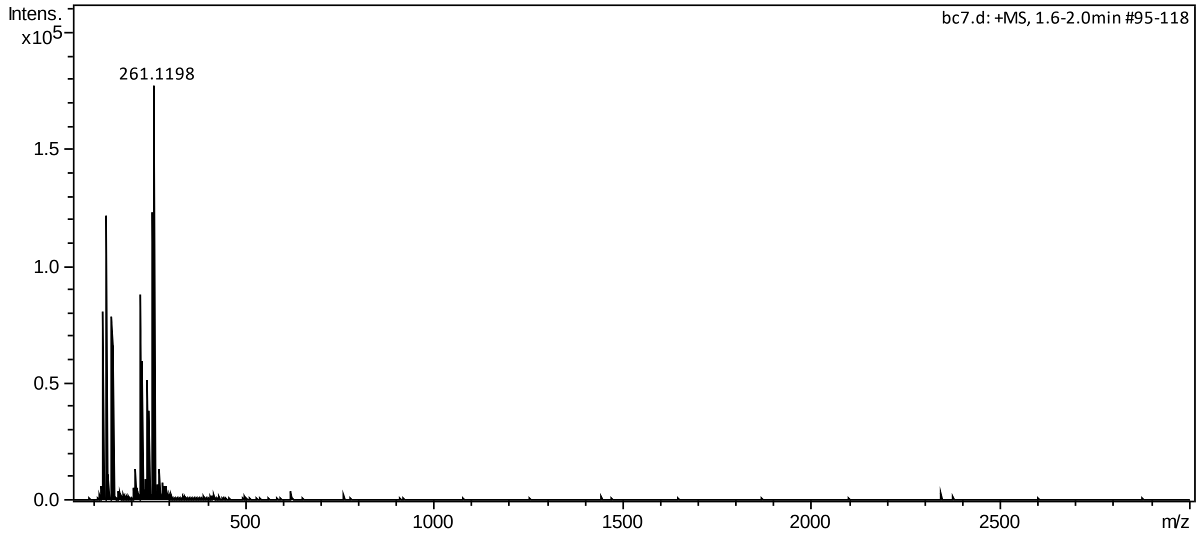

Figure S50. Compound **4b**, HRMS (APCI+) calc for $[C_{15}H_{14}N_2F_2+H]^+$: 261.1198 found 261.1198 $[M+H]^+$.

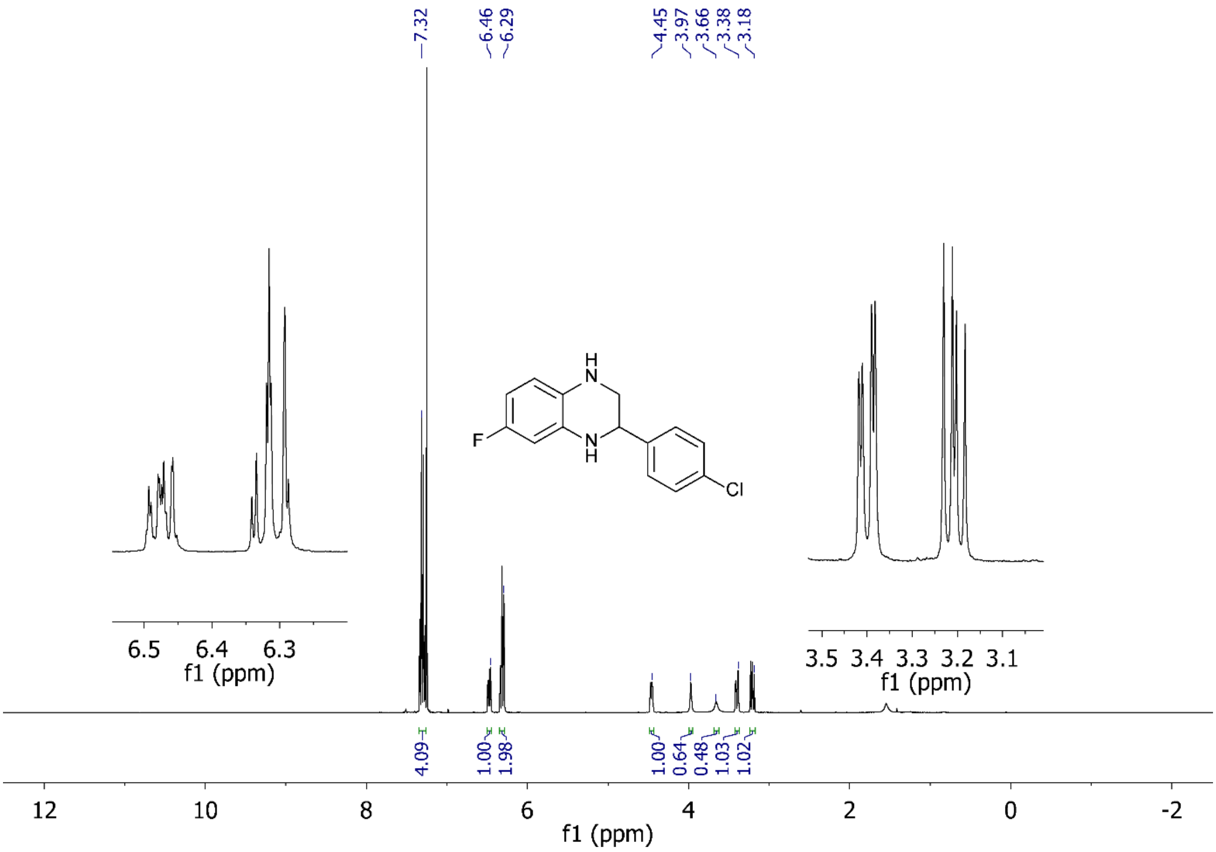

Figure S51. Compound **4c**, ^{1}H NMR (400 MHz, $CDCl_3$).

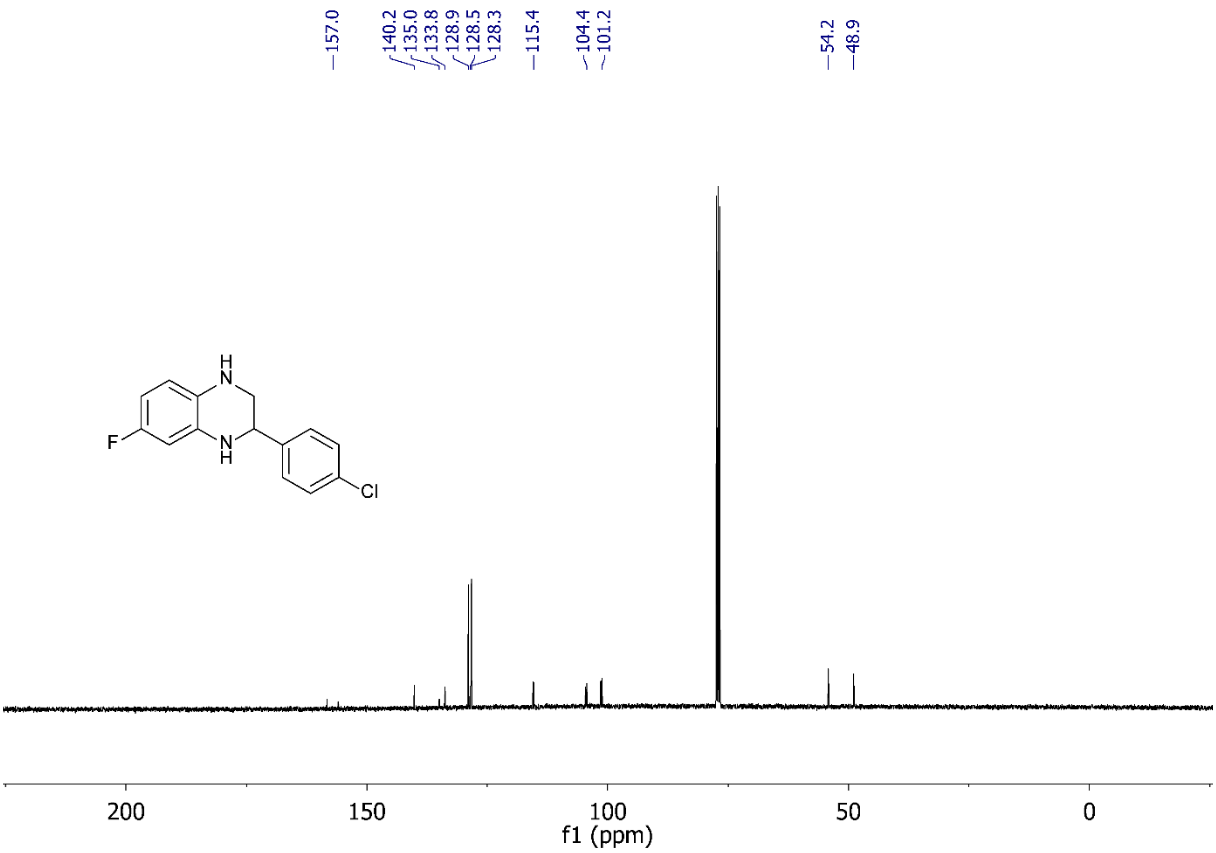

Figure S52. Compound **4c**, ^{13}C NMR (101 MHz, $CDCl_3$).

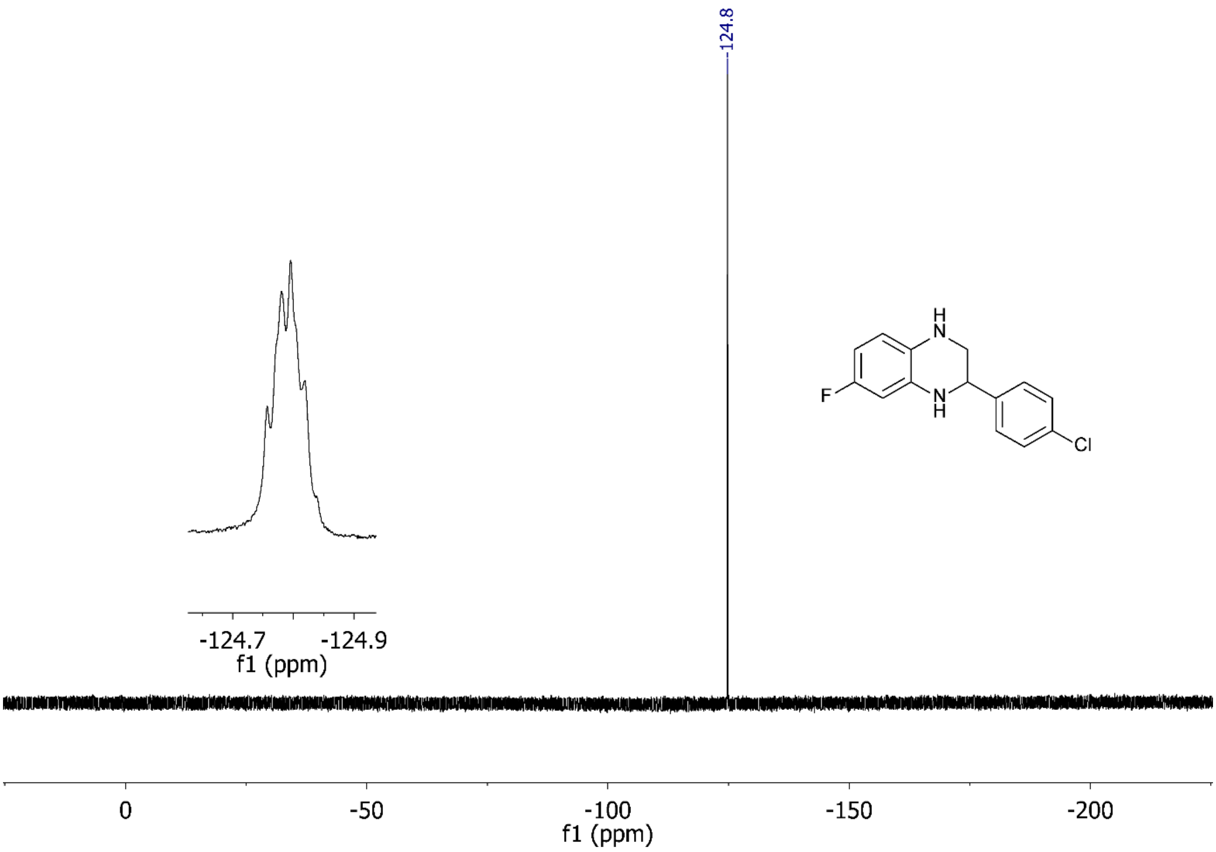

Figure S53. Compound **4c**, ^{19}F NMR (376.5 MHz, $CDCl_3$).

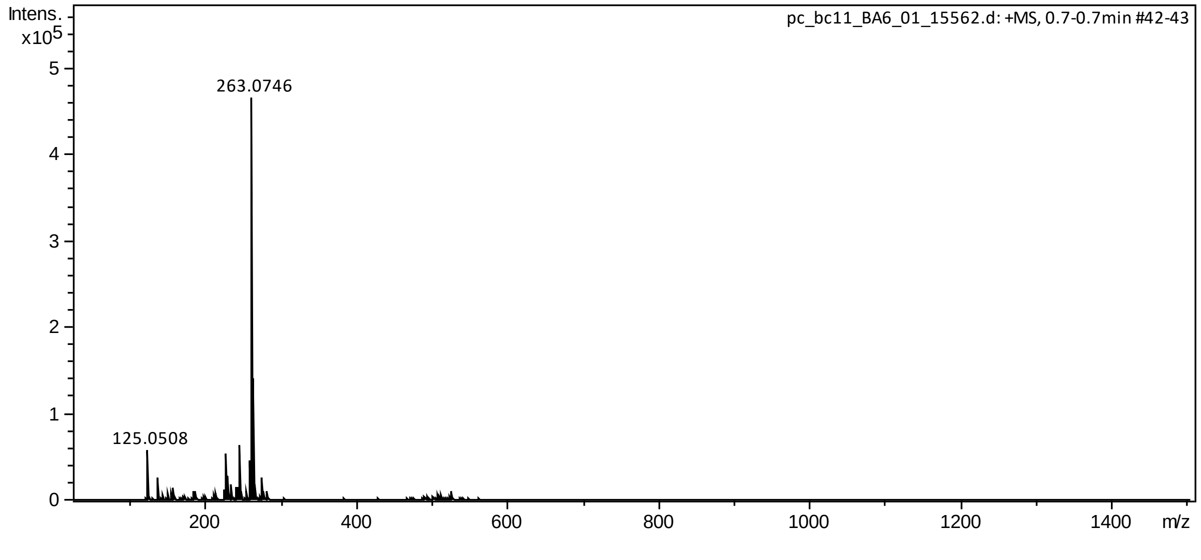

Figure S54. Compound **4c**, HRMS (ESI+) calc for $[C_{14}H_{12}FClN_2+H]^+$: calc 263.0746 found 263.0746 $[M+H]^+$

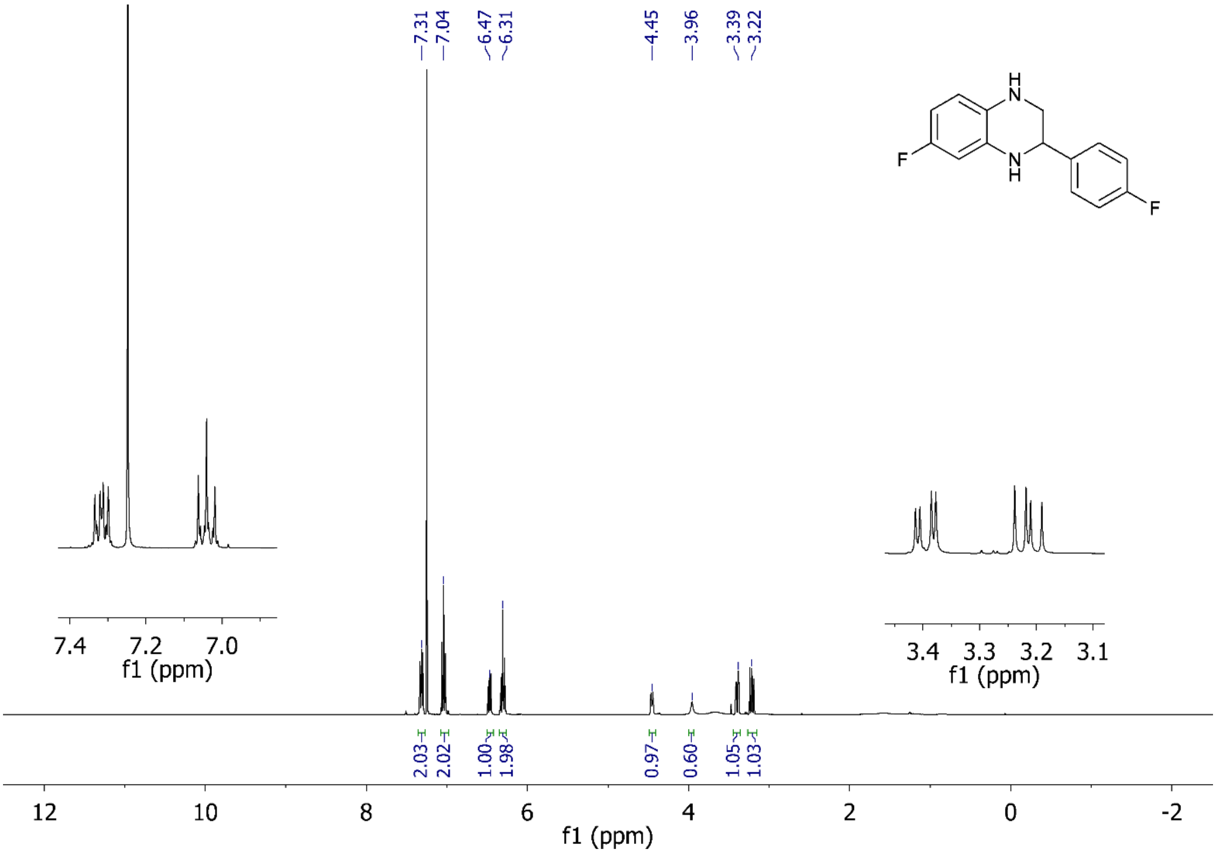

Figure S55. Compound **4d**, ^{1}H NMR (400 MHz, $CDCl_3$).

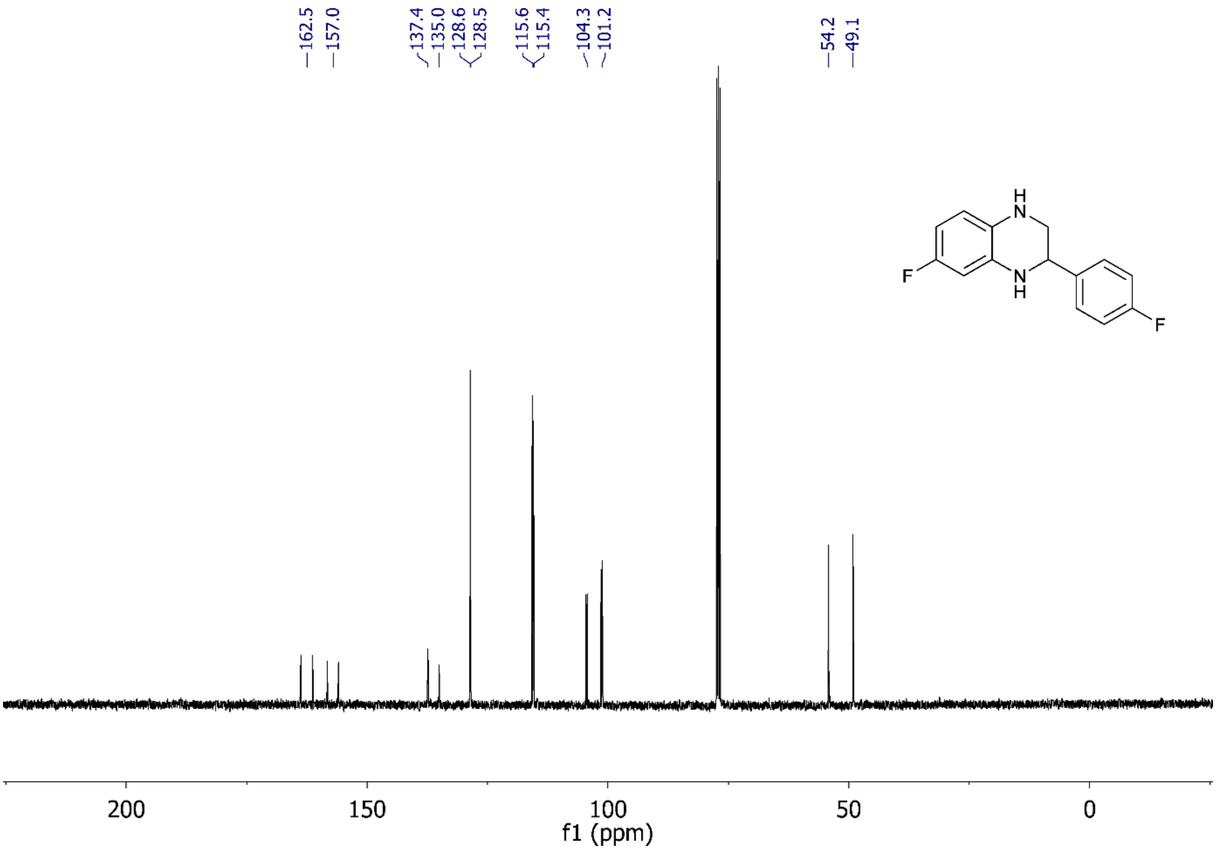

Figure S56. Compound **4d**, ^{13}C NMR (101 MHz, $CDCl_3$).

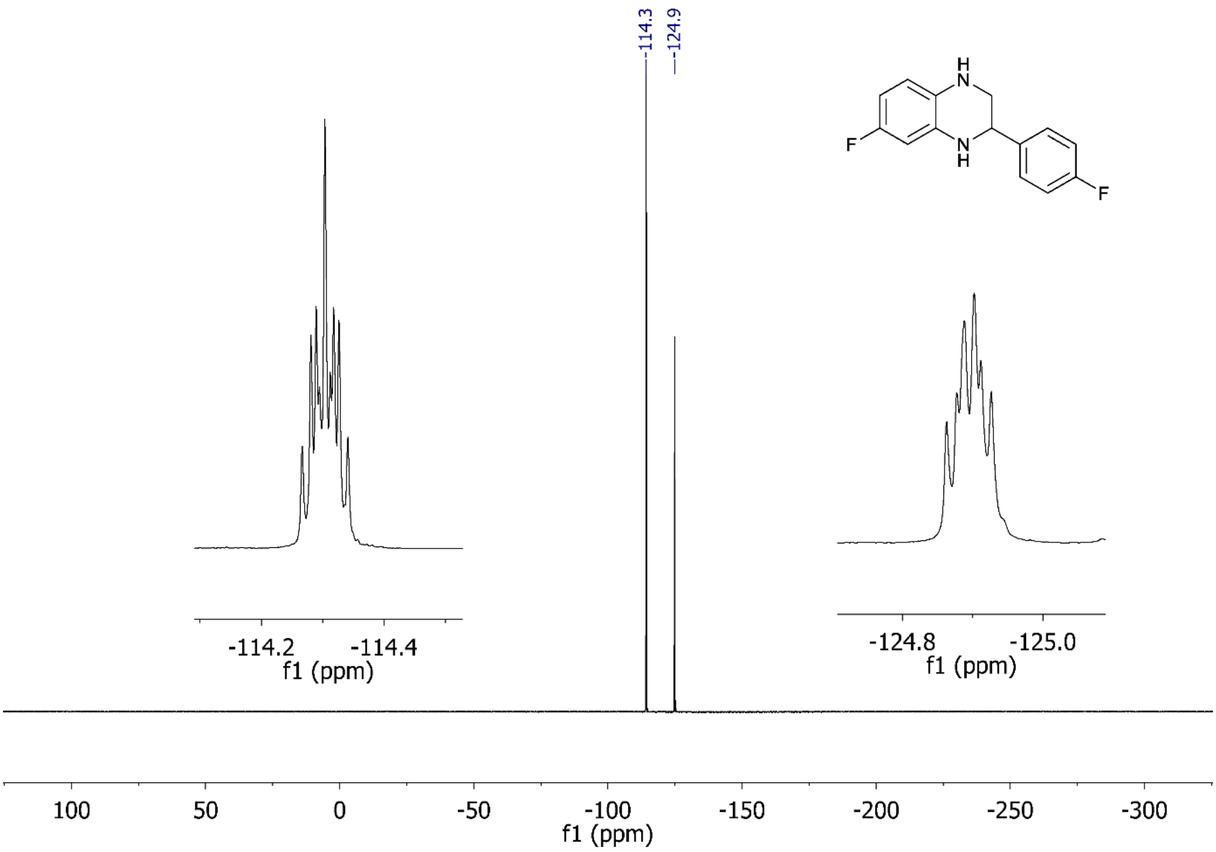

Figure S57. Compound **4d**, ^{19}F NMR (376.5 MHz, $CDCl_3$).

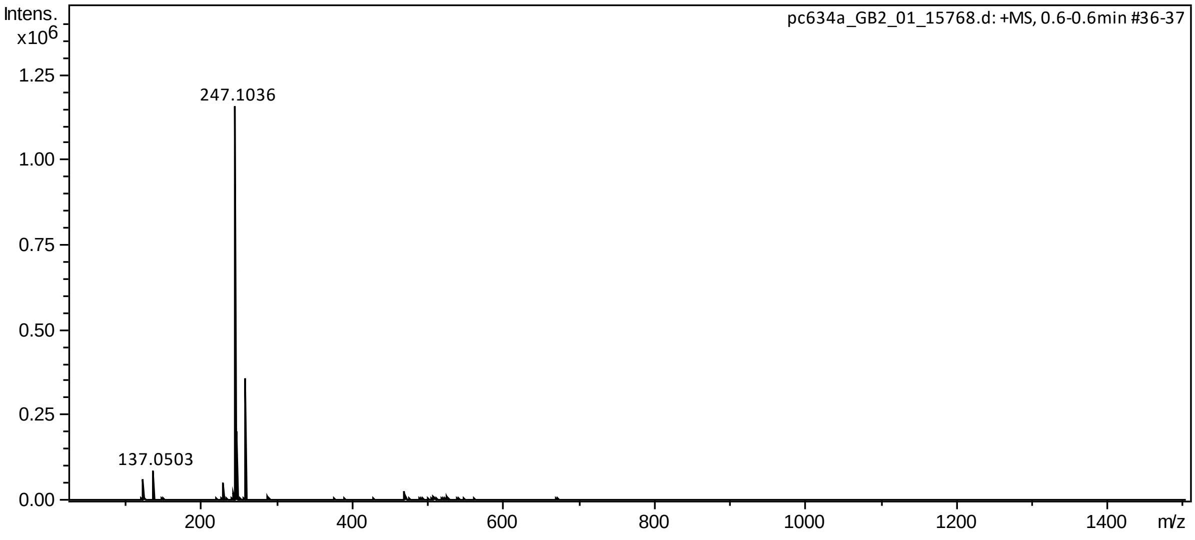

Figure S58. Compound **4d**, HRMS (ESI+) calc for $[C_{14}H_{12}N_2F_2+H]^+$: 247.1041 found 247.1036 $[M+H]^+$.

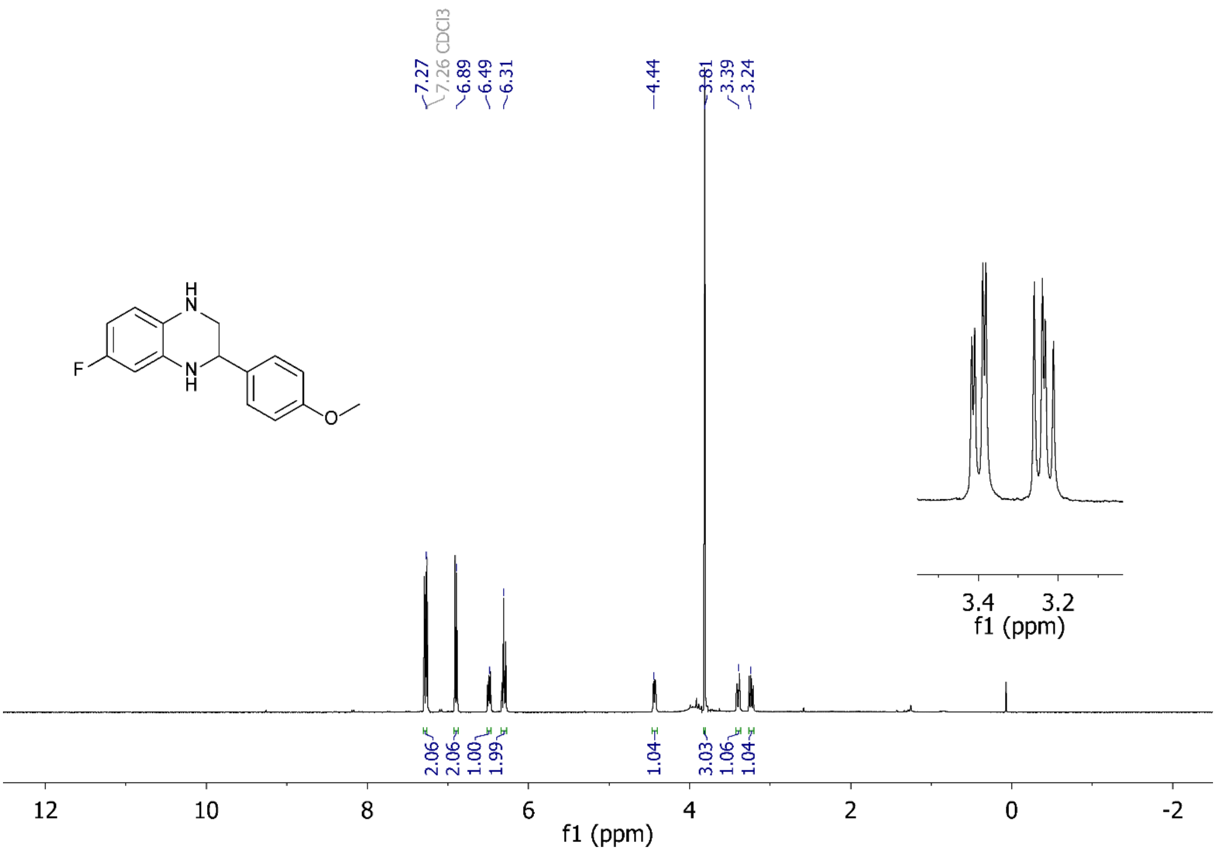

Figure S59. Compound **4e**, ^{1}H NMR (400 MHz, $CDCl_3$).

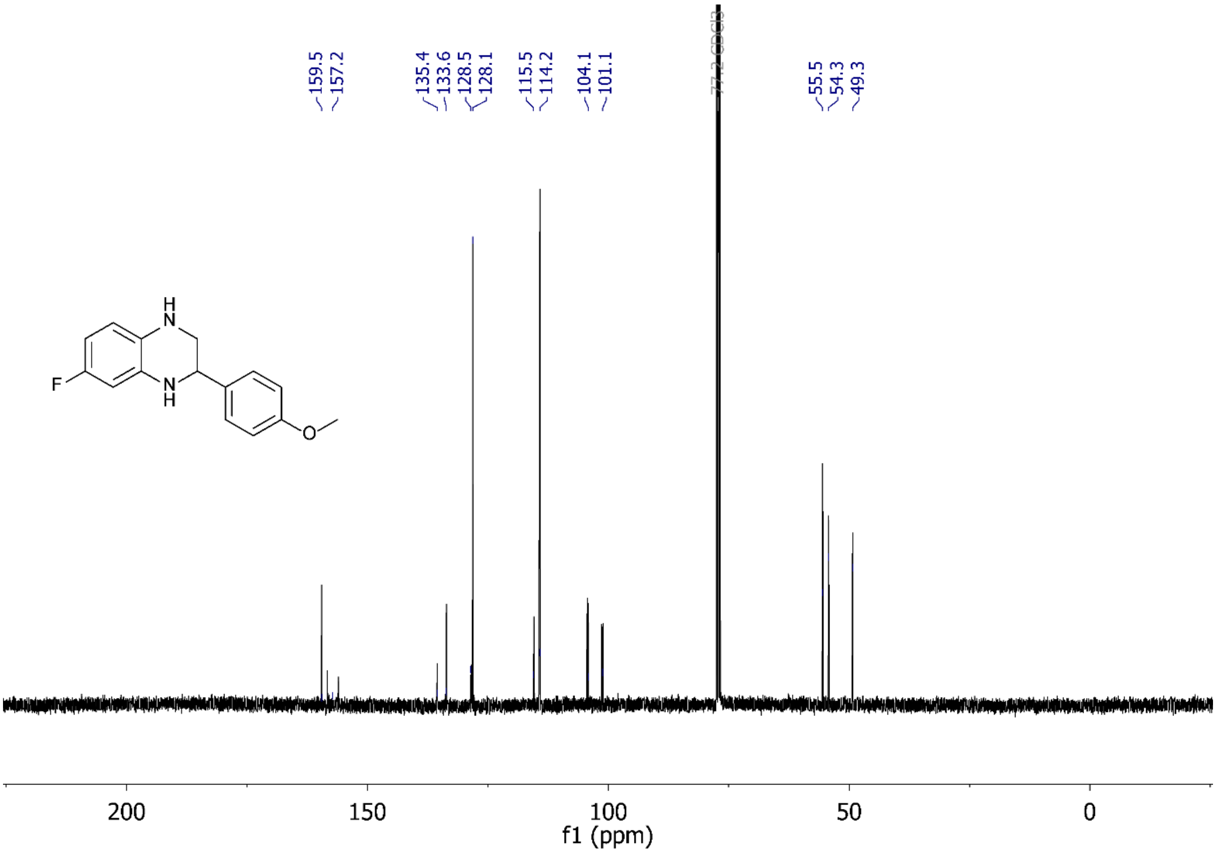

Figure S60. Compound **4e**, ^{13}C NMR (101 MHz, $CDCl_3$).

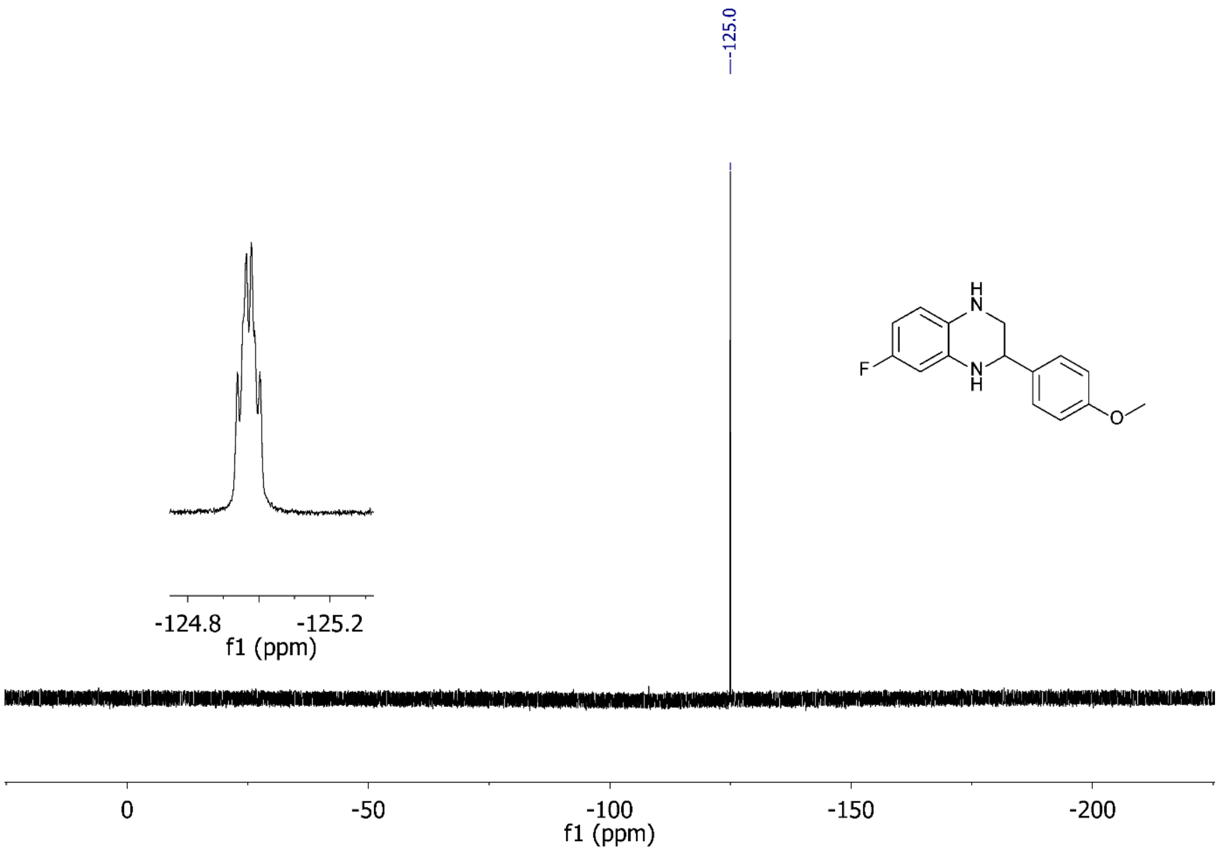

Figure S61. Compound **4e**, ^{19}F NMR (376.5 MHz, $CDCl_3$).

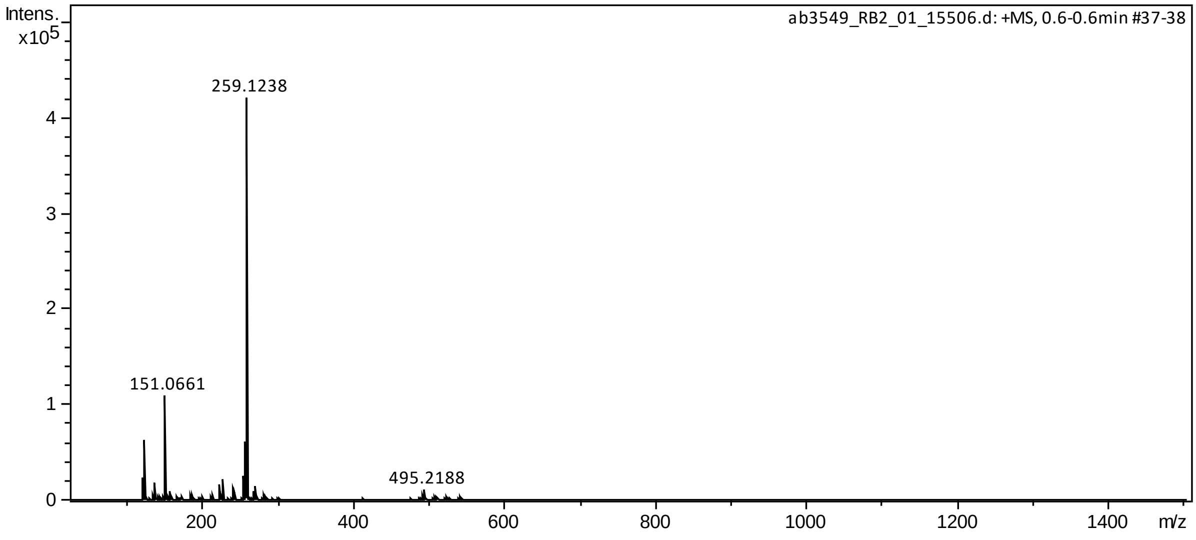

Figure S62. Compound **4e**, HRMS (ESI+) calc for $[C_{15}H_{15}N_2OF+H]^+$: 259.1241 found 259.1238 $[M+H]^+$.

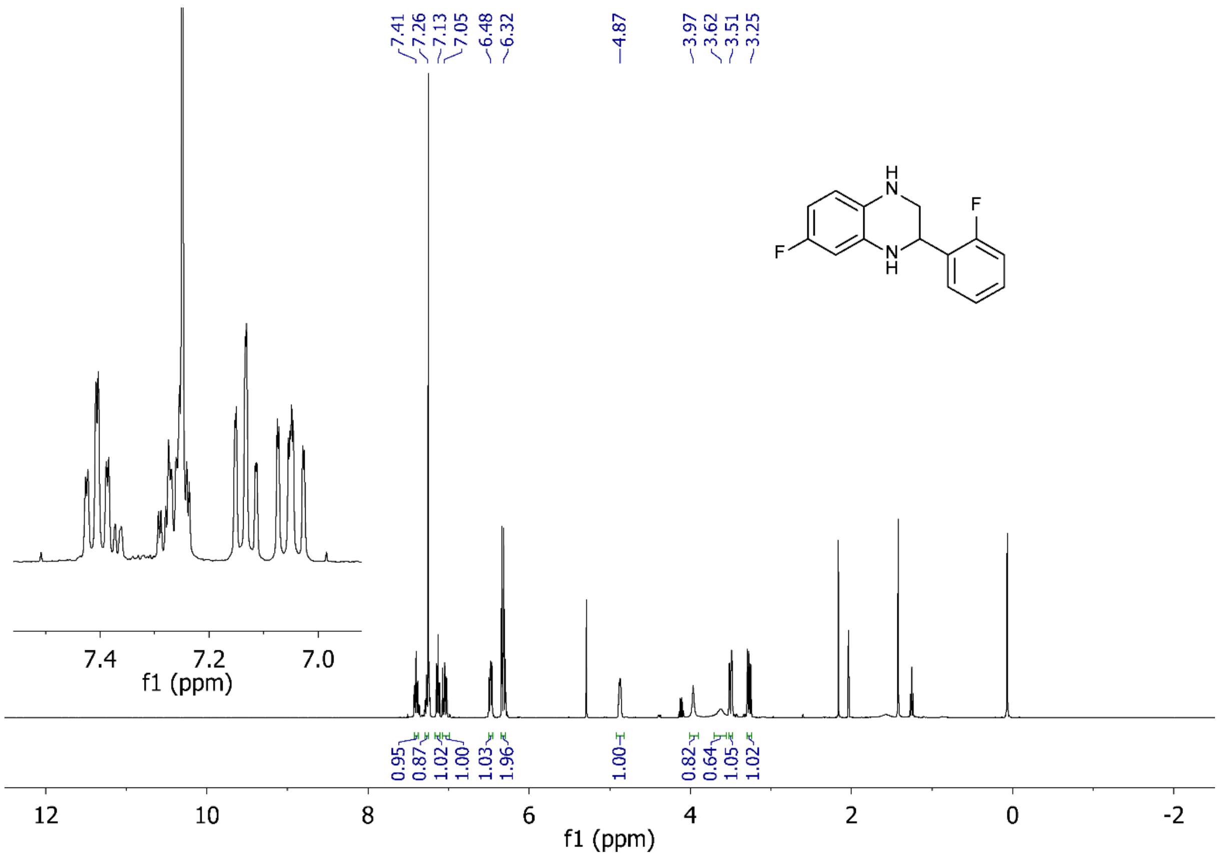

Figure S63. Compound **4f**, ^{1}H NMR (400 MHz, $CDCl_3$).

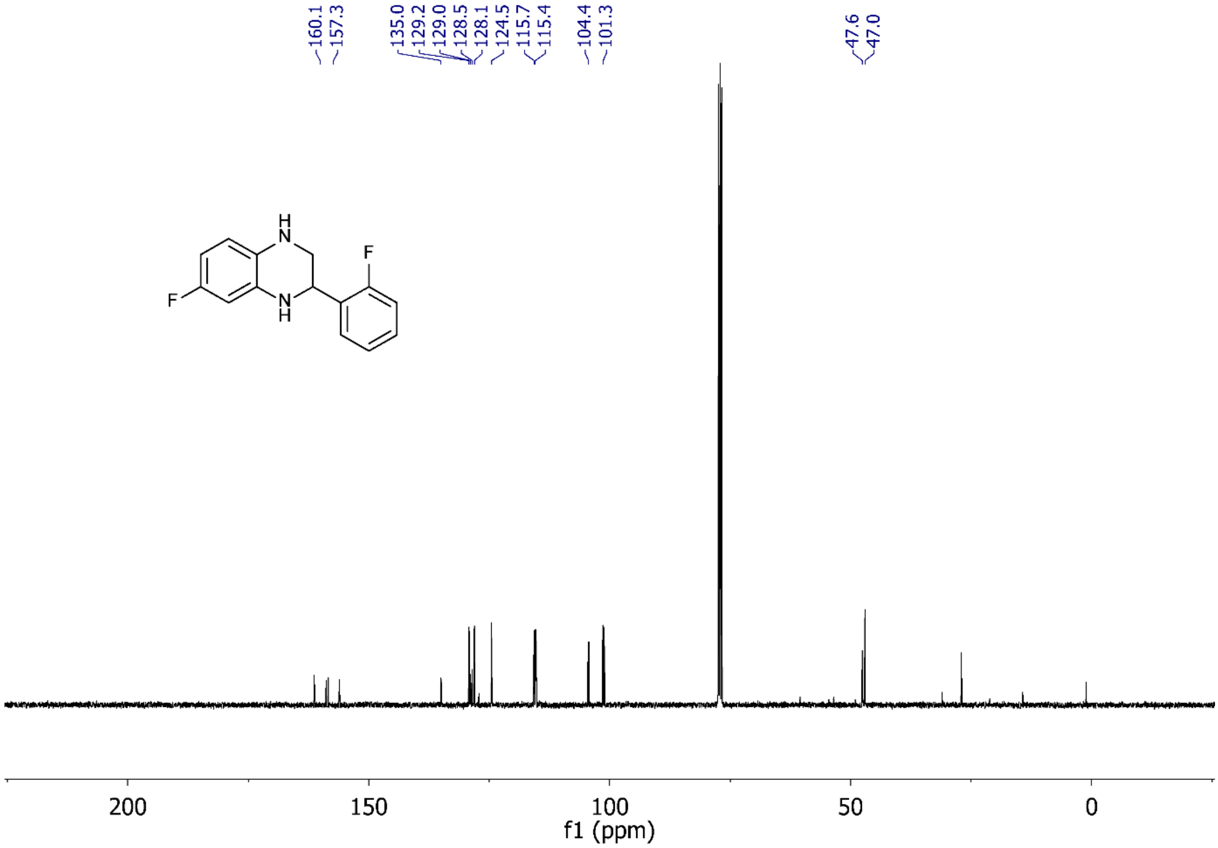

Figure S64. Compound **4f**, ^{13}C NMR (101 MHz, $CDCl_3$).

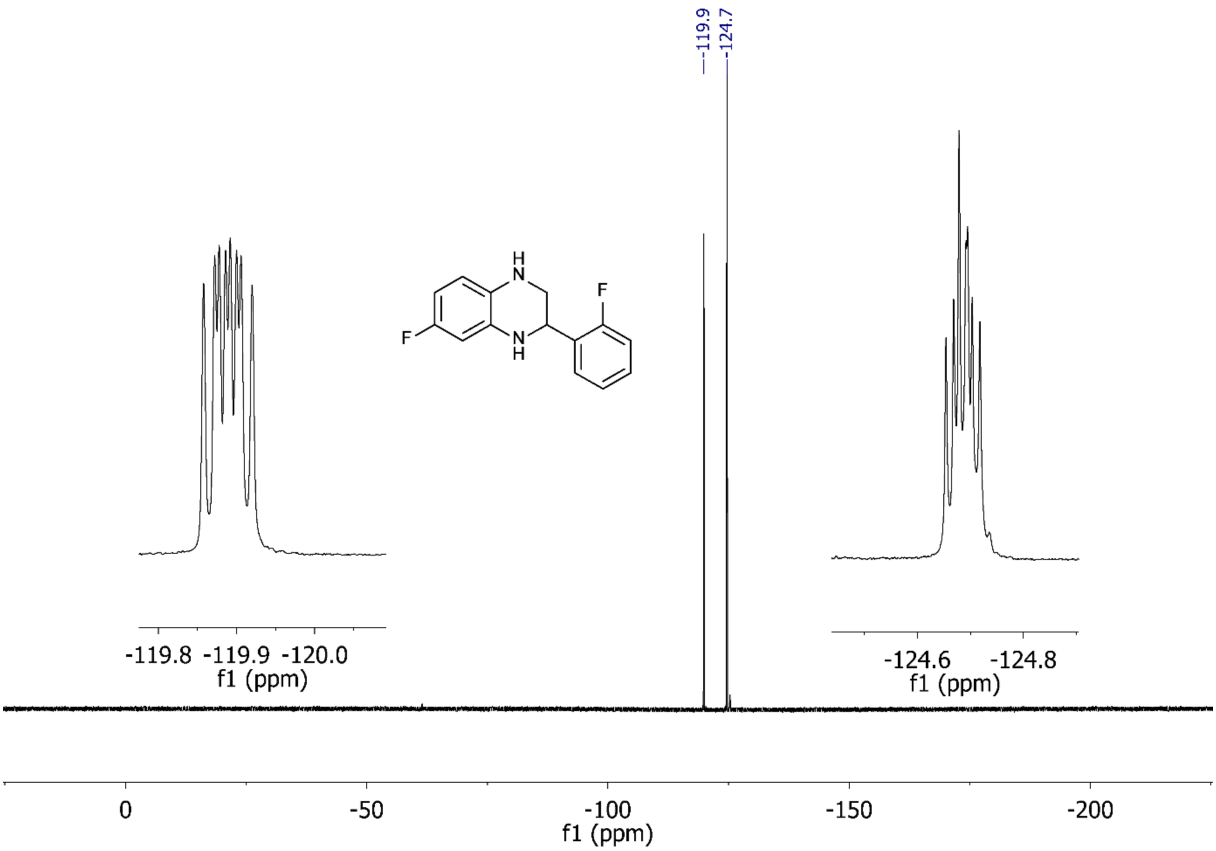

Figure S65. Compound **4f**, ^{19}F NMR (376.5 MHz, $CDCl_3$).

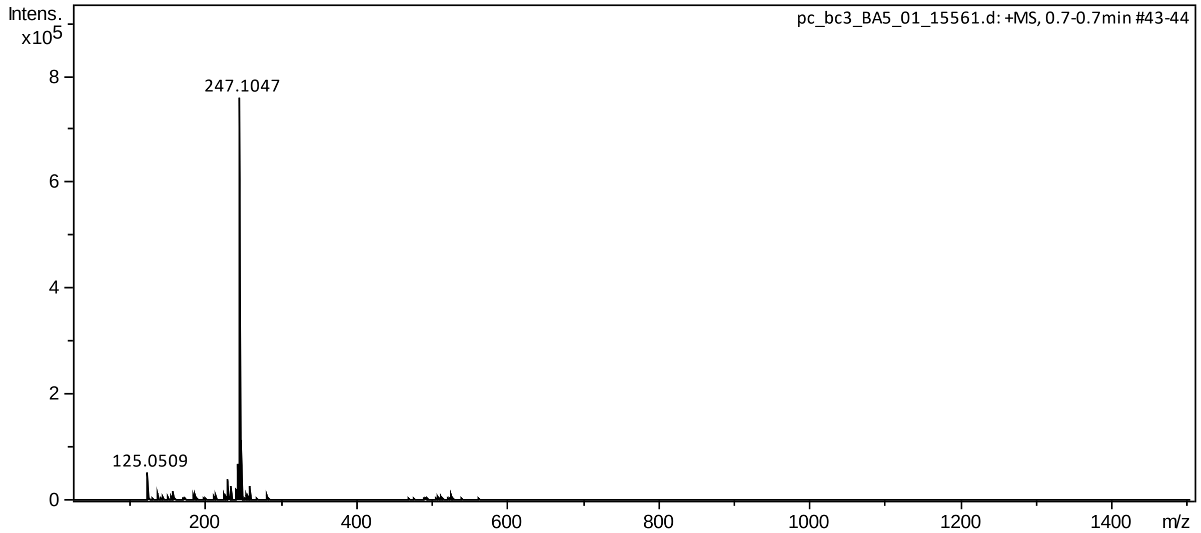

Figure S66. Compound **4f**, HRMS (APCI+) calc for $[C_{14}H_{12}F_2N_2+H]^+$: 247.1041 found 247.1047 $[M+H]^+$

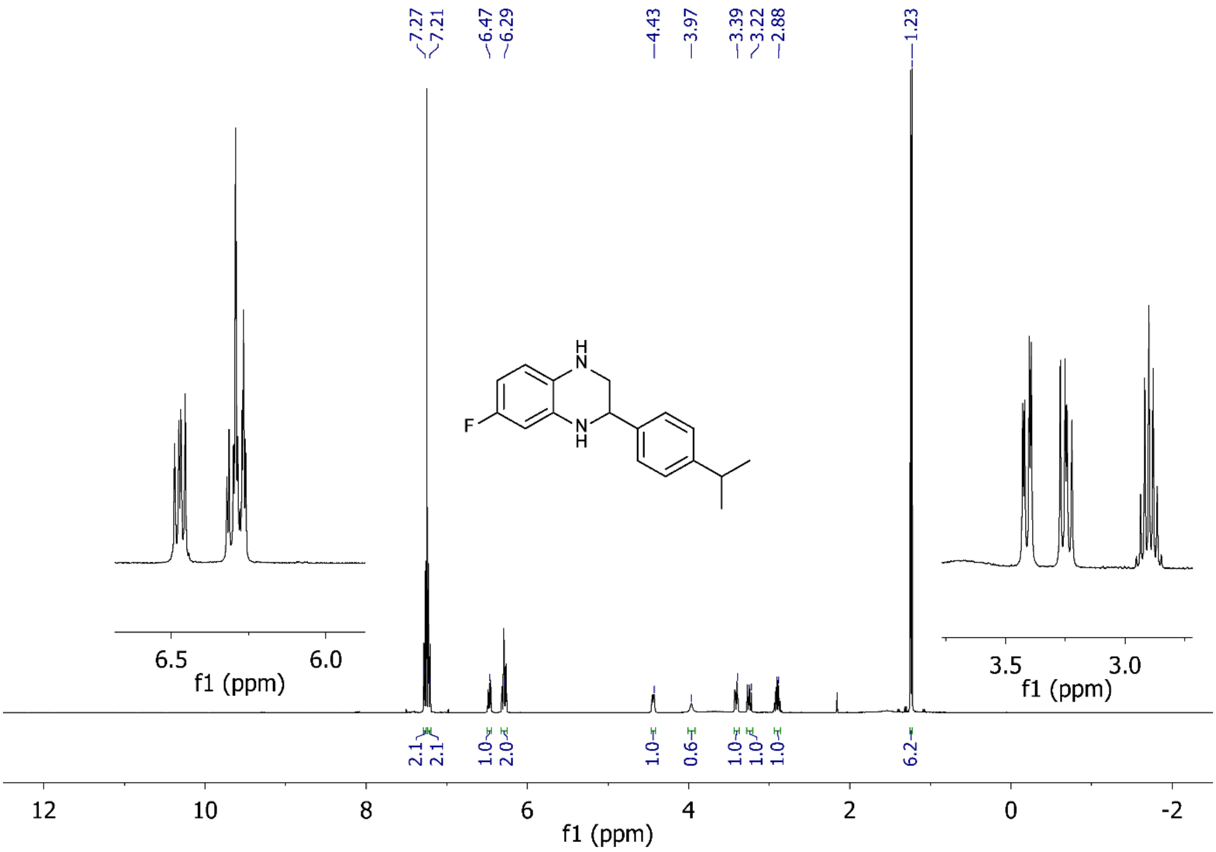

Figure S67. Compound **4g**, ^{1}H NMR (400 MHz, $CDCl_3$).

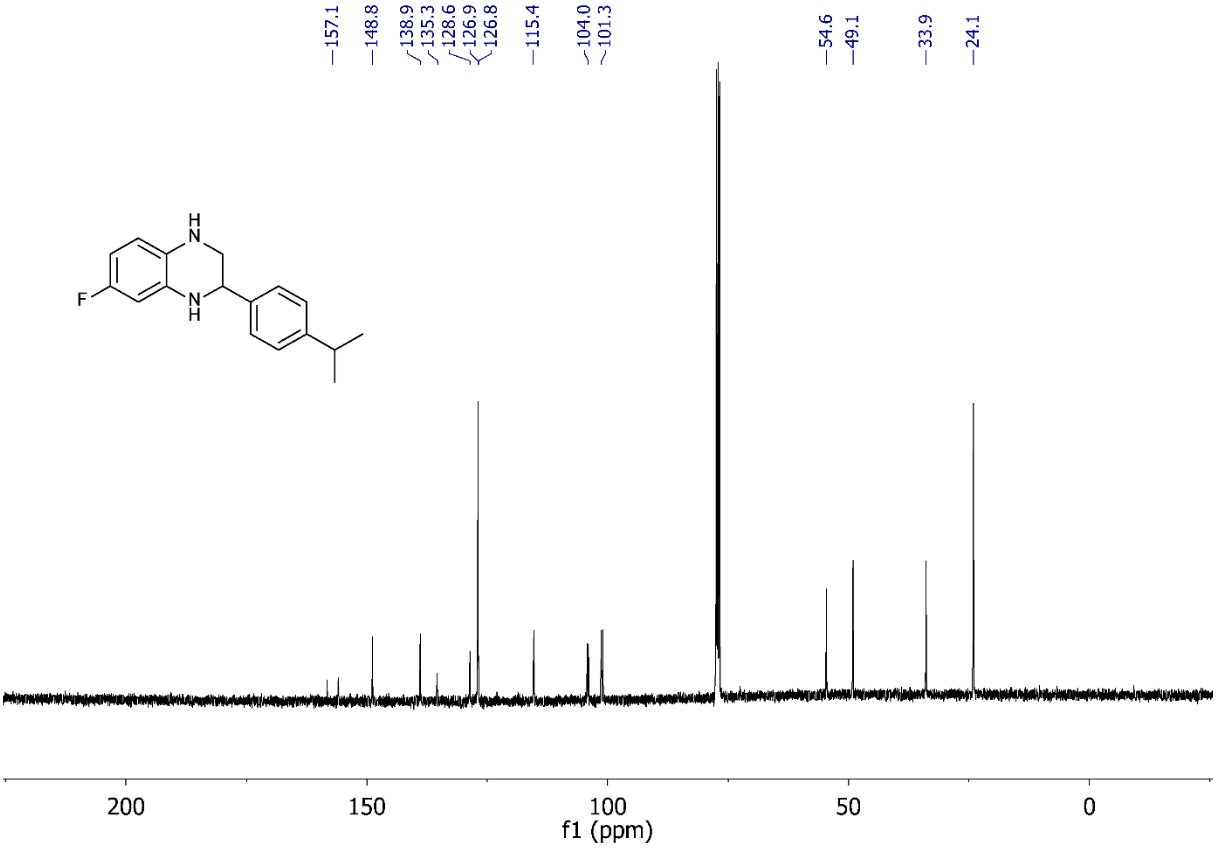

Figure S68. Compound **4g**, ^{13}C NMR (101 MHz, $CDCl_3$).

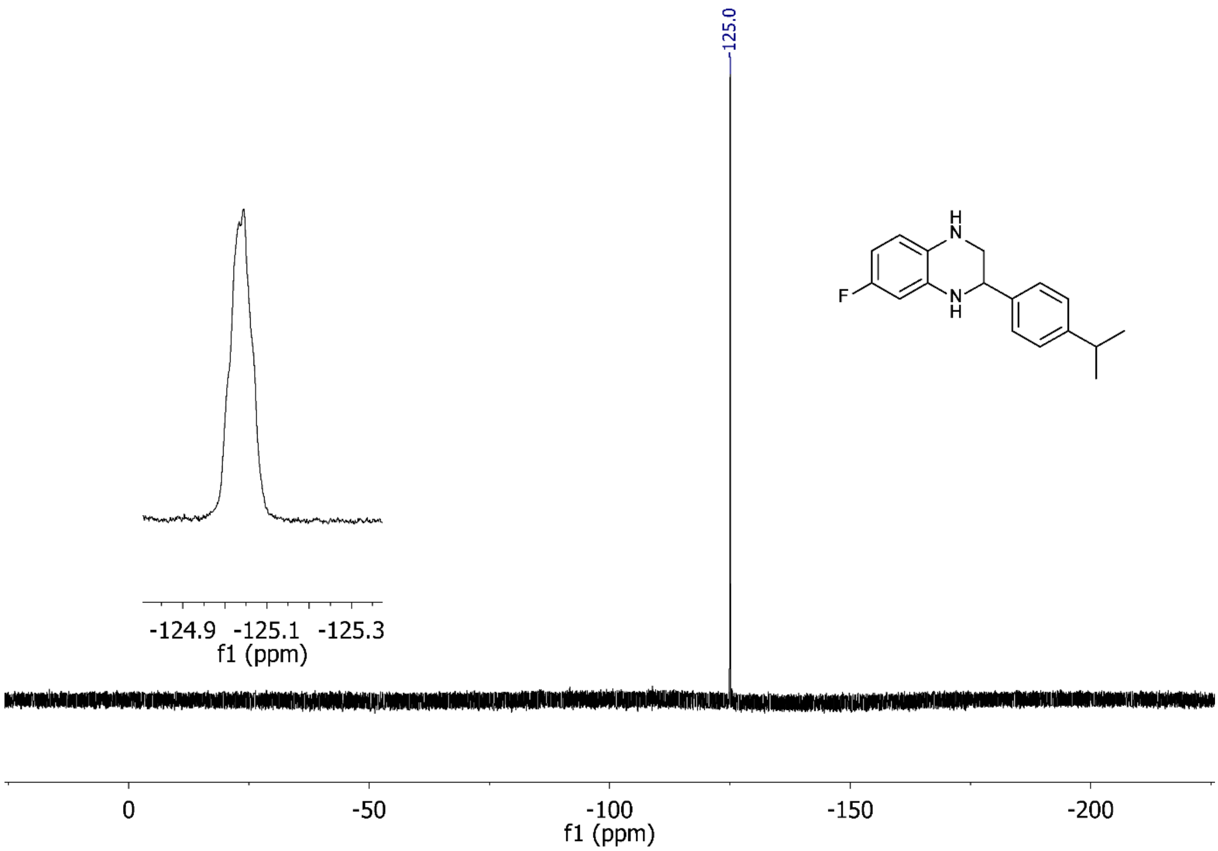

Figure S69. Compound **4g**, ^{19}F NMR (376.5 MHz, $CDCl_3$).

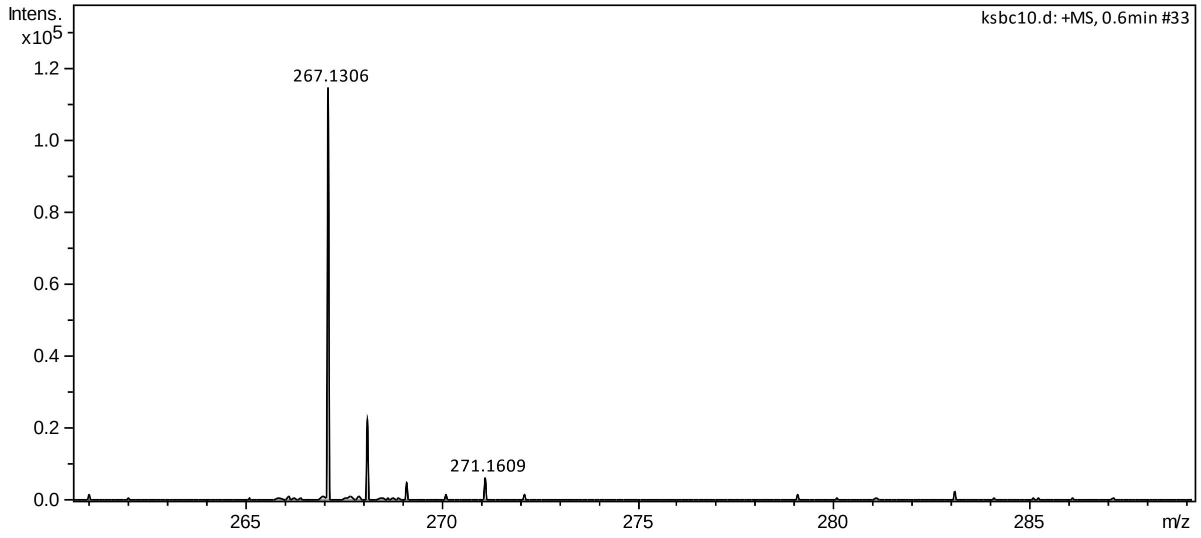

Figure S70. Compound **4g**, HRMS (APCI+) calc for $[C_{17}H_{20}N_2F+H]^+$: 271.1605 found 271.1609 $[M+H]^+$.

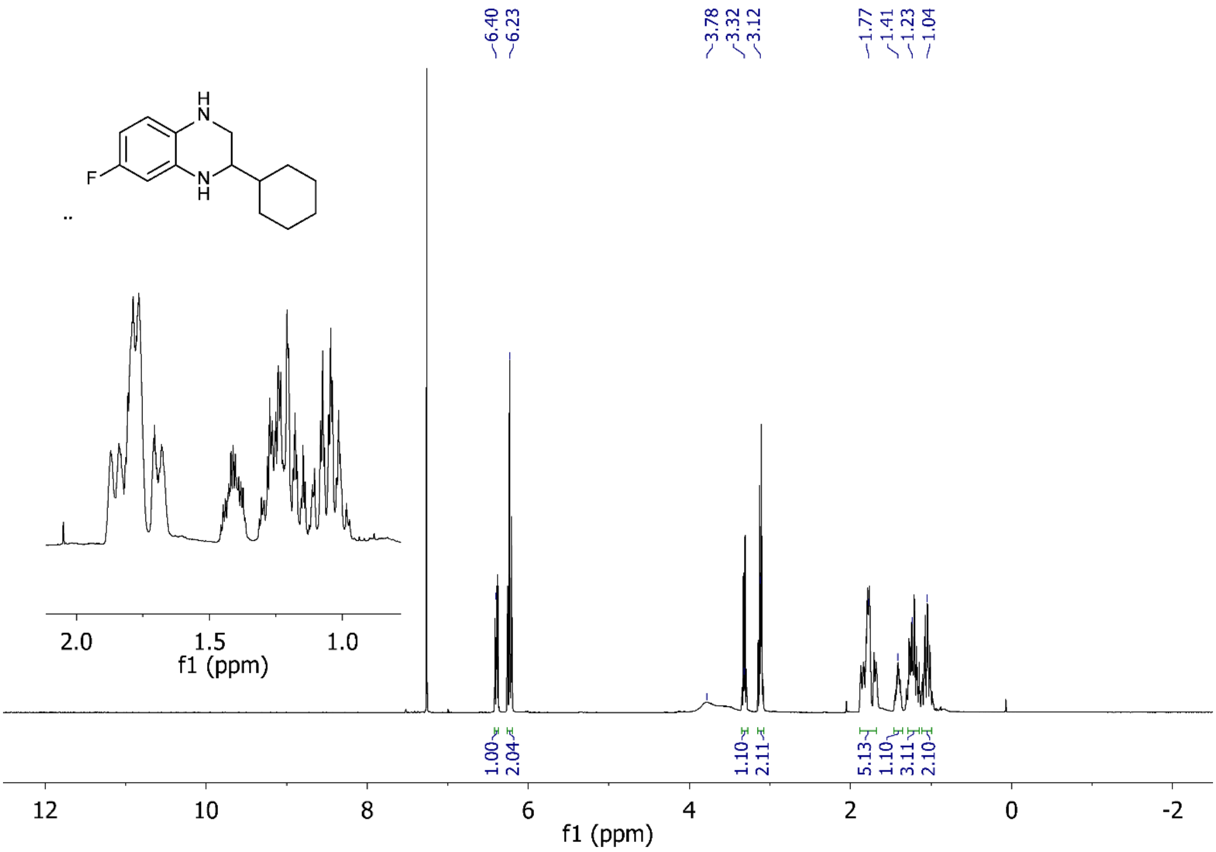

Figure S71. Compound **4h**, ^{1}H NMR (400 MHz, $CDCl_3$).

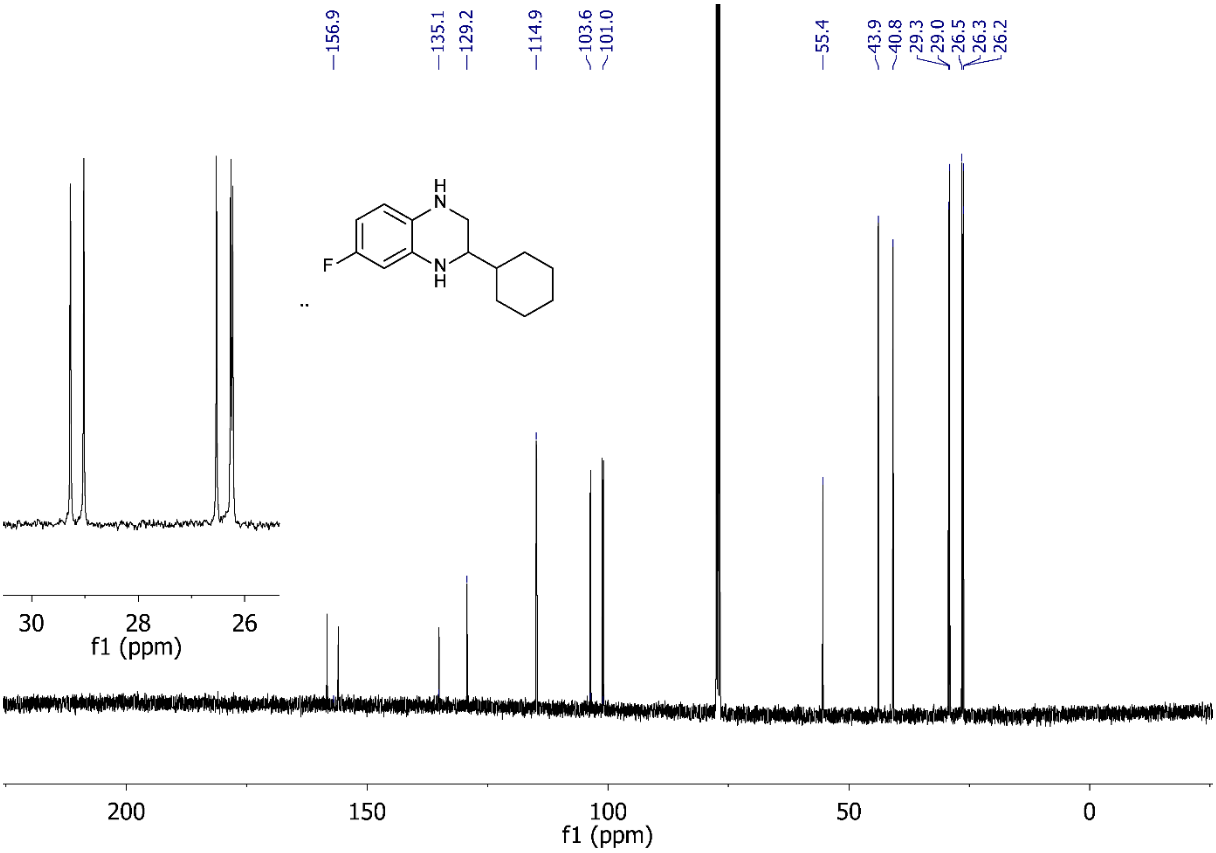

Figure S72. Compound **4h**, ^{13}C NMR (101 MHz, $CDCl_3$).

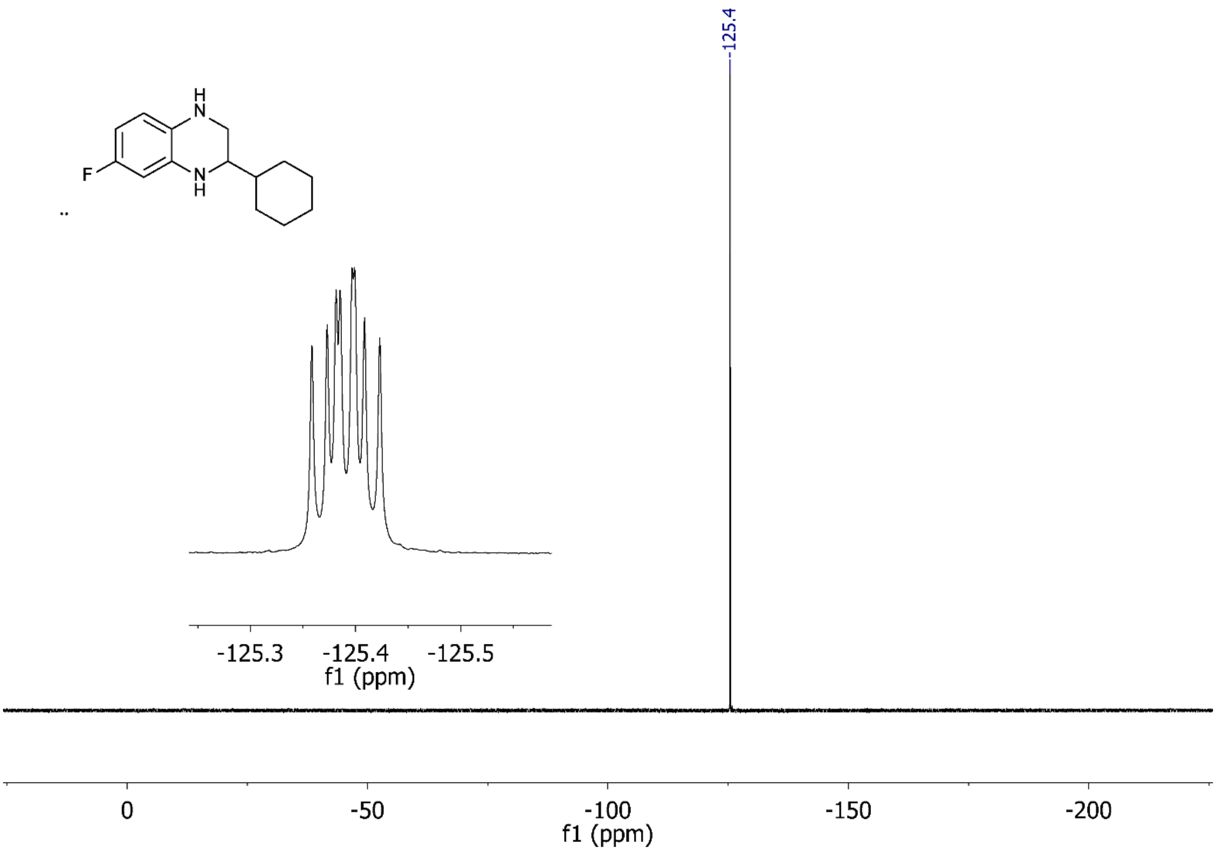

Figure S73. Compound **4h**, ^{19}F NMR (376.5 MHz, $CDCl_3$).

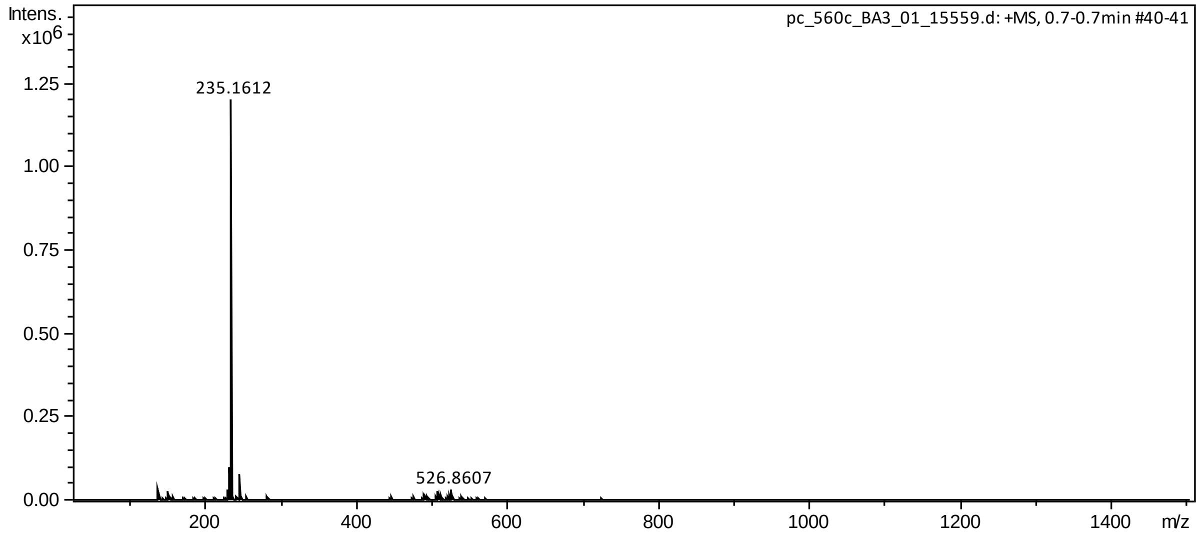

Figure S74. Compound **4h**, HRMS (ESI+) calc for $[C_{14}H_{19}FN_2+H]^+$ 235.1605, found 235.1612 $[M+H]^+$.

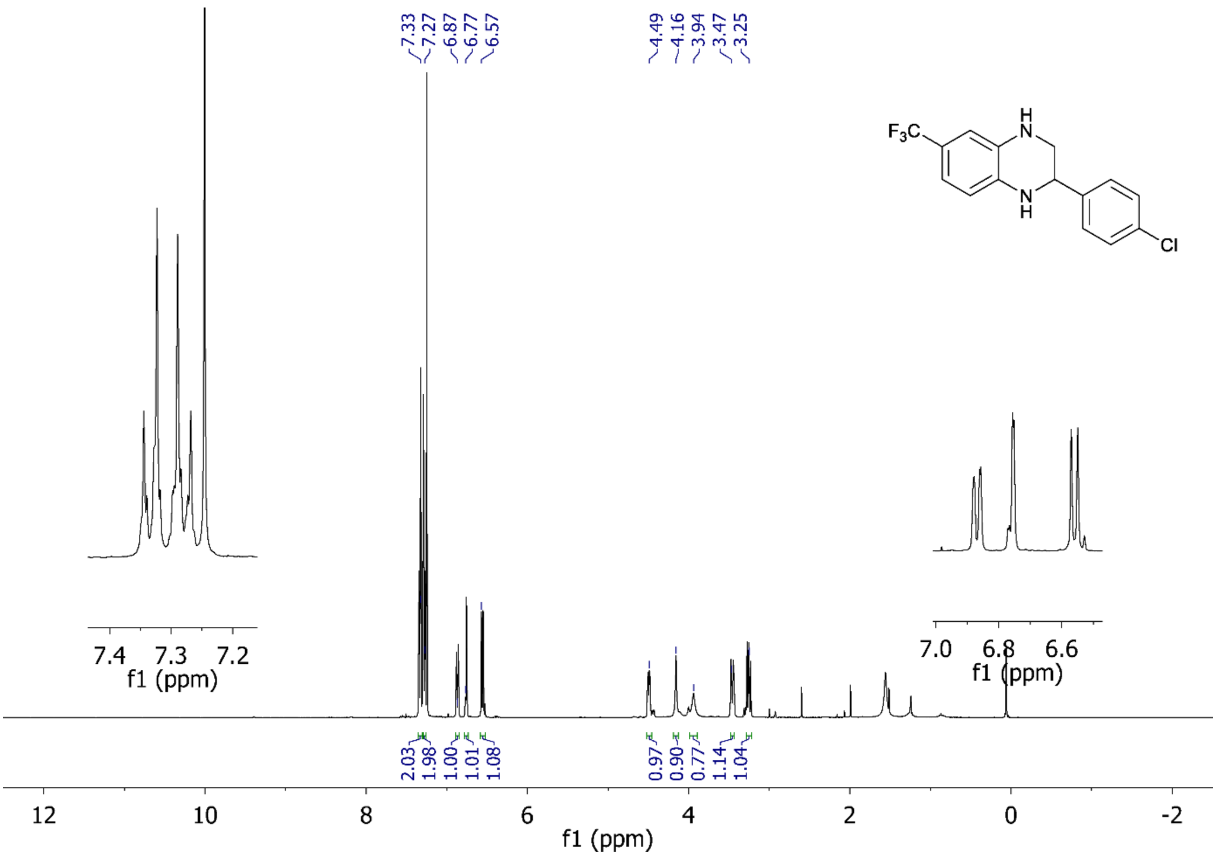

Figure S75. Compound **5a**, ^{1}H NMR (400 MHz, $CDCl_3$).

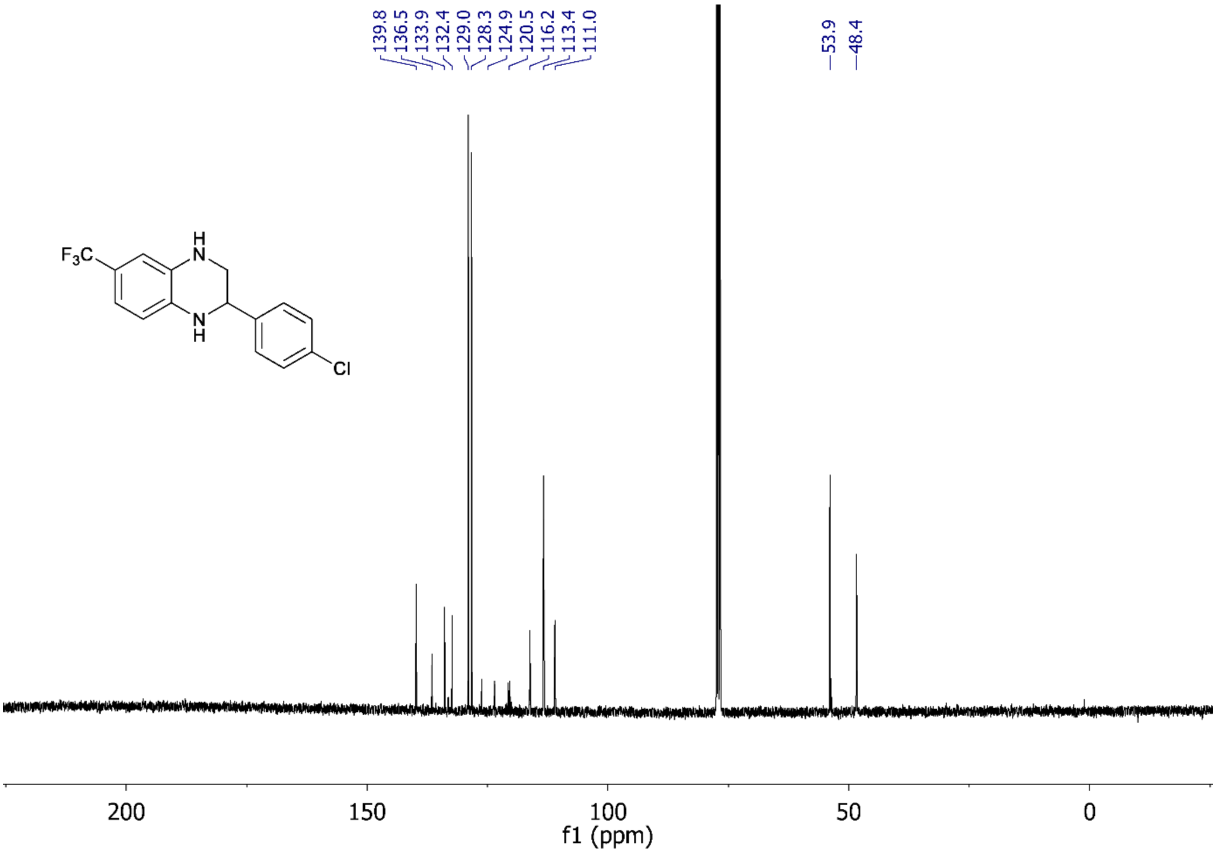

Figure S76. Compound **5a**, ^{13}C NMR (101 MHz, $CDCl_3$).

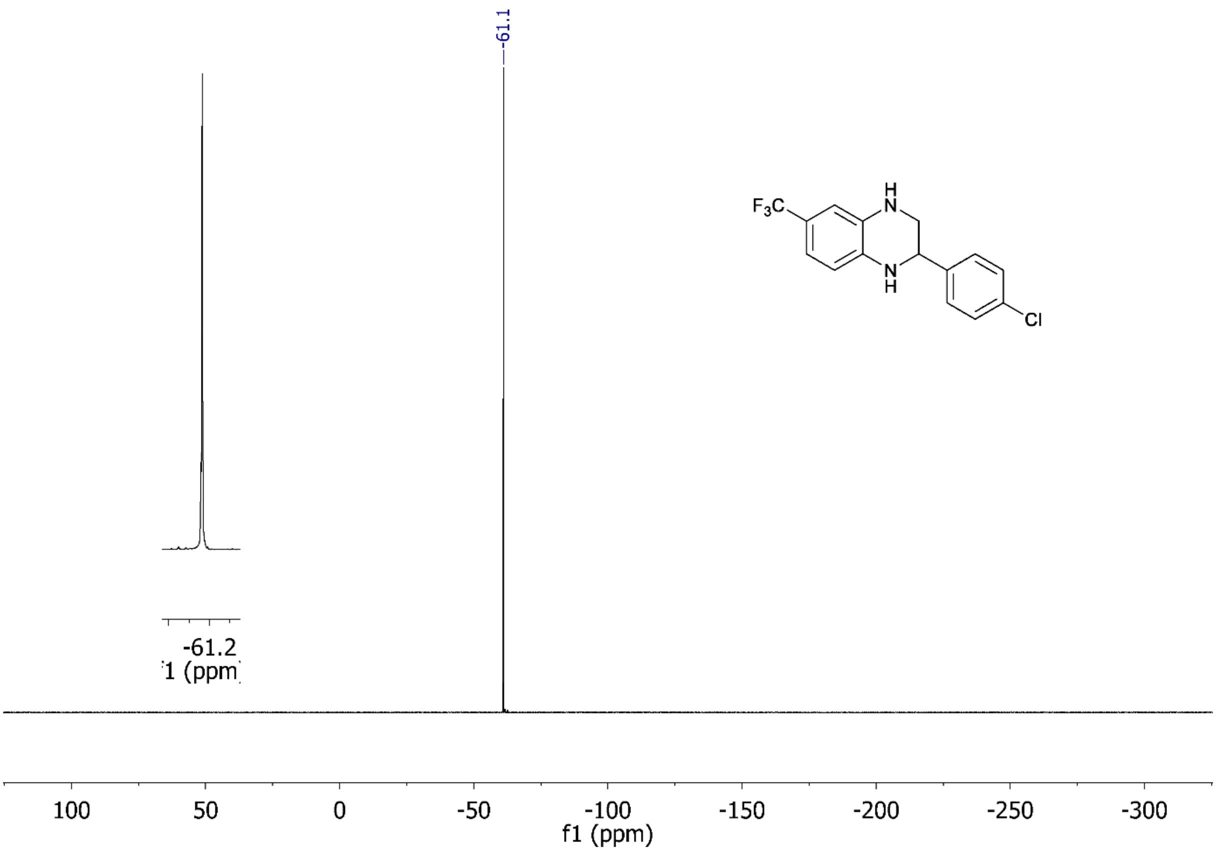

Figure S77. Compound **5a**, ^{19}F NMR (376.5 MHz, $CDCl_3$).

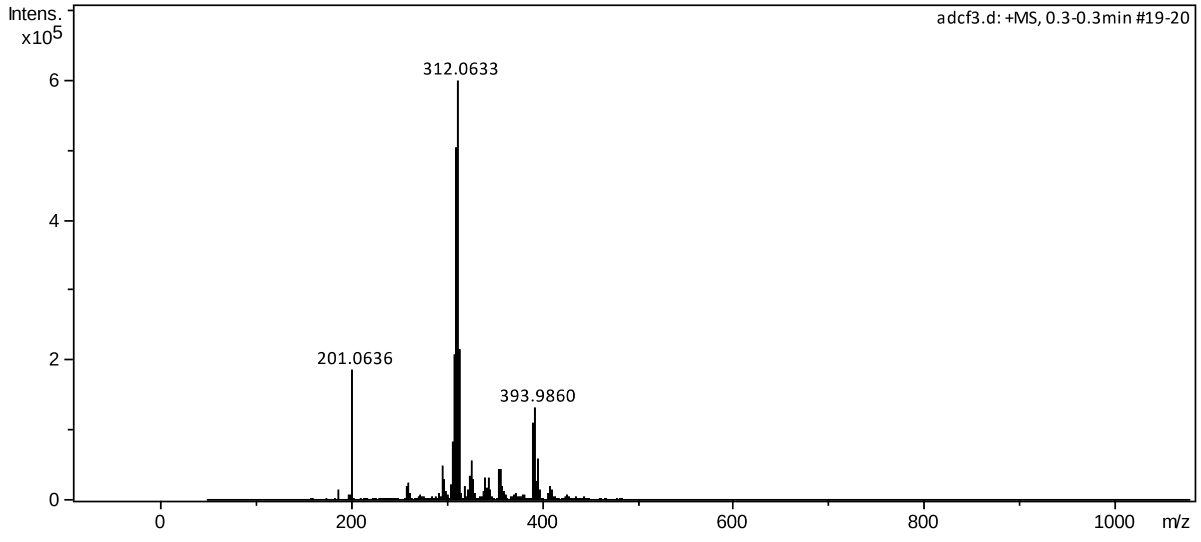

Figure S78. Compound **5a**, , HRMS (APCI+) calc for $[C_{15}H_{12}N_2ClF_3+H]^+$: 312.0635 found 312.0633 $[M+H]^+$

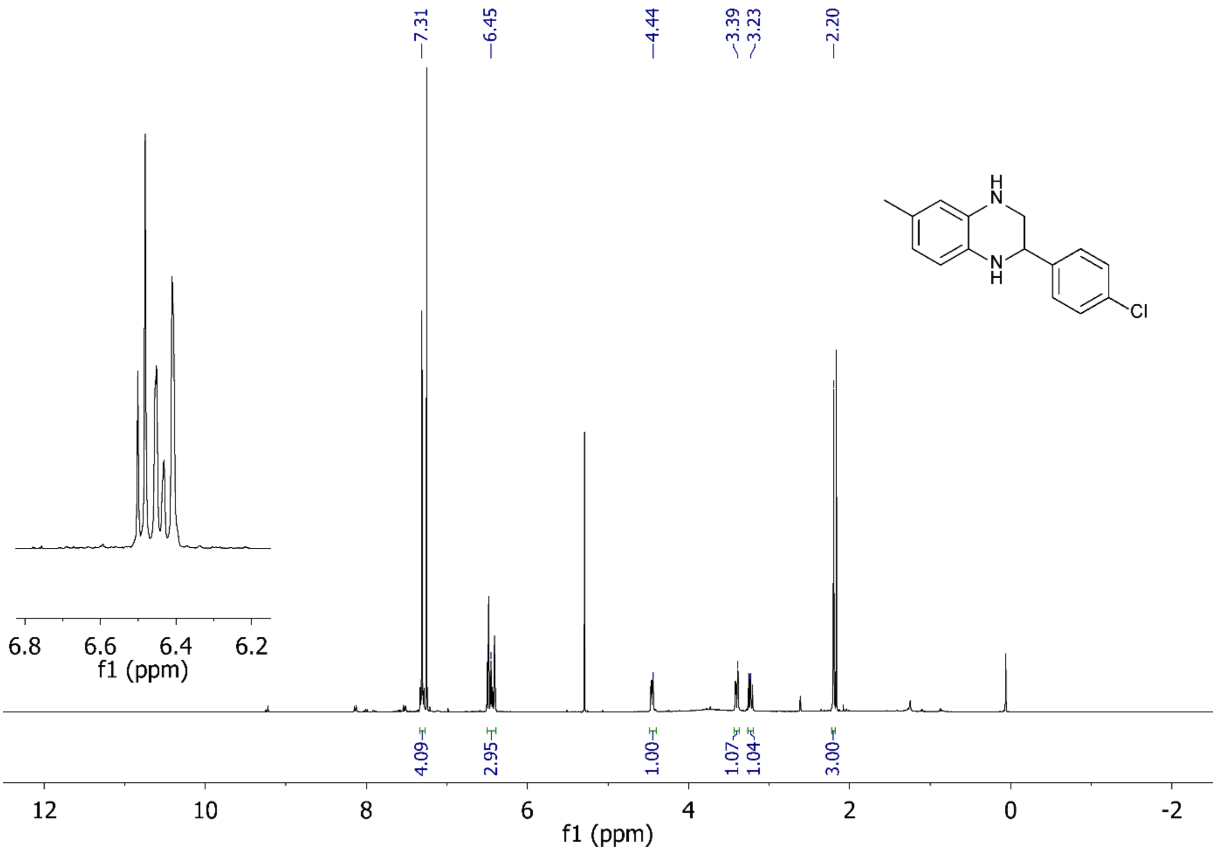

Figure S79. Compound **5b**, ^{1}H NMR (400 MHz, $CDCl_3$).

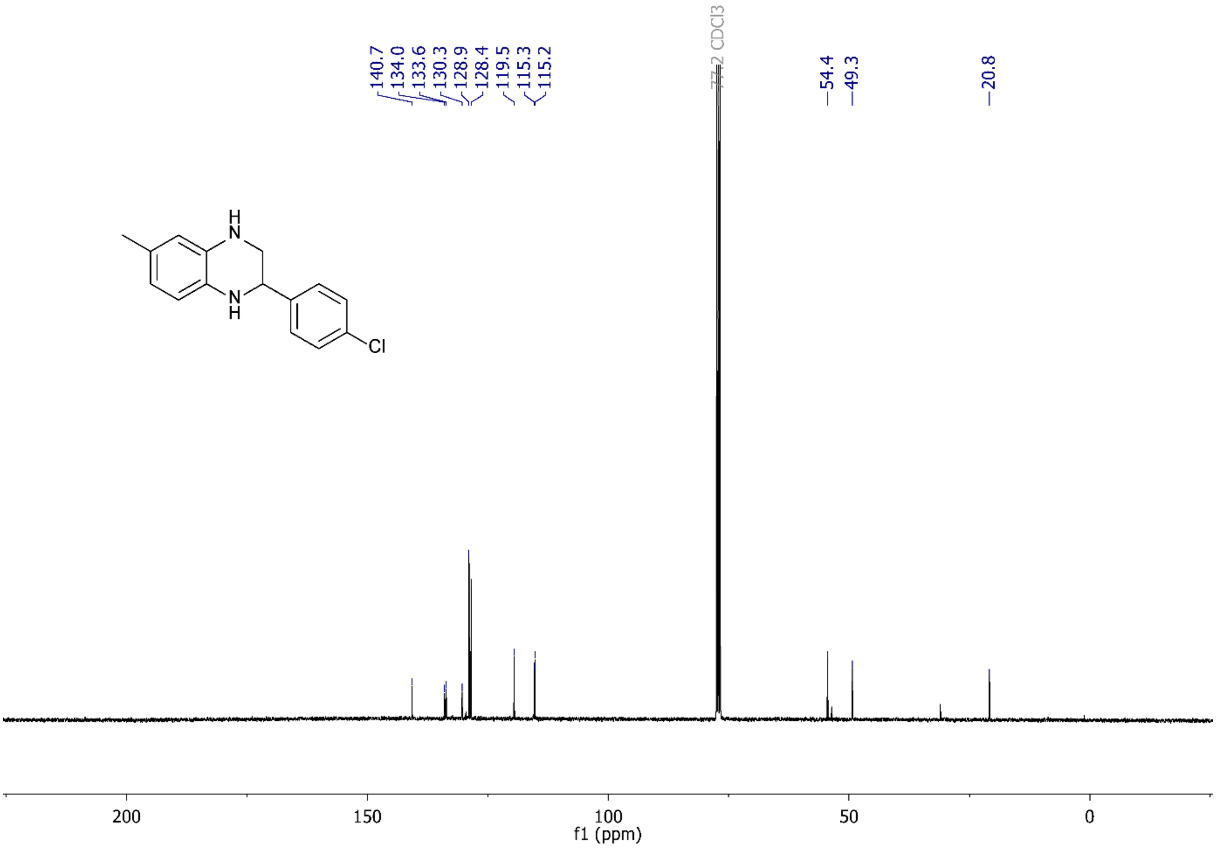

Figure S80. Compound **5b**, ^{13}C NMR (101 MHz, $CDCl_3$).

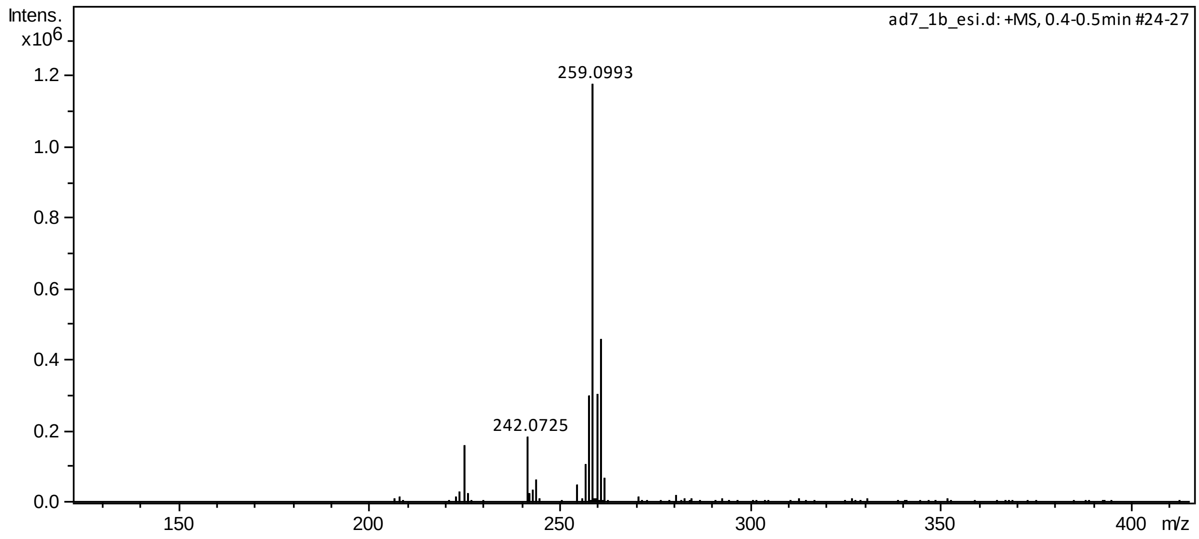

Figure S81. Compound **5b**, HRMS (APCI+) calc for $[C_{15}H_{15}ClN_2+H]^+$: 259.0996 found 259.0993 $[M+H]^+$.

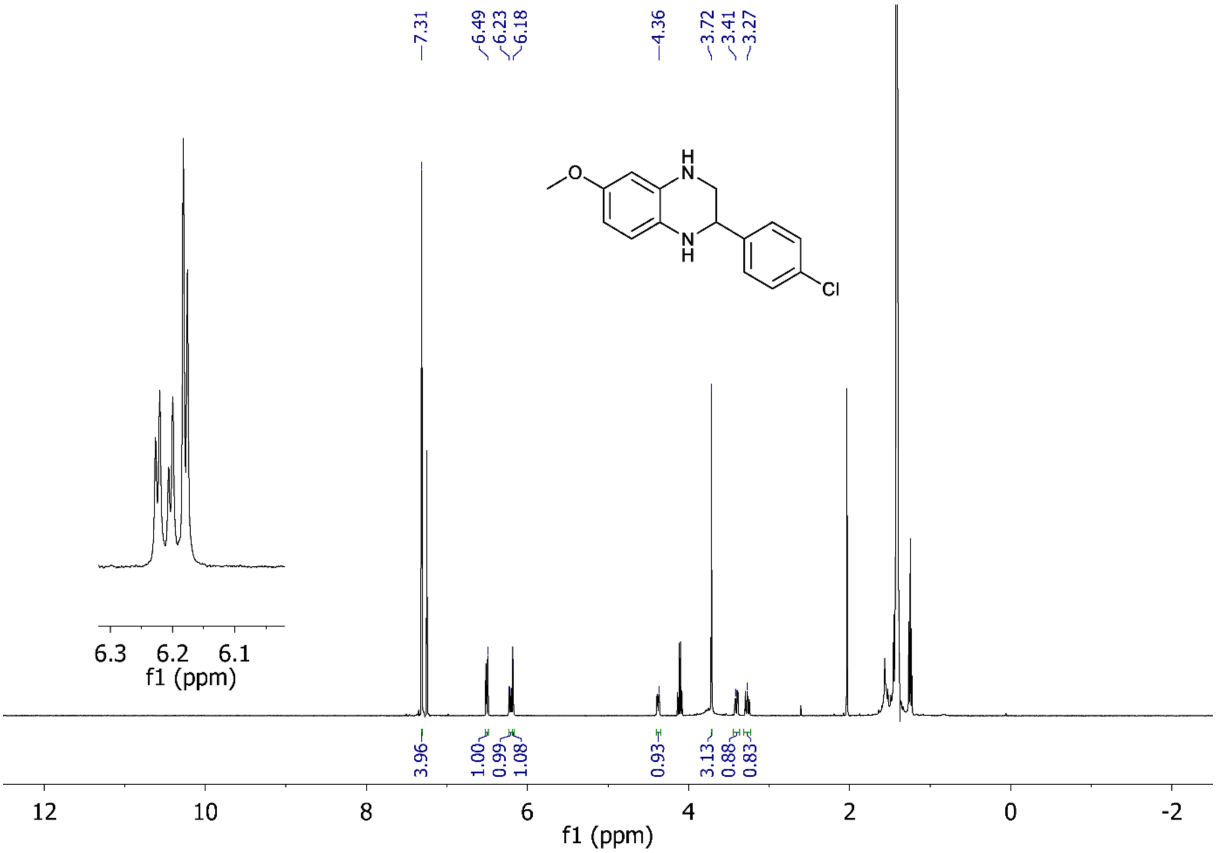

Figure S82. Compound **5c**, ^{1}H NMR (400 MHz, $CDCl_3$).

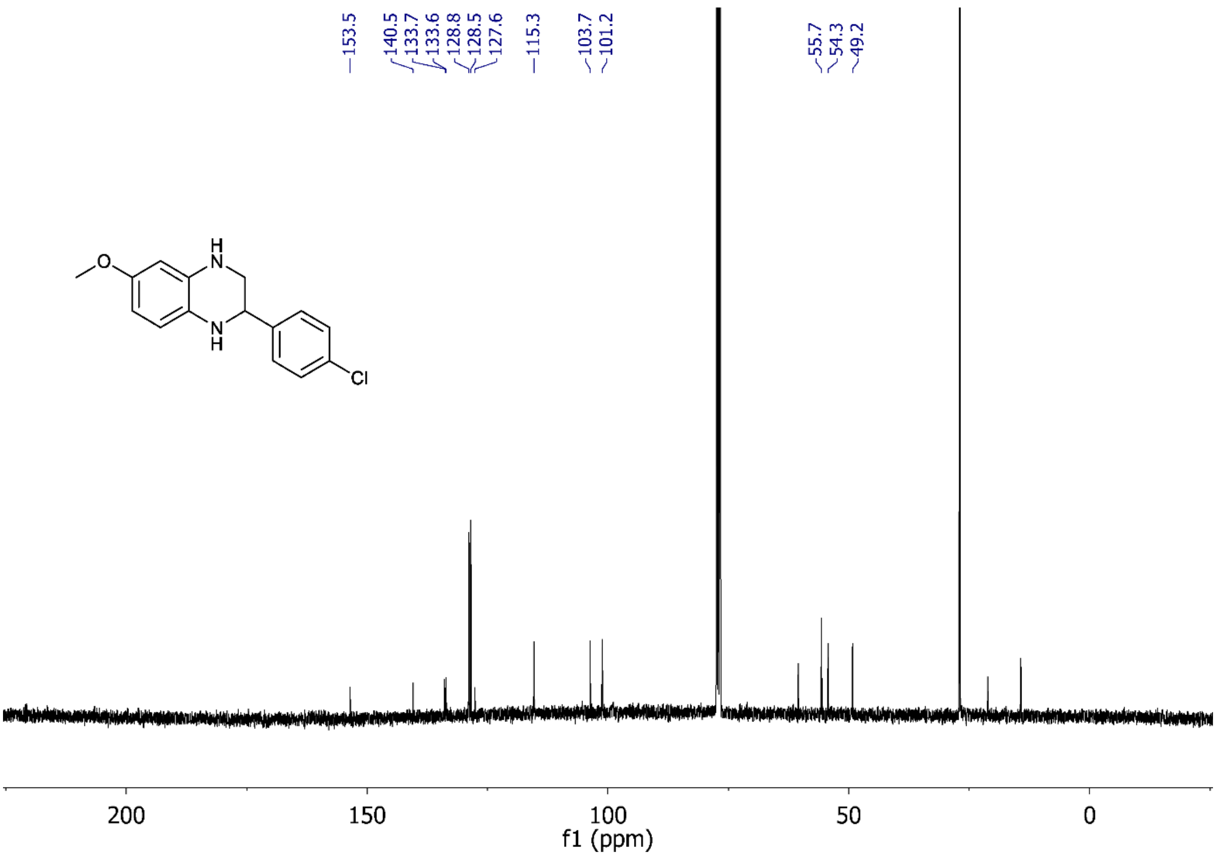

Figure S83. Compound **5c**, ^{13}C NMR (101 MHz, $CDCl_3$).

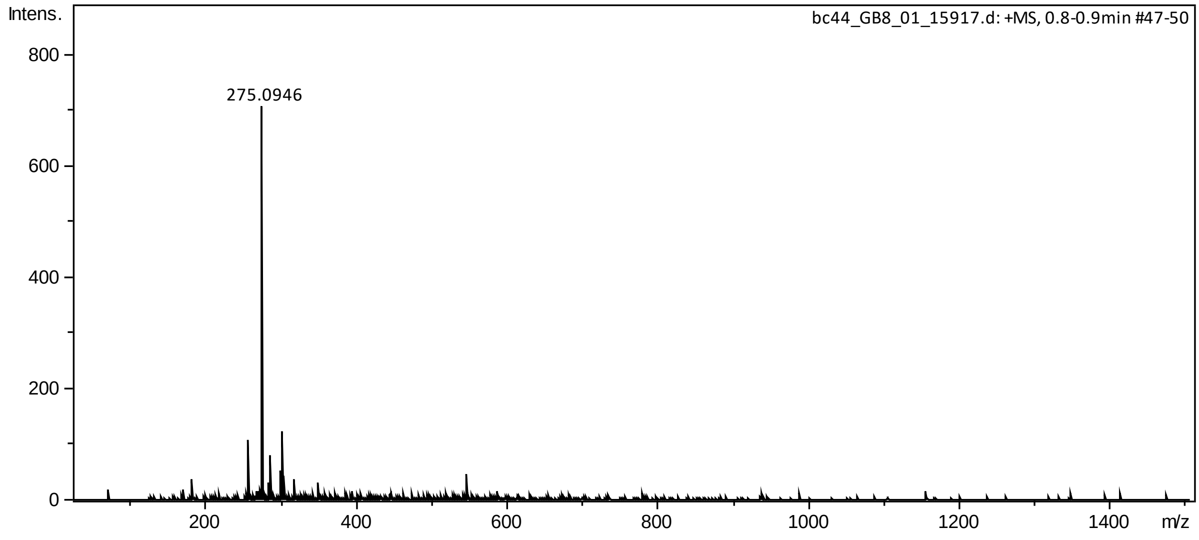

Figure S84. Compound **5c**, HRMS (ESI+) calc for $[C_{15}H_{15}OClN_2+H]^+$: 275.0945, found 275.0946 $[M+H]^+$.

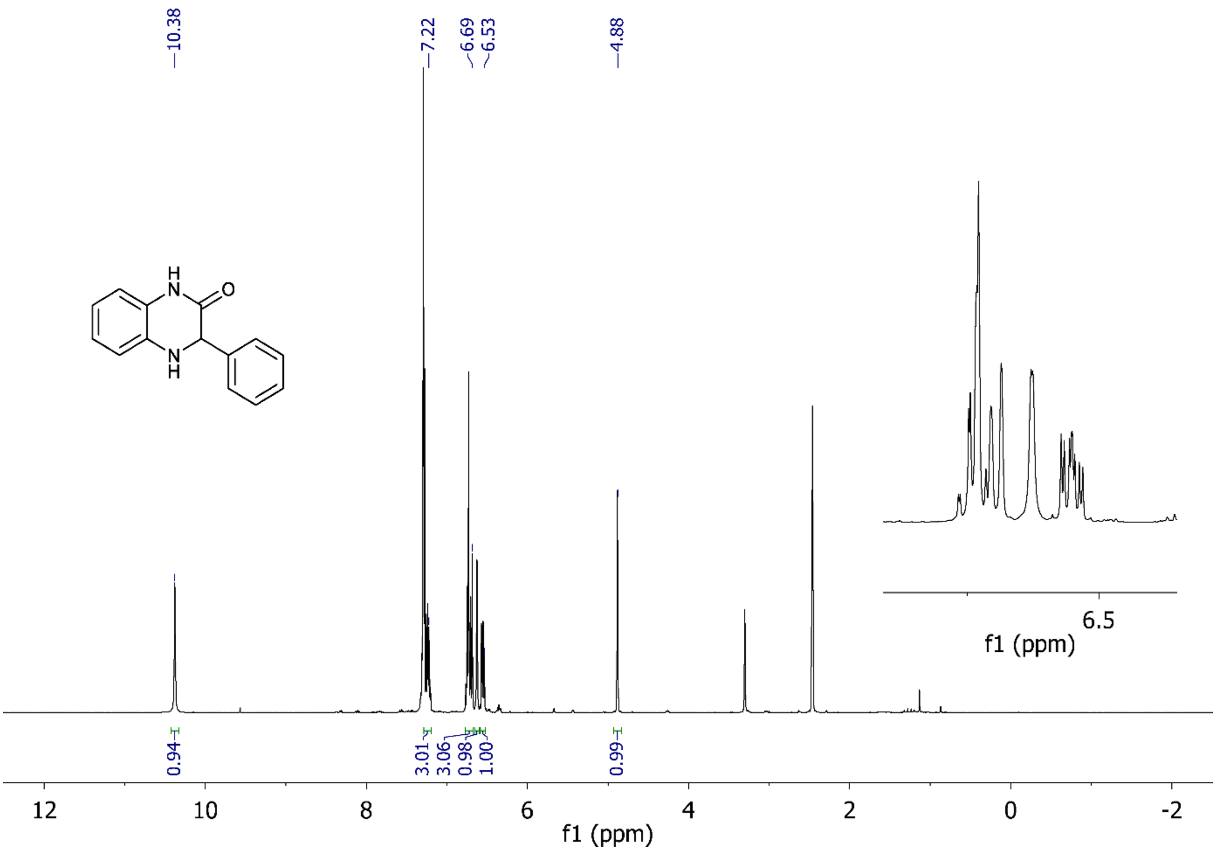

Figure S85. Compound **6a**, ^{1}H NMR (400 MHz, DMSO-d_6).

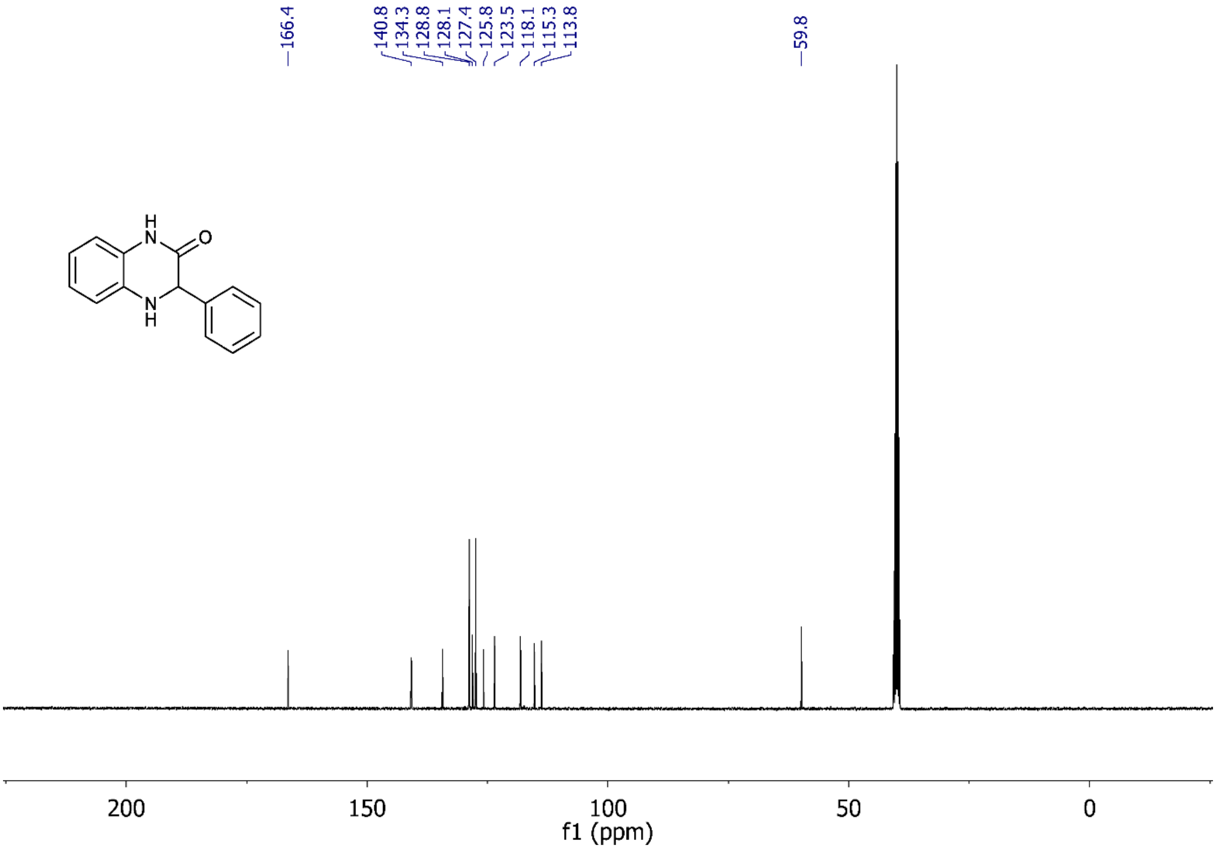

Figure S86. Compound **6a**, ^{13}C NMR (101 MHz, DMSO-*d*$_6$).

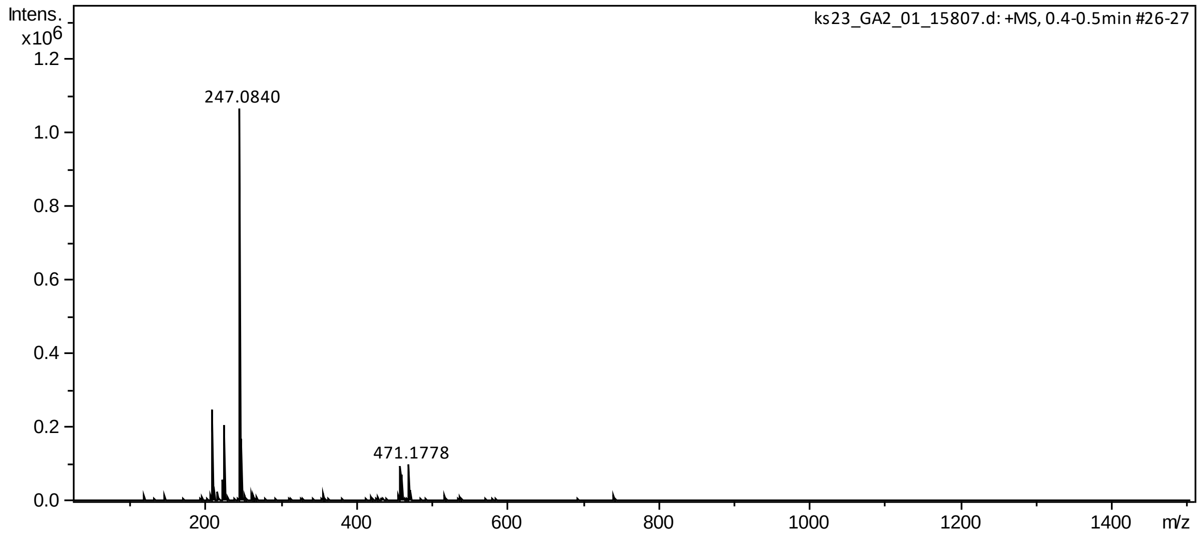

Figure S87. Compound **6a**, HRMS (ESI+) calc for $[C_{14}H_{12}N_2O+Na]^+$: 248.0841 found 248.0840 $[M+Na]^+$.

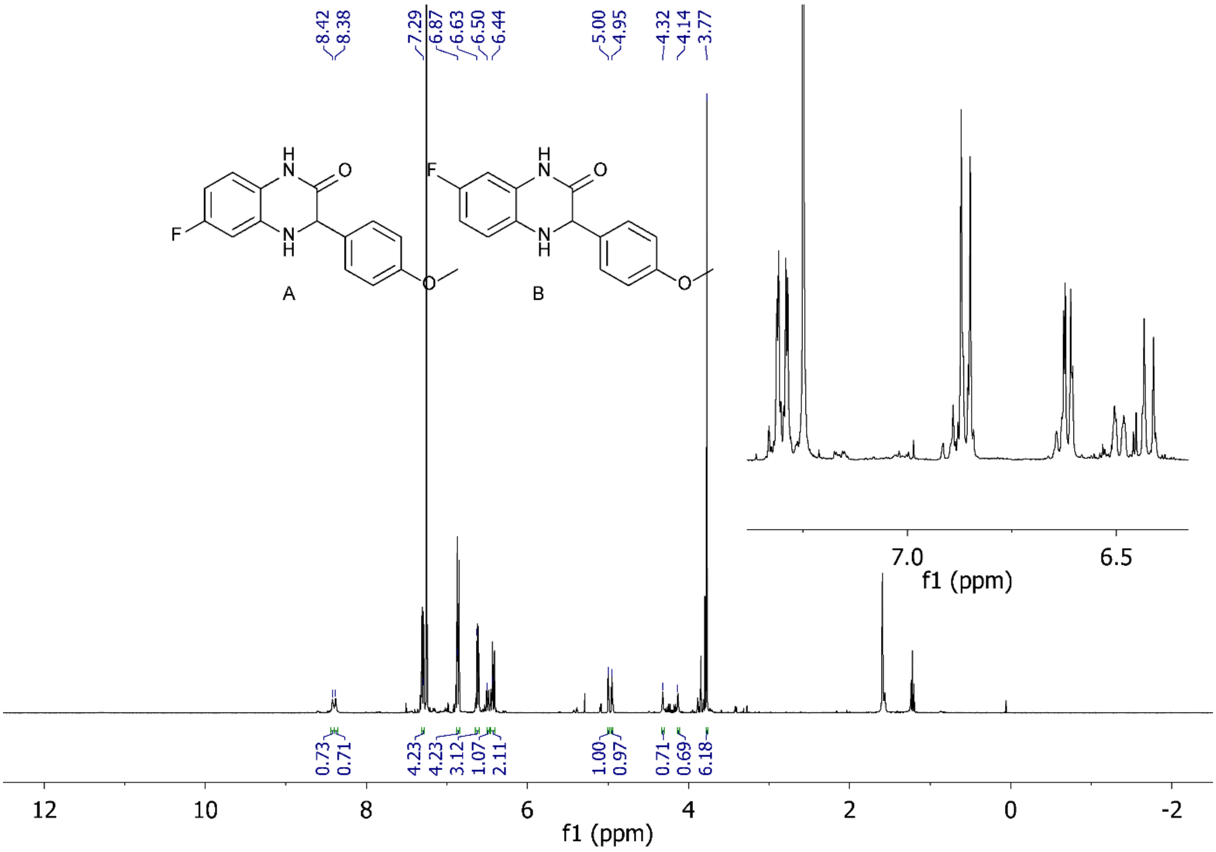

Figure S88. Compound **6b**, ^1H NMR (400 MHz, $CDCl_3$).

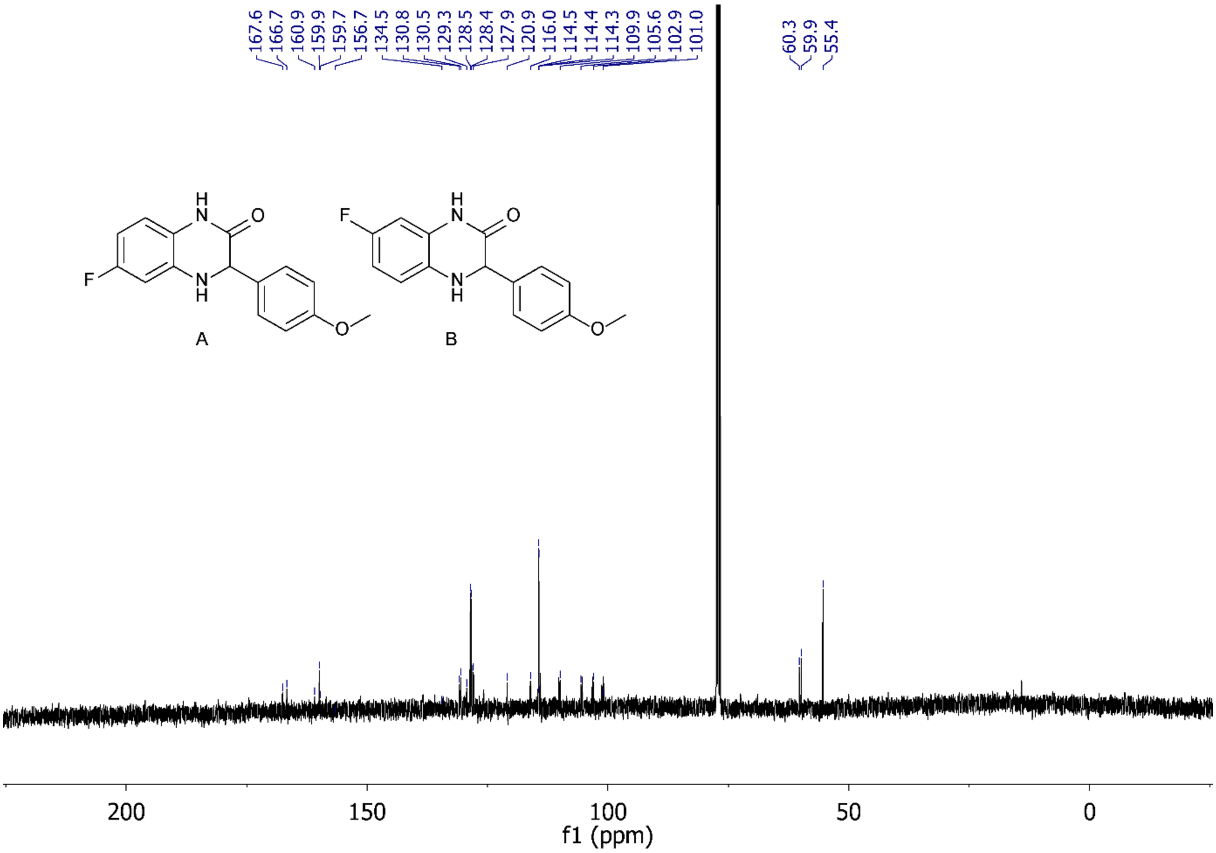

Figure S89. Compound **6b**, ^{13}C NMR (101 MHz, $CDCl_3$).

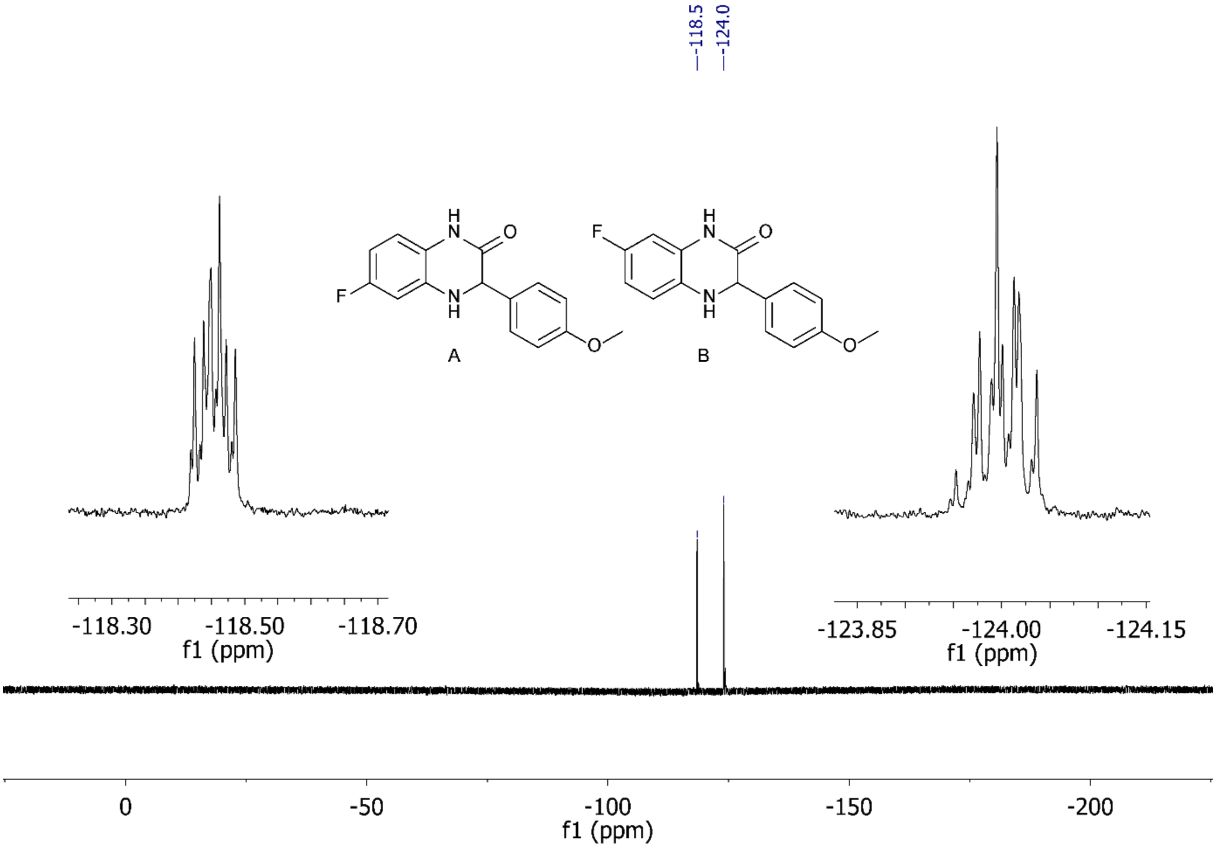

Figure S90. Compound **6b**, ^{19}F NMR (376.5 MHz, $CDCl_3$).

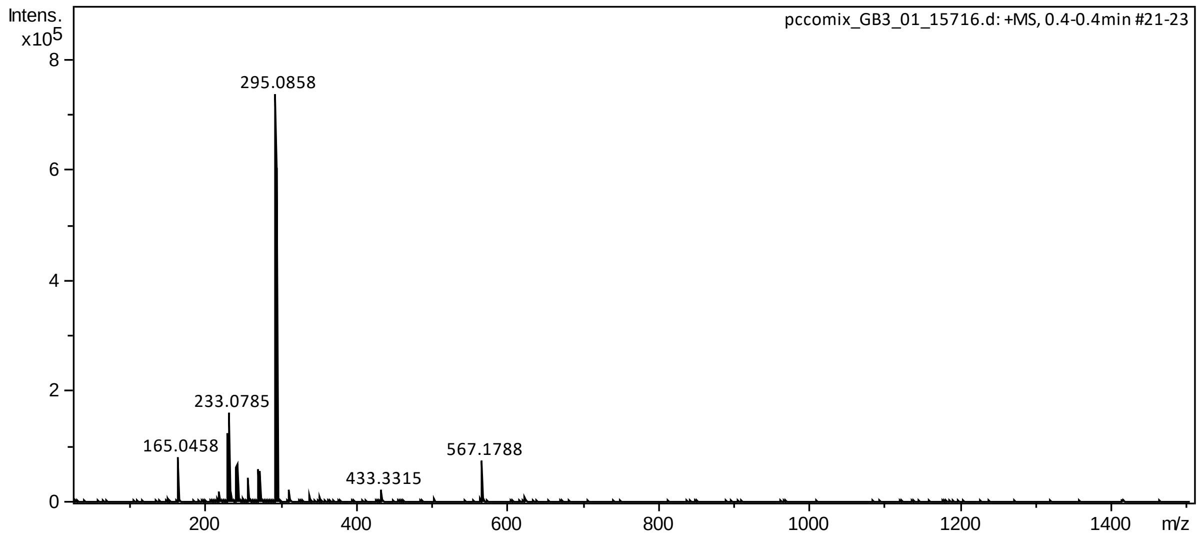

Figure S91. Compound **6b**, HRMS (ESI+) calc for $[C_{15}H_{13}FN_2O_2+Na]^+$: 295.0853 found 295.0858 $[M+Na]^+$.

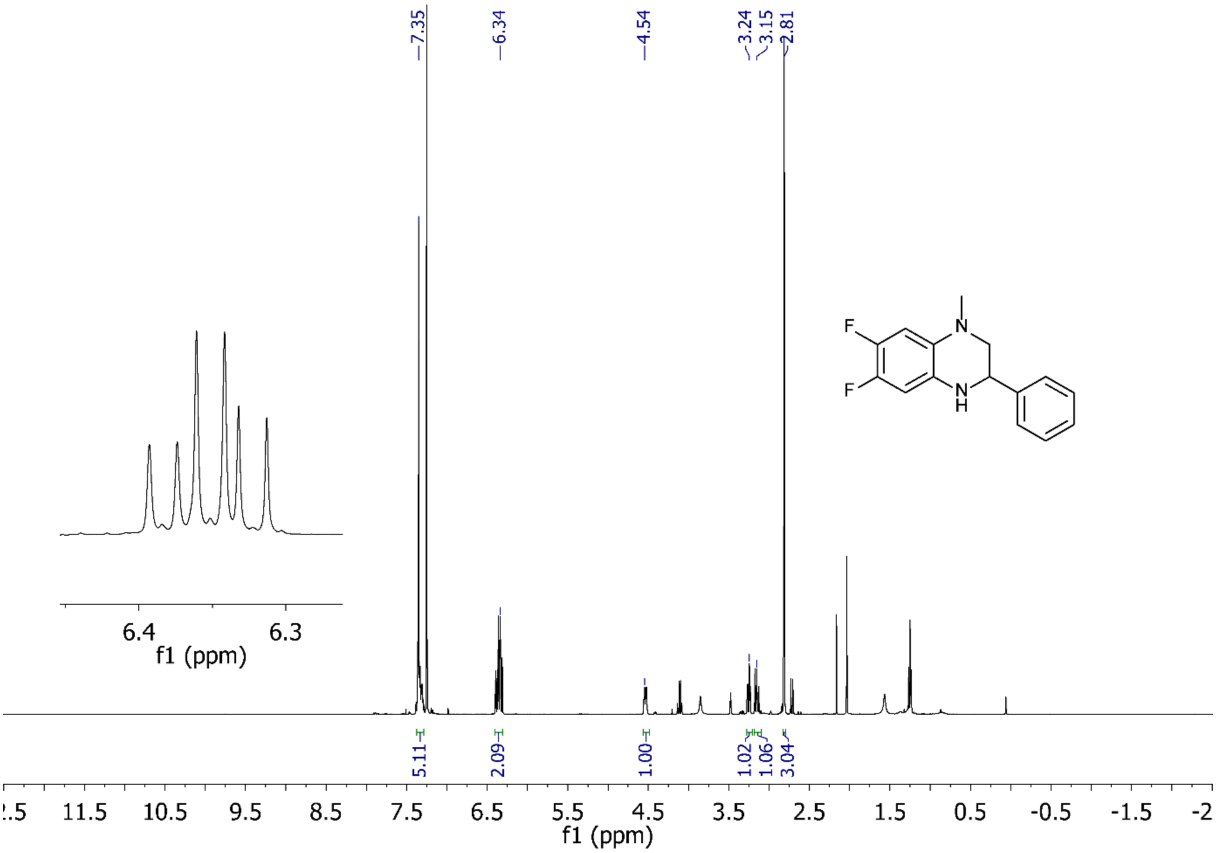

Figure S92. Compound **7a**, ^{1}H NMR (400 MHz, $CDCl_3$).

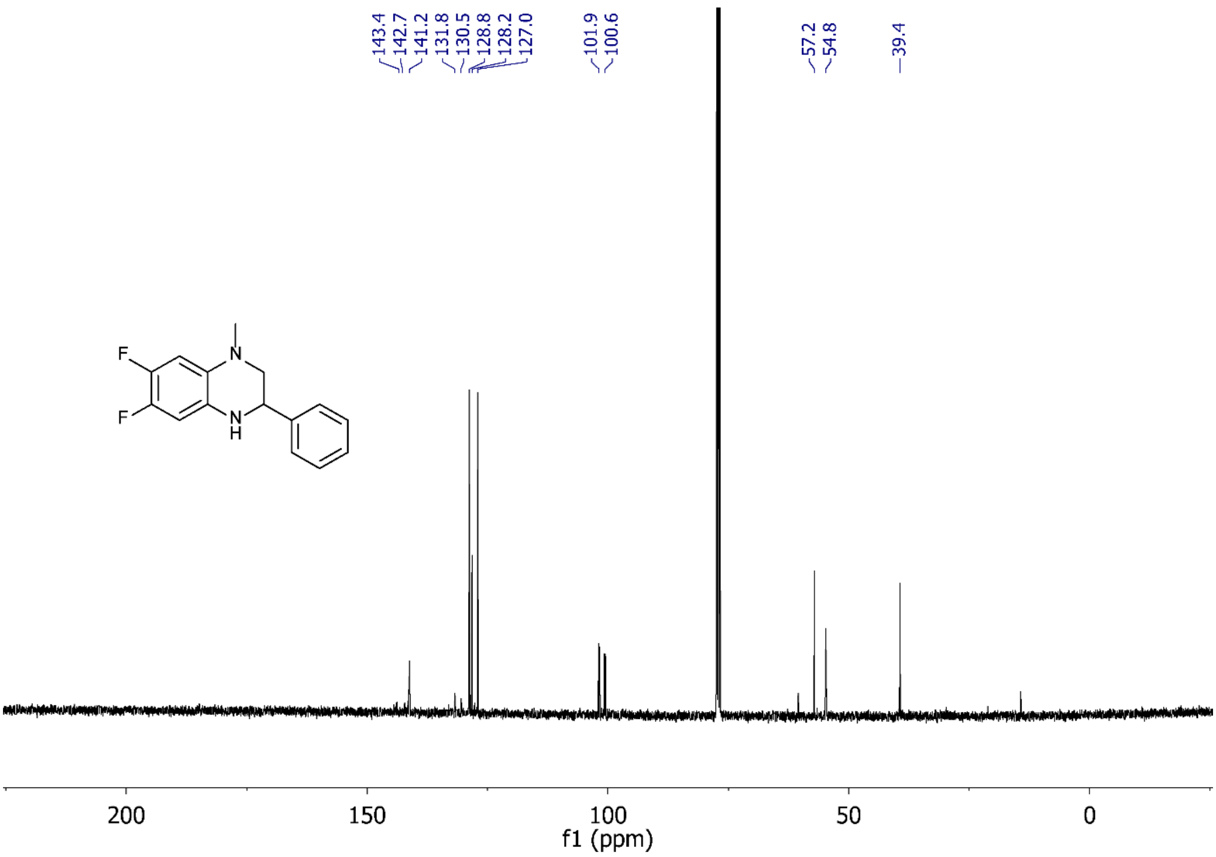

Figure S93. Compound **7a**, ^{13}C NMR (101 MHz, $CDCl_3$).

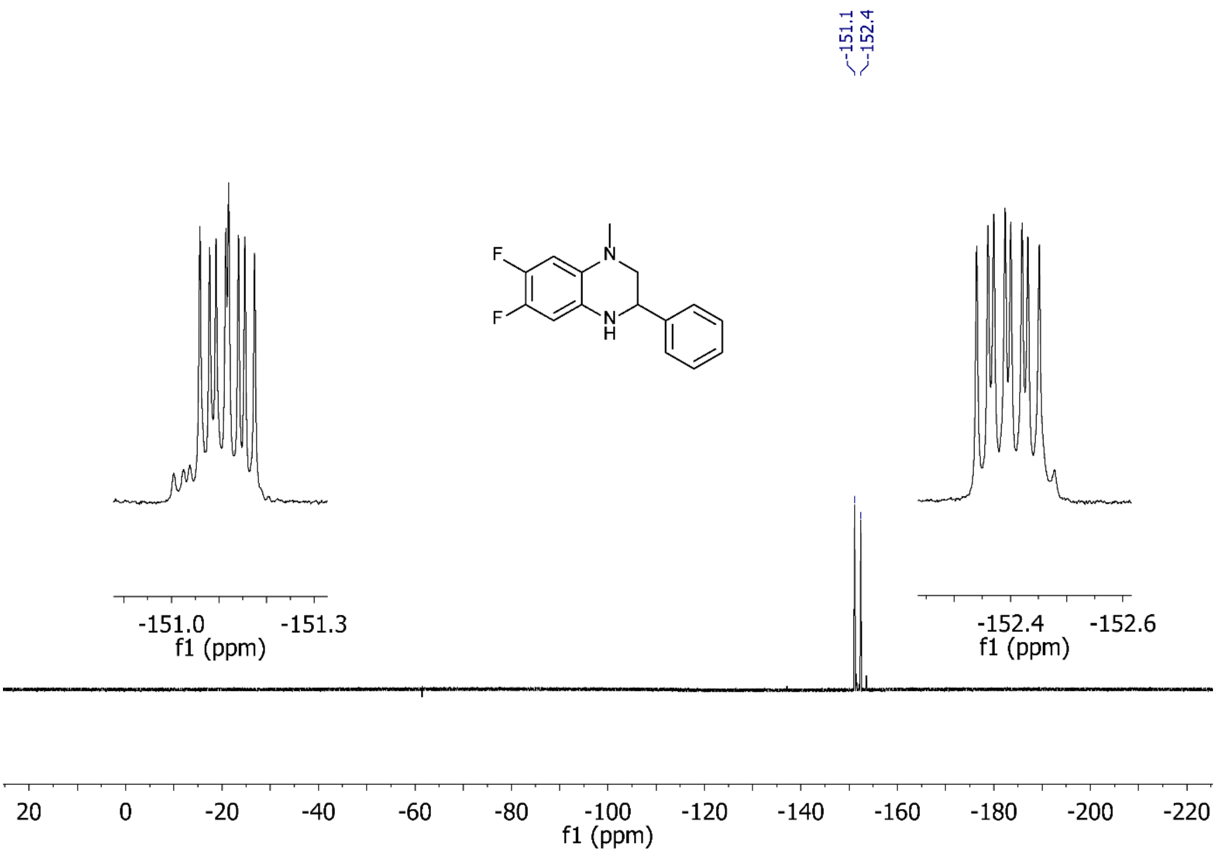

Figure S94. Compound **7a**, ^{19}F NMR (376.5 MHz, $CDCl_3$).

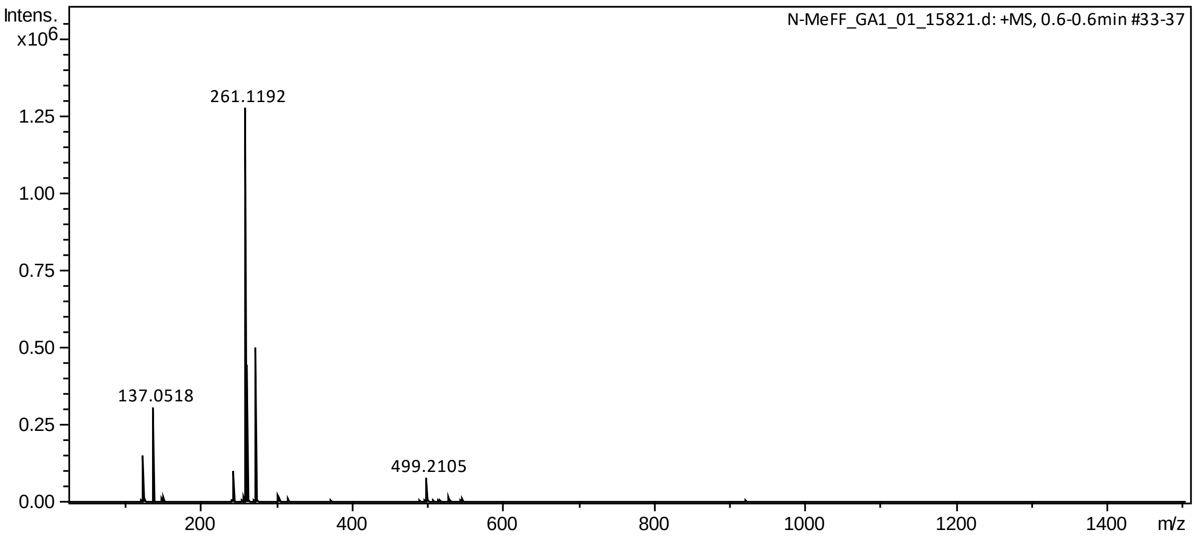

Figure S95. Compound **7a**, HRMS (ESI+) calc for $[C_{15}H_{14}F_2N_2+H]^+$: 261.1198 found 261.1192 $[M+H]^+$.

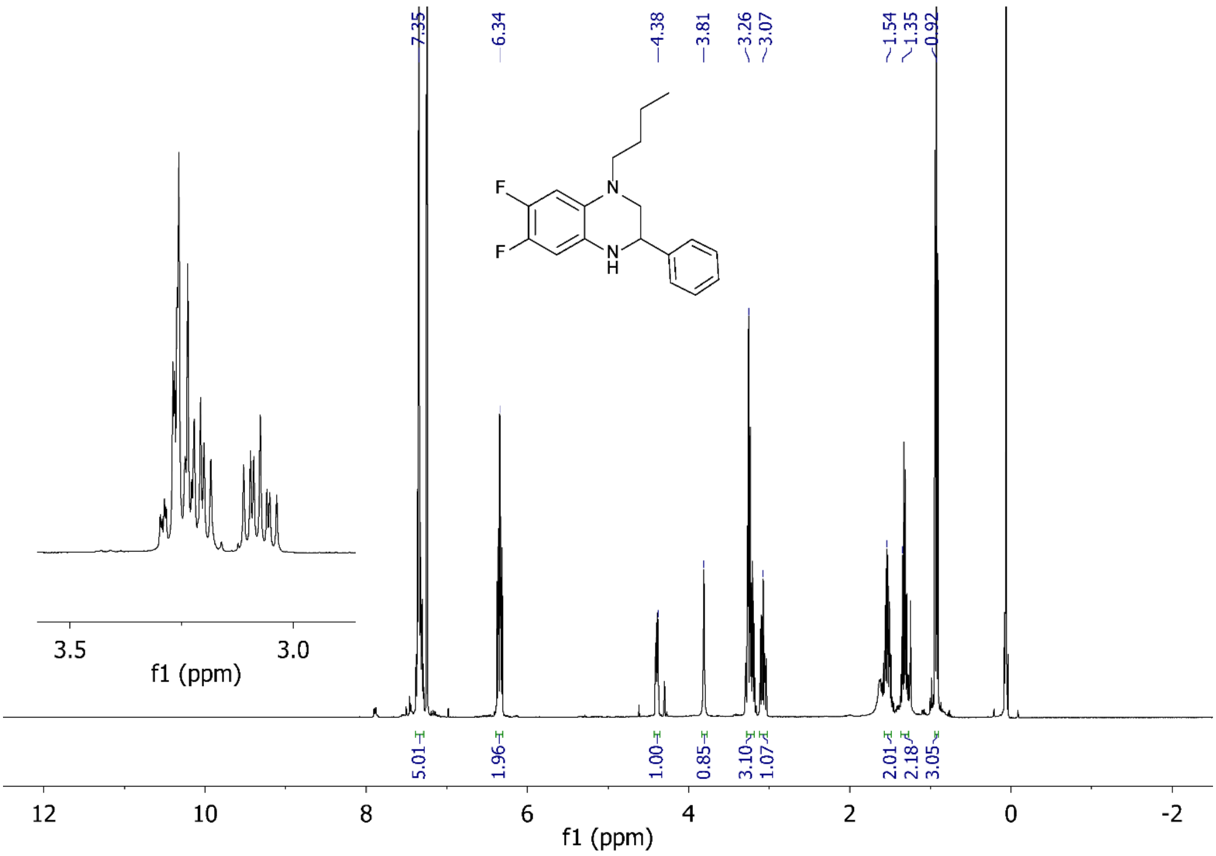

Figure S96. Compound **7b**, ^1H NMR (400 MHz, $CDCl_3$).

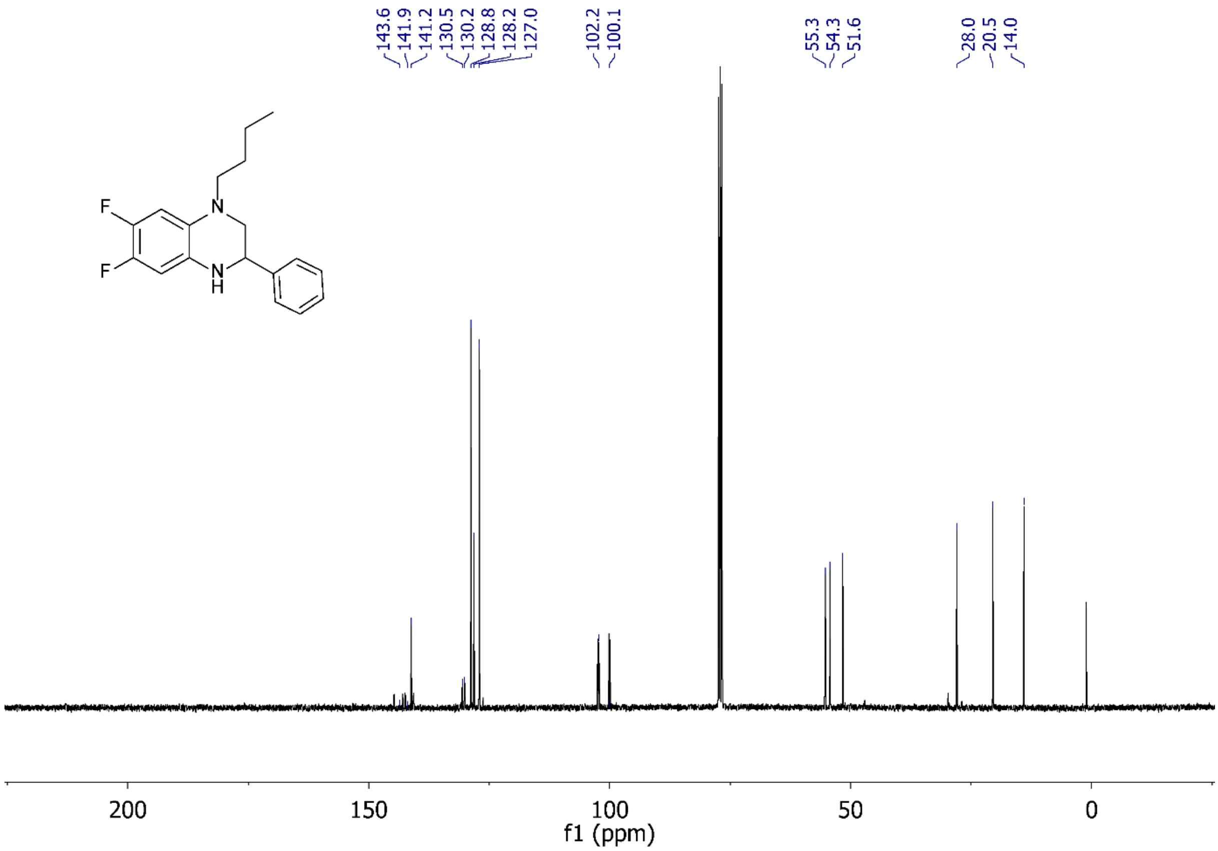

Figure S97. Compound **7b**, ^{13}C NMR (101 MHz, $CDCl_3$).

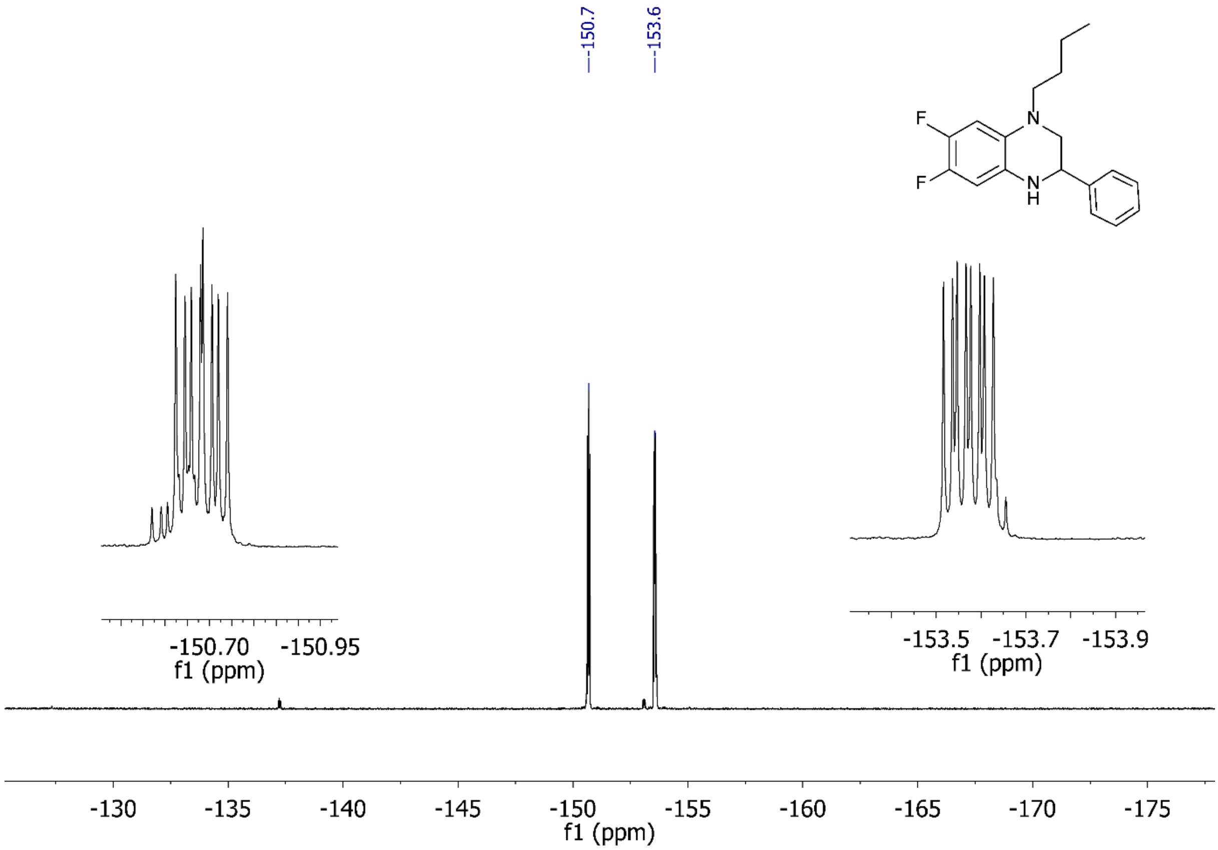

Figure S98. Compound **7b**, ^{19}F NMR (376.5 MHz, $CDCl_3$).

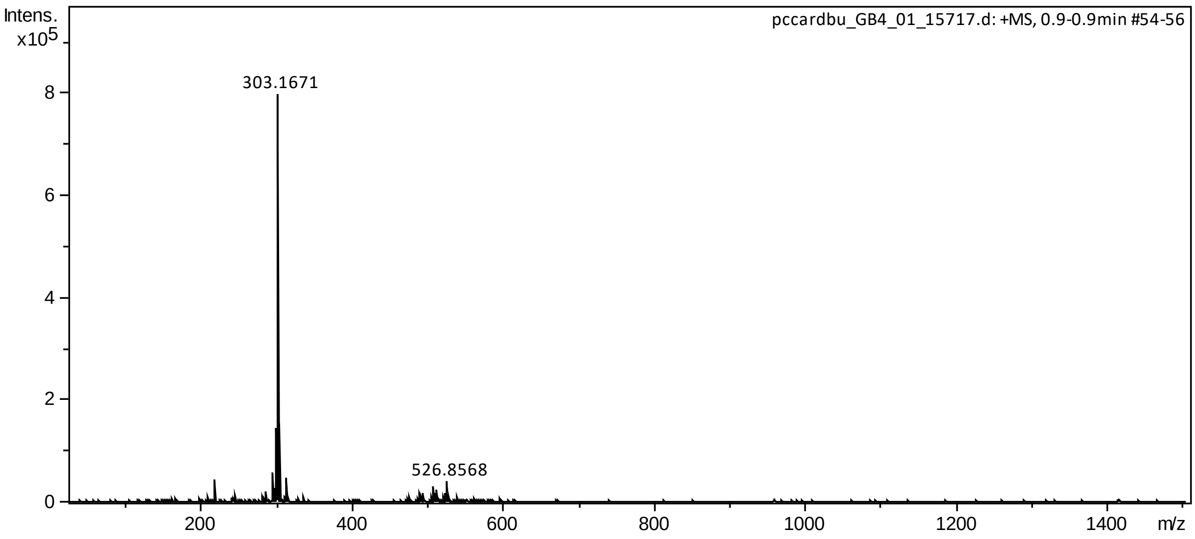

Figure S99. Compound **7b**, HRMS (ESI+) calc for $[C_{18}H_{20}F_2N_2+H]^+$: 303.1667 found 303.1671 $[M+H]^+$.

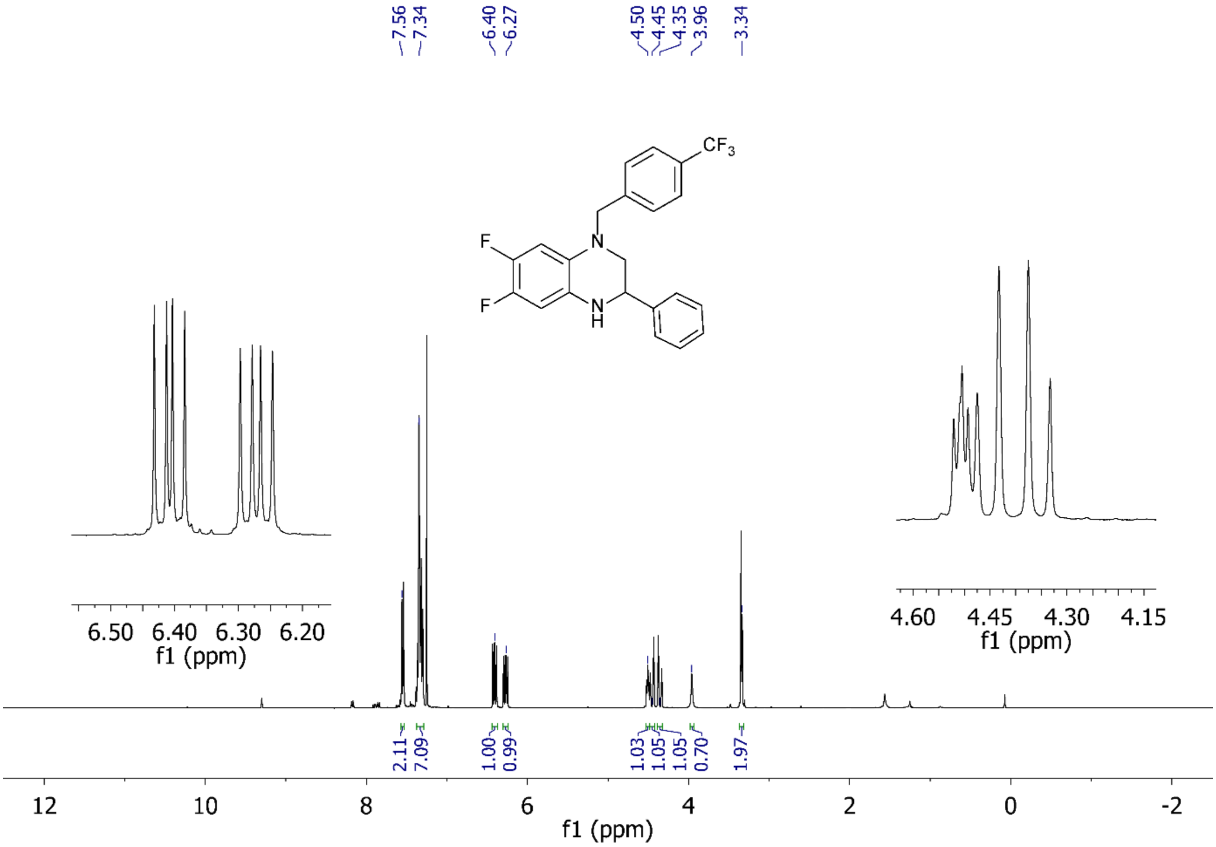

Figure S100. Compound **7c**, ^{1}H NMR (400 MHz, $CDCl_3$).

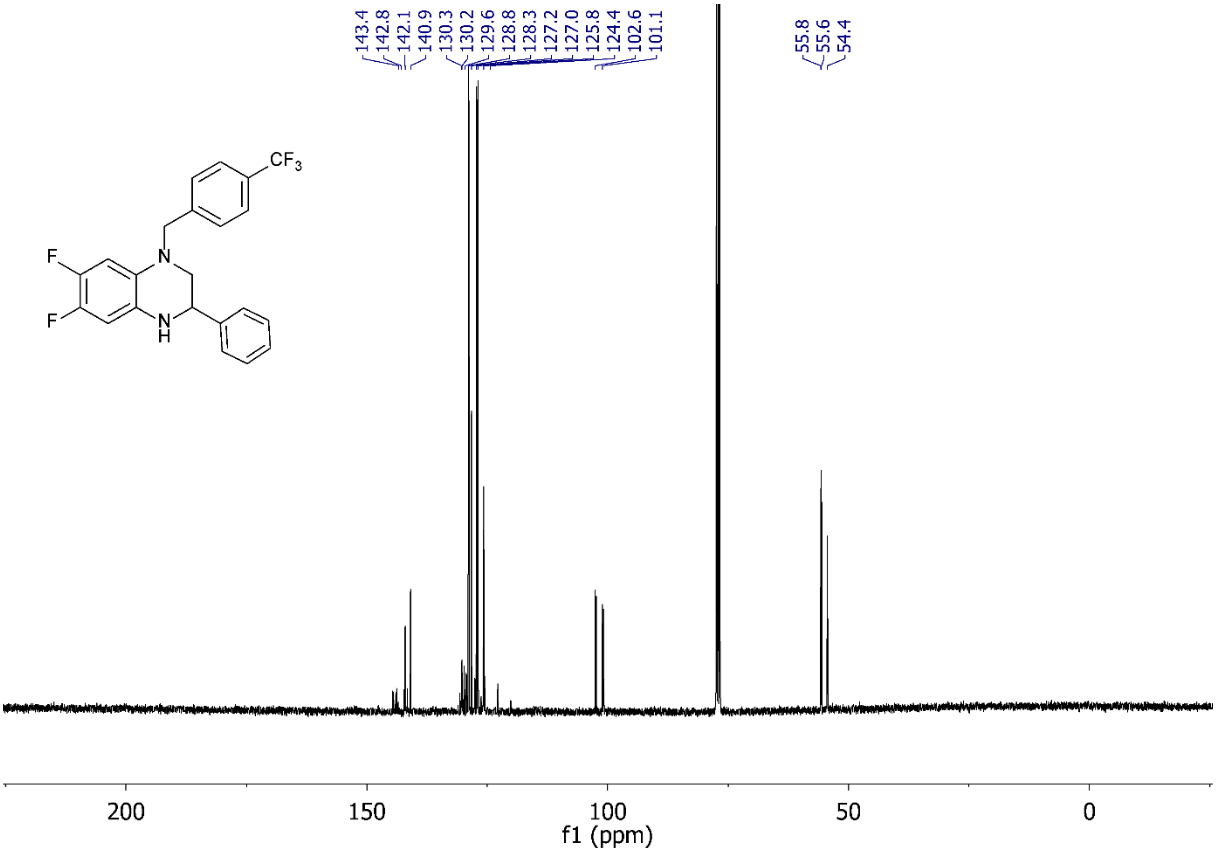

Figure S101. Compound **7c**, ^{13}C NMR (101 MHz, $CDCl_3$).

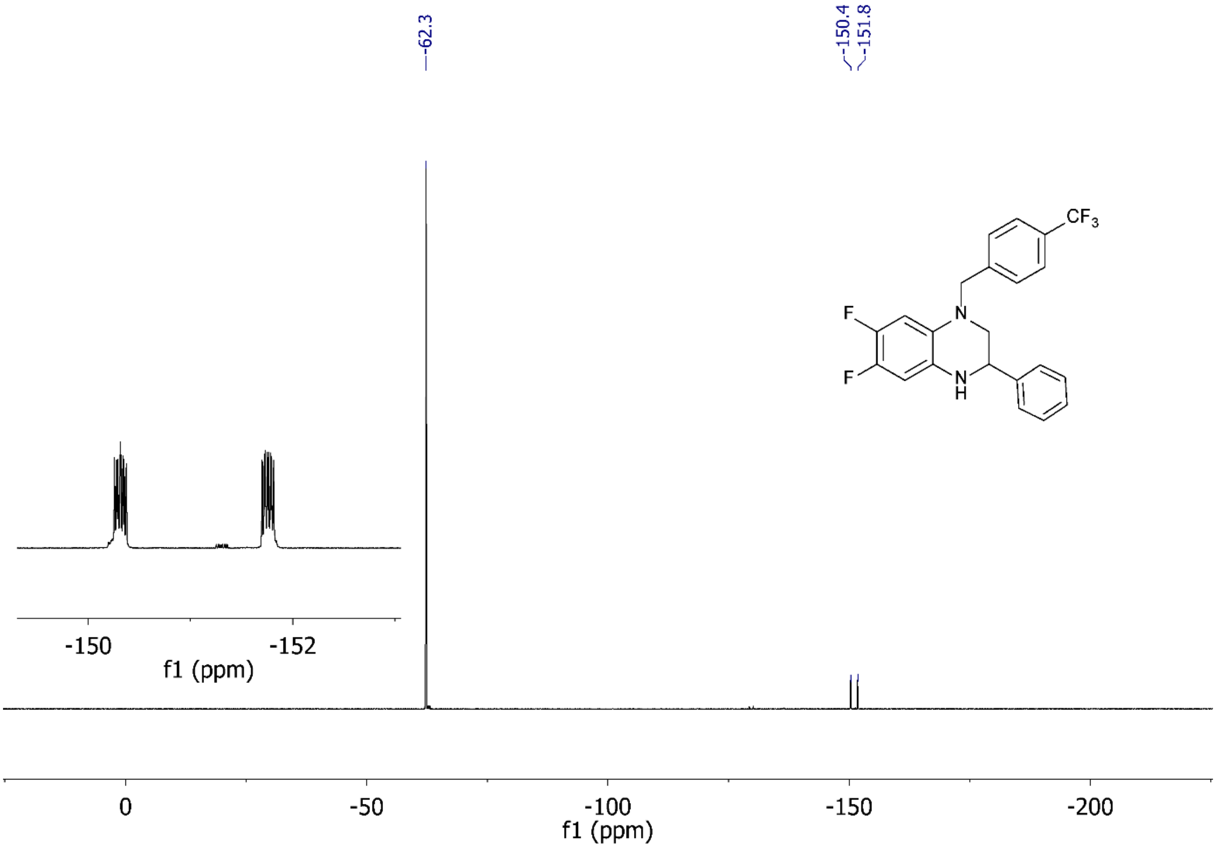

Figure S102. Compound **7c**, ^{19}F NMR (376.5 MHz, $CDCl_3$).

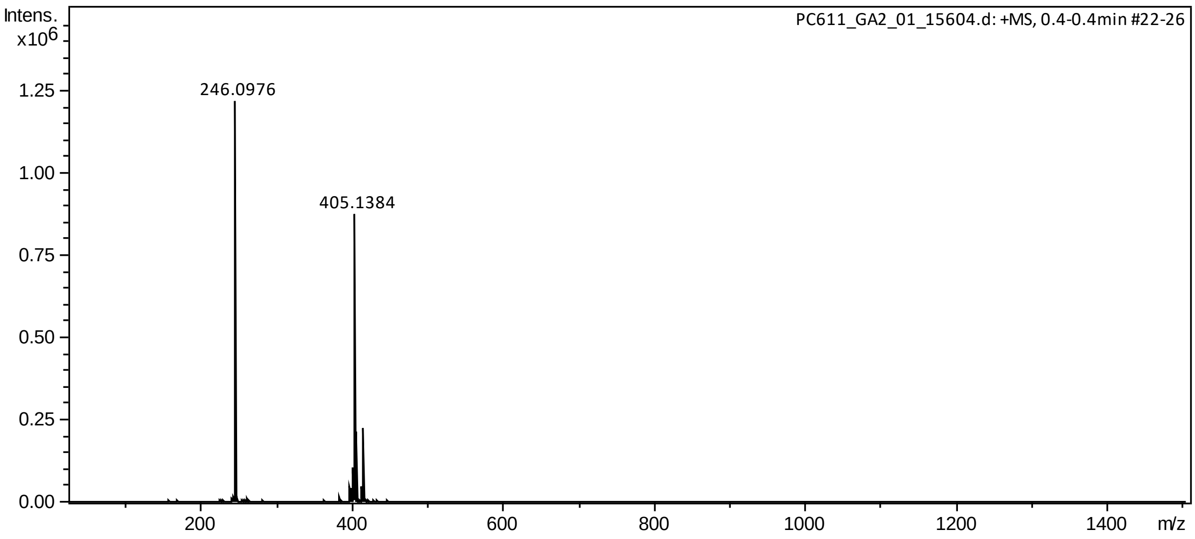

Figure S103. Compound **7c**, HRMS (ESI+) calc for $[C_{22}H_{17}F_5N_2+H]^+$: 405.1383 found 405.1384 $[M+H]^+$.

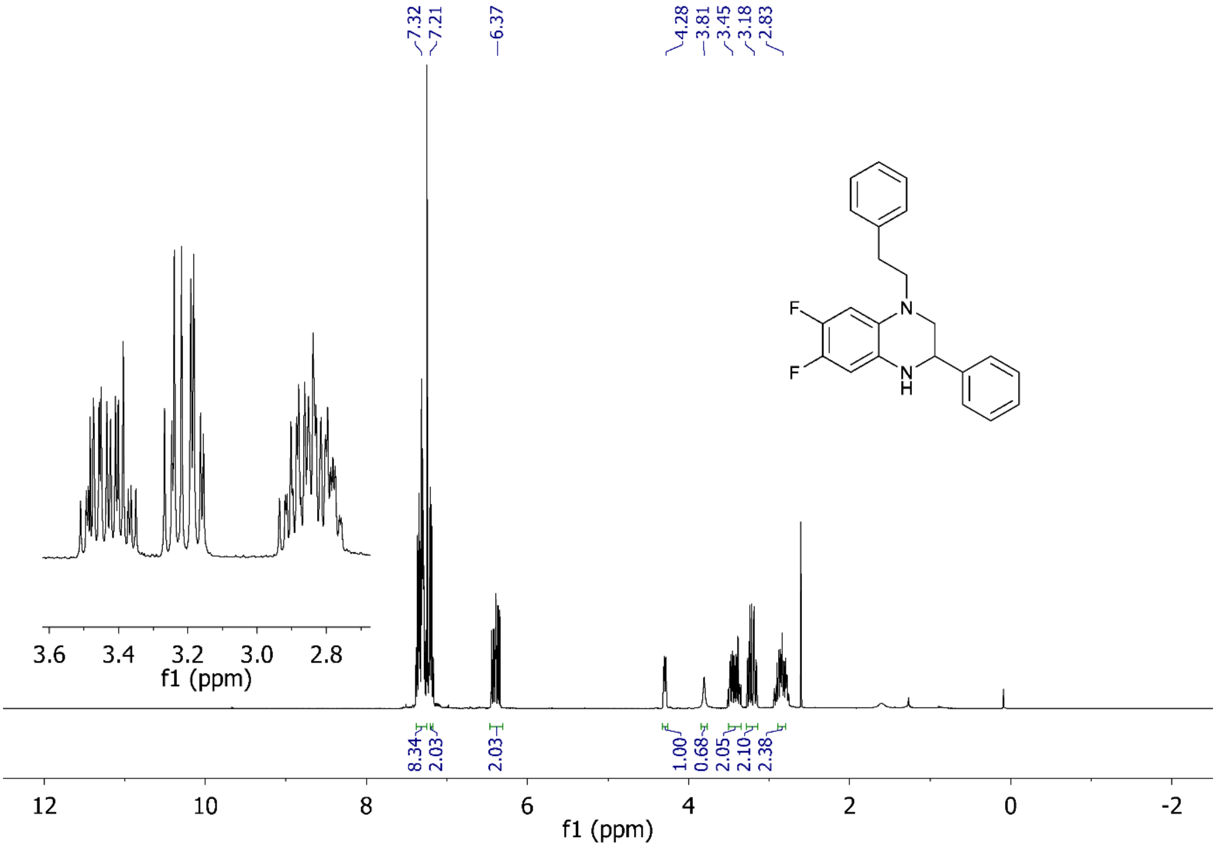

Figure S104. Compound **7d**, ^{1}H NMR (400 MHz, $CDCl_3$).

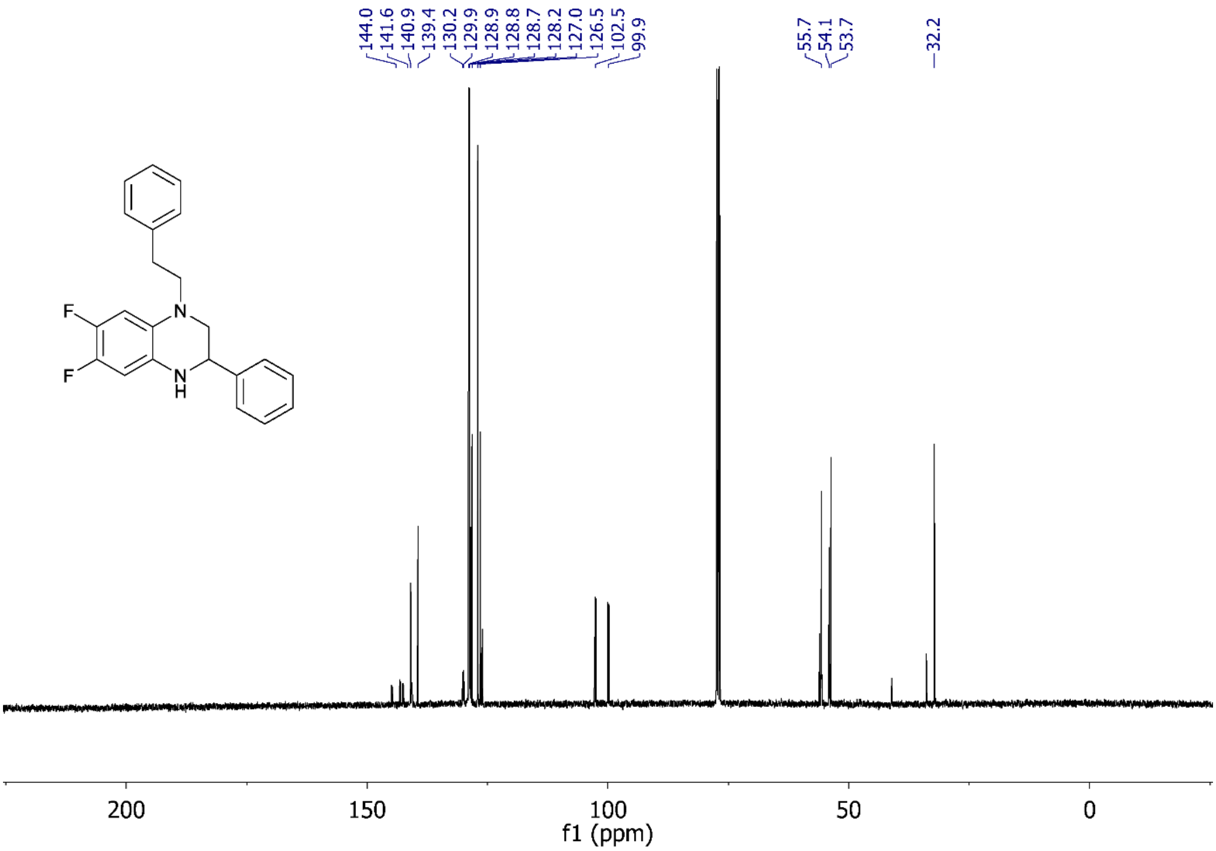

Figure S105. Compound **7d**, ^{13}C NMR (101 MHz, $CDCl_3$).

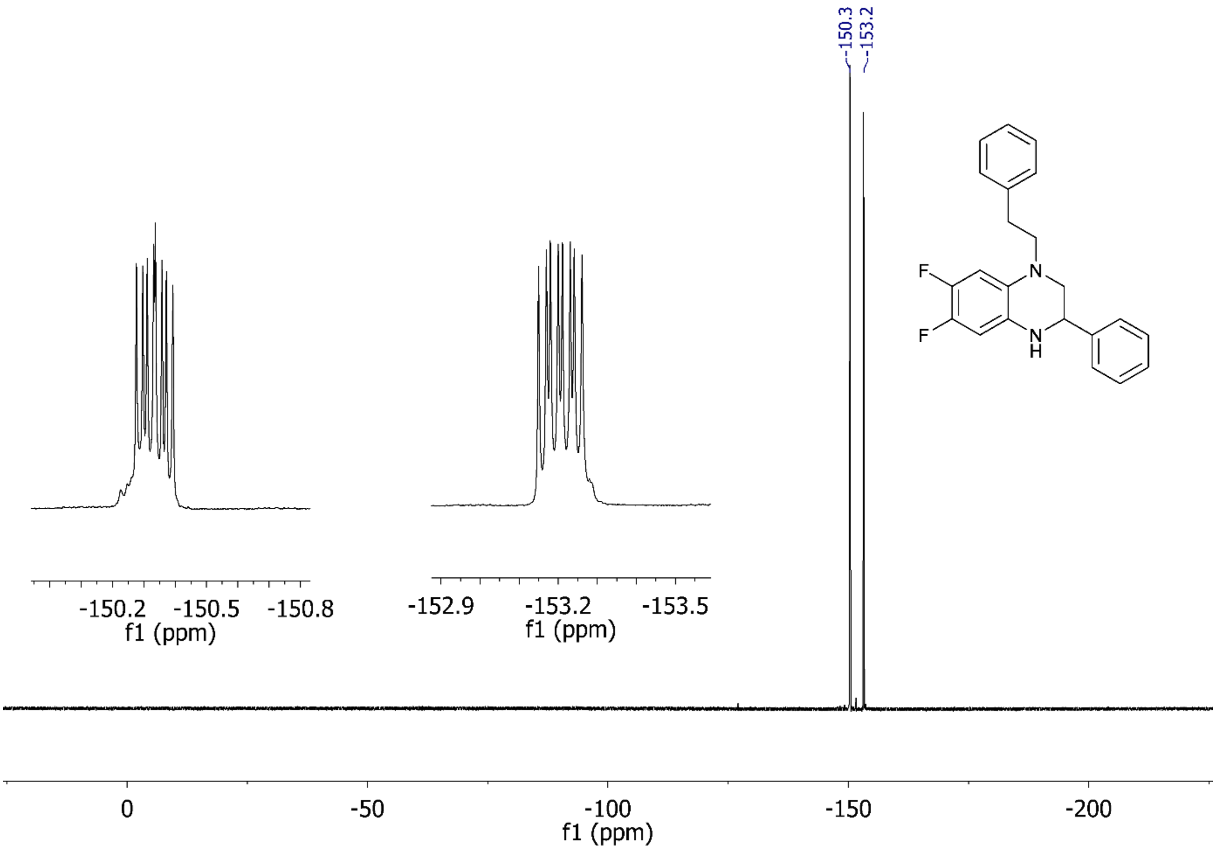

Figure S106. Compound **7d**, ^{19}F NMR (376.5 MHz, $CDCl_3$).

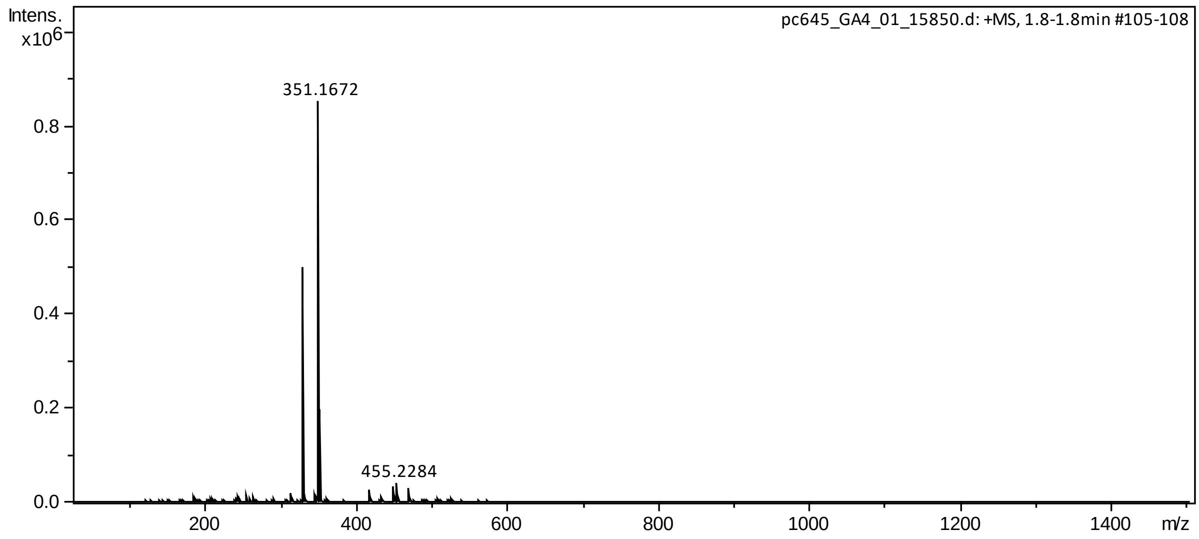

Figure S107. Compound **7d**, HRMS (ESI+) calc for $[C_{22}H_{20}N_2F_2]^+$: 351.1668 found 351.1672 $[M+H]^+$.

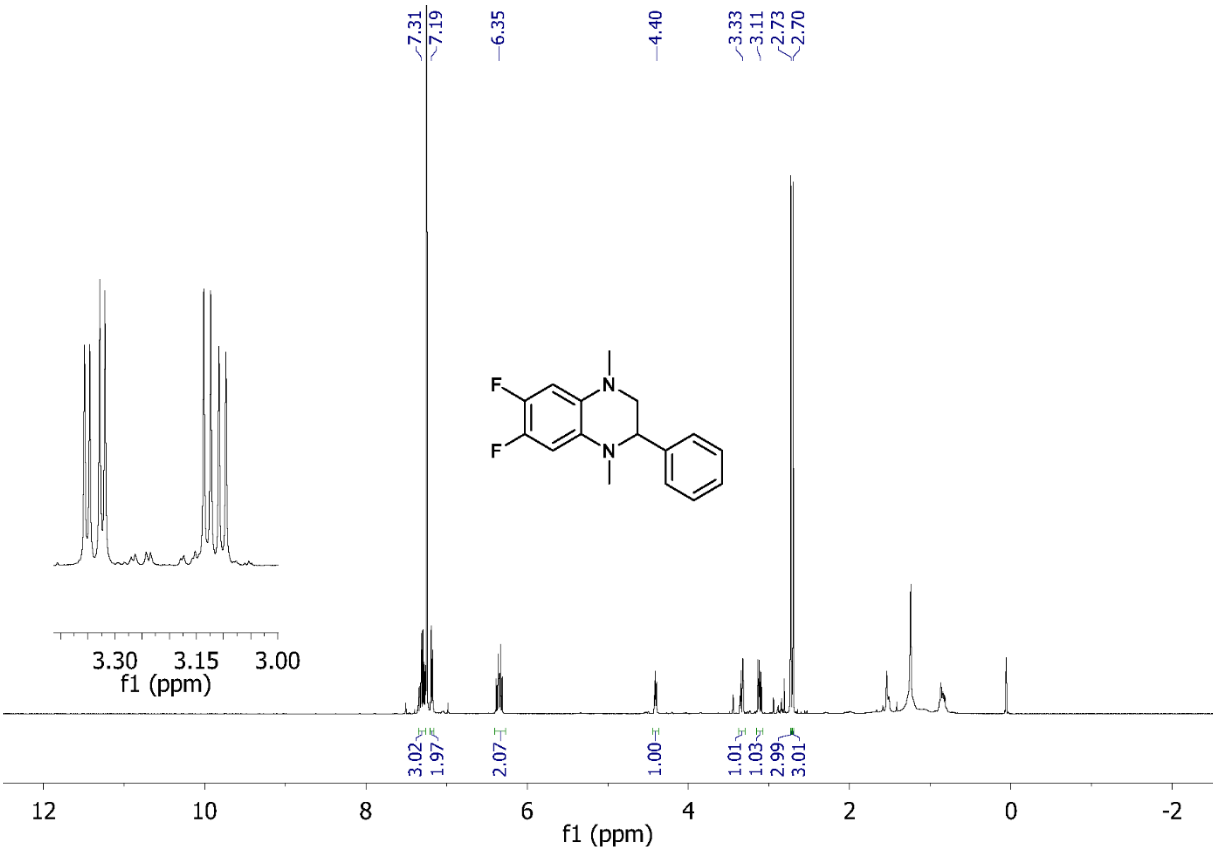

Figure S108. Compound **7e**, ^{1}H NMR (400 MHz, $CDCl_3$).

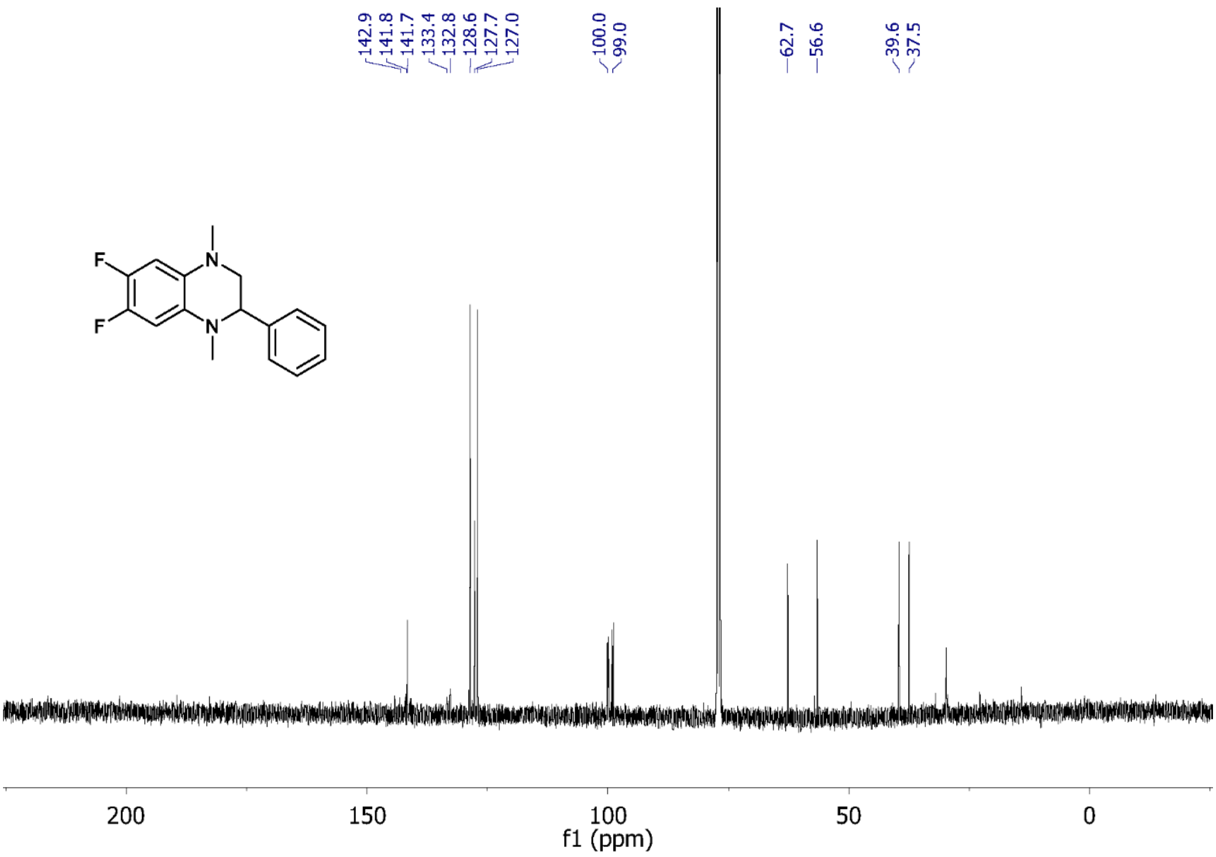

Figure S109. Compound **7e**, ^{13}C NMR (101 MHz, $CDCl_3$).

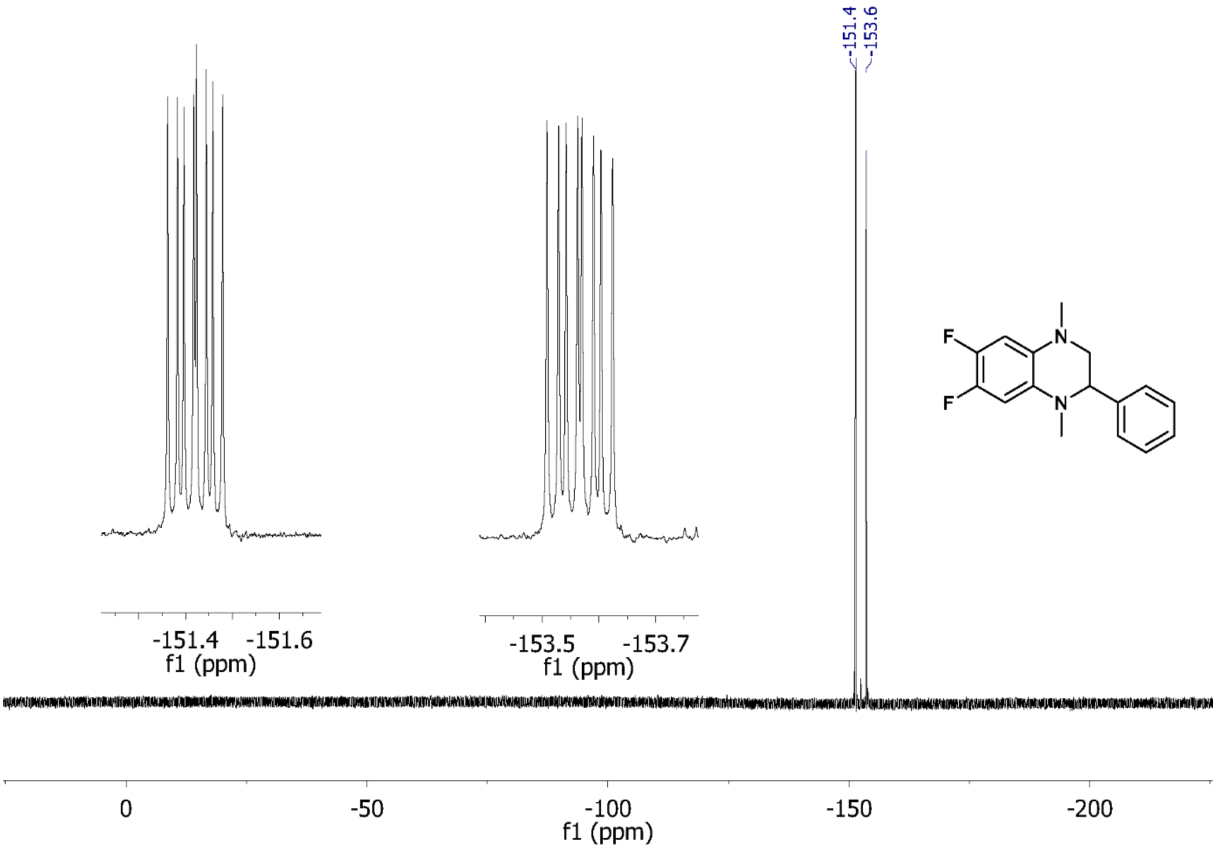

Figure S110. Compound **7e**, ^{19}F NMR (376.5 MHz, $CDCl_3$).

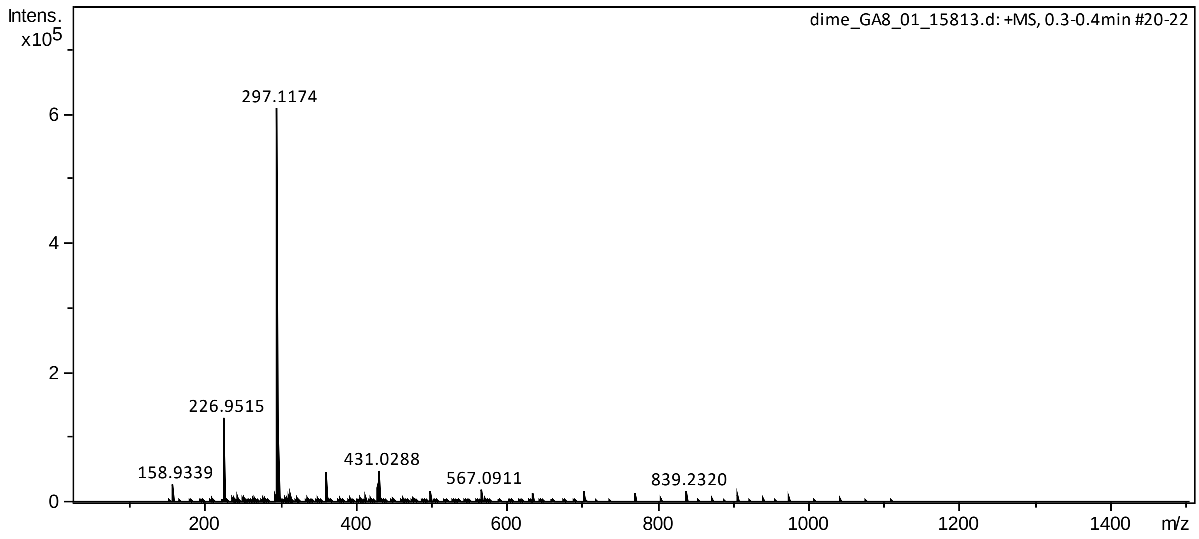

Figure S111. Compound **7e**, HRMS (ESI+) calc for $[C_{16}H_{16}F_2N_2+Na]^+$: 297.1173, found 297.1174 $[M+Na]^+$.

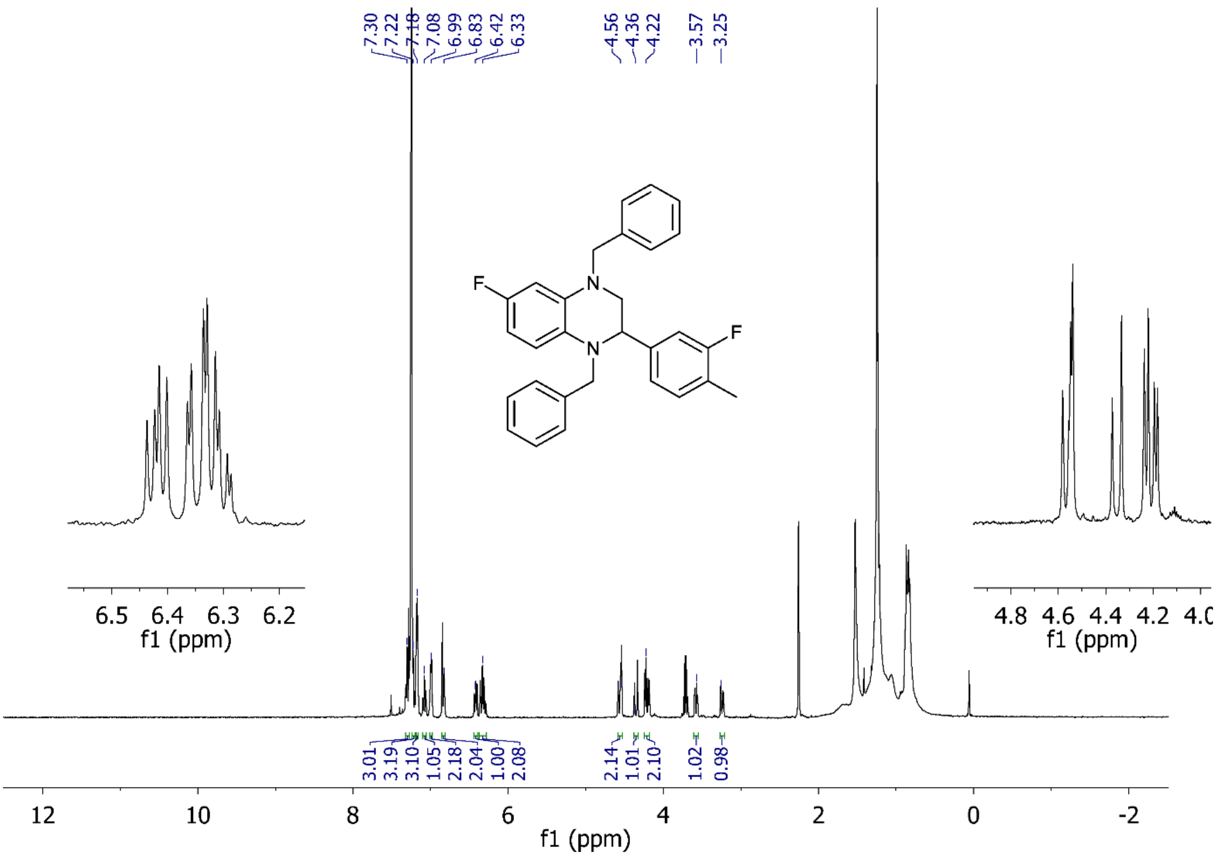

Figure S112. Compound **8a**, ^1H NMR (400 MHz, $CDCl_3$).

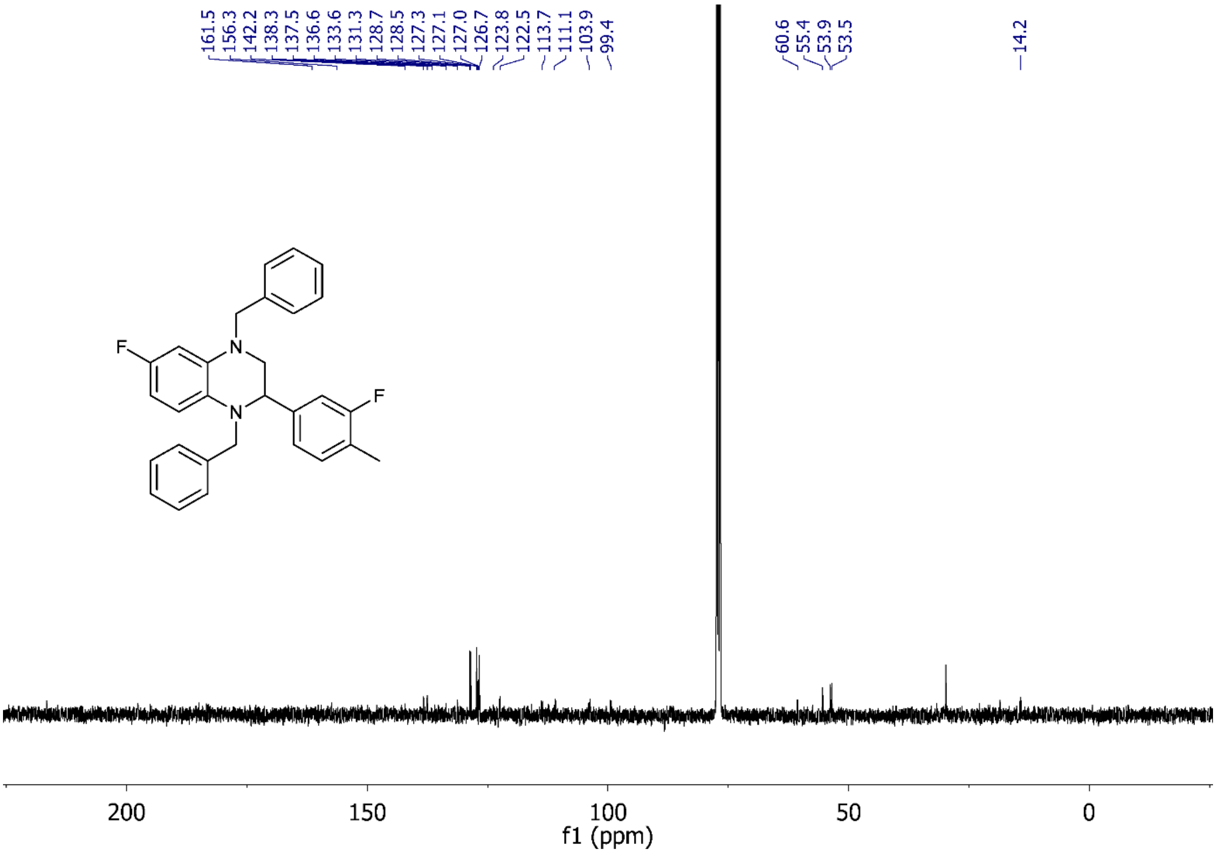

Figure S113. Compound **8a**, ^{13}C NMR (101 MHz, $CDCl_3$), ^{13}C shifts confirmed by HSQC.

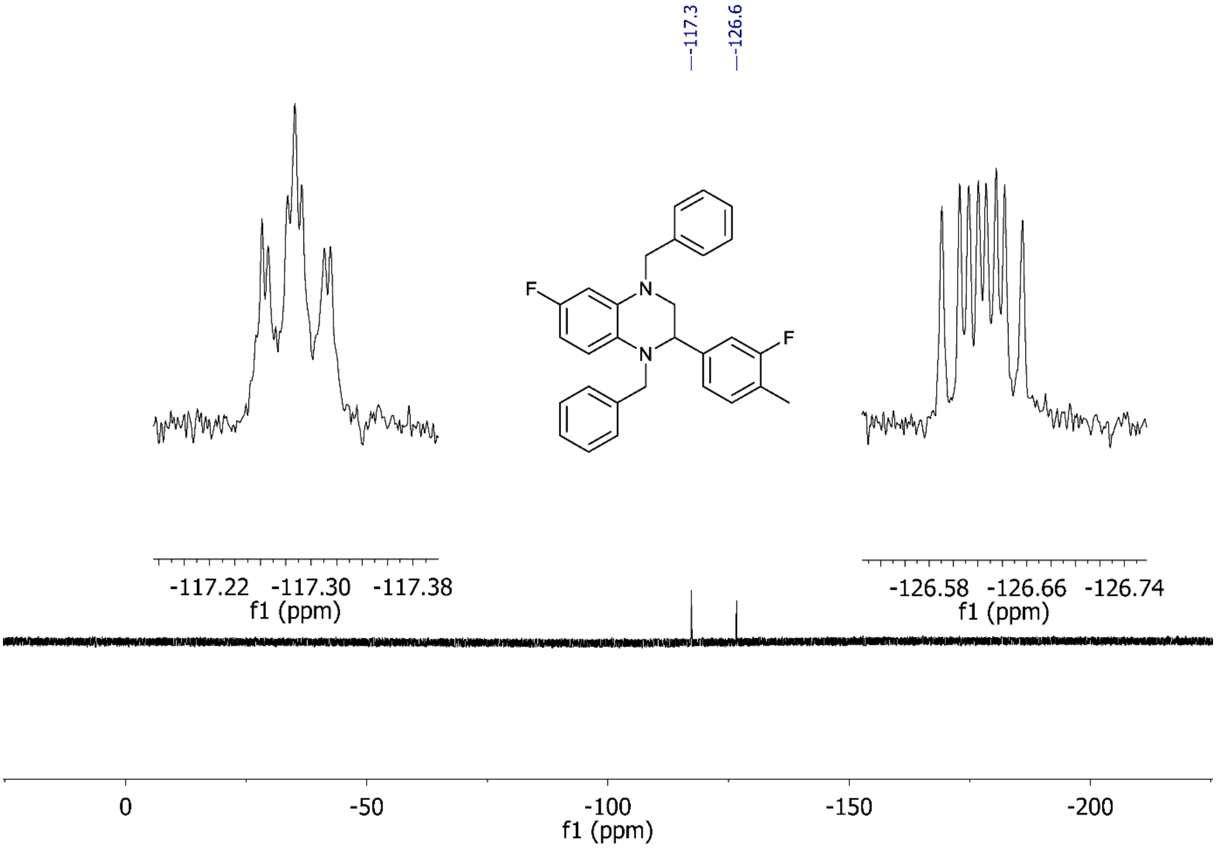

Figure S114. Compound **8a**, ^{19}F NMR (376.5 MHz, $CDCl_3$).

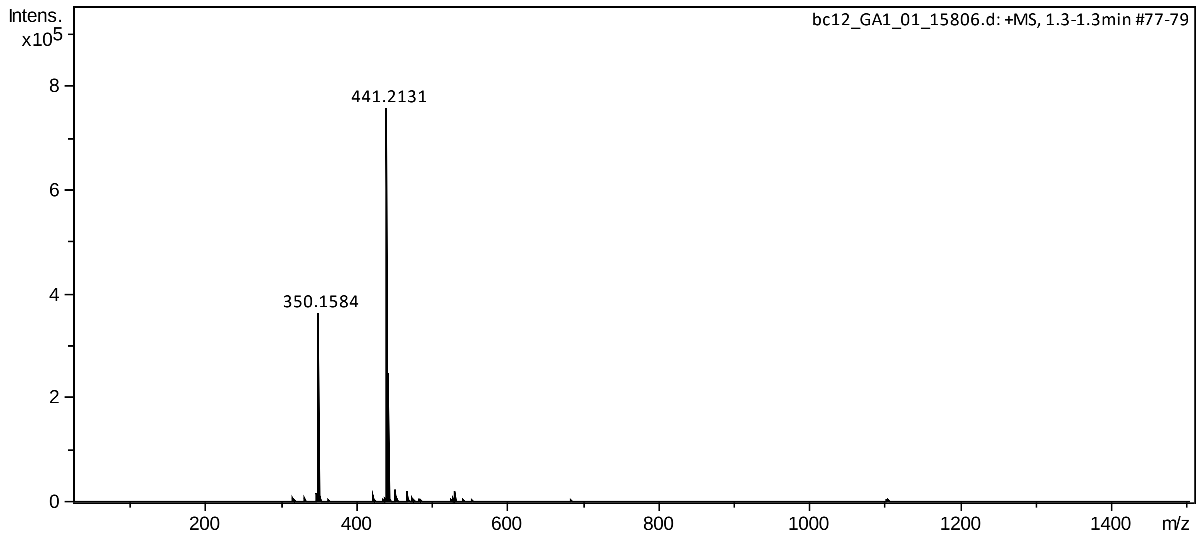

Figure S115. Compound **8a**, HRMS (ESI+) calc for $[C_{29}H_{26}F_2N_2+H]^+$: 441.2137 found 441.2131 $[M+H]^+$.

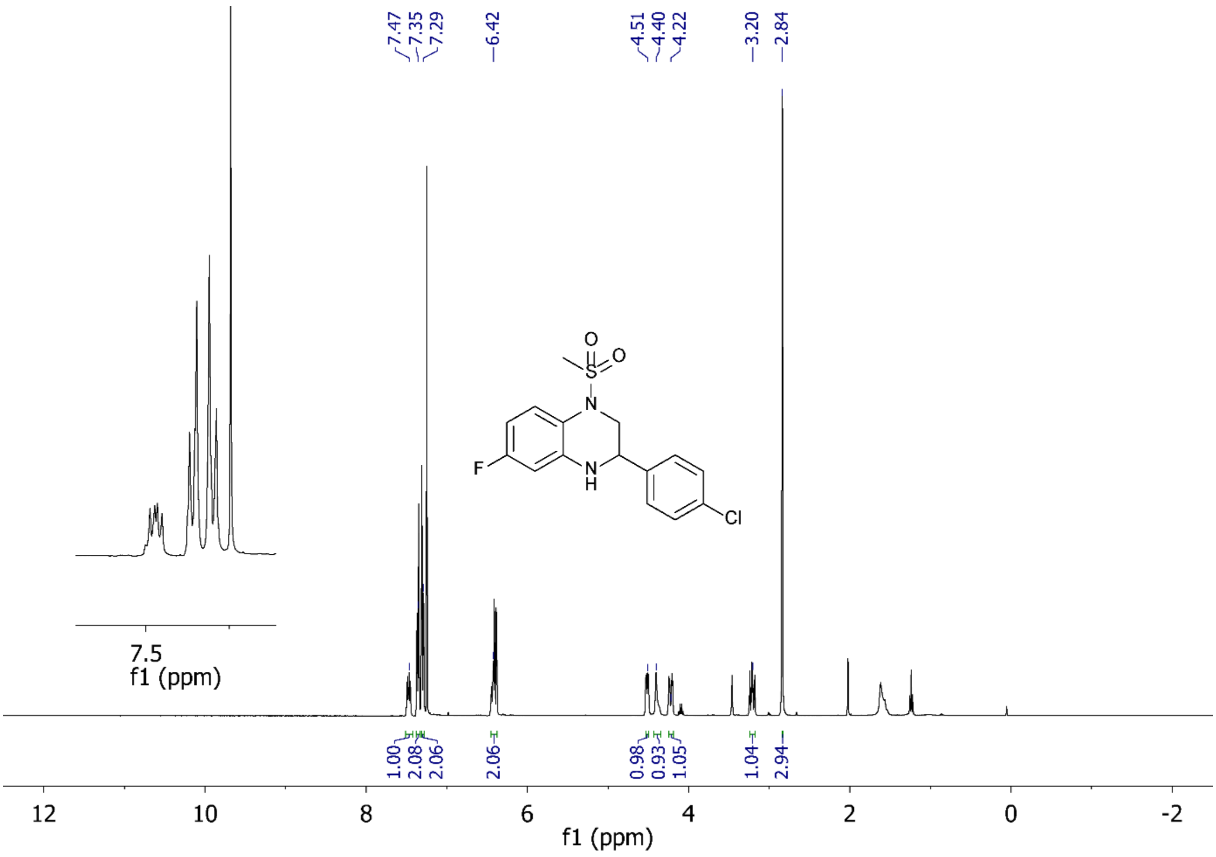

Figure S116. Compound **9a**, ^{1}H NMR (400 MHz, $CDCl_3$).

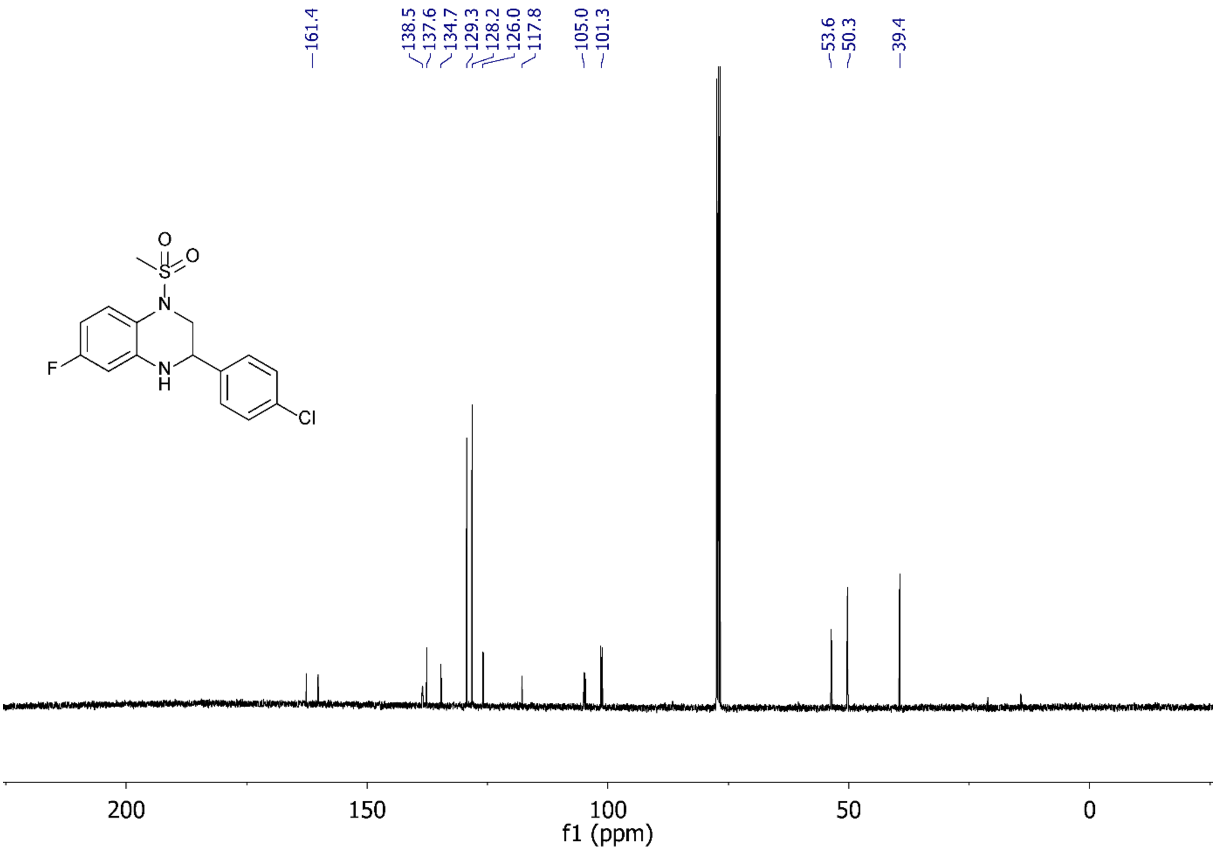

Figure S117. Compound **7a**, ^{13}C NMR (101 MHz, $CDCl_3$).

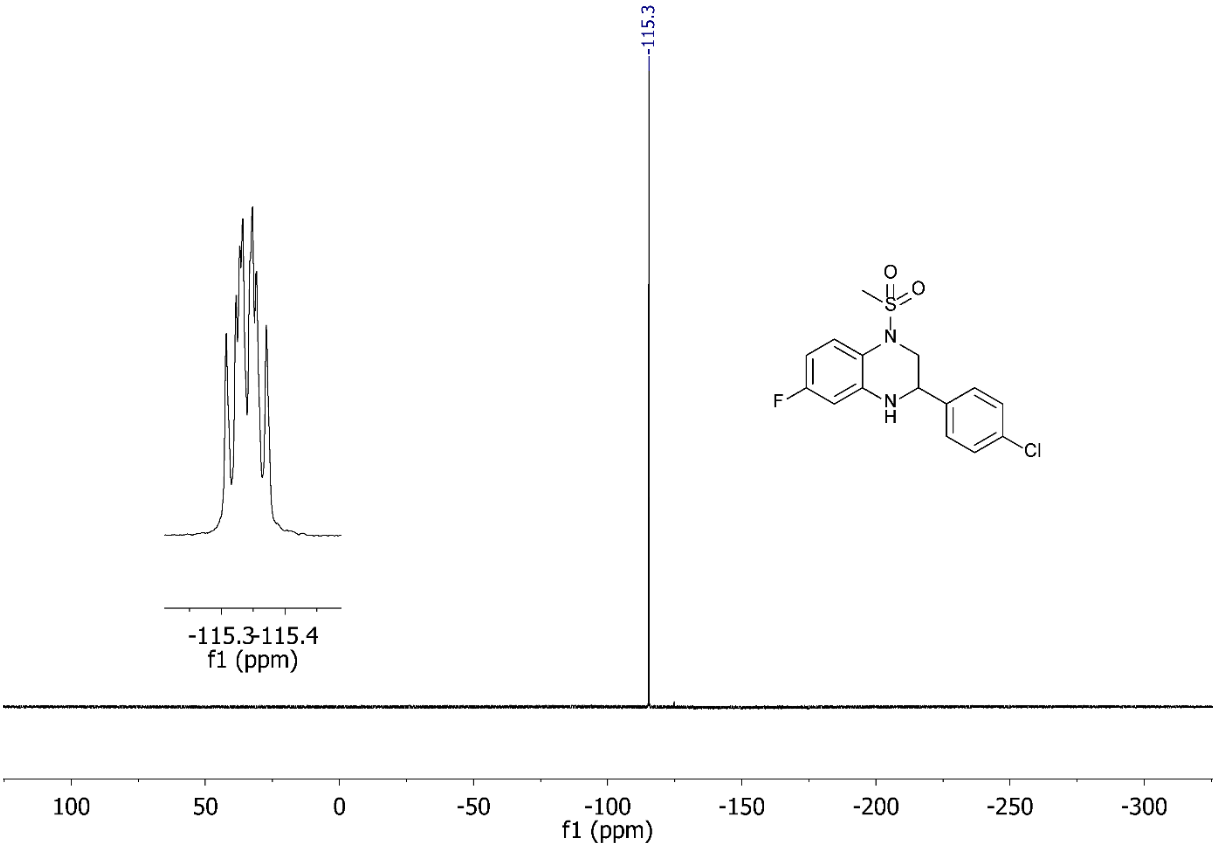

Figure S118. Compound **9a**, ^{19}F NMR (376.5 MHz, $CDCl_3$).

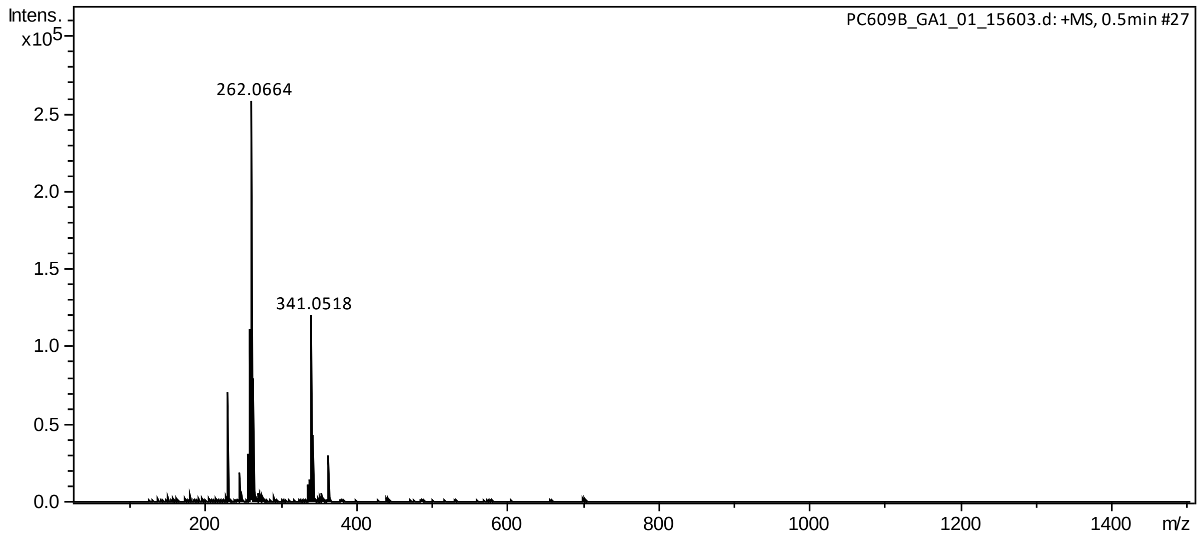

Figure S119. Compound **9a**, HRMS (ESII+) calcd for $[C_{15}H_{14}ClFN_2O_2S+H]^+$: 341.521 found 341.518 $[M+H]^+$

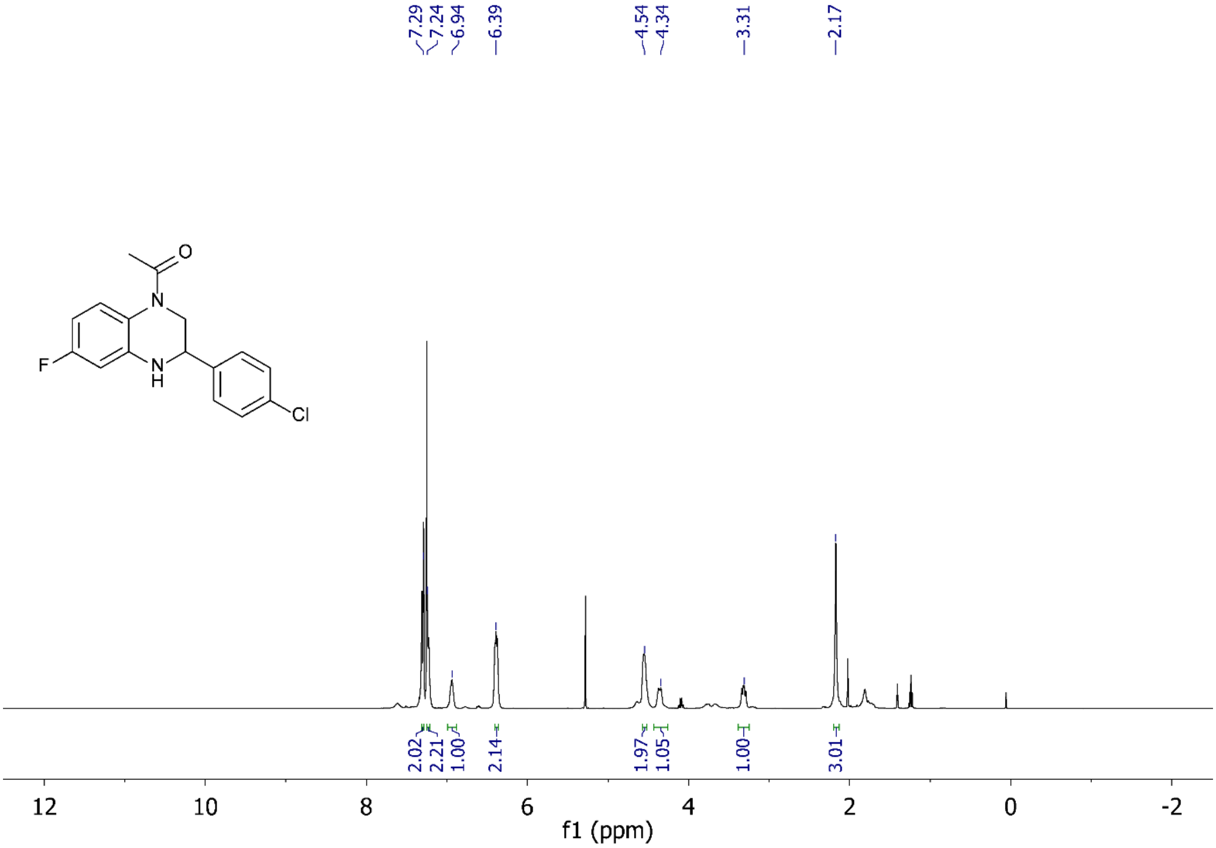

Figure S120. Compound **9b**, ^{1}H NMR (400 MHz, $CDCl_3$).

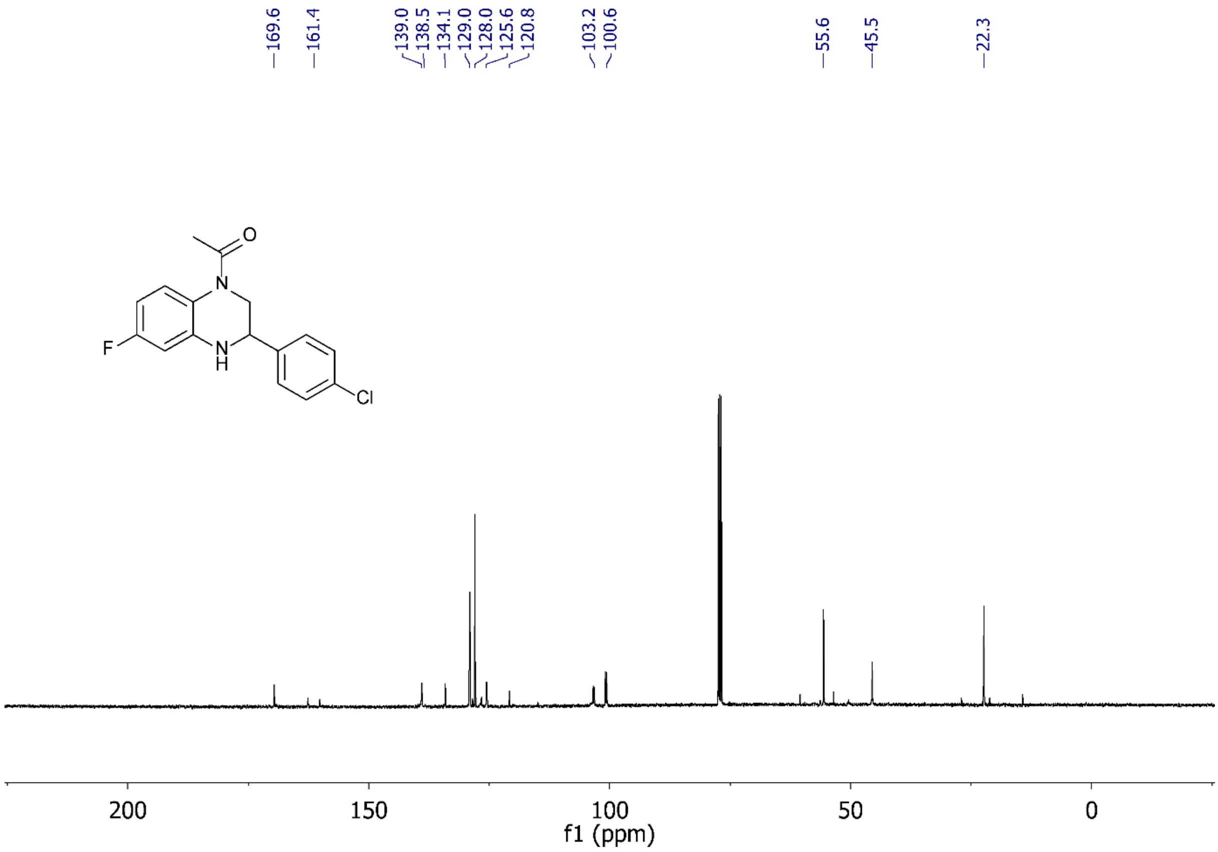

Figure S121. Compound **9b**, ^{13}C NMR (101 MHz, $CDCl_3$).

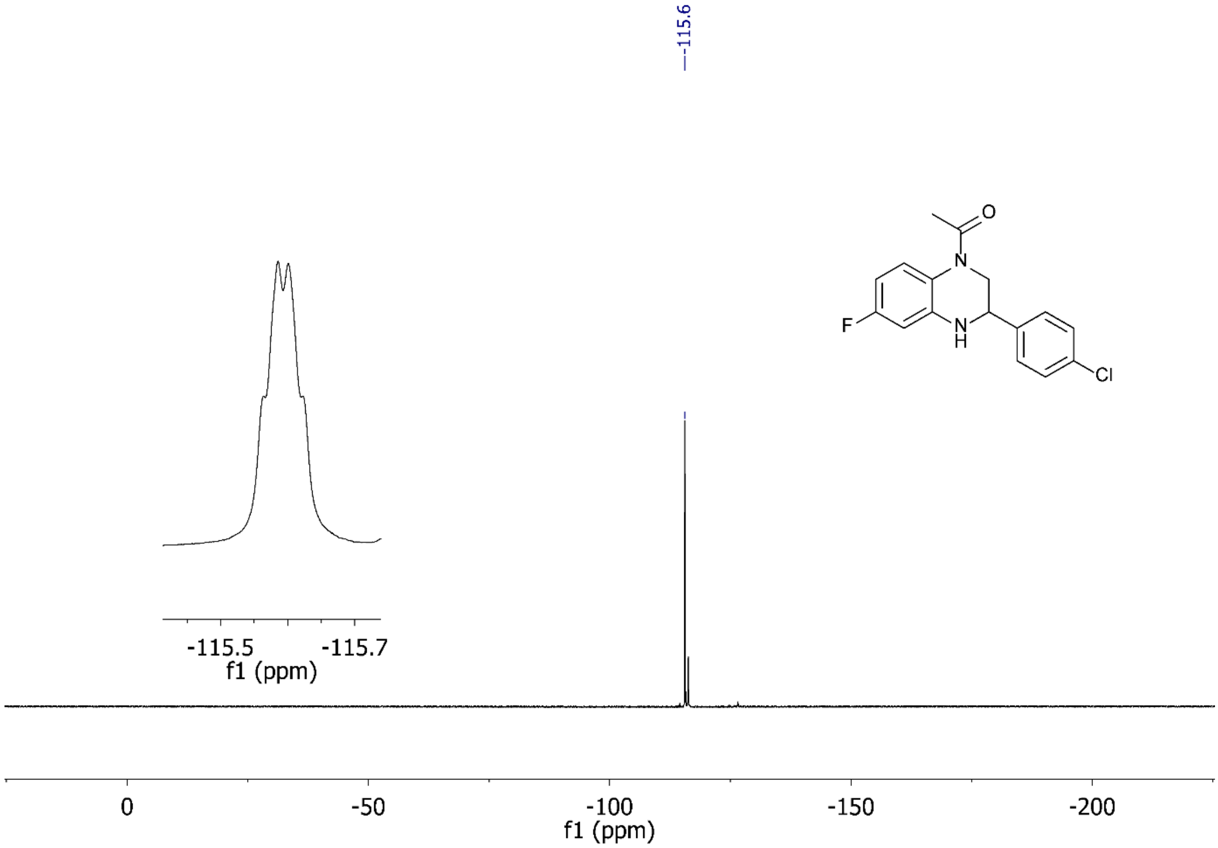

Figure S122. Compound **9b**, ^{19}F NMR (376.5 MHz, $CDCl_3$).

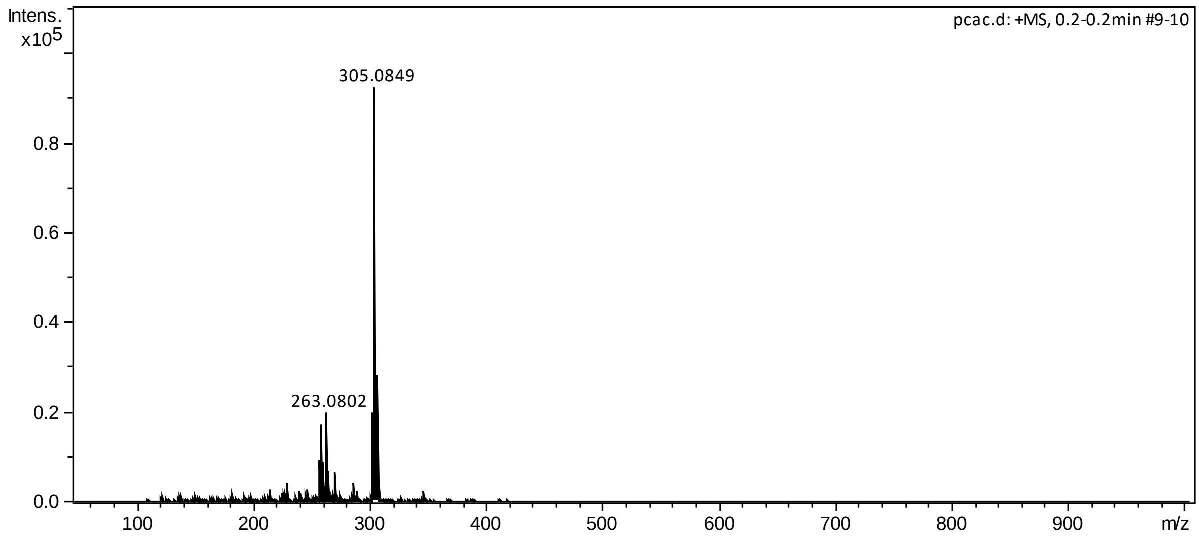

Figure S123. Compound **9b**, HRMS (APCI+) calc for $[C_{16}H_{14}OClFN_2]^+$: 305.0851, found 305.0849 $[M+H]^+$.

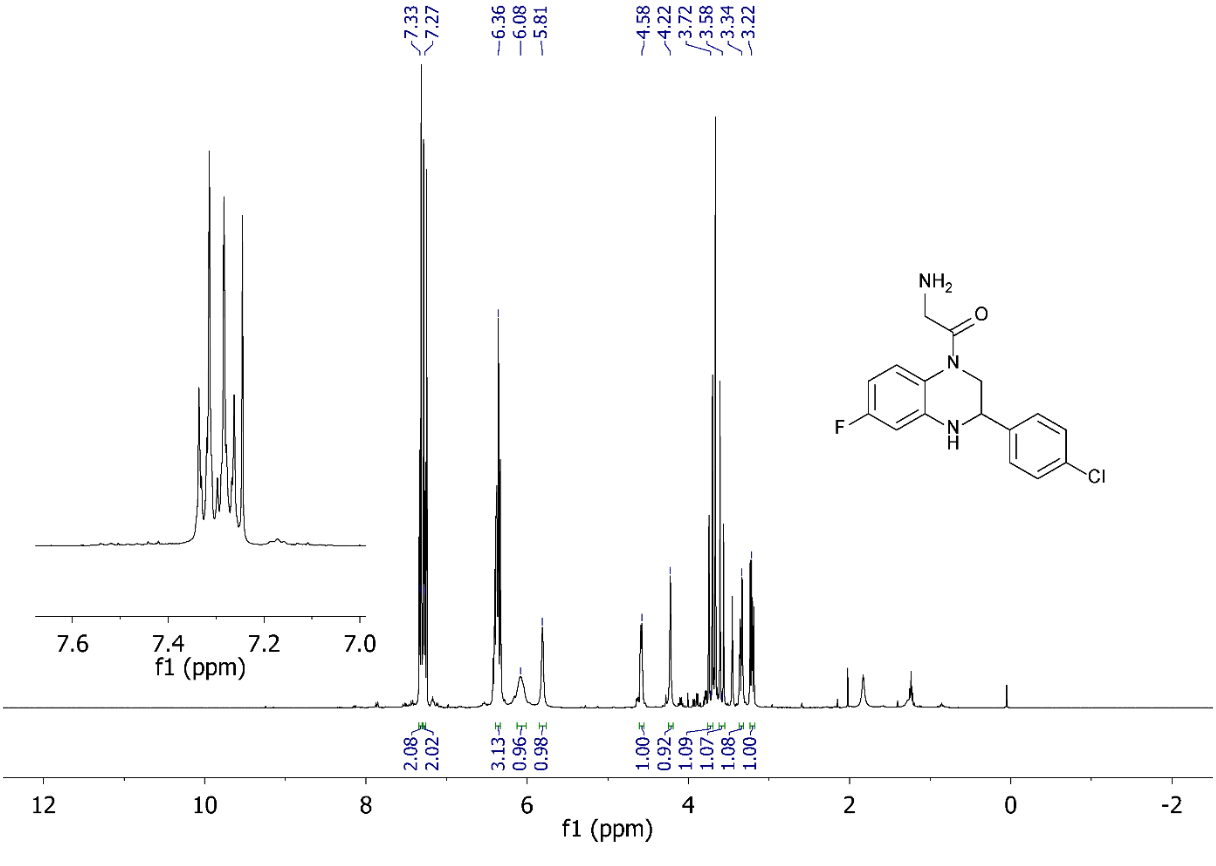

Figure S124. Compound **9c**, ^{1}H NMR (400 MHz, $CDCl_3$).

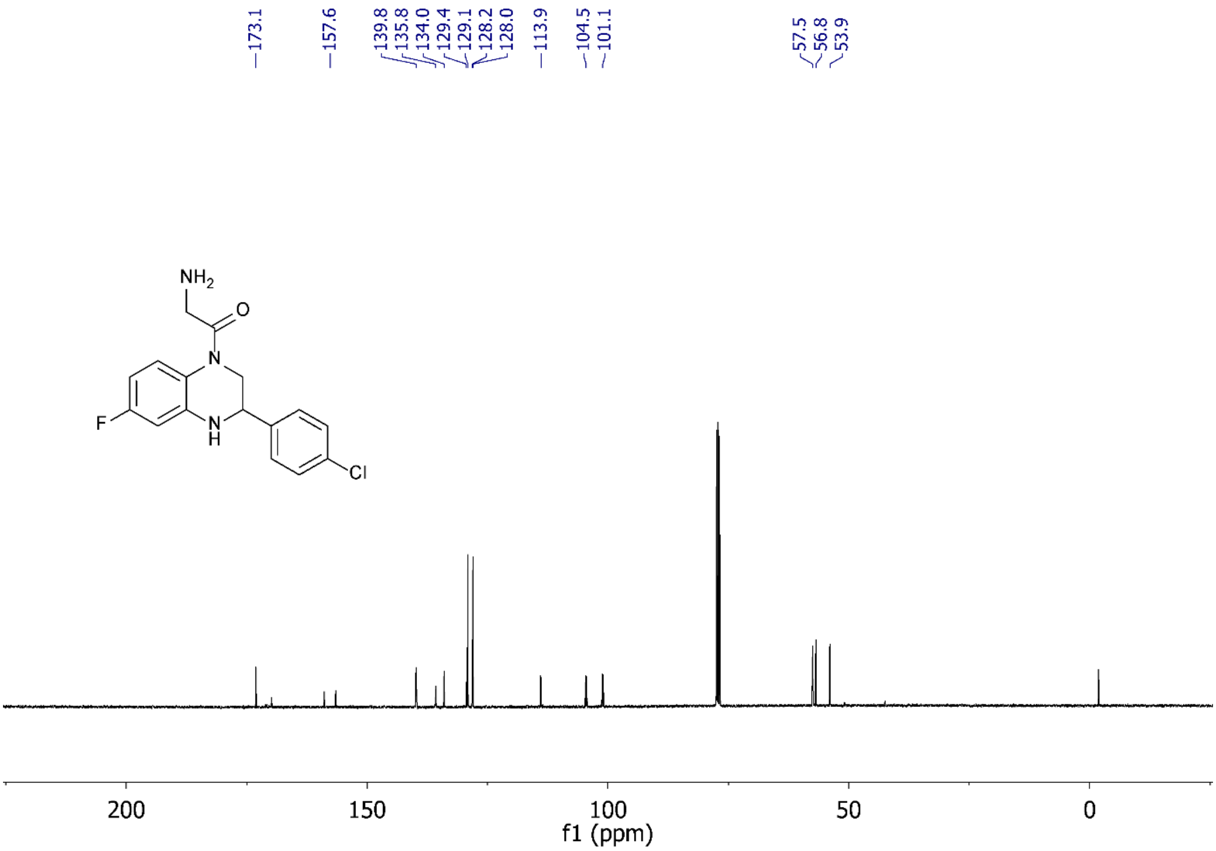

Figure S125. Compound **9c**, ^{13}C NMR (101 MHz, $CDCl_3$).

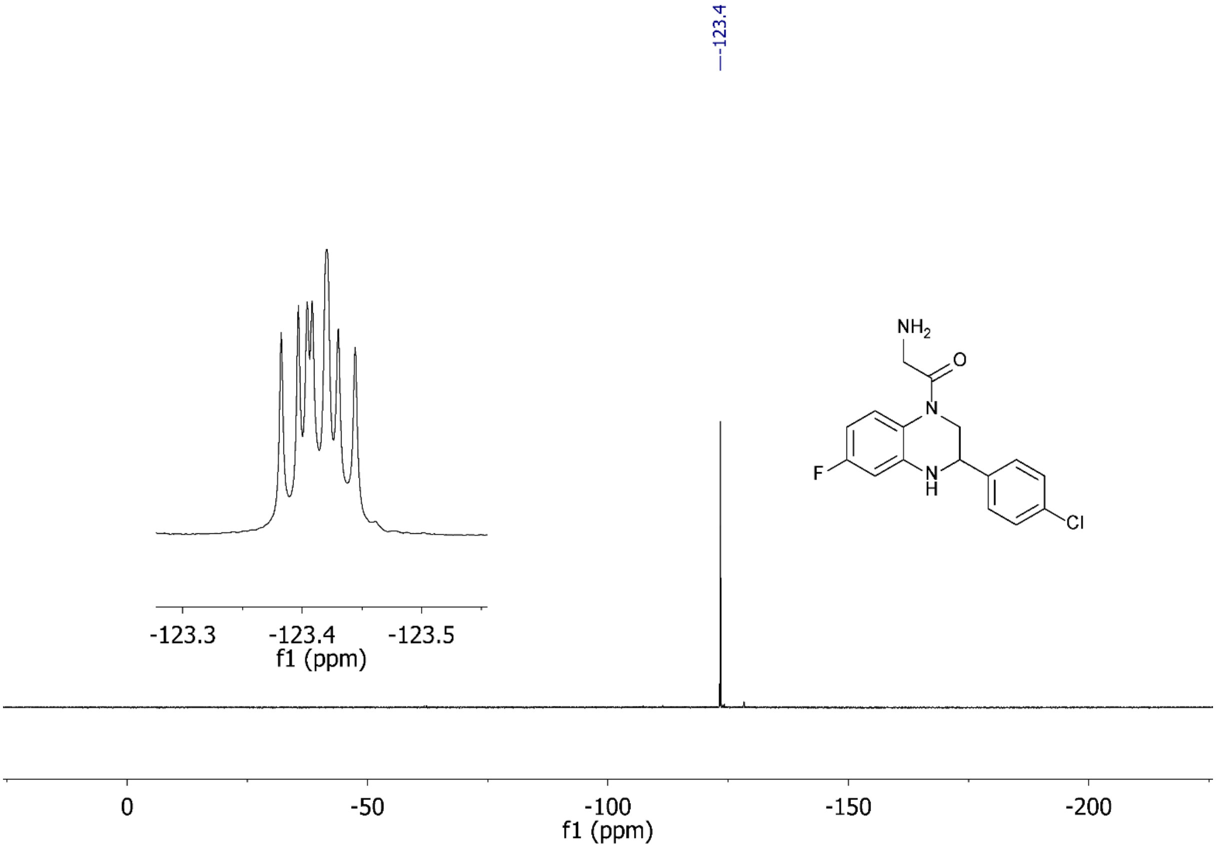

Figure S126. Compound **9c**, ^{19}F NMR (376.5 MHz, $CDCl_3$).

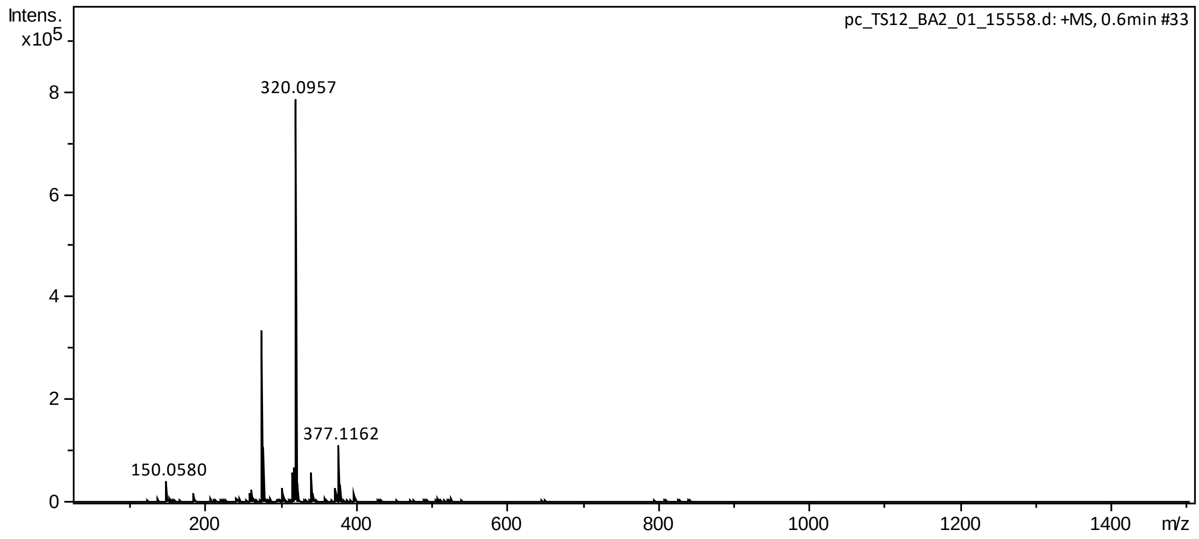

Figure S127. Compound **9c**, HRMS (ESI+) calc for $[C_{16}H_{15}ClFON_3+H]^+$: 320.0960, found 320.0957 $[M+H]^+$.

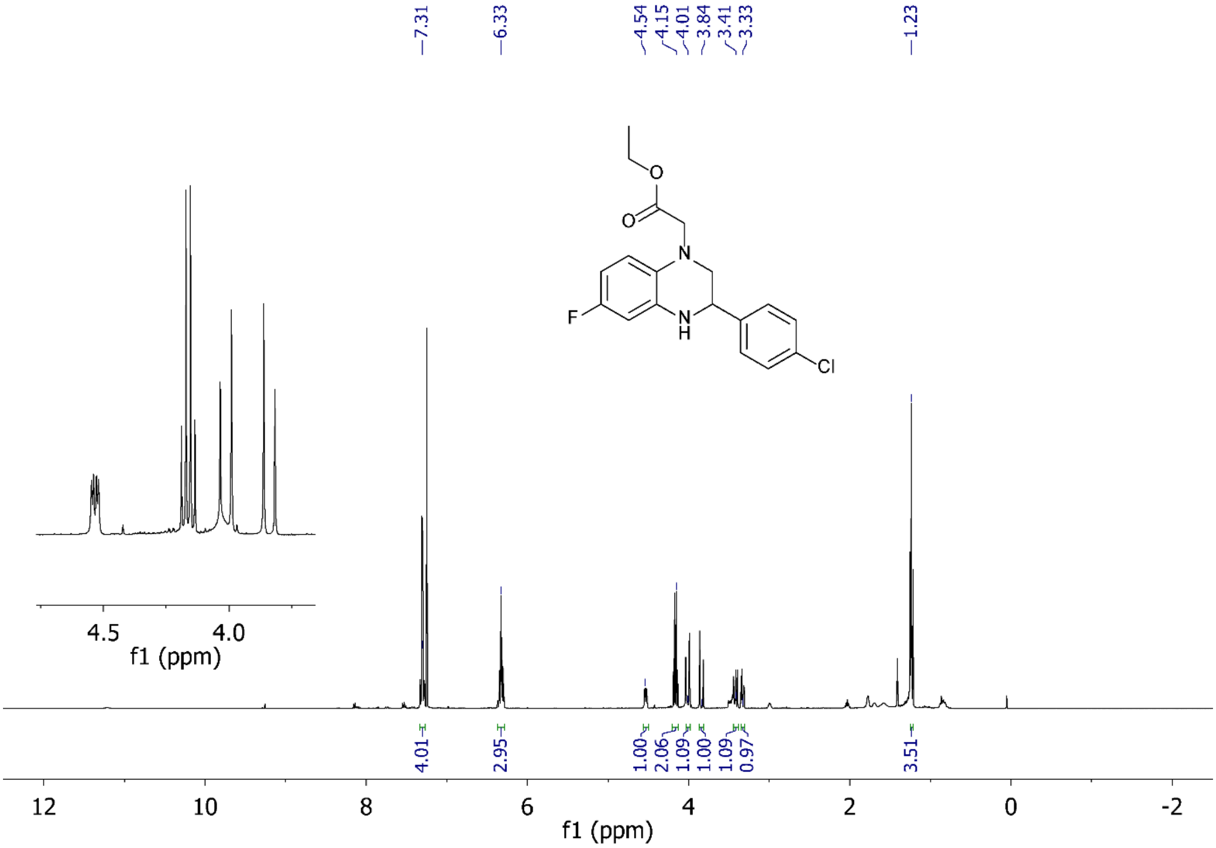

Figure S128. Compound **9d**, ^{1}H NMR (400 MHz, $CDCl_3$).

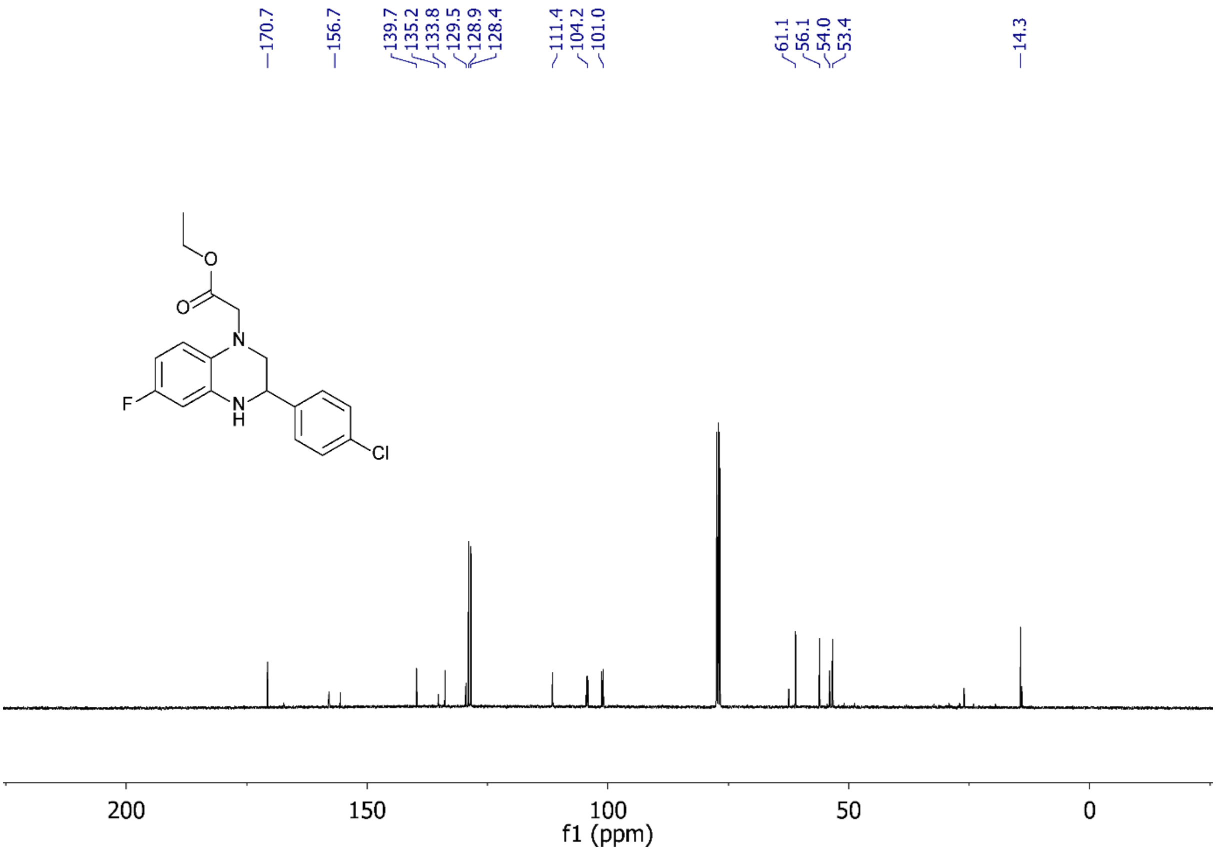

Figure S129. Compound **9d**, ^{13}C NMR (101 MHz, $CDCl_3$).

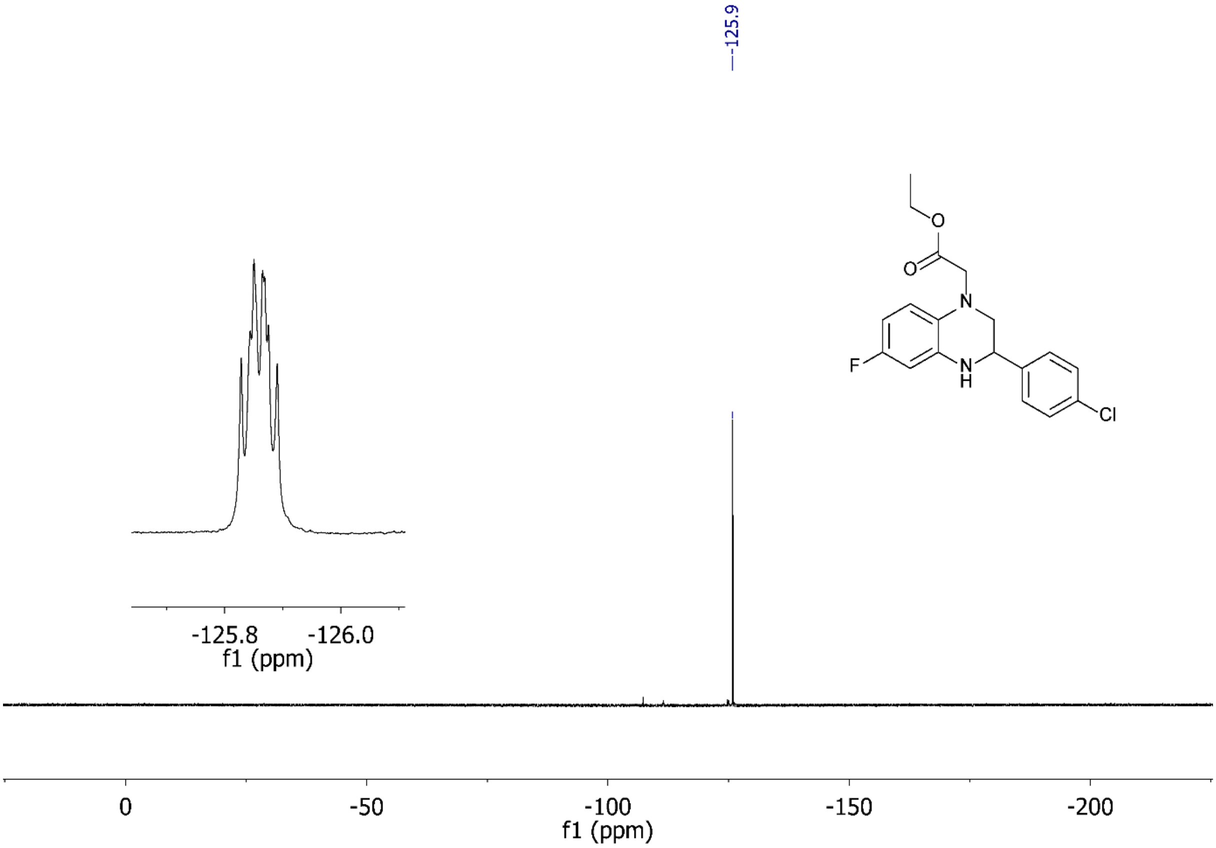

Figure S130. Compound **9d**, ^{19}F NMR (376.5 MHz, $CDCl_3$).

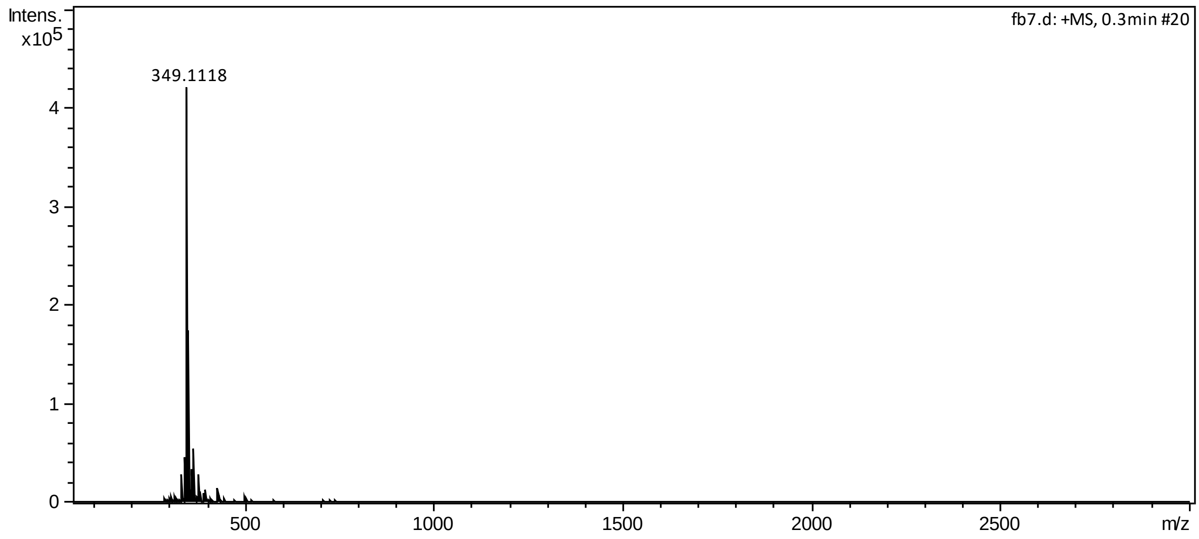

Figure S131. Compound **9d**, HRMS (APCI+) calc for $[C_{18}H_{18}N_2O_2ClF+H]^+$ calc 349.1113, found 349.118 $[M+H]^+$.

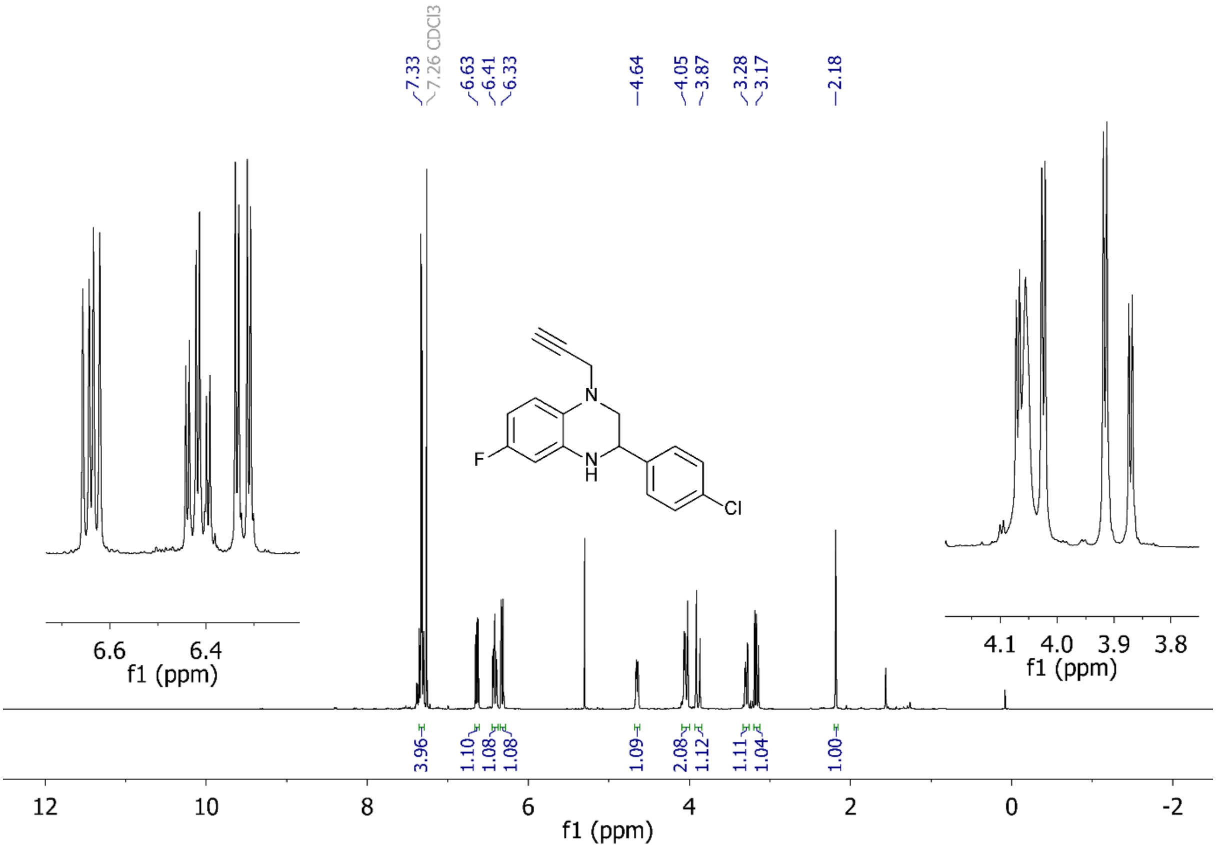

Figure S132. Compound **9e**, ^{1}H NMR (400 MHz, $CDCl_3$).

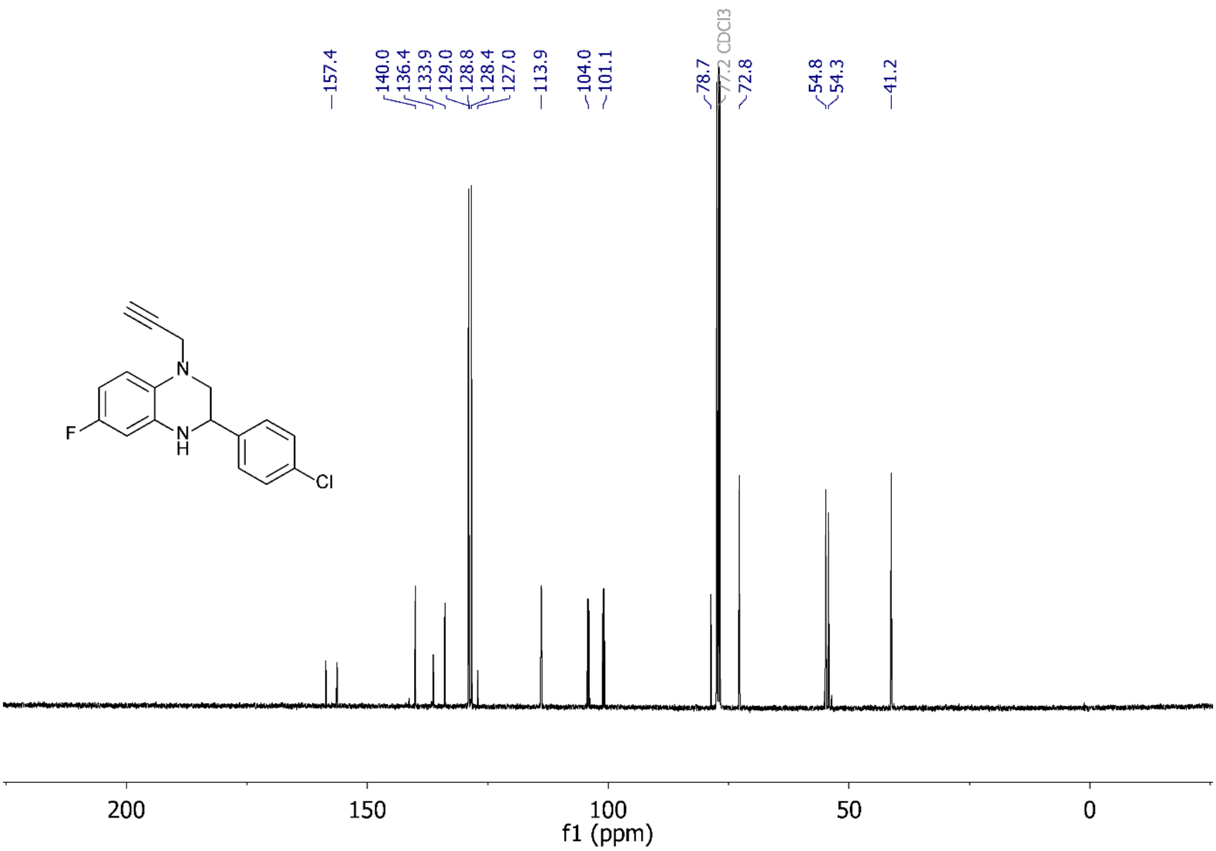

Figure S133. Compound **9e**, ^{13}C NMR (101 MHz, $CDCl_3$).

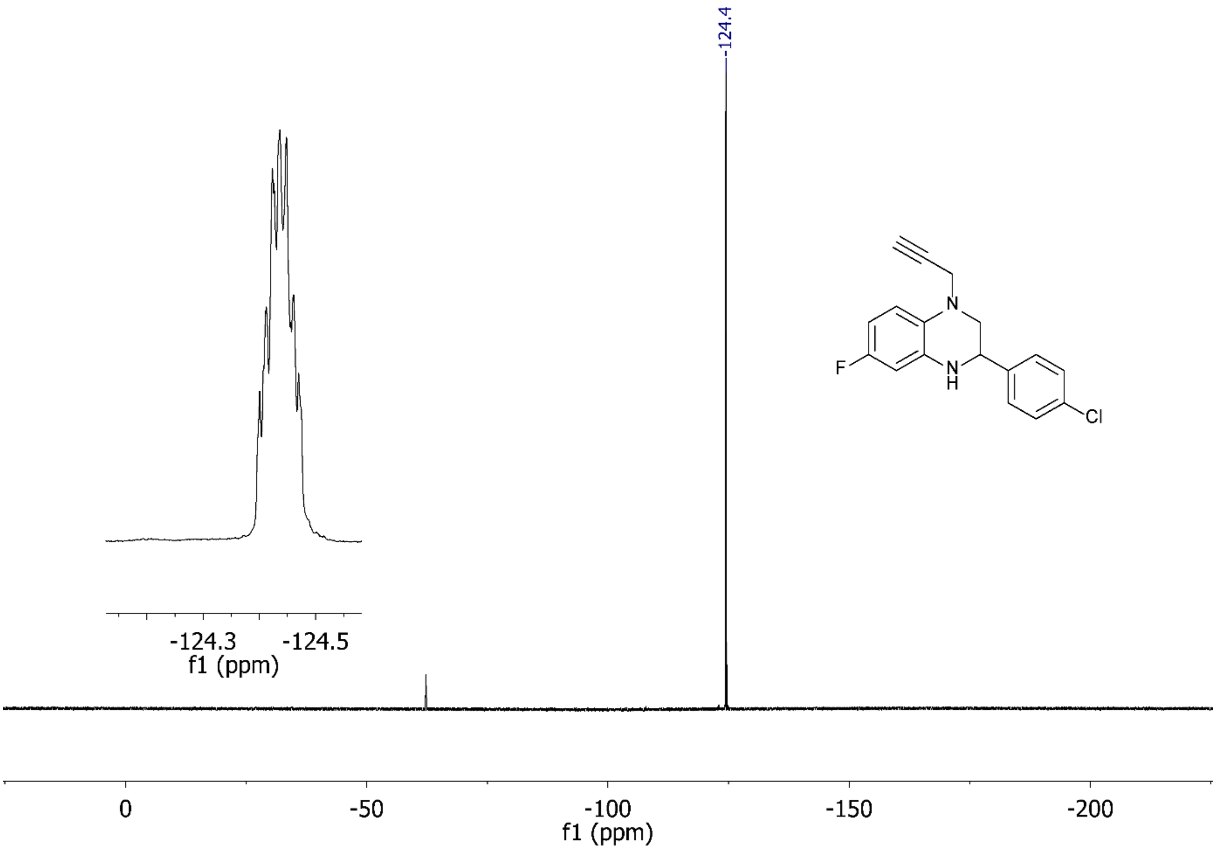

Figure S134. Compound **9e**, ^{19}F NMR (376.5 MHz, $CDCl_3$).

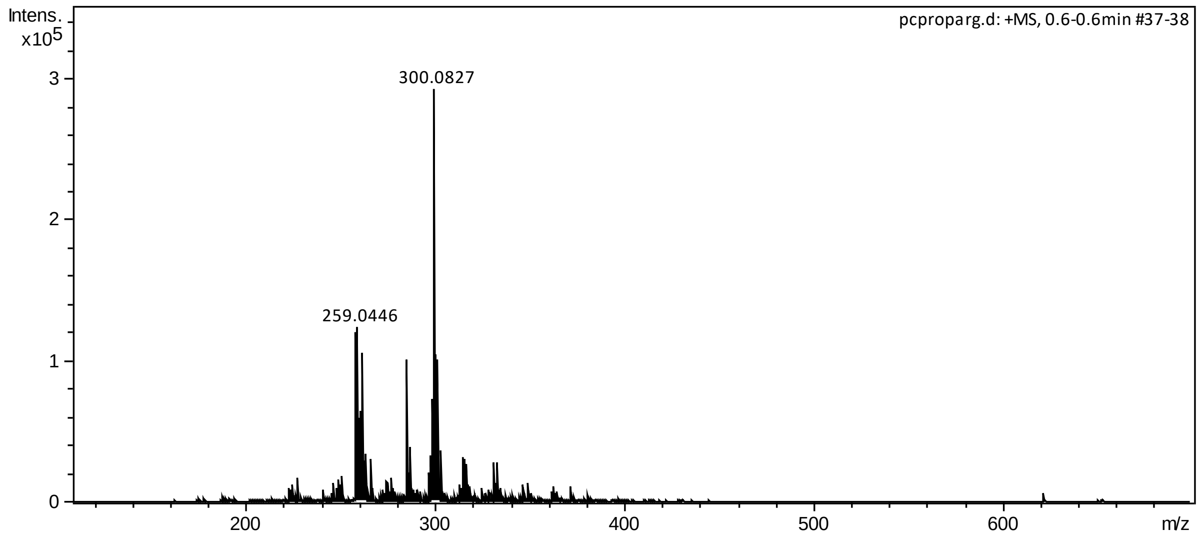

Figure S135. Compound **9e**, HRMS (APCI+) calc for $[C_{17}H_{14}F_2ClN_2]^+$: 300.0824, found 300.0827 $[M]^+$.

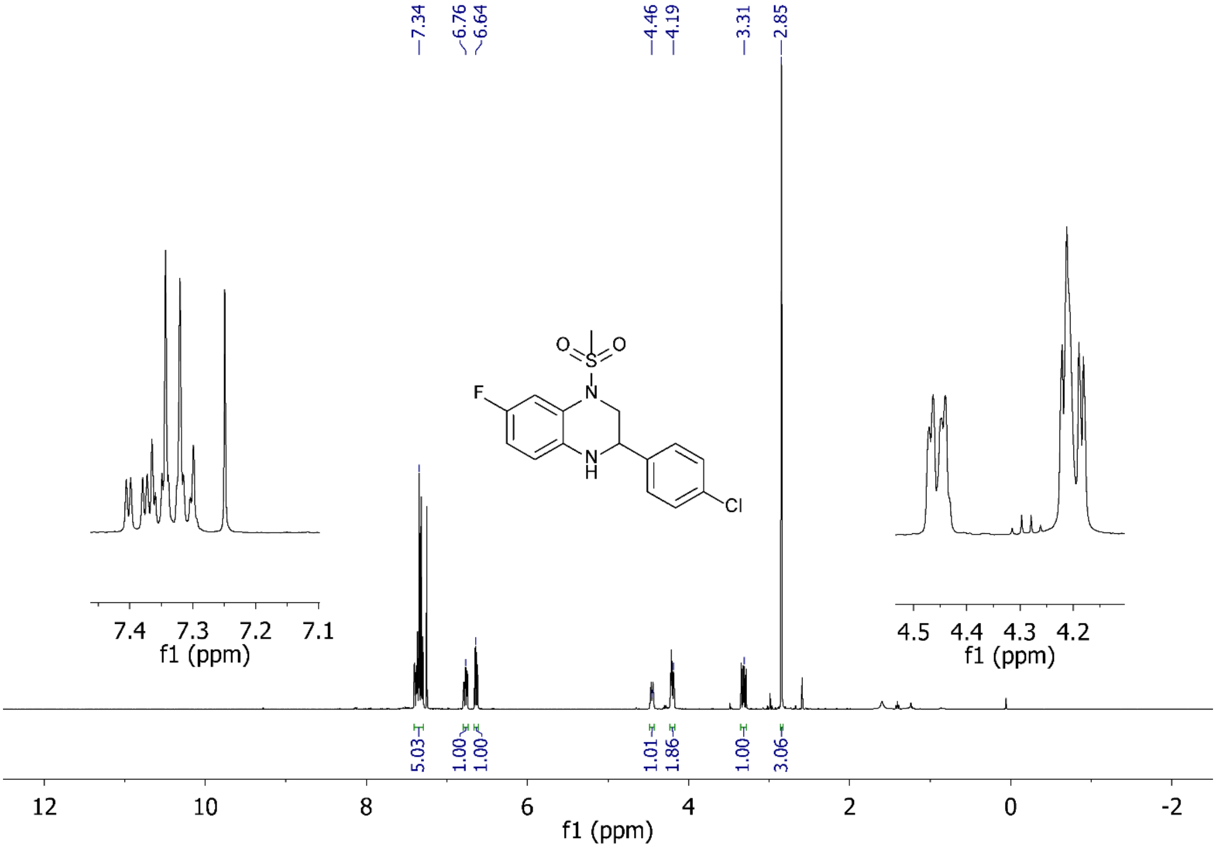

Figure S136. Compound **10a**, ^{1}H NMR (400 MHz, $CDCl_3$).

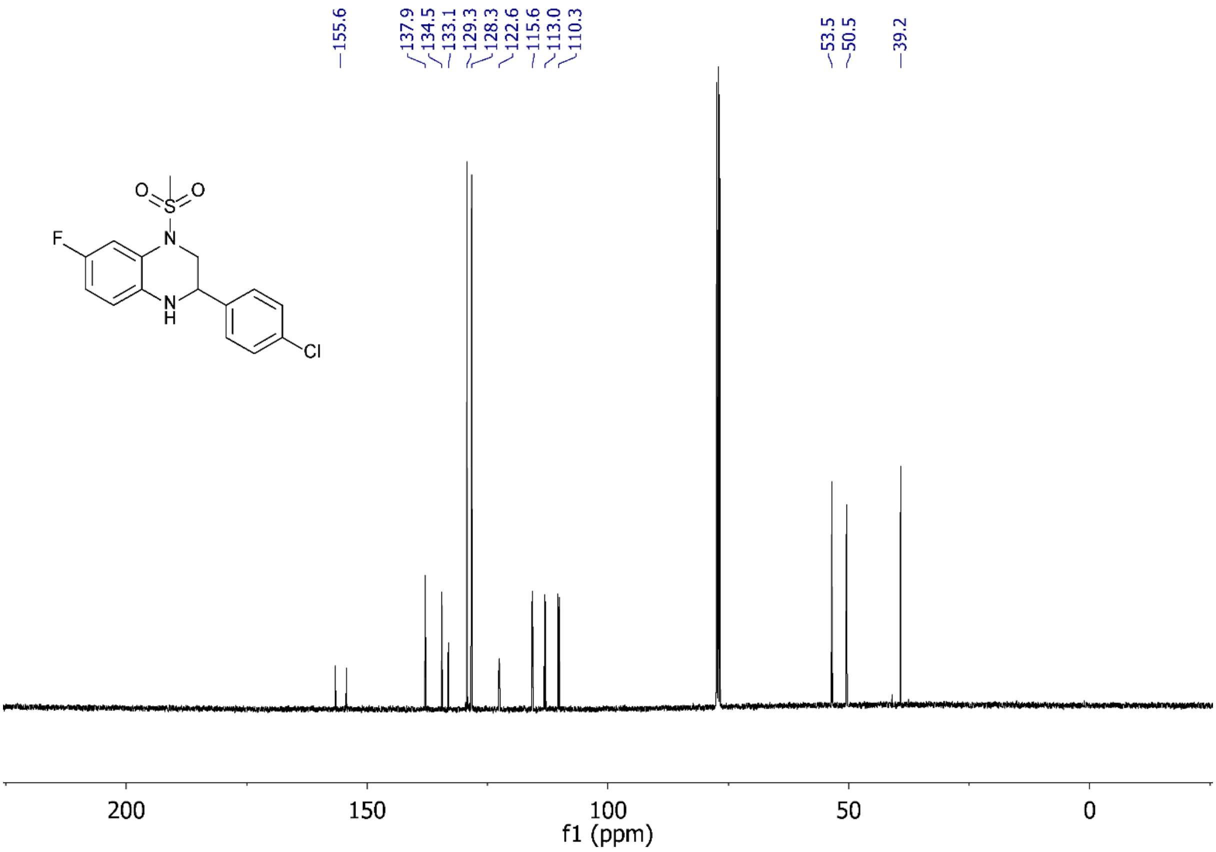

Figure S137. Compound **10a**, ^{13}C NMR (101 MHz, $CDCl_3$).

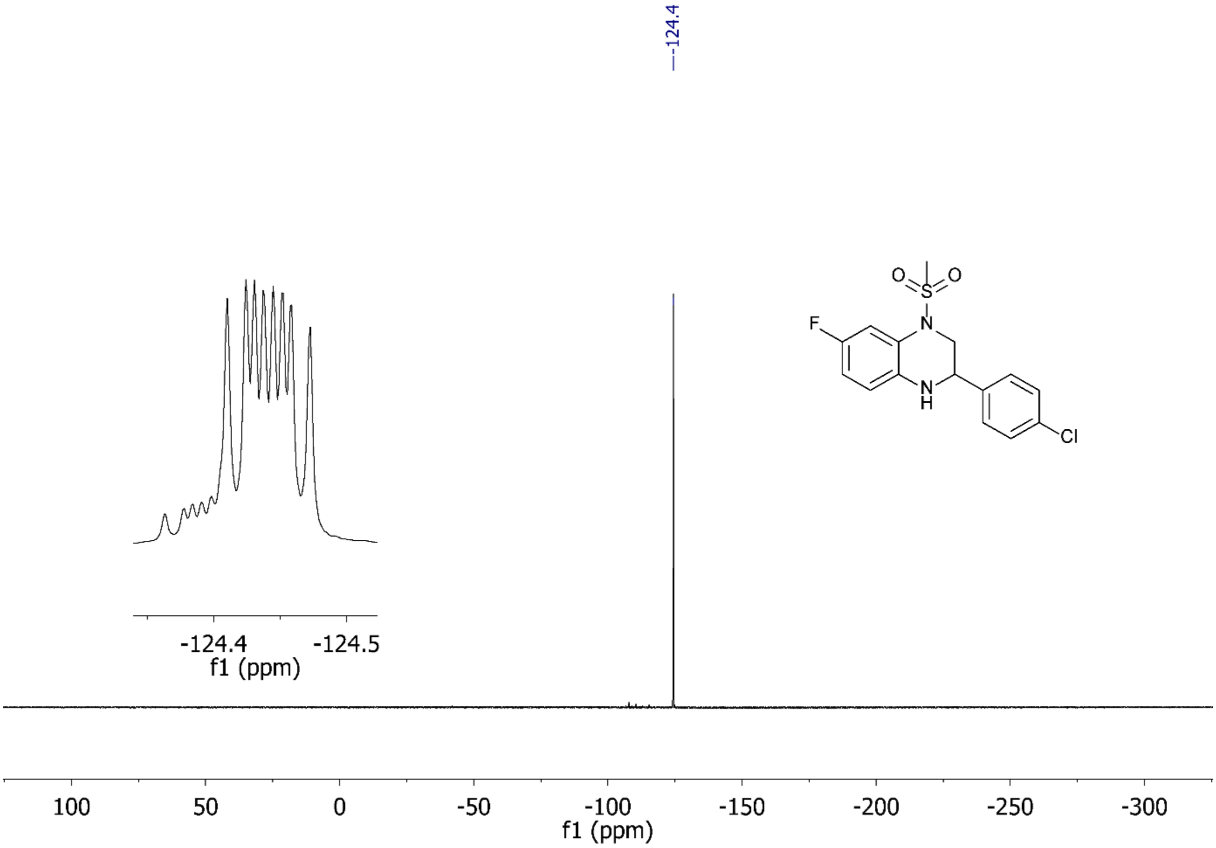

Figure S138. Compound **10a**, ^{19}F NMR (376.5 MHz, $CDCl_3$).

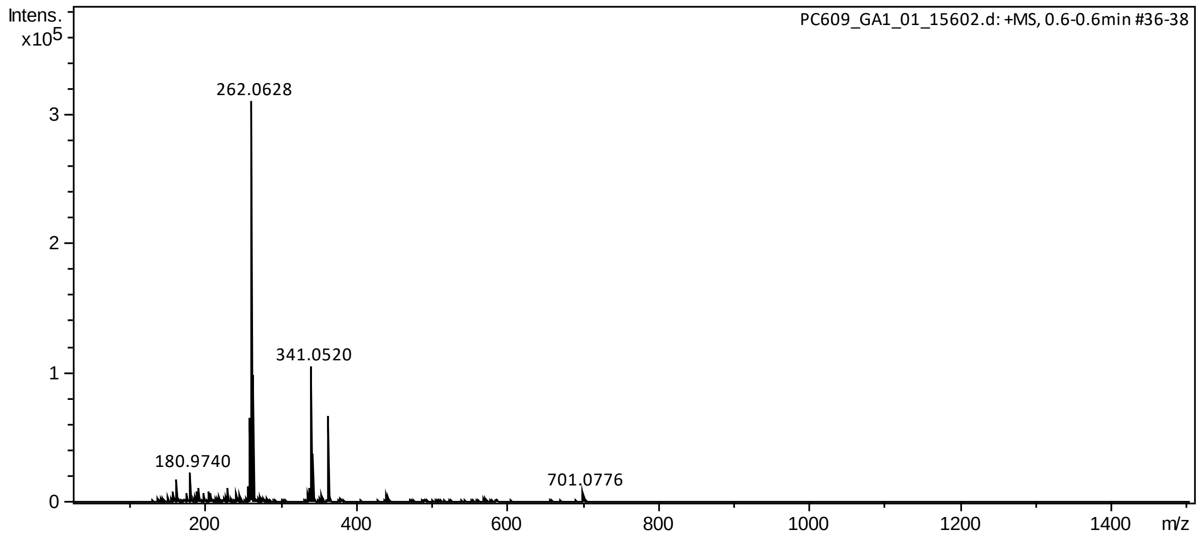

Figure S139. Compound **10a**, HRMS (ESII+) calcd for $[C_{15}H_{14}ClFN_2O_2S+H]^+$: 341.521 found 341.520 $[M+H]^+$

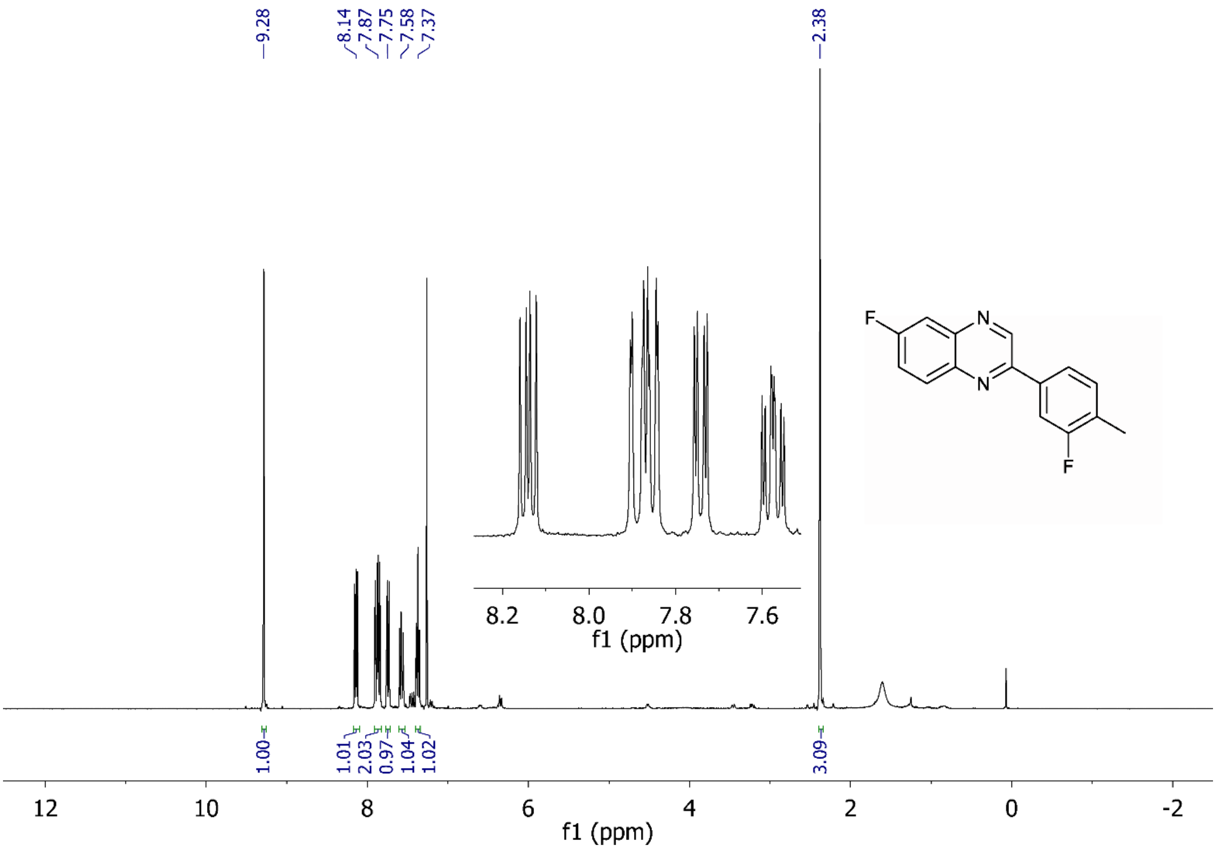

Figure S140. Compound **3b-Ar**, ^1H NMR (400 MHz, $CDCl_3$).

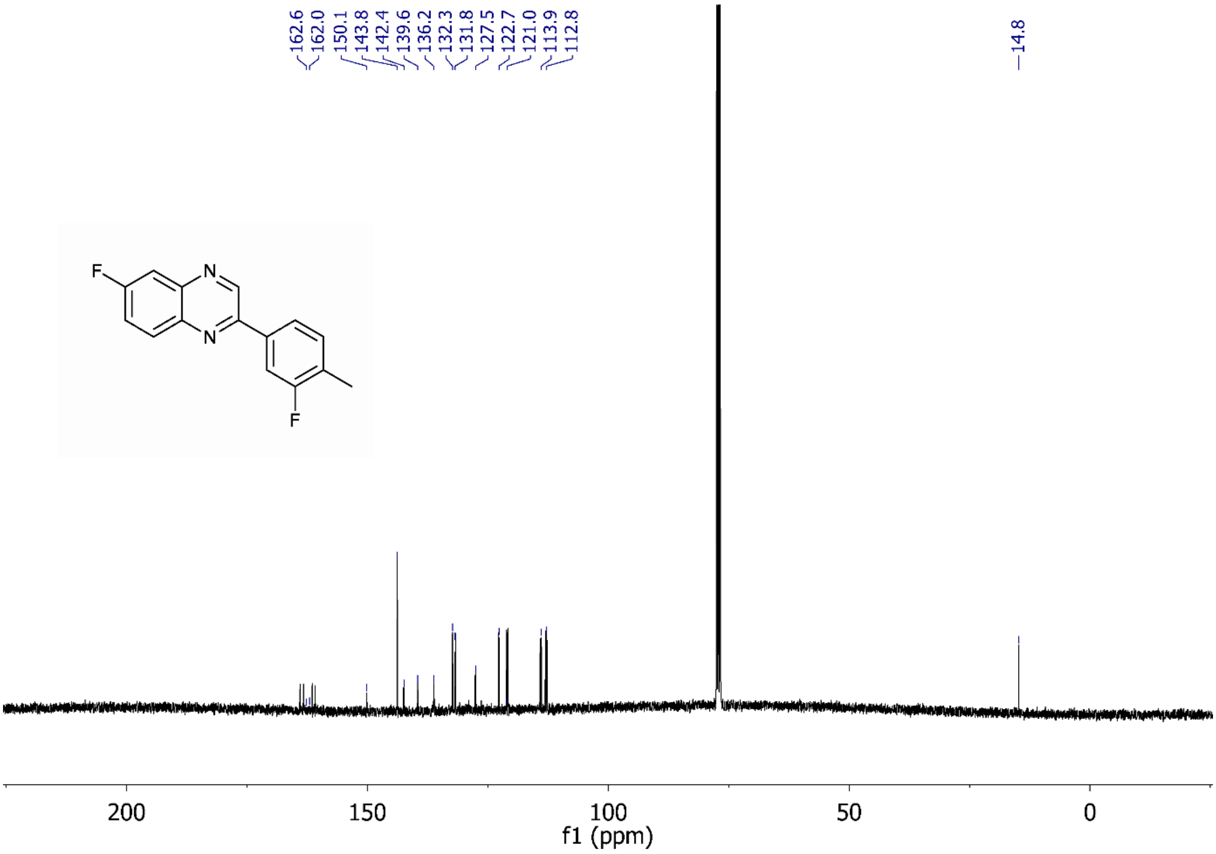

Figure S141. Compound **3b-Ar**, ^{13}C NMR (101 MHz, $CDCl_3$).

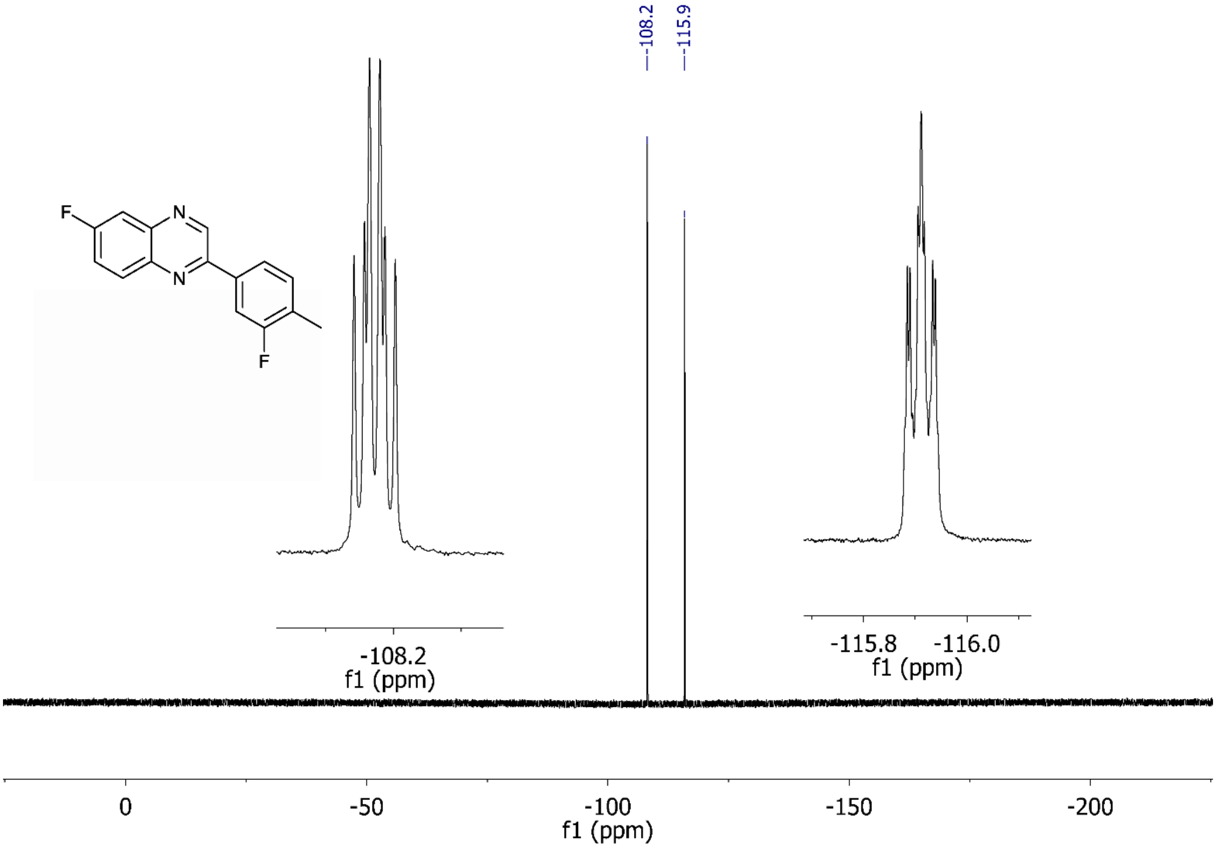

Figure S142. Compound **3b-Ar**, ^{19}F NMR (376.5 MHz, $CDCl_3$).

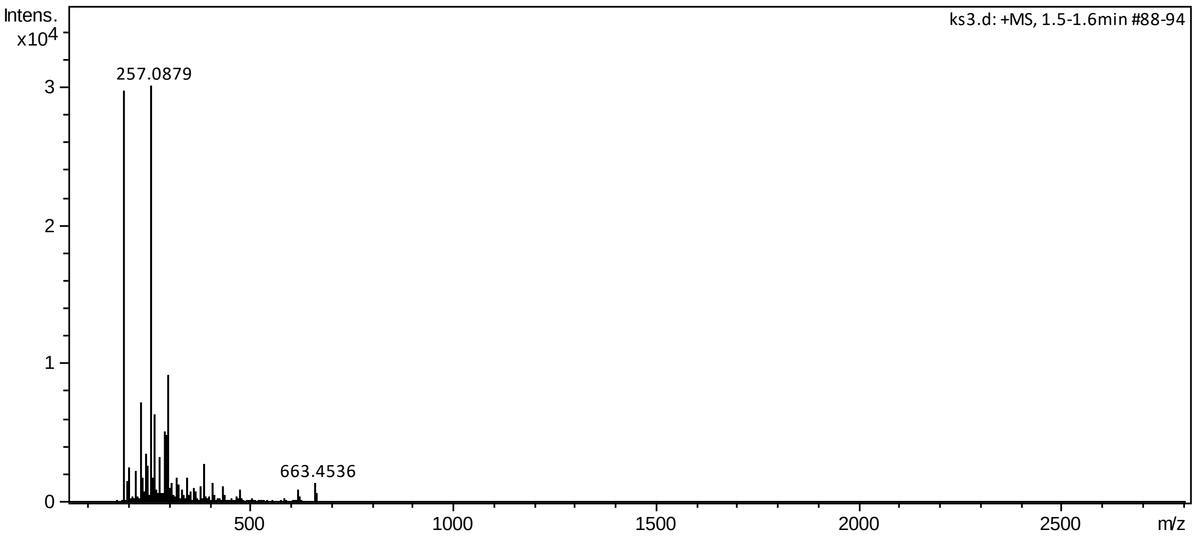

Figure S143. Compound **3b-Ar**, HRMS (APCI+) calcd for $[C_{15}H_{11}N_2F_2+H]^+$: 257.0884 found 257.0879 $[M+H]^+$.

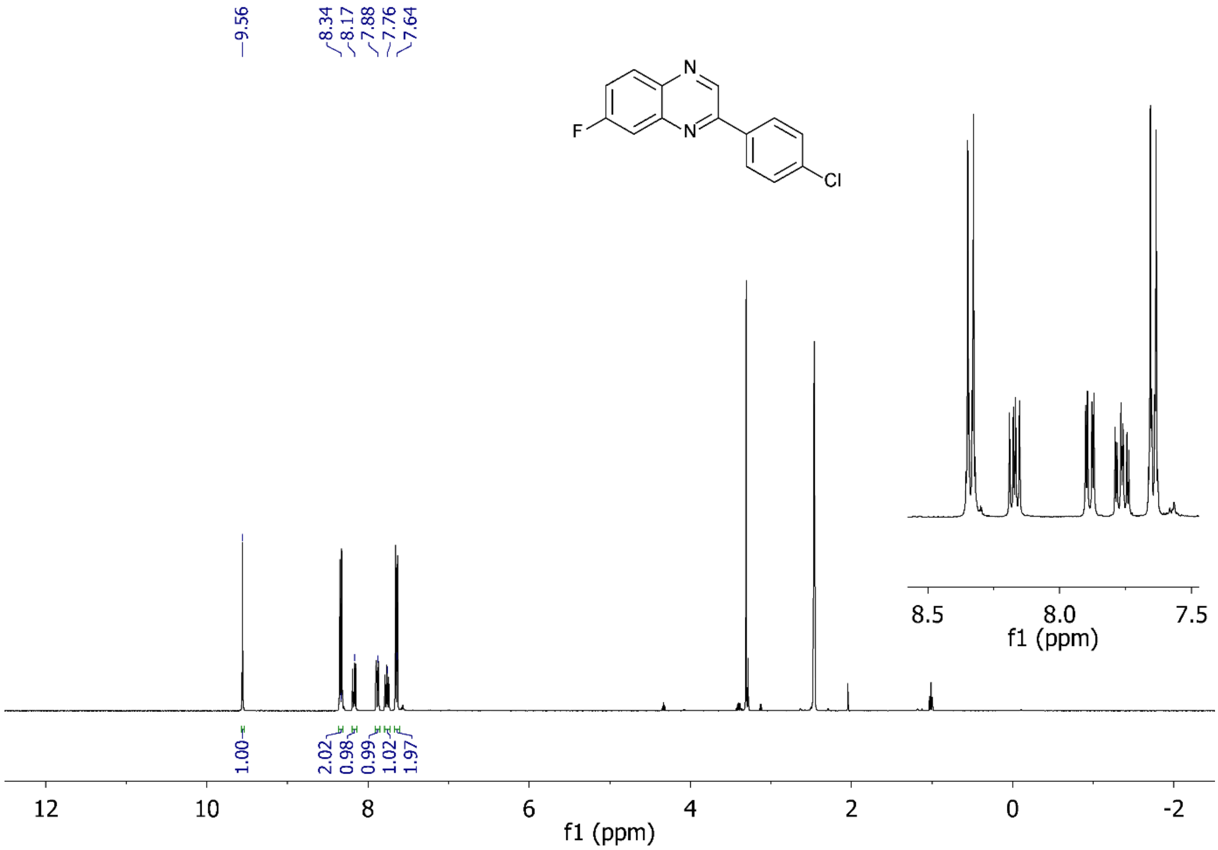

Figure S144. Compound **4c-Ar**, ^1H NMR (400 MHz, $CDCl_3$).

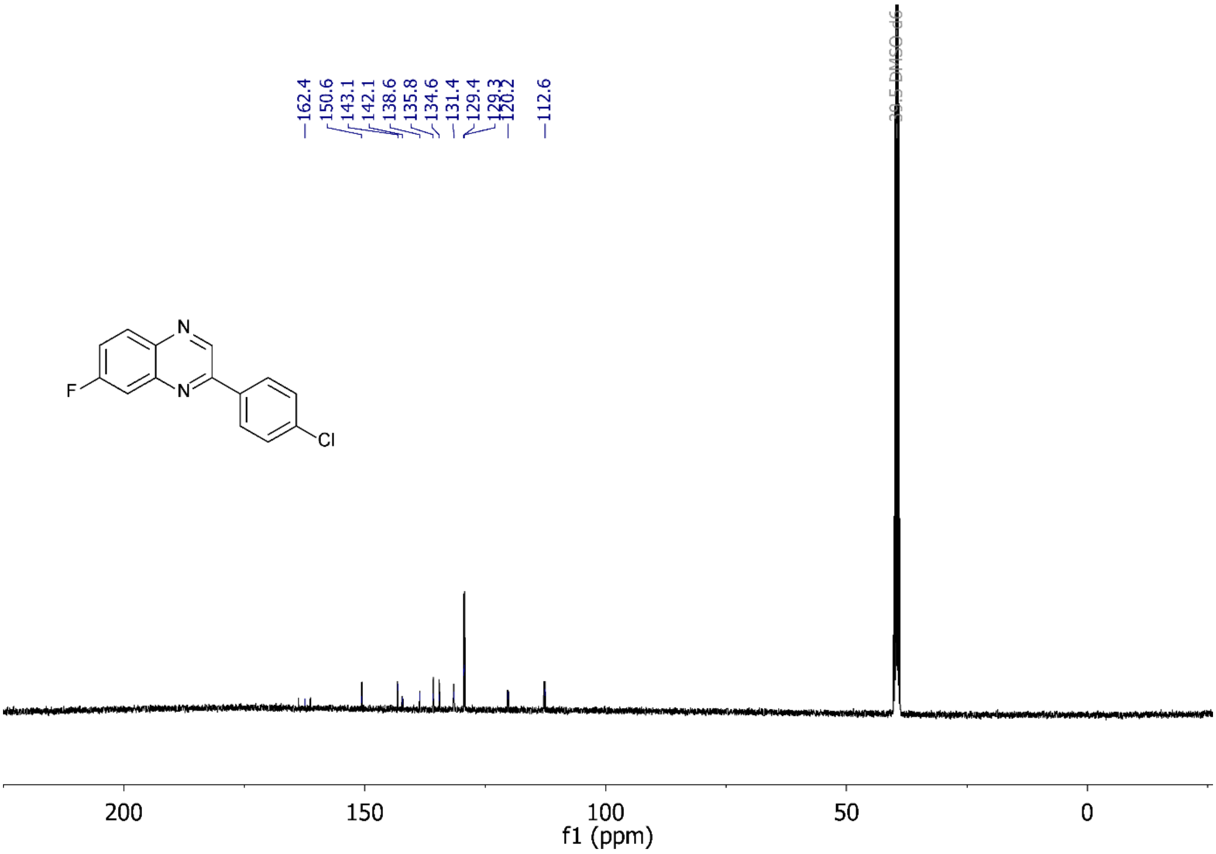

Figure S145. Compound **4c-Ar**, ^{13}C NMR (101 MHz, $CDCl_3$).

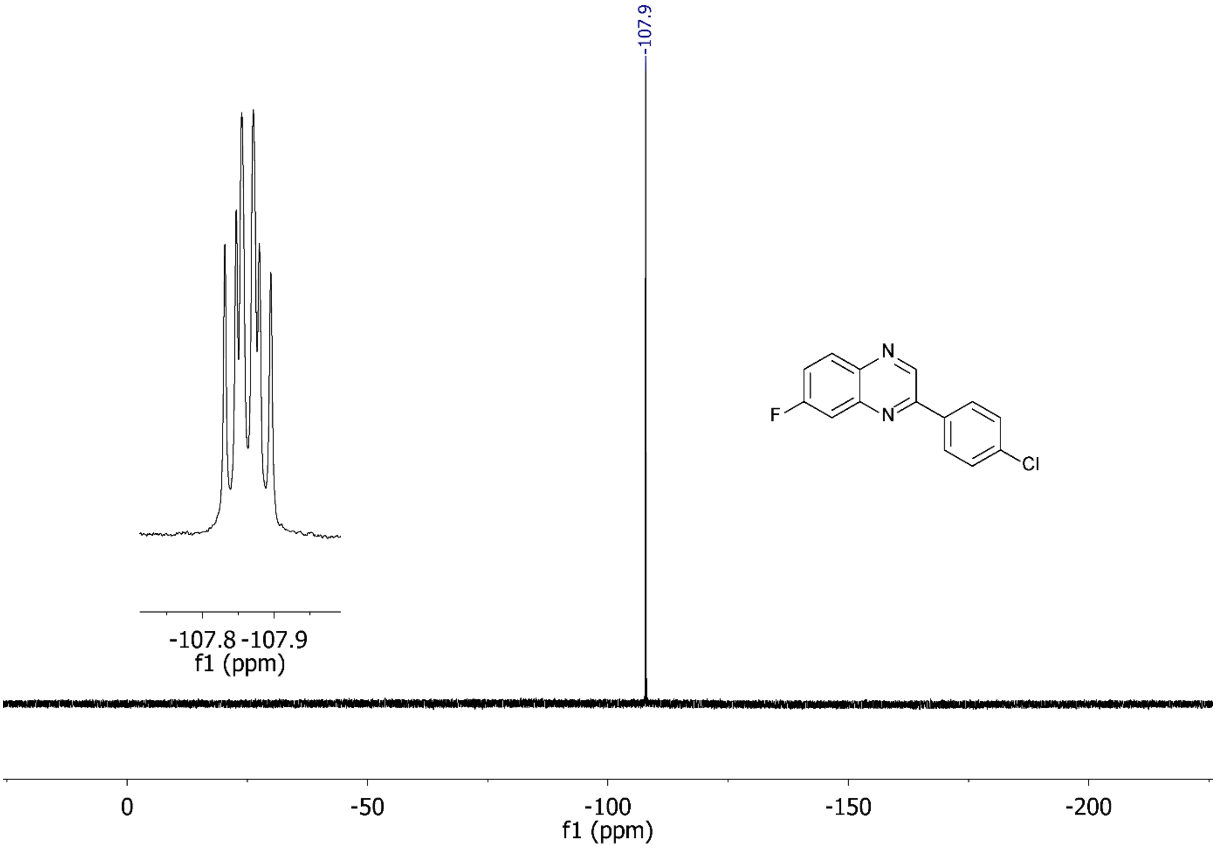

Figure S146. Compound **4c-Ar**, ^{19}F NMR (376.5 MHz, $CDCl_3$).

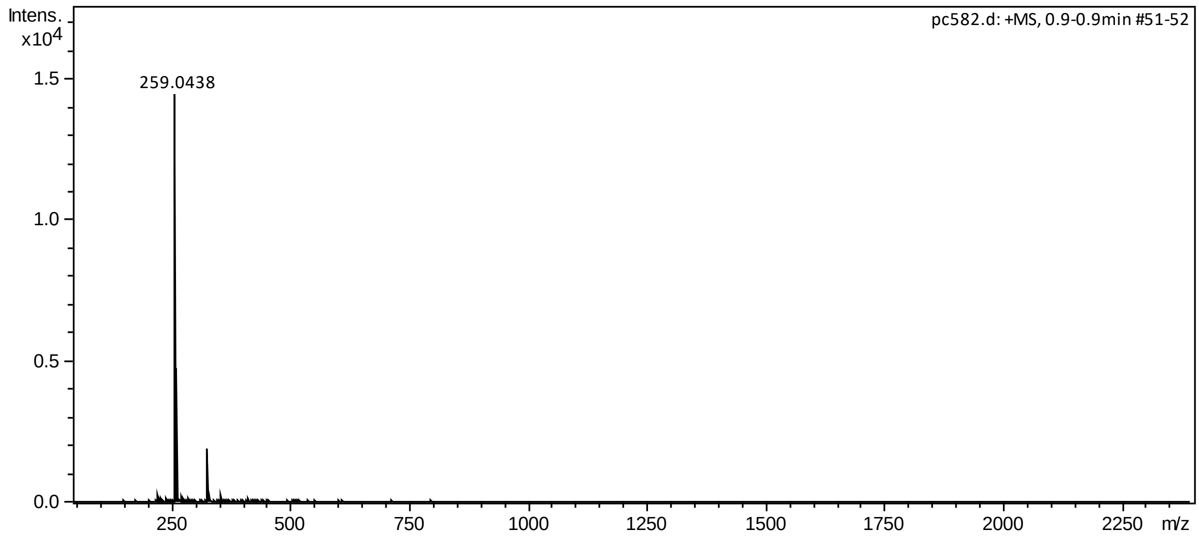

Figure S147. Compound **4c-Ar**, HRMS (APCI+) calc for $[C_{14}H_8N_2FCl +H]^+$ calc 259.0432, found 259.0438 $[M+H]^+$.

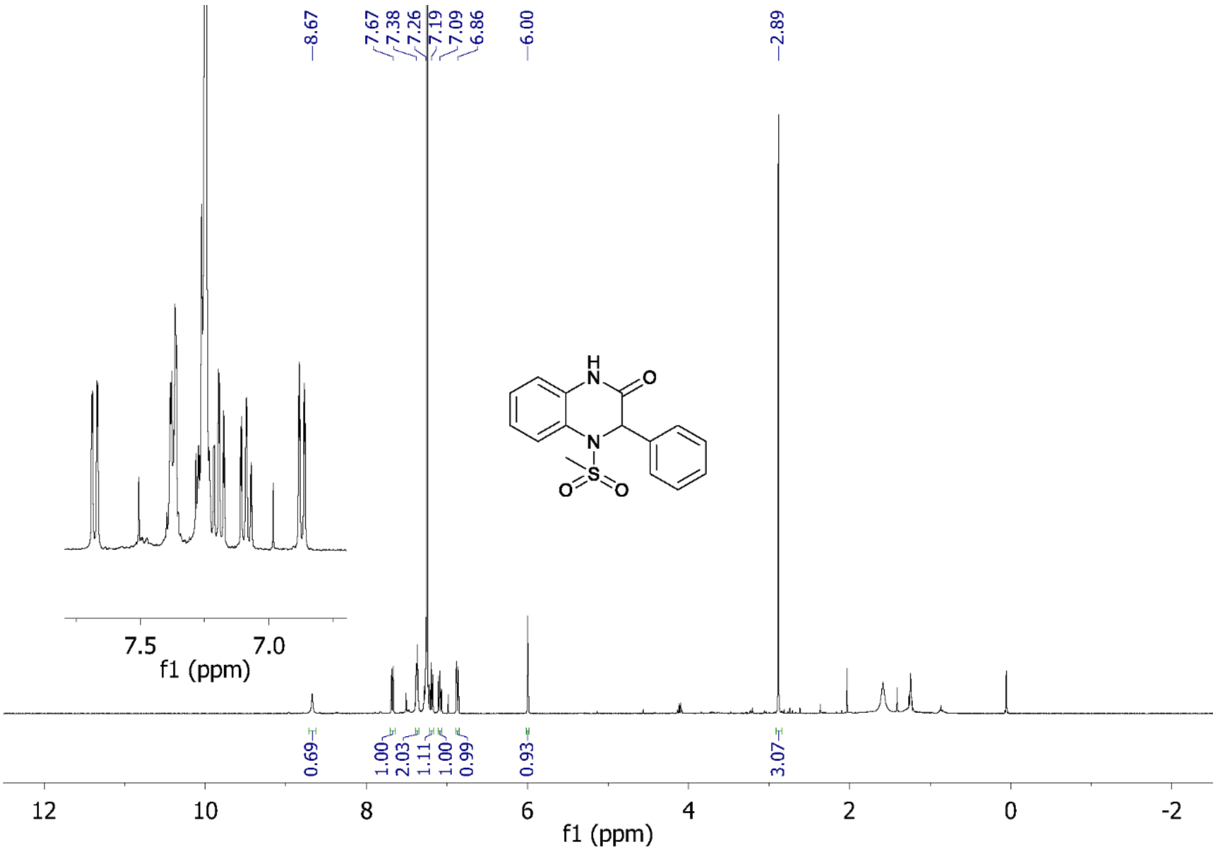

Figure S148. Compound **11a**, ^{1}H NMR (400 MHz, $CDCl_3$).

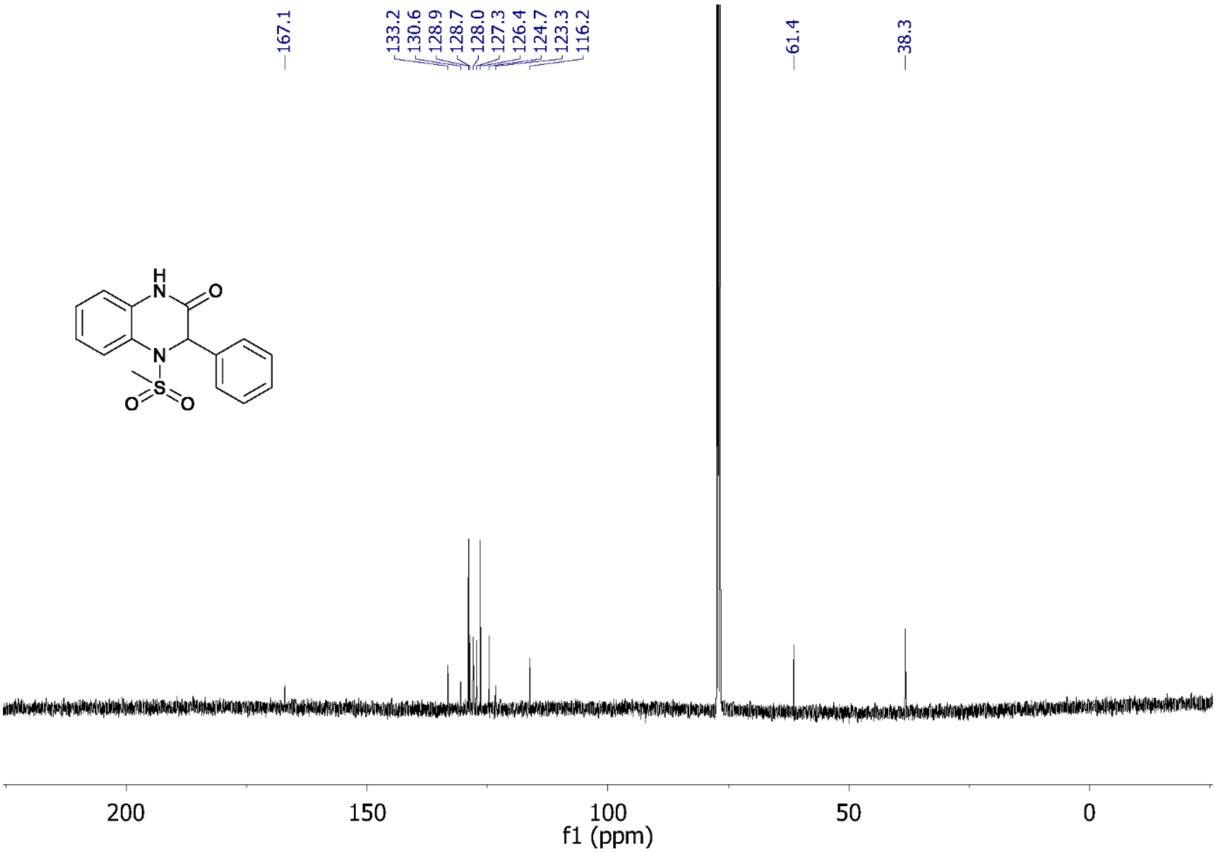

Figure S149. Compound **11a**, ^{13}C NMR (101 MHz, $CDCl_3$).

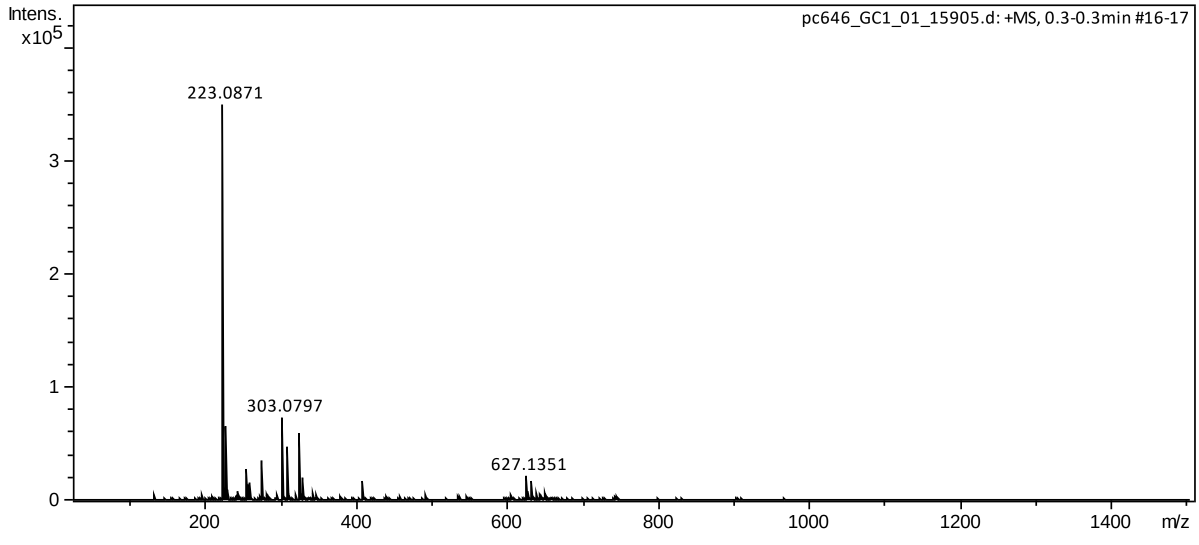

Figure S150. Compound **11a**, HRMS (ESI+) calc for $[C_{15}H_{14}N_2O_3S+H]^+$: 303.0798; found 303.0797 $[M+H]^+$.

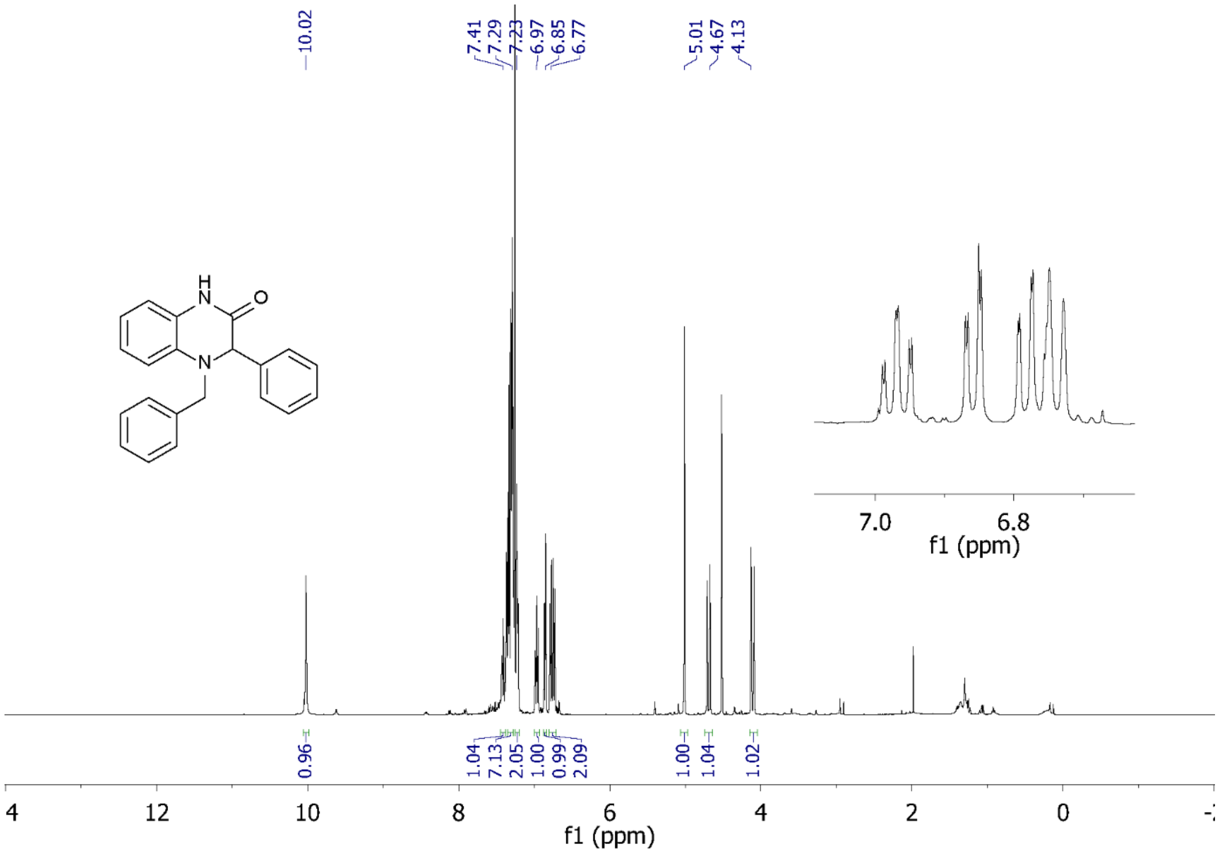

Figure S151. Compound **11b**, ^{1}H NMR (400 MHz, $CDCl_3$).

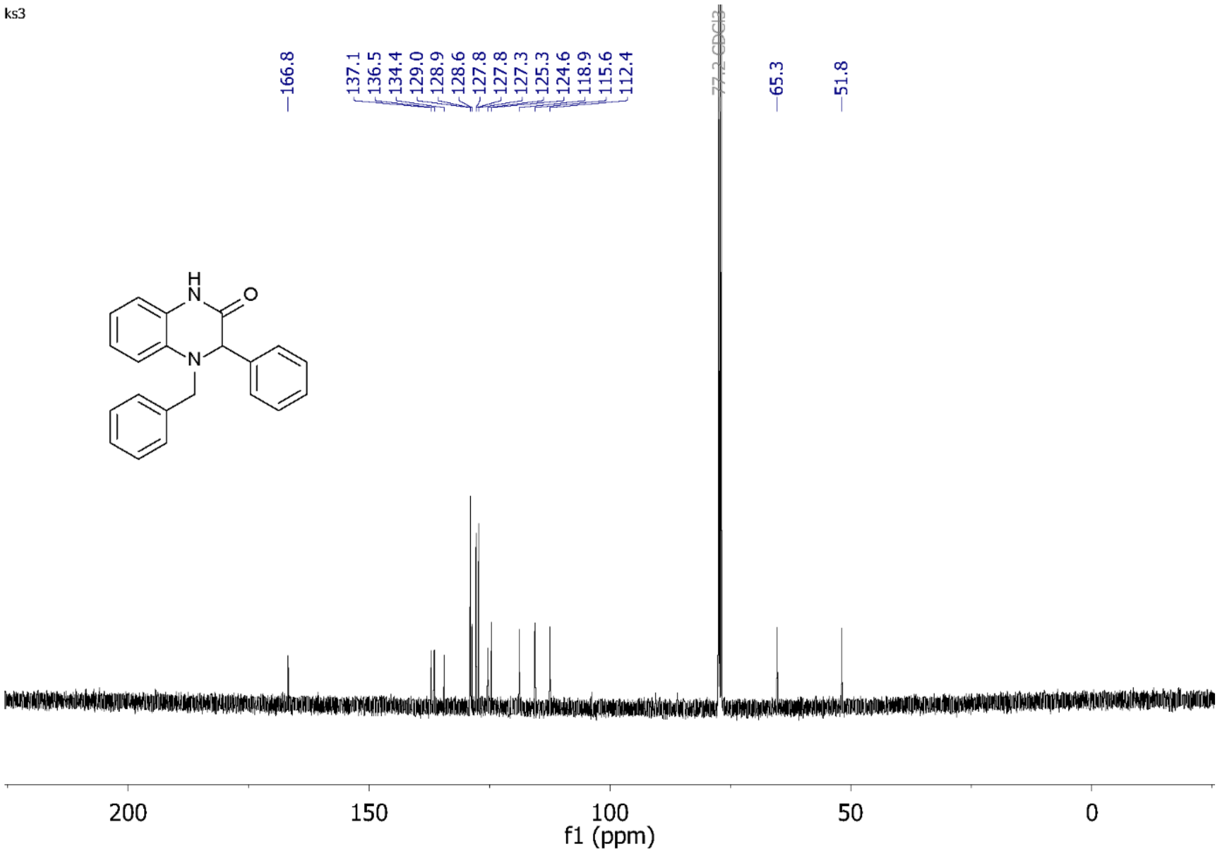

Figure S152. Compound **11b**, ^{13}C NMR (101 MHz, $CDCl_3$).

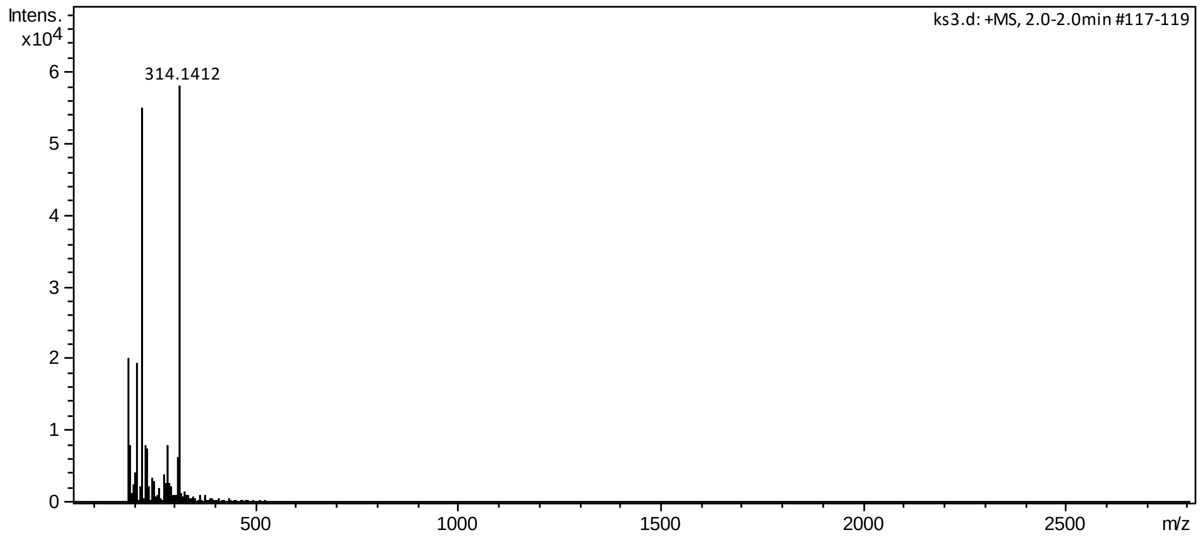

Figure S153. Compound **11b**, HRMS (APCI+) calc for $[C_{21}H_{19}N_2O+H]^+$: 314.1414; found 314.1412 $[M+H]^+$.

3. Crystalographic data

Crystallographic data for *R*-**4A**

M = 228.27 g.mol^{-1}, orthorhombic system, space group $P2_12_12_1$, *a* = 6.0144 (2) Å, *b* = 12.6163 (3) Å, *c* = 14.9289 (4) Å, *Z* = 4, *V* = 1132.80 (6) Å^3, D_c = 1.338 g.cm^{-3}, *μ*(Cu-Kα) = 0.75 mm^{-1}, crystal dimensions of 0.46 × 0.34 × 0.28 mm. Data were collected at 180 (2) K on a Bruker D8 Venture Photon CMOS diffractometer with Incoatec microfocus sealed X-ray tube (Cu-*Kα* radiation). The structure was solved by charge flipping methods and anisotropically refined by full matrix least squares on F squared using the CRYSTALS to final value *R* = 0.026 and *wR* = 0.070 using 2240 independent reflections (θ_{max} = 72.1°), 164 parameters and 8 restrains. The hydrogen atoms bonded to carbon atoms were placed in calculated positions and refined with a riding constrains, while hydrogen atoms bonded to nitrogen we refined with restrained geometry. MCE was used for visualization of electron density maps. The structure was deposited into Cambridge Structural Database under number CCDC 2505539.

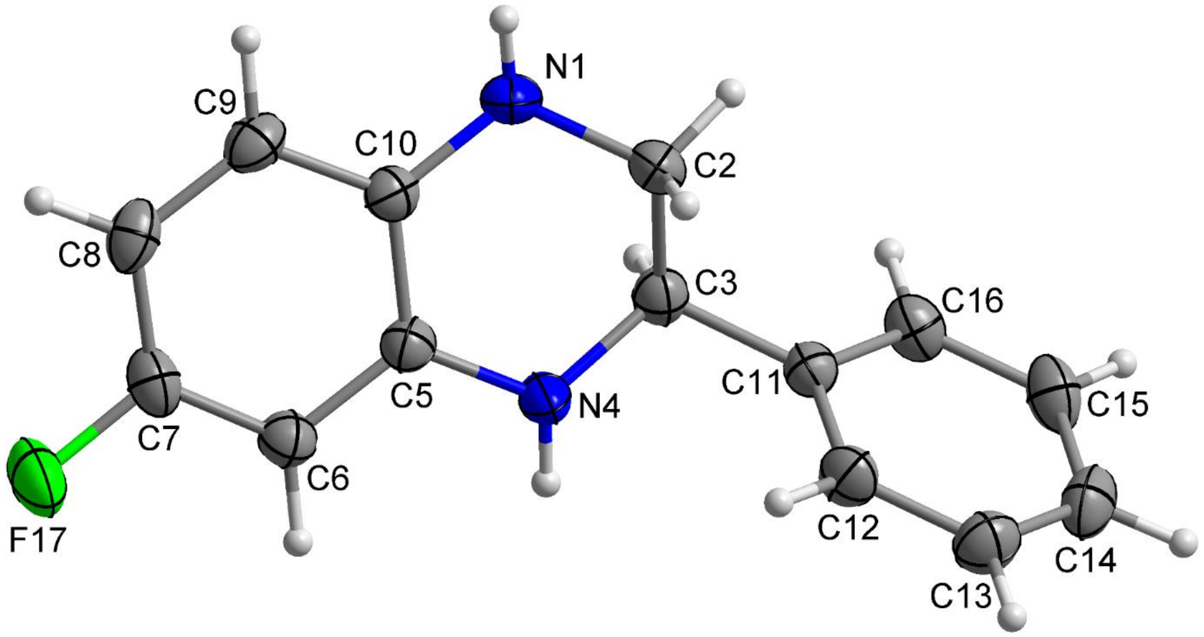

The crystal structure *R*-**4A**, the ADPs drawn at 50% probability level.

Crystallographic data for S-**4A**

M = 228.27 g.mol^{-1}, orthorhombic system, space group $P2_12_12_1$, *a* = 6.00666 (5) Å, *b* = 12.59608 (11) Å, *c* = 14.83212 (12) Å, *Z* = 4, *V* = 1122.20 (2) Å^3, D_c = 1.351 g.cm^{-3}, *μ*(Cu-Kα) = 0.75 mm^{-1}, crystal dimensions of 0.41 × 0.27 × 0.18 mm. Data were collected at 120 (2) K on a Rigaku OD Gemini Atlas S2 CCD diffractometer with mirror collimated sealed X-ray tube (Cu-*Kα* radiation). The structure was solved by charge flipping methods and anisotropically refined by full matrix least squares on F squared using the CRYSTALS to final value *R* = 0.027 and *wR* = 0.065 using 2034 independent reflections (θ_{max} = 67.8°), 164 parameters and 8 restrains. The hydrogen atoms bonded to carbon atoms were placed in calculated positions and refined with a riding constrains, while hydrogen atoms bonded to nitrogen were refined with restrained geometry. MCE was used for visualization of electron density maps. The structure was deposited into Cambridge Structural Database under number CCDC 2505538.

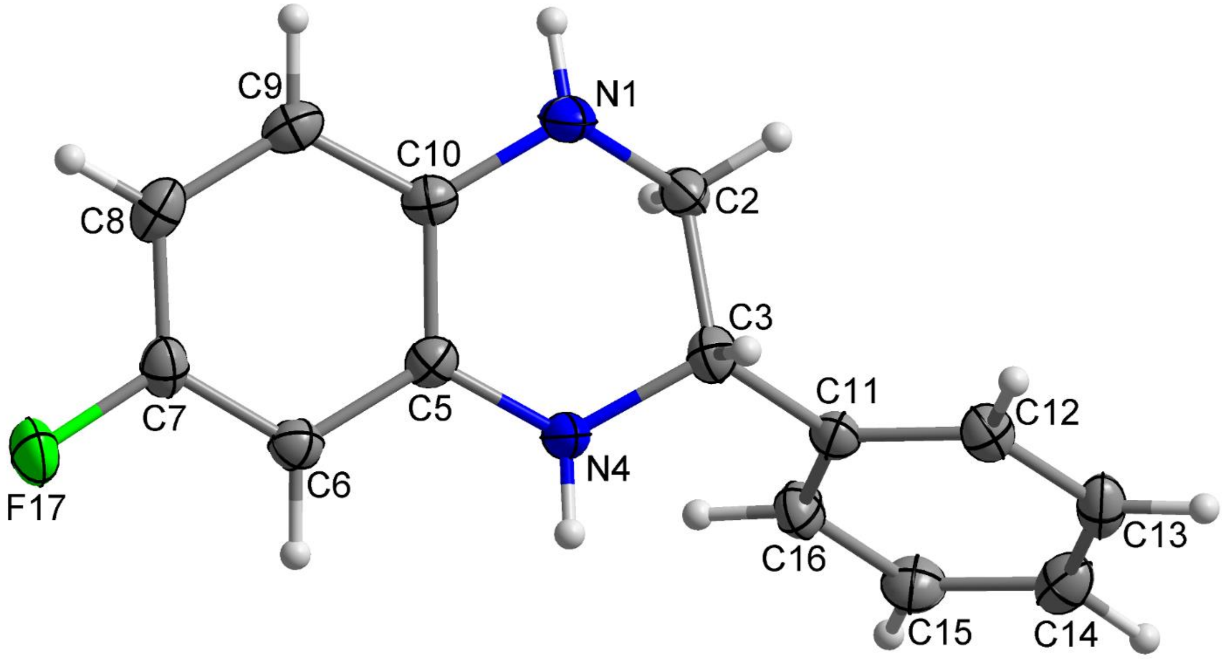

The crystal structure *S*-**4A**, the ADPs drawn at 50% probability level.

Crystallographic data for **11b**

M = 314.39 g.mol^{-1}, monoclinic system, space group $P2_1/c$, *a* = 15.3156 (3) Å, *b* = 15.0662 (3) Å, *c* = 7.05713 (14) Å, *β* = 91.6862 (18)°, *Z* = 4, *V* = 1627.72 (7) Å^3, D_c = 1.283 g.cm^{-3}, *μ*(Cu-Kα) = 0.63 mm^{-1}, crystal dimensions of 0.43 × 0.30 × 0.07 mm. Data were collected at 180 (2) K on a Rigaku OD Gemini Atlas S2 CCD diffractometer with mirror collimated sealed X-ray tube (Cu-*Kα* radiation). The structure was solved by charge flipping methods and anisotropically refined by full matrix least squares on F squared using the CRYSTALS to final value *R* = 0.034 and *wR* = 0.097 using 2921 independent reflections (θ_{max} = 67.5°), 222 parameters and 4 restrains. The hydrogen atoms bonded to carbon atoms were placed in calculated positions and refined with a riding constrains, while hydrogen atoms bonded to nitrogen were refined with restrained geometry. MCE was used for visualization of electron density maps. The structure was deposited into Cambridge Structural Database under number CCDC 2505537.

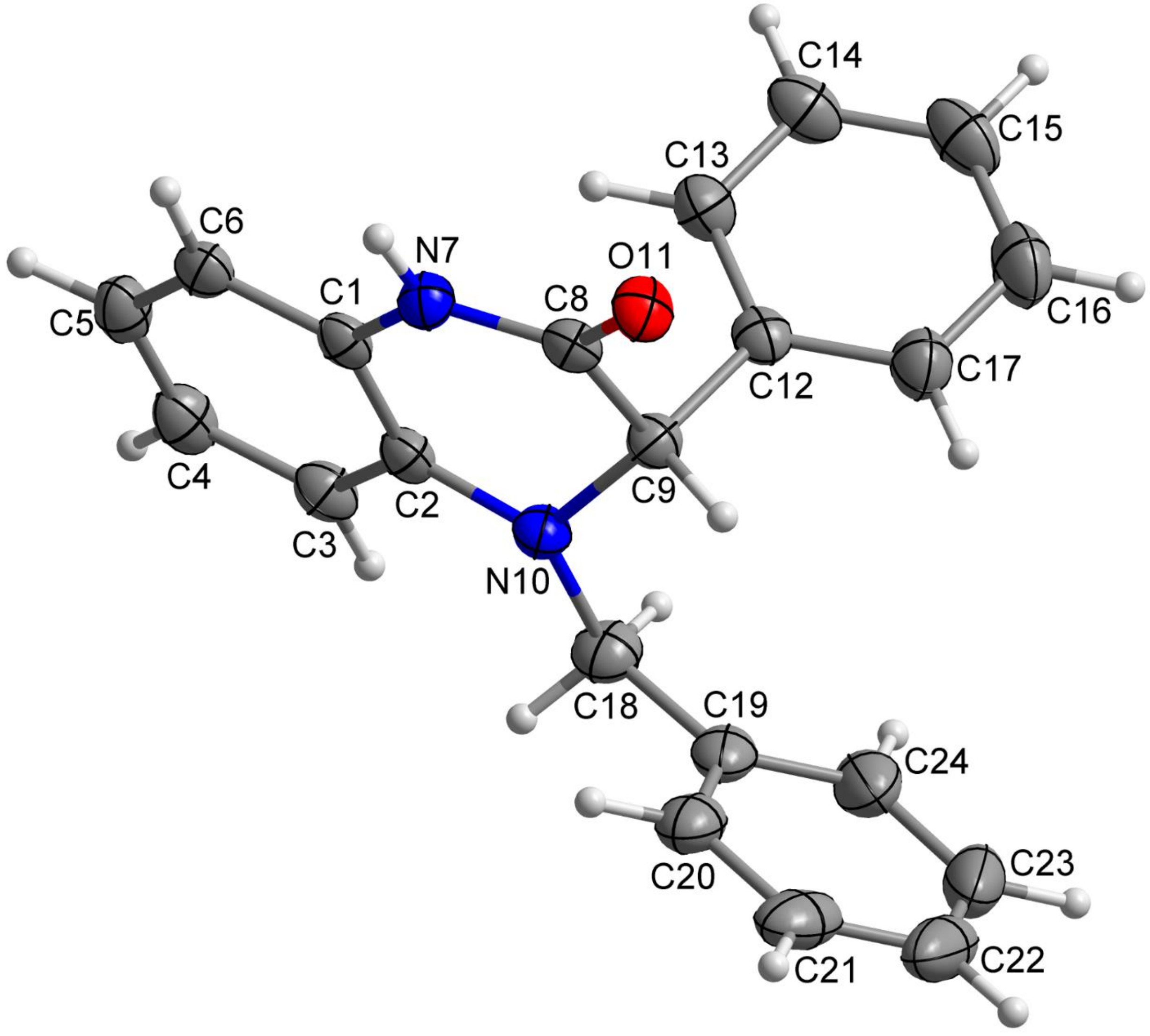

The crystal structure **11b** the ADPs drawn at 50% probability level.

Figure S154. Crystalographic data and structures of compounds structure of compound *R*-**4A**, *S*-**4B**, **11b**.

4. HPLC/UPLC data

The prepared compounds were after chromatographic purification checked for purity by UPLC/MS, selected examples are provided below, covering compound **1**, separated isomers **3c**,**4c**, *N*-alkylated **7b**, *N*-dialkylated **7a** and **8a** and *N*-sulfonated **9a**. Data for **4b** before separation to enantiomers are also attached. The separation of enantiomers is shown.

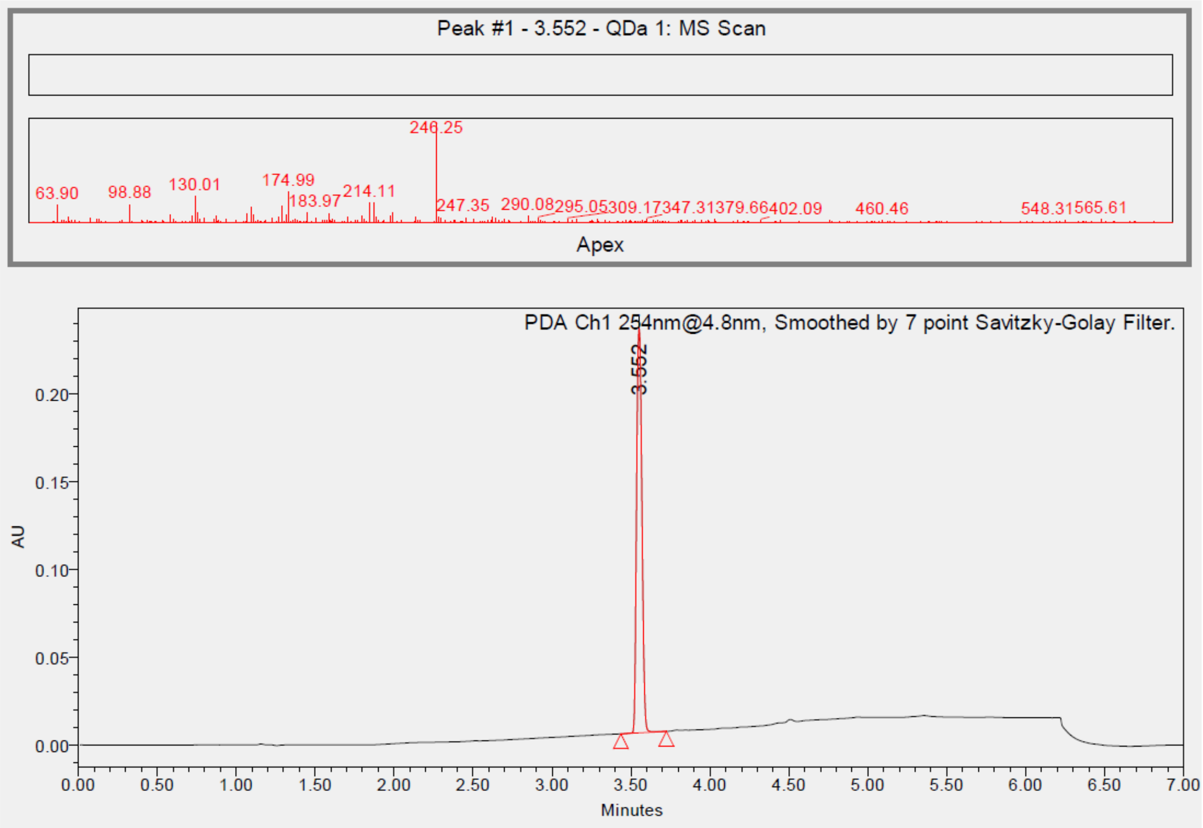

Figure S155. UPLC/MS data for **1**.

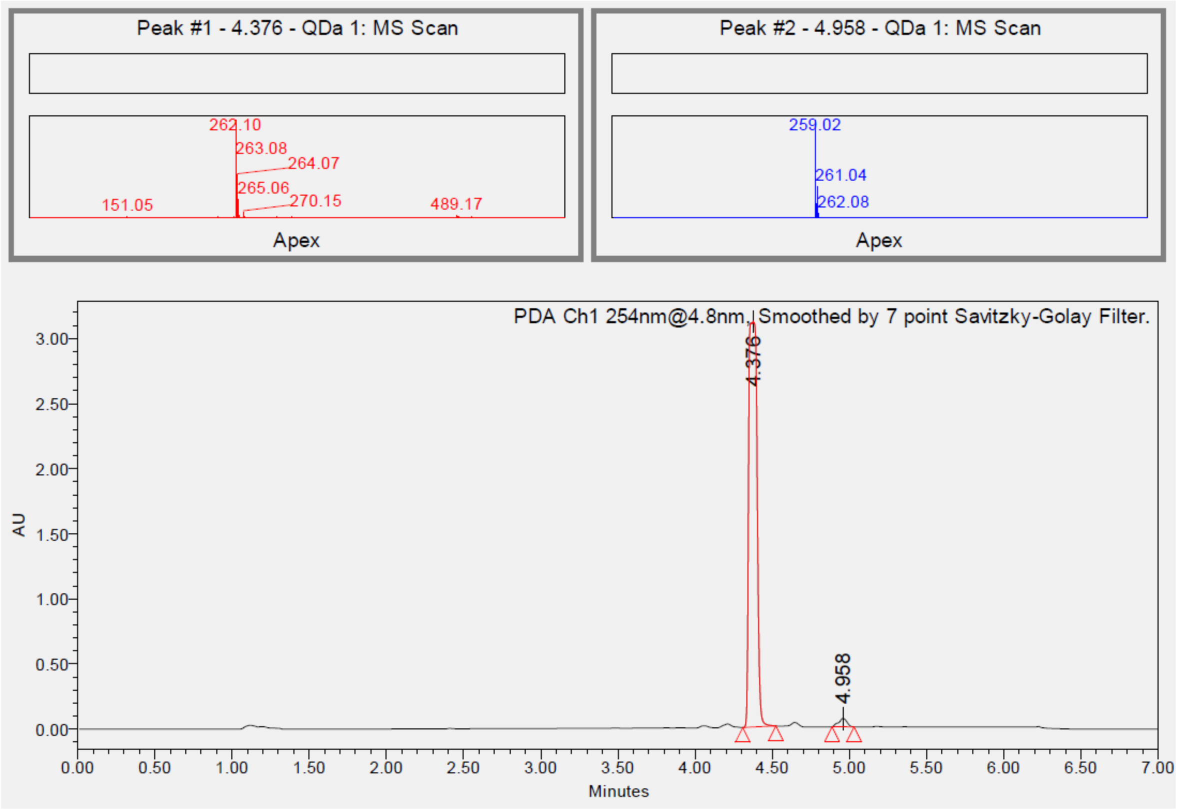

Figure S156. UPLC/MS data for **3c**.

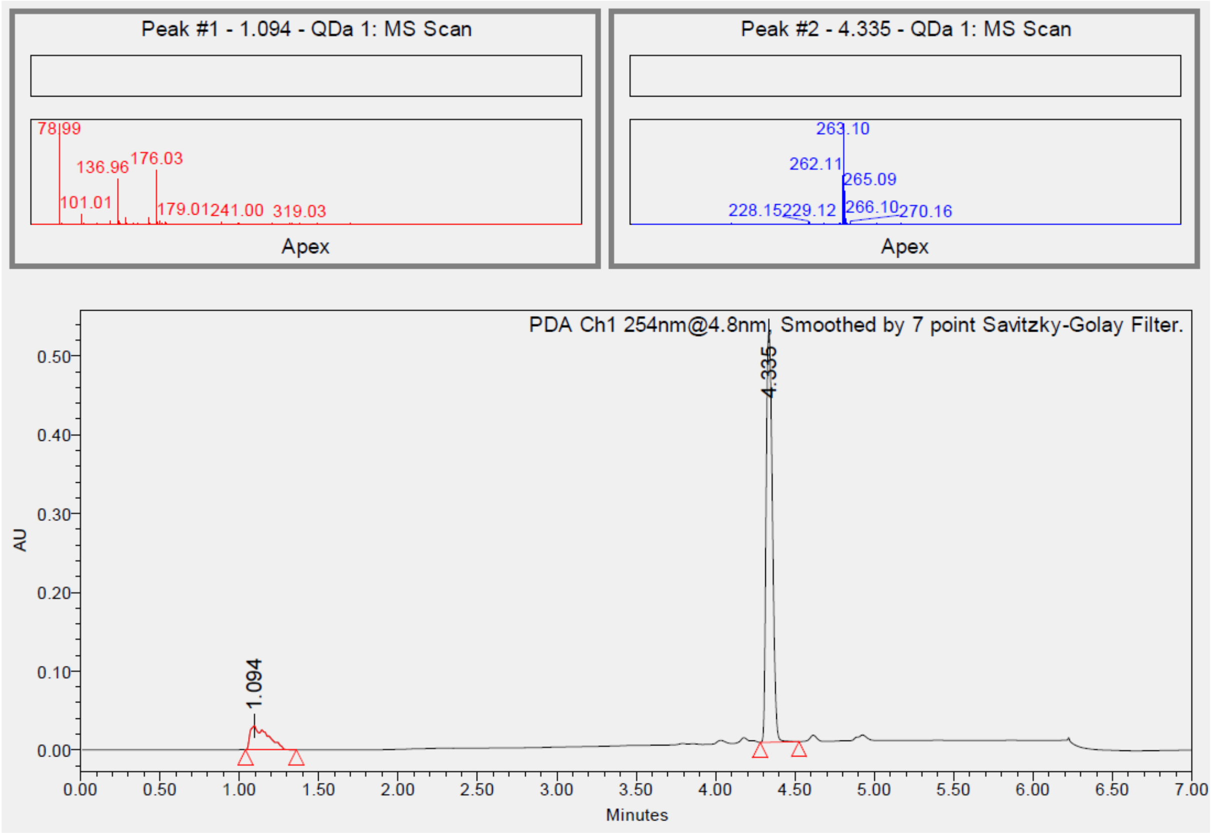

Figure S157. UPLC/MS data for **4c**.

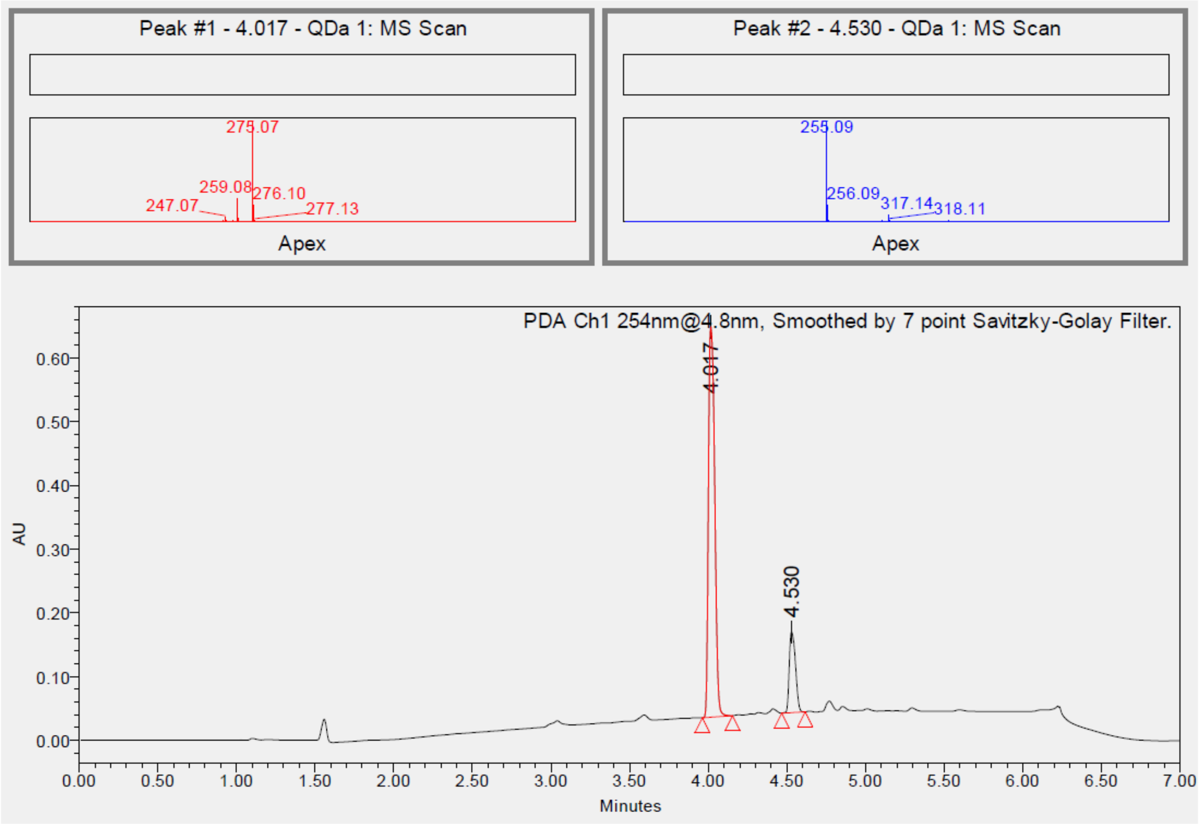

Figure S158. UPLC/MS data for **7a**.

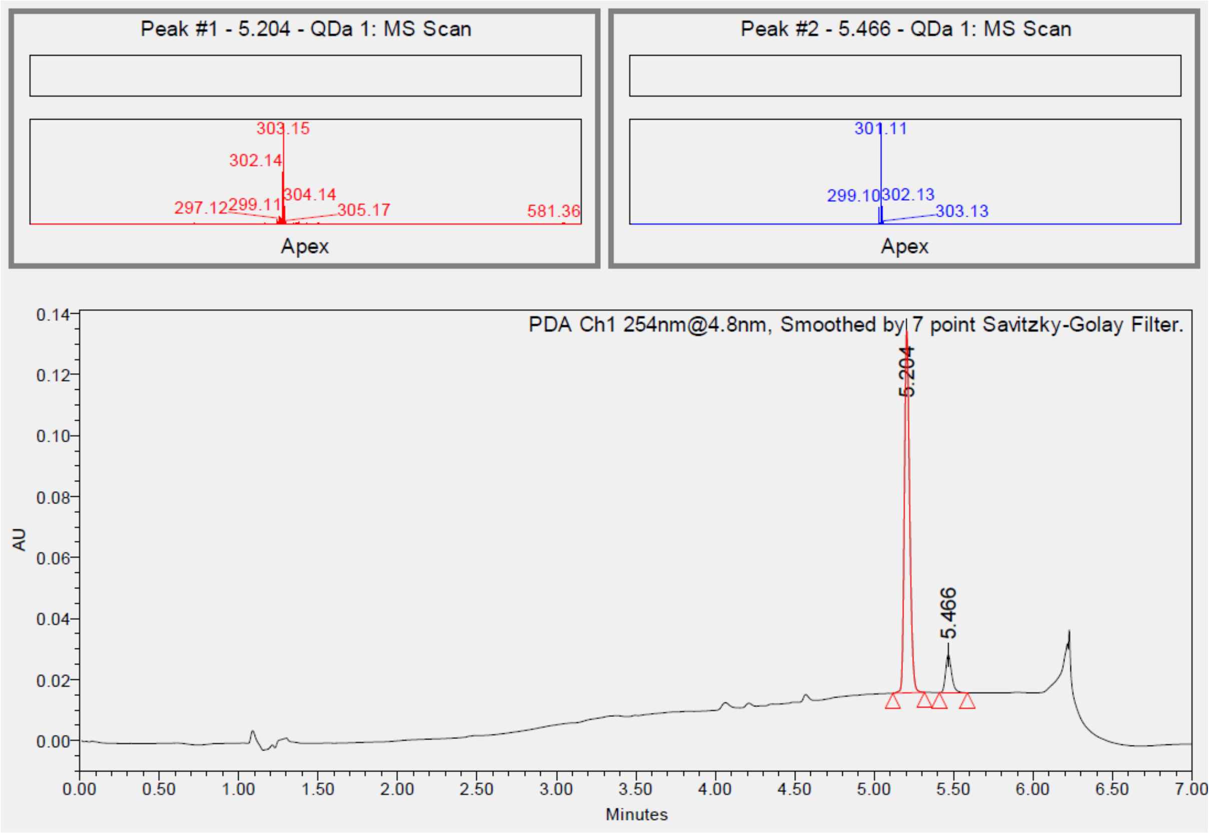

Figure S159. UPLC/MS data for **7b**.

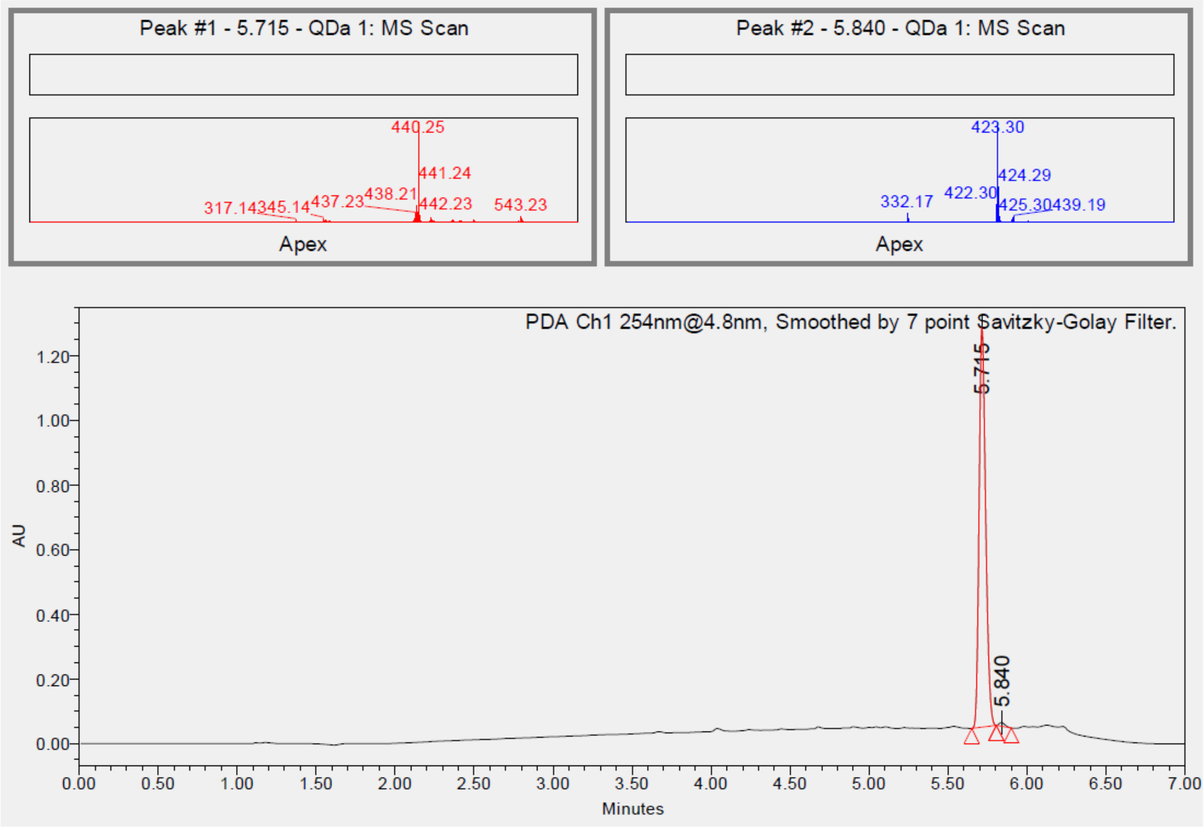

Figure S160. UPLC/MS data for **8a**.

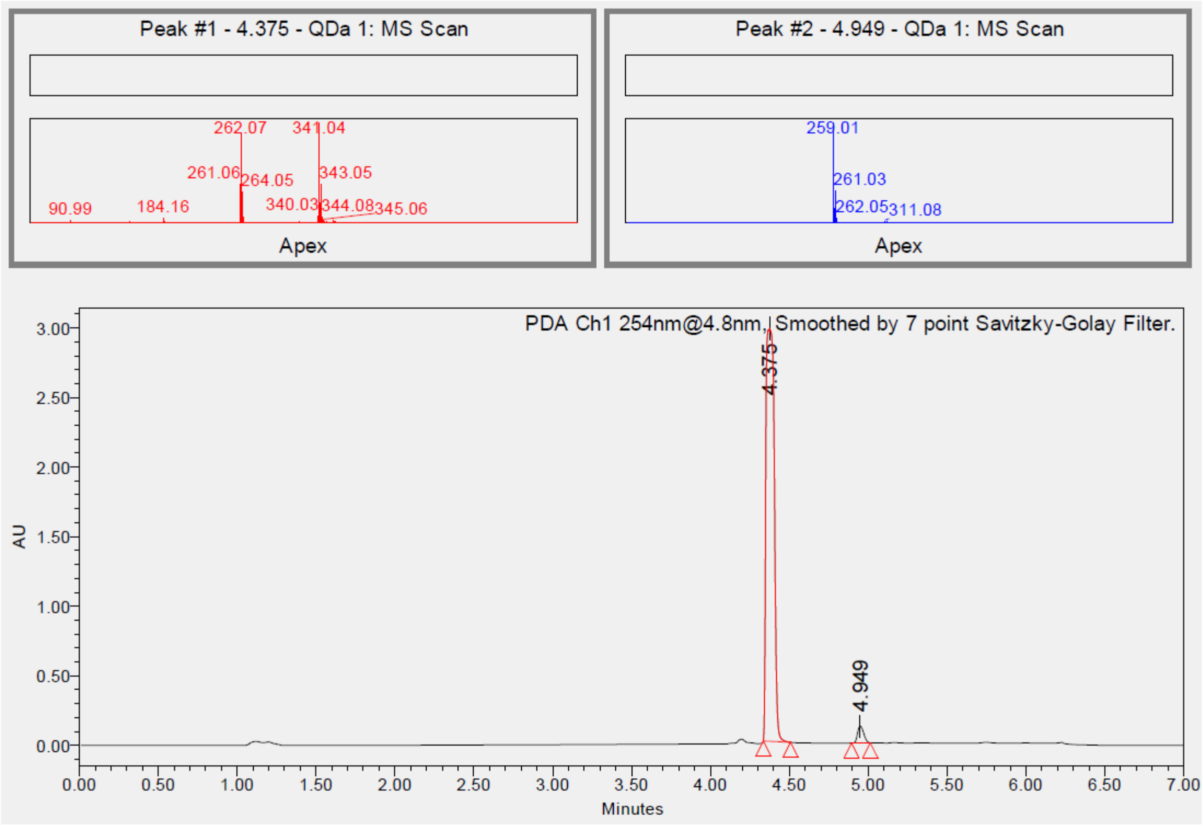

Figure S161. UPLC/MS data for **9a**.

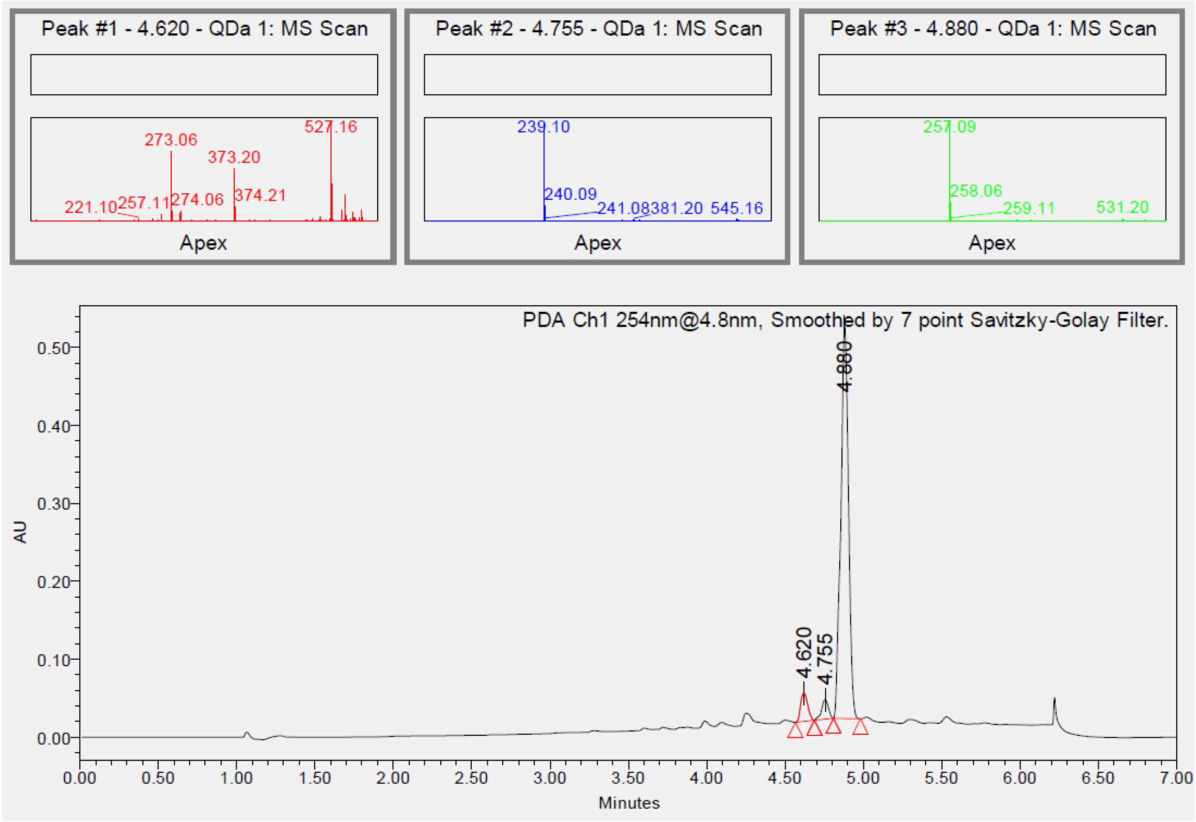

Figure S162. UPLC/MS data for **4b,** racemate.

Prep

Method Name:
Run Name: Michal/2023-04-27_17-14-35 PETRA C
Run Date: 2023-04-27 17:19

Column: Chiralpak IA	Solvent A: Hexane	UV Threshold: 0.01 AU	Collection: Collect Peaks
Flow Rate: 15 mL/min	Solvent B: i-Propanol	UV Sensitivity: Low	Per-Vial Volume: 25 mL
Equilibration: 3.0 min	Solvent C: Empty	UV1 λ: 254 nm	Non-Peak Volume: 25 mL
Run Length: 15.0 min	Solvent D: Empty	UV2 λ: 265 nm	
Mode: Prep	Slope Detection: Off	UV3 λ: 280 nm	
Sample type: Liquid		UV4 λ: 320 nm	
		UV scan start λ: 254 nm	
		UV scan end λ: 400 nm	

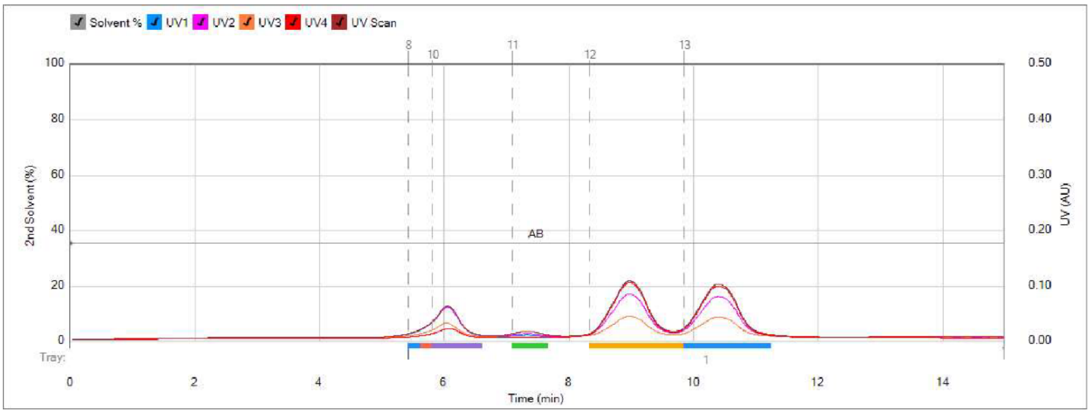

Figure S163. Compound **4a**, chiral separation, 6 min -4a-Ar, 9 min-R-4a, 10.5 min S-4a.

Prep

Method Name:
Run Name: Michal/2023-04-28_10-06-54 PETRA C - FMe6
Run Date: 2023-04-28 10:12

BUCHI

Column: Chiralpak IA
Flow Rate: 15 mL/min
Equilibration: 4.0 min
Run Length: 17.7 min
Mode: Prep
Sample type: Liquid

Solvent A: Hexane
Solvent B: i-Propanol
Solvent C: Empty
Solvent D: Empty
Slope Detection: Off

UV Threshold: 0.01 AU
UV Sensitivity: Low
UV1 λ: 254 nm
UV2 λ: 265 nm
UV3 λ: 280 nm
UV4 λ: 320 nm
UV scan start λ: 254 nm
UV scan end λ: 400 nm

Collection: Collect Peaks
Per-Vial Volume: 25 mL
Non-Peak Volume: 25 mL

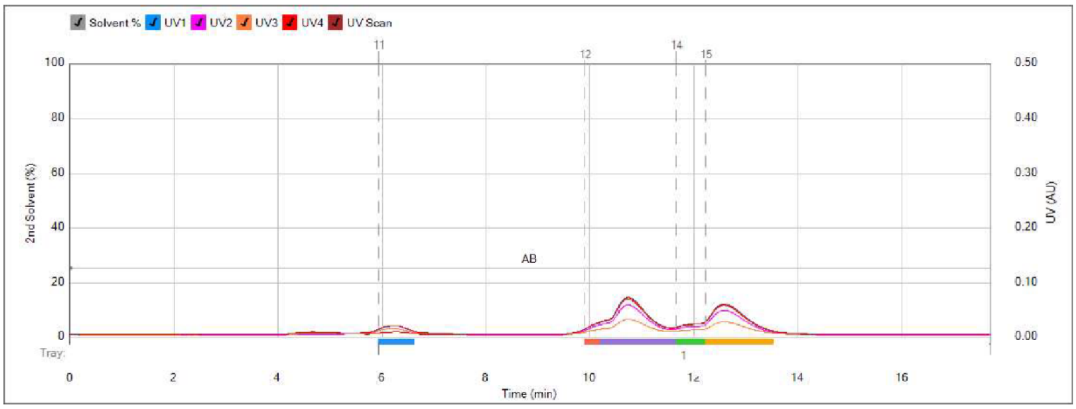

Figure S164. Compound **4b**, chiral separation, 6.5 min -4b-Ar, 11 min-R-4b, 13 min S-4b.

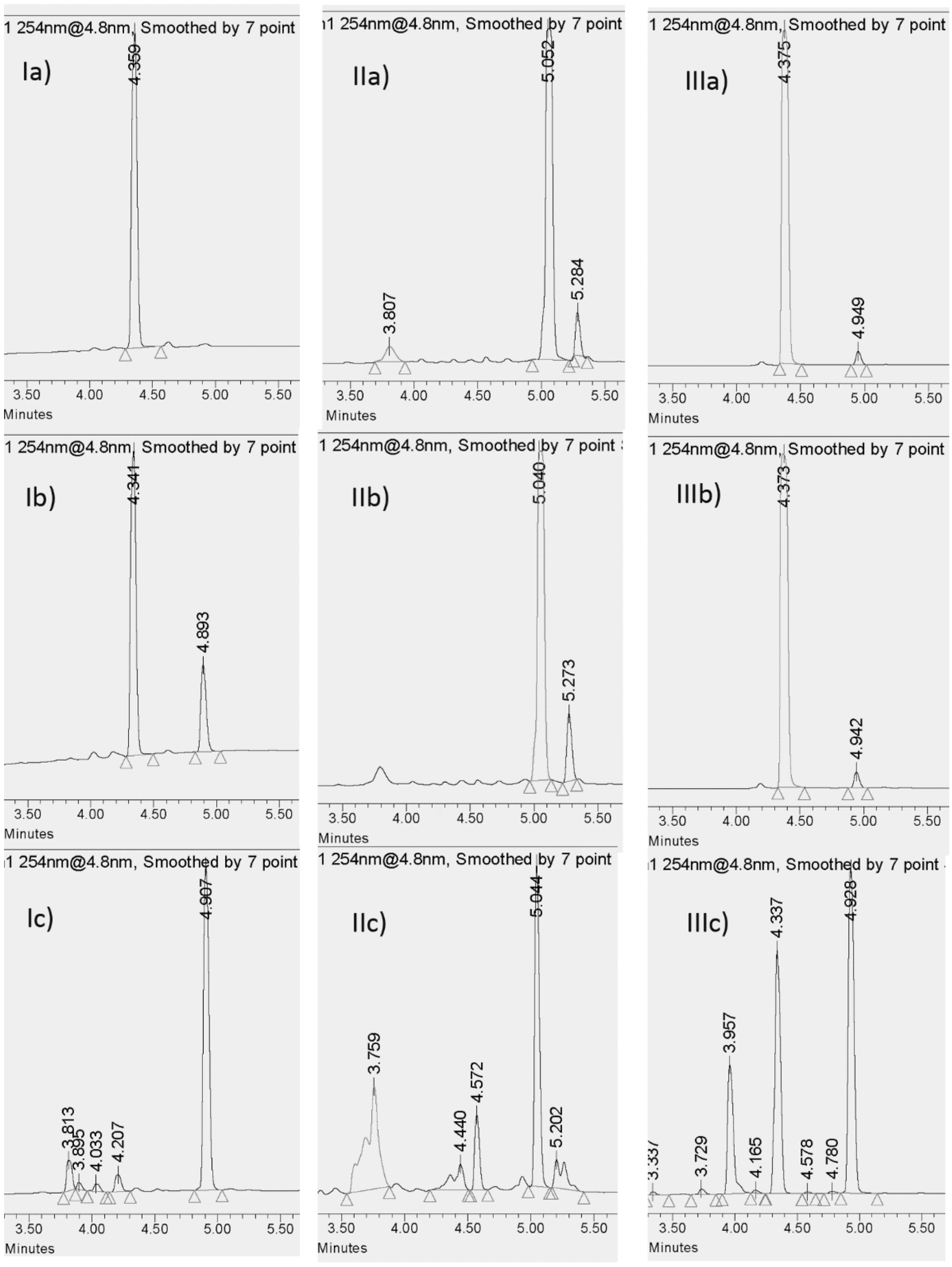

Figure S165. Stability of **3c** (I), **7d** (II), and **9a** (III) to aromatisation; a) without treatment, b) addition of HCl, 24h, c) addition of NaH, measured immediately.

5. Cell assays

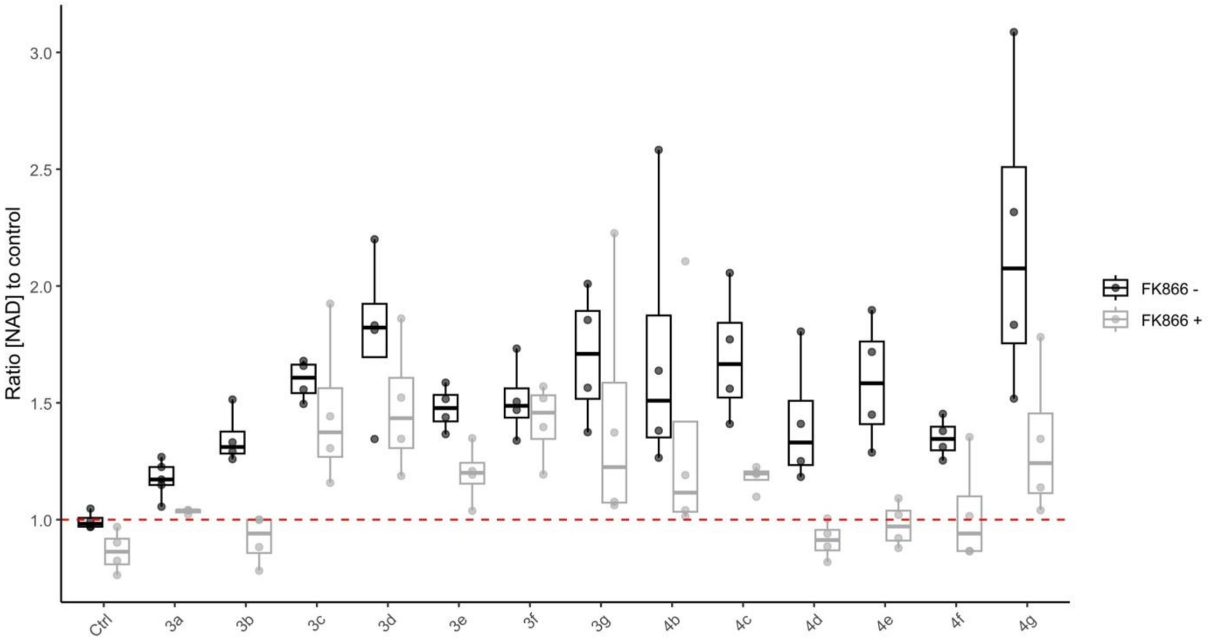

Figure S166. Fold change in NAD in primary cortical neurons incubated with FK866 100 µM for 1 hour and with compounds for 2 hours at 5 µM.

6. DMPK results

**Molecule**			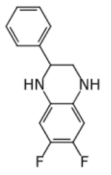 1			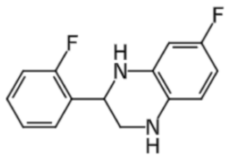 4f			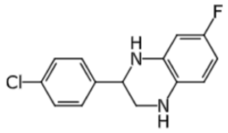 4c		
**log P**	**pKa**	**Exact mass (g/mol)**	2,4	4,4	246,0969	1,9	2,1	246,0969	2,9	3,9	262,0673
Batch Name	**Batch ID**		001	UU_BY7151001		001	UU_BY7151002		001	UU_BY7151005	
**Solubility Kinetic: Solubility Kinetic (µM)**			34,9			20,3			8,9		
**Met Stab MLM**											
t1/2 (in vitro, min)	Clint (in vitro, µL/min/mg)	E (in vitro)	3,85	359,8	0,94	9,1	152,4	0,87	6,11	226,8	0,91
**Met Stab HLM**											
t1/2 (in vitro, min)	Clint (in vitro, µL/min/mg)	E (in vitro)	25,07	55,3	0,63	21,04	65,9	0,67	300	4,62	0,13
**PPB and Stability**											
fu (%)	Stability Plasma (4h, %)		3,31	77,5		6,175	87,5		1,28	72	
**Caco-2 Permeability**											
Papp A-B (1E-6 cm/s)	Efflux Ratio B-A/A-B										

**Molecule**			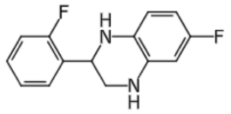 3f			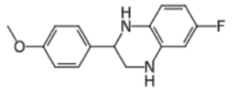 3e		
**log P**	**pKa**	**Exact mass (g/mol)**	2,1	3,8	246,0969	2,5	3,9	258,1168
Batch Name	**Batch ID**		001	UU_BY7151006		001	UU_BY7151009	
**Solubility Kinetic: Solubility Kinetic (µM)**			11,9			21,3		
**Met Stab MLM**								
t1/2 (in vitro, min)	Clint (in vitro, µL/min/mg)	E (in vitro)	11,2	123,74	0,84	2,89	479,8	0,95
**Met Stab HLM**								
t1/2 (in vitro, min)	Clint (in vitro, µL/min/mg)	E (in vitro)	300	4,62	0,13	20,35	68,12	0,68
**PPB and Stability**								
fu (%)	Stability Plasma (4h, %)		5,165	74,5		7,22	64	
**Caco-2 Permeability**								
Papp A-B (1E-6 cm/s)	Efflux Ratio B-A/A-B		97,05	0,94				

**Molecule**			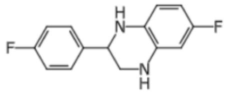 3d			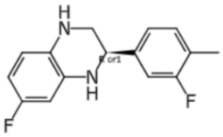 R-4b			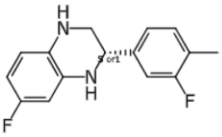 S-4b		
**log P**	**pKa**	**Exact mass (g/mol)**	2,6	3,9	246,0969	2,7	3,9	260,1125	2,7	3,9	260,1125
**Batch Name**	**Batch ID**		001	UU_BY7151010		001	UU_BY7151003		001	UU_BY7151004	
**Solubility Kinetic: Solubility Kinetic (µM)**			34,8			18,5			41,3		
**Met Stab MLM**											
t1/2 (in vitro, min)	Clint (in vitro, µL/min/mg)	E (in vitro)	12,33	112,4	0,83	2,05	676,2	0,97	1	1381,6	0,98
**Met Stab HLM**											
t1/2 (in vitro, min)	Clint (in vitro, µL/min/mg)	E (in vitro)	42,19	32,86	0,51	21,71	63,86	0,67	7,08	195,82	0,86
**PPB and Stability**											
fu (%)	Stability Plasma (4h, %)		5,07	68		1,725	74,5		1,04	68	
**Caco-2 Permeability**											
Papp A-B (1E-6 cm/s)	Efflux Ratio B-A/A-B		74,36	1,05							

Figure S167. *In vitro* DMPK results for selected compounds.

7. References

1. Loos, M.; Gerber, C.; Corona, F.; Hollender, J.; Singer, H., Accelerated isotope fine structure calculation using pruned transition trees. *Anal Chem* **2015,** *87* (11), 5738-44.
2. Palatinus, L.; Chapuis, G. SUPERFLIP - a computer program for the solution of crystal structures by charge flipping in arbitrary dimensions. *J Appl Cryst* **2007,** *40*, 786-790.
3. Betteridge, P.W.; Carruthers, J.R.; Cooper, R.I.; Prout, K.; Watkin, D.J. CRYSTALS version 12: software for guided crystal structure analysis. *J Appl Cryst* **2003**, *36*, 1487.
4. Rohlicek J.; Husak M. MCE2005 - a new version of a program for fast interactive visualization of electron and similar density maps optimized for small molecules. *J Appl Cryst* **2007**, *40*, 600.
5. Flare™, V10 , Cresset®, Litlington, Cambridgeshire, UK; https://cresset-group.com/flare/; Cheeseright, T.; Mackey, M.; Rose, S.; Vinter, A., Molecular Field Extrema as Descriptors of Biological Activity: Definition and Validation. *J Chem Inf Model* **2006**, *46* (2), 665-676; Bauer, M. R.; Mackey, M. D., Electrostatic Complementarity as a Fast and Effective Tool to Optimize Binding and Selectivity of Protein–Ligand Complexes. *J Med Chem* **2019**, *62* (6), 3036-3050; Kuhn, M.; Firth-Clark, S.; Tosco, P.; Mey, A. S. J. S.; Mackey, M.; Michel, J., Assessment of Binding Affinity via Alchemical Free-Energy Calculations. *J Chem Inf Model* **2020**, 60 (6), 3120–3130.
6. FieldTemplater™; in: Flare, V10 , Cresset®, Litlington, Cambridgeshire, UK; https://cresset-group.com/flare/; Cheeseright, T.; Mackey, M.; Rose, S.; Vinter, A., Molecular Field Extrema as Descriptors of Biological Activity: Definition and Validation. *J Chem Inf Model* **2006**, 46 (2), 665-676.
